# Musical emotions in the absence of music: A cross-cultural investigation of emotion communication in music by extra-musical cues

**DOI:** 10.1371/journal.pone.0241196

**Published:** 2020-11-18

**Authors:** Marco Susino, Emery Schubert

**Affiliations:** 1 Assemblage Centre for Creative Arts, College of Humanities, Arts and Social Sciences, Flinders University, Adelaide, Australia; 2 Empirical Musicology Group, School of the Arts and Media, University of New South Wales, Sydney, Australia; University of Otago, NEW ZEALAND

## Abstract

Research in music and emotion has long acknowledged the importance of extra-musical cues, yet has been unable to measure their effect on emotion communication in music. The aim of this research was to understand how extra-musical cues affect emotion responses to music in two distinguishable cultures. Australian and Cuban participants (*N* = 276) were instructed to name an emotion in response to written lyric excerpts from eight distinct music genres, using genre labels as cues. Lyrics were presented primed with genre labels (original priming and a false, lured genre label) or unprimed. For some genres, emotion responses to the same lyrics changed based on the primed genre label. We explain these results as emotion expectations induced by extra-musical cues. This suggests that prior knowledge elicited by lyrics and music genre labels are able to affect the musical emotion responses that music can communicate, independent of the emotion contribution made by psychoacoustic features. For example, the results show a lyric excerpt that is believed to belong to the Heavy Metal genre triggers high valence/high arousal emotions compared to the same excerpt primed as Japanese *Gagaku*, without the need of playing any music. The present study provides novel empirical evidence of extra-musical effects on emotion and music, and supports this interpretation from a multi-genre, cross-cultural perspective. Further findings were noted in relation to fandom that also supported the emotion expectation account. Participants with high levels of fandom for a genre reported a wider range of emotions in response to the lyrics labelled as being a song from that same specific genre, compared to lower levels of fandom. Both within and across culture differences were observed, and the importance of a culture effect discussed.

## Introduction

The desire to understand music’s ability to communicate emotion have long been investigated, and numerous explanations have been proposed [1, for a review see, 2]. Communication of emotion by music also occurs between listeners from different cultures [3], but little is known about the specifics of how culture affects emotion communication in music. The Cue-Redundancy Model [4] argues that emotion in music is communicated through a combination of universal and culture-specific psychoacoustic cues. According to Balkwill and Thompson [4], when perceiving emotion in music, a listener may rely on either or both of these cues to understand the musically expressed emotion. The authors explain that the degree to which a listener relies on universal cues, culture-specific cues, or both, depends on their familiarity with the music in question. A listener’s familiar with the music of a culture, such as Brazilian *Samba*, will likely rely more on culture-specific cues than a listener unfamiliar with *this music genre*. In turn, the emotion response might differ between a culturally familiar and a culturally less familiar listener. Put another way, less familiar listeners with a particular musical culture are unlikely to use culture-specific cues to understand emotion in music, and instead base their emotion response on ‘universal’ cues embedded in the music. Yet, can music communicate emotion without the need of sound, through extra-musical cues? Furthermore, would culture affect this communication?

Considerable effort has been devoted to identify ‘universal’ cues intrinsic within music to determine the communication of specific emotion [e.g., 5–9], however, considerably less research has focused on extra-musical [e.g., 10, 11] and culture-specific cues [e.g., 12–14]. Furthermore, the specifics of the cultural component remain to be understood. Learned sources of culture-specific knowledge are not bound only to psychoacoustic features (for instance tempo and timbre), but also to extra-musical cues, such as genre labels [15, 16]. It is argued, therefore, that emotion responses to music can be informed by extra-musical information detached from auditory music signals, and that this information might differ between cultures. The purpose of this research was to investigate further this emotion processing and provide a theoretical explanation.

## Emotion stereotyping as a cognitive process in music

Stereotyping refers to a categorical, generalised, socially-constructed belief about a particular agent, be it a culture, a fan group, or even a music genre [17, 18]. Researchers in music have been intrigued with the effects of stereotyping [19] and scholars have investigated musicians’ and music fans’ personality trait stereotypes [20–22], gender-instrument association, and effects of music and music videos on gender and sex stereotypes [23, 24] as well as racial stereotypes, as has been attributed to Hip Hop music and culture [25].

More recently, stereotyping has been proposed as a predictor of emotion in music. The Stereotype Theory of Emotion in Music [26], STEM hereafter, contends that some music genres may activate an emotion due to a stereotype based trigger. STEM proposes that emotion in music depends in part on the cultural stereotype associated with the music. For example, if a listener holds a stereotype of Japanese culture as calm and spiritual, the same listener might apply this generalization to Japanese music, such as *Jōruri*, even if the composer or musician is expressing a different emotion. In this example, the same listener is unlikely to associate vastly opposing emotions to the stereotype, such as anger or disgust in addition to the stereotyped emotions calm and spiritual.

STEM was empirically tested by investigating how music activated an emotional stereotype in listeners [26]. Participants were asked to report spontaneous responses to each of eight short song samples, each from distinct music genres. The eight genres investigated were *Fado*, *Koto*, Heavy Metal, Hip Hop, Pop, *Samba*, *Bolero*, and Western Classical. It was found that for all genres but *Fado*, participants with low familiarity consistently linked a small number of stereotyped emotions with the emotions evoked when considering the cultures associated with these genres. For instance, Heavy Metal culture and Heavy metal music were stereotyped as expressing negative emotions, particularly anger. Previous research corroborates this finding and suggests that fans of Heavy Metal are stereotyped as negative and violent [27]. Moreover, STEM makes more specific predictions for different levels of familiarity. Listeners with high familiarity are less likely to stereotype than less familiar listeners because they have more refined mental representations of the stimulus being perceived [28, 29]. And, those with no experience of a specific music genre will less likely stereotype because an association has not been formed between the music and cultural stereotype. Instead, psychoacoustic cues will be used to determine the emotion in the music.

Another study tested for emotional stereotypes in music when controlled for lyrics [16]. This study focused on Heavy Metal and Hip Hop music, often stereotyped as negative [30]. Participants were randomly assigned to rate two Pop (control) and two Heavy Metal (test) excerpts using identical lyrics, or two Pop (control) and two Hip Hop (test) excerpts, again using identical lyrics. After each excerpt, participants were asked to choose which emotion, if any out of a pre-determined list of eight options including ‘no-emotion’, they perceived the music to express. Results revealed that both Heavy Metal and Hip Hop music expressed more negative emotions than Pop music, even though the excerpts had identical lyrics, and despite the emotional content of the lyrics based on sentiment analysis was positive or neutral. These responses were made by listeners with average to low levels of fandom, corroborating with other studies investigating Death Metal music fandom [31]. Pop music was not perceived as expressing significantly more negative, or other emotion categories, suggesting it was not emotion stereotyped. It was concluded that these results were influenced by the listeners’ stereotyping Heavy Metal and Hip Hop, consistent with STEM.

While the studies reported above provide empirical support of stereotype effects on emotion communication in music, some variables that may affect the extent to which stereotyping occurs are still to be investigated. First, it remains an open question whether the emotion responses in these studies are due to extra-musical cues or triggered by means of psychoacoustic features. In other words, although a stereotype effect was observed in both studies, it is unclear if these are unrelated to the musical features, such as distorted guitars in Heavy Metal. One way to control for the effect of psychoacoustic cues on emotion is to manipulate them—such as, to change happy-sounding musical features (fast tempo, major mode, etc.) to sound sad [32–34]. However, this would produce an inferior design because the music may concurrently change its identity, no longer ‘sounding like’ the target genre. A more radical approach would be to remove the musical features altogether and to invoke the genre using extra-musical cues, such as providing a listener with a lyric combined with a genre label using a written cue without sounded music. If stereotyping does not occur, it would be expected for lyrics combined with any genre label or without one to communicate the same emotions. Second, a matter of interest would be whether emotion stereotypes and extra-musical cues apply to the same extent across different cultures and different levels of fandom with the music.

## Aims

The aim of this study was to assess the extent to which emotion stereotypes in response to music are affected by extra-musical cues. A specific hypothesis was tested to inform our aim: Music genre label influences the emotion that song lyrics convey.

Furthermore, we explored this hypothesis for both cultural and fandom effects. Two exploratory positions were formed. First, as stereotypes are culturally learned schemata, it was proposed that a number of differences would be observed from two culturally distinct groups.

Second, based on STEM [18, 35] it was argued that more emotion stereotyping would be expected for participants with low and average fandom.

## Materials and method

### Particpants

All included studies in this paper received an ethics approval as part of the Meaning-Making and the Perception of Music Project (UNSW Human Ethics Approval HC16942). All participants provided informed written consent. No minors were included in these studies. Data were anonymised prior to analysis. A convenience sample of 276 participants were recruited from Australia and Cuba (153 females, 123 males), between the ages of 18 and 66 years (mean = 24, *SD* = 8.5). Of these, 172 were recruited in Sydney, Australia (113 females, 59 males, age range = 18–28 years, mean = 20.8, *SD* = 2.1), and 104 in Havana, Cuba (47 females, 57 males, age range = 18–66 years, mean = 30.5, *SD* = 12.2). All Australian participants were undergraduate students at a university in Sydney, while all Cuban participants were recruited via word of mouth throughout the course of fieldwork in Cuba.

### Stimuli

Music stimuli were used for Phase 1—Genre Awareness (see Procedure). Two, 20 to 30 seconds long exemplars were used for each genre. These stimuli were recommended as prototypical by two expert musicians (for a definition of musician, see [36]) in their respective genre, each with over 20 years of professional experience and/or represented in an official Billboard music chart specific to this genre. Noticeably, the two stimuli for each genre were melodically, harmonically and rhythmically different of each other, to invoke as much as possible realistic differences in a single genre.

Stimuli used in Phase 3, Lyric-condition combination phase comprised eight lyric excerpts each taken from one of eight different music genres (see S1 Table). Of these stimuli, three were originally written in English (Heavy Metal, Hip Hop and Pop), two in Portuguese (*Bossa Nova* and *Fado*), one in Italian (*Western Opera*), one in Japanese (*Gagaku*) and one in Spanish (*Bolero*). Following certified professional translation, non-English stimuli were translated into English and presented to Australian participants. The same English translations and English original stimuli were professionally translated into Spanish and presented to Cuban participants. All translations were professionally back-translated.

### Sentiment analysis of lyrics

To understand the nature of the emotion expressed by the lyrics, sentiment analysis software was used to assess the prevalance of *positive*, *neutral* and *negative sentiment*, generated by iFeel Sentiment Analysis Framework (http://blackbird.dcc.ufmg.br:1210). iFeel documents the output of 16 computerised sentiment analysis tools, and categorizes each of these outputs into positive, negative or neutral sentiment ‘polarity’ [37]. S1 Table summarises the sentiment polarity counts for both English and also their Spanish translation. Overall, the sentiment polarity between the English lyrics and their Spanish translation were similar for each of the eight sets of lyrics.

### Procedure

For Australian participants, responses were collected using an online survey designed using Key Survey software (www.keysurvey.com). Due to lack of availability of internet in Cuba, the survey was presented to Cuban participants in hardcopy form for completion by pen and the excerpts were played by the author MS using a Sonos One loudspeaker. For Australian participants, the order of the excerpts were randomised and for Cuban, counterbalanced (see S1 Table for order of presentation).

**Fig 1:**
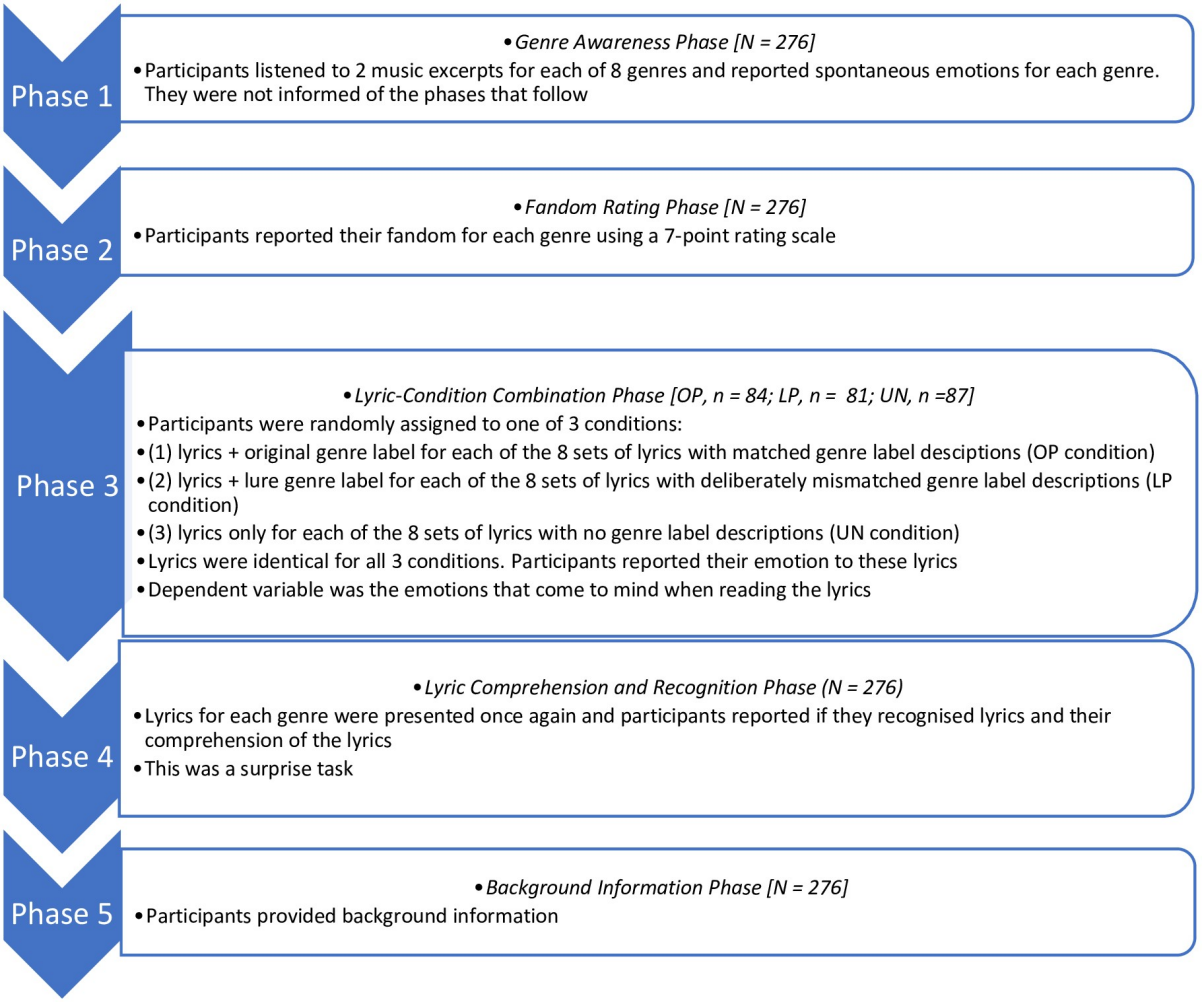
Flow chart of the 5 phase design.

The experiments consisted of five phases: 1. Genre awareness phase (phase 1); Fandom rating phase (2); Lyric-condition combination phase (3); Lyrics comprehension and recognition phase (4), and Background information phase (5). Fig 1 shows a flow chart summarising the procedure.

In the genre awareness phase 1, emotion response to genre using music excerpts were collected. Participants were asked to listen to two exemplars for each genre investigated, with each of the eight genres presented in different random order for each participant in Australia, and counterbalanced in Cuba (see S2 Table). The participants’ instructions were: ‘Please listen to these short excerpts EXACTLY ONCE EACH. The excerpts are examples of [music genre]. What is the first emotion that comes to your mind when you hear this music genre (not necessarily these two pieces)? Please provide just one word. There is no right or wrong answer’. This is the only time where music was played to the participants.

In the fandom rating phase 2, participants were asked to rate their fandom for each of the eight music genres with the instruction, ‘Please indicate the extent to which you agree that you are a fan (that is, a keen follower) of the music’ using a 7-point rating scale from 1 (Strongly Disagree) to 4 (Neither Agree nor Disagree), to 7 (Strongly Agree):. A further option, ‘I don’t know this music’, was also available.

For the lyric-condition combination phase 3, the music genre factor consisted of three conditions, original priming (OP), lure priming (LP) and unprimed (UN) distributed randomly or counterbalanced as a between participants factor. OP condition refers to lyrics (stimuli) presented to participants belonging to their original genre. LP condition refers to lyrics presented to participants belonging to a different genre than their original lyrics; for example, lyrics from a *Western Opera Aria* introduced as being from a Pop piece (See S1 Table). For the UN condition only the lyric excerpt was presented without any indication of the genre to which the lyrics belong. Self-reported emotion responses were recorded for each of the eight stimuli.

The three conditions (OP, LP and UN) in the genre label priming test (phase 3) were distributed between-participants. For conditions presented as OP and LP, participants were primed with either the original genre the lyrics were taken from or with a deceptive genre label: ‘Below are the lyrics from a [label for original genre/label for lure genre] excerpt. What is the first emotion, if any, that comes to your mind when you read these lyrics? Please provide just one word. There is no right or wrong answer.’ For UN presented conditions, no genre label priming information was presented: ‘Below are the lyrics from a music excerpt’ followed by the same instruction used for the other two conditions.

In the lyrics comprehension and recognition phase 4, the lyrics for each genre were presented once again one by one, and participants were asked to answer with yes, no or not sure as to whether they were familiar with the song from which the lyric excerpt was taken. Participants were also asked to rate what percentage of the words they understood, along a scale from 0 to 100 in increments (0% to 10%, 11% to 20%, 21% to 30% through to 91% to 100%). If they answered yes or not sure, they were instructed to name or guess the song. In the background information phase 5, details including sex and age were collected.

## Results

### Data pre-processing for emotion responses

To prepare data for subsequent analysis, participants’ open-ended emotional responses were pre-processed for both the genre awareness phase 1 and lyric condition combination phase 3 using eight data steps, based on Augustin, Wagemans [38] and Susino and Schubert [26]:

1. Correction of spelling errors.
2. Extraction of task-related parts from a sentence. For example: “Melancholy and longing” was coded as one count of ‘melancholy’ and one count of ‘longing’.
3. Removal of function words (articles and pronouns).
4. Removal of qualifiers. For example, “a little nostalgic” was coded as ‘nostalgic’.
5. Consolidating verbs (such as, joy, joyous).
6. Consolidating words that have the same stem (i.e., part of a word). For example, peace and peaceful have the same stem, ‘peace’.
7. Participants’ emotion responses were identified by author MS using these steps and checked by author ES.

Responses where the lyrics were recognised in the lyrics comprehension and recognition phase 4 were removed. Two Australian participants correctly identified the Heavy Metal excerpt used in Phase 3, and 72 Cuban participants identified the lyrics in the *Son* music excerpt used in the same phase. These responses for Heavy Metal and *Son* for these participants were removed from further analysis. The mode percentage range for words understood was 91% to 100%, confirming that with few exception, lyrics were understood by participants. No responses fell below the 71% to 80% band, and thus lyrics understood for all unidentified excerpts were retained.

### Emotion responses in the absence of psychoacoustic cues

Emotion response words reported in the lyric-condition combination phase 3 were converted into two emotion dimension scores: valence (the pleasantness) and arousal (the intensity of the emotion). This procedure allows to make an approximate, though simple and objective grouping of emotions by semantic similarity. Emotions appearing geometrically closer to each other on the two-dimensional emotion space can be treated as more similar than emotions far apart. The quantification of each emotion was based on the Affective Norms for English Words database—ANEW [39]. The ANEW database lists empirically assessed valence and arousal values, with means and standard deviations on a scale of 1 (low) to 9 (high). Nevertheless, some emotions reported by the participants were not listed in the ANEW database (e.g., nostalgia). Therefore, synonyms of these emotions were identified using the online English Oxford Living Dictionary (https://en.oxforddictionaries.com), and a synonym was selected that best matched with an entry in ANEW for which valance and arousal scores were available. S3 Table lists all terms that were matched in ANEW using a synonym.

### Data checks and preparation for phase 3

A One-way ANOVA indicated a significant mean age difference between Australian (mean = 20.8, *SD* = 2.1) and Cuban (mean = 30.5, *SD* = 12.2) participants (F(2,276) = 1.624, *p* = 0.021). Cuban participants were on average 10 years older than Australian participants. Thus, to reduce the possibility that age would conflate the results, two closely matched groups selected randomly were formed from the Australian and Cuban cohort of participants, such that participants were reasonably well matched across culture groups according to age and sex. The Australian matched group consisted of 51 participants, 27 females and 24 males, between the ages of 18 and 26 years (mean = 23.4, *SD* = 1.8). The Cuban matched group consisted of 56 participants, 26 females and 30 males, between the ages of 18 and 29 years (mean = 23.9, *SD* = 3.6). The following analysis was conducted using both balanced groups and all subjects groups.

**Fig 2:**
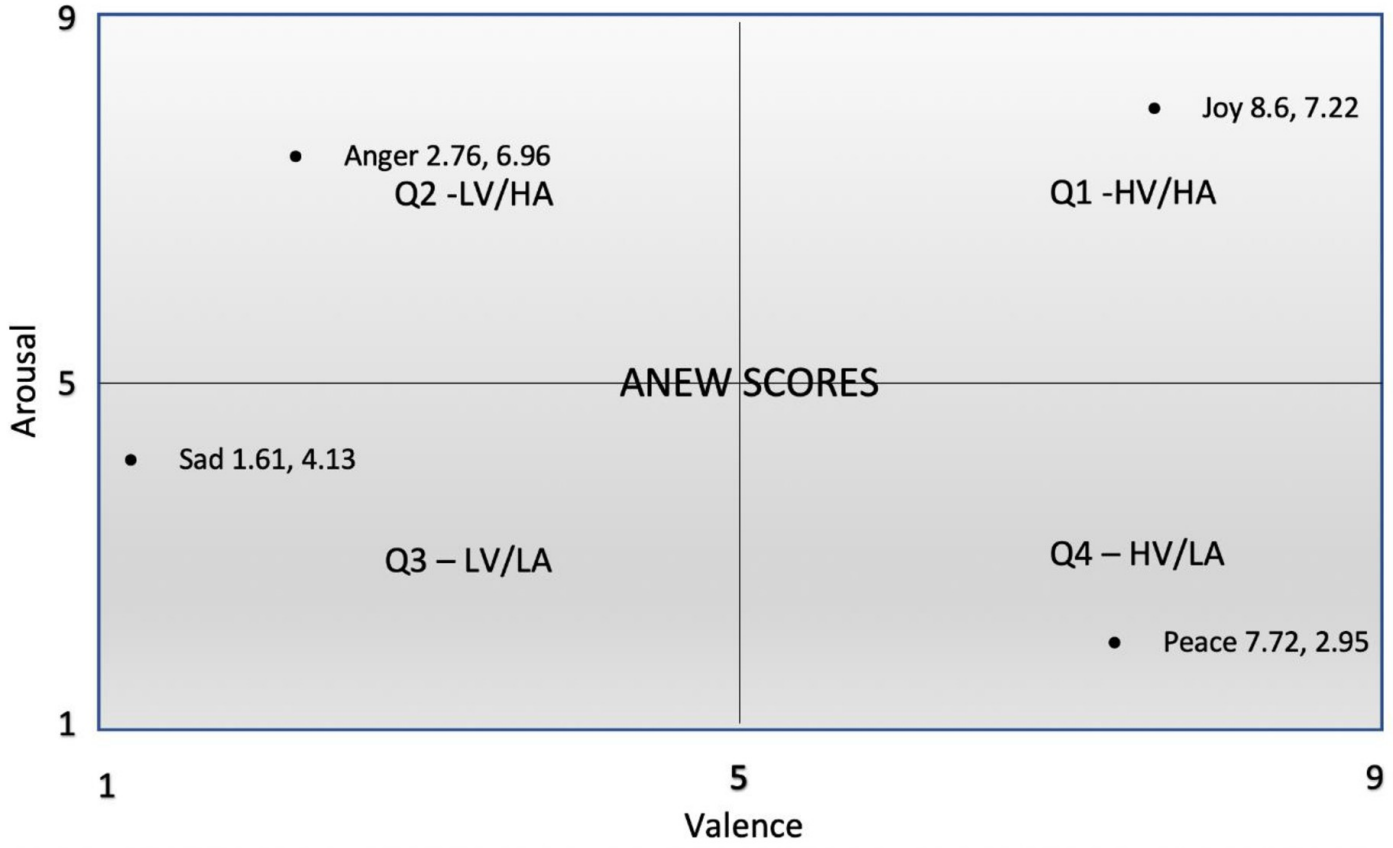
Two-dimensional emotion space showing regions used to define emotion categories described in Table 3. QX—YV/ZA refers to the rectangular region in quadrant X and its Y Valence and Z Arousal range. Valence and arousal scores of each emotion word are based on ANEW scores. Four sample emotion words are plotted, one belonging to each quadrant—Joy, Anger, Sad and Peace—for the purpose of illustration.

Emotion scores were categorised into one of four quadrants of the two-dimensional emotion space [40, 41] using the midpoint of the scale for the ANEW scores as a cut-off for each dimension. These quadrants were used for the subsequent analysis. Fig 2 provides a visual representation. To be part of, for example, Quadrant 1, High Valence/High Arousal (Q1–HV/HA), emotions would require both a valence score not lower than 5 and an arousal score not lower than 5. The number of emotions in each quadrant constituted the dependent variable. Quadrant 1 (Q1-HV/HA) represented high valence, high arousal emotions (e.g., joy, see Fig 2); Quadrant 2 (Q2-LV/HA) represented low valence, high arousal emotions (e.g., anger); Quadrant 3 (Q3-LV/LA) represented low valence, low arousal emotions (e.g., sad), and, Quadrant 4 (Q4-HV/LA) represented high valence, low arousal emotions (e.g., peace).

### Effect of genre label priming on emotional responses to lyrics

To test the hypothesis, a Chi-Square test of independence was performed to examine the relationship between genre labels and emotion responses. OP and LP counts were calculated for each quadrant. UN counts for each quadrant were used as the expected counts. This process was repeated for each of the eight genres.

Similar results were noted for both the balanced groups and all participants groups, suggesting age was unlikely to confound the results. As both balanced and all participants groups were nearly identical, the following analyses and results are for all participant groups (Table 1). Twelve out of 16 conditions (8OP and 8LP) for eight genres were significant. Two OP conditions, *Gagaku* and Pop, and two LP conditions, *Bossa Nova* and Pop did not produce statistically significant differences compared to their lyric-matching, UN stimuli. For the 12 significant conditions, emotion quadrant counts differed above chance compared to the UN distribution, supporting the hypothesis.

**Table 1:** Chi square test results for both original priming and lure priming using corresponding unprimed as control.

Condition ^a^ ^b^	Quadrant ^c^	Observed counts for each quadrant	Expected counts ^d^	Chi-square test results^+^
OP Western Opera**	Q1-HV/HA	110	80.50	(χ^2^ (3, *N* = 142) = 32.370 *p* < 0.0016, *V* = .28)
	Q2-LV/HA	9	36.89	
	Q3-LV/LA	13	12.30	
	Q4-HV/LA	10	12.30	
LP Western Opera (*Fado*) **	Q1-HV/HA	21	5.29	(χ^2^ (3, *N* = 135) = 94.449 *p* < 0.0008, *V* = .48)
	Q2-LV/HA	41	18.53	
	Q3-LV/LA	69	109.85	
	Q4-HV/LA	4	1.32	
OP *Fado* **	Q1-HV/HA	29	4.67	(χ^2^ (3, *N* = 119) = 158.085 *p* < 0.0005, *V* = .67)
	Q2-LV/HA	25	16.33	
	Q3-LV/LA	60	96.83	
	Q4-HV/LA	5	1.17	
LP *Fado* (Pop)*	Q1-HV/HA	28	24.99	(χ^2^ (3, *N* = 108) = 6.276 *p* < 0.0122, *V* = .14)
	Q2-LV/HA	63	67.70	
	Q3-LV/LA	11	12.90	
	Q4-HV/LA	6	2.42	
OP Heavy Metal **	Q1-HV/HA	14	20.34	(χ^2^ (3, *N* = 115) = 20.436 *p* = 0.0004, *V* = .24)
	Q2-LV/HA	60	69.63	
	Q3-LV/LA	41	22.68	
	Q4-HV/LA	0	2.35	
LP Heavy Metal (*Gagaku*) *	Q1-HV/HA	44	51.55	(χ^2^ (3, *N* = 107) = 11.603 *p* = 0.0158, *V* = .19)
	Q2-LV/HA	44	35.02	
	Q3-LV/LA	10	4.86	
	Q4-HV/LA	9	15.56	
OP Hip Hop *	Q1-HV/HA	19	10.53	(χ^2^ (3, *N* = 99) = 9.792 *p* = 0.0297, *V* = .18)
	Q2-LV/HA	37	44.23	
	Q3-LV/LA	41	39.31	
	Q4-HV/LA	2	4.91	
LP Hip Hop (*Bossa Nova*) **	Q1-HV/HA	25	24.01	(χ^2^ (3, *N* = 121) = 106.299 *p* < 0.0003, *V* = .54)
	Q2-LV/HA	57	65.30	
	Q3-LV/LA	28	30.73	
	Q4-HV/LA	11	0.96	
OP *Son* **	Q1-HV/HA	6	3.34	(χ^2^ (3, *N* = 51) = 19.180 *p* < 0.0007, *V* = .35)
	Q2-LV/HA	21	11.05	
	Q3-LV/LA	20	4.4	
	Q4-HV/LA	1	2.21	
DP *Son* (*Heavy Metal*) *	Q1-HV/HA	22	23.35	(χ^2^ (3, *N* = 132) = 12.461 *p* < 0.012, *V* = .18)
	Q2-LV/HA	66	79.91	
	Q3-LV/LA	42	26.04	
	Q4-HV/LA	2	2.69	
OP *Gagaku*	Q1-HV/HA	45	42.88	(χ^2^ (3, *N* = 89) = 1.089 *p* = 0.7798, *V* = .06)
	Q2-LV/HA	26	29.12	
	Q3-LV/LA	8	4.06	
	Q4-HV/LA	10	12.95	
LP *Gagaku* (Son) **	Q1-HV/HA	3	2.42	(χ^2^ (3, *N* = 63) = 29.45 *p* < 0.0003, *V* = .39)
	Q2-LV/HA	20	18.58	
	Q3-LV/LA	40	41.19	
	Q4-HV/LA	0	0.81	
OP Pop	Q1-HV/HA	21	18.50	(χ^2^ (3, *N* = 80) = 3.510 *p* = 0.3408, *V* = .12)
	Q2-LV/HA	44	50.15	
	Q3-LV/LA	14	9.55	
	Q4-HV/LA	1	1.79	
LP Pop (Western Opera)	Q1-HV/HA	71	60.09	(χ^2^ (3, *N* = 106) = 6.748 *p* < 0.1072, *V* = .15)
	Q2-LV/HA	24	27.54	
	Q3-LV/LA	8	9.18	
	Q4-HV/LA	3	9.18	
OP Bossa Nova*	Q1-HV/HA	33	19.84	(χ^2^ (3, *N* = 100) = 11.076 *p* = 0.018, *V* = .19)
	Q2-LV/HA	44	53.97	
	Q3-LV/LA	22	25.40	
	Q4-HV/LA	1	0.79	
LP *Bossa Nova* (Hip Hop)	Q1-HV/HA	20	12.77	(χ^2^ (3, *N* = 120) = 5.697 *p* = 0.1294, *V* = .13)
	Q2-LV/HA	49	53.62	
	Q3-LV/LA	48	47.66	
	Q4-HV/LA	3	5.96	

^a^ Genre source of lyrics indicated within parenthesis.

^b^ OP stands for the Original Priming genre label presented to participants. LP stands for the Lure Priming genre label presented to participants.

^c^ Q1-HV/HA, Q2-LV/HA, Q3-LV/LA, and Q4-HV/LA represent quadrants 1 high valence/high arousal, 2 low valence/high arousal, 3 low valence/low arousal and 4 high valence/low arousal respectively, of the two-dimensional (valance and arousal) emotion space (see Fig 2).

^d^ proportional adjusted UN counts for each quadrant where OP counts were compared to UN counts form the original genre and LP counts were compared to UN counts from the lure genres.

*V* is effect size (Cramer’s V)

* *p* < .05

** *p* < .005

^+^ All *p* values are Benjamini-Hochberg corrected [42]

### Characteristic emotions induced by music genre

**Fig 3:**
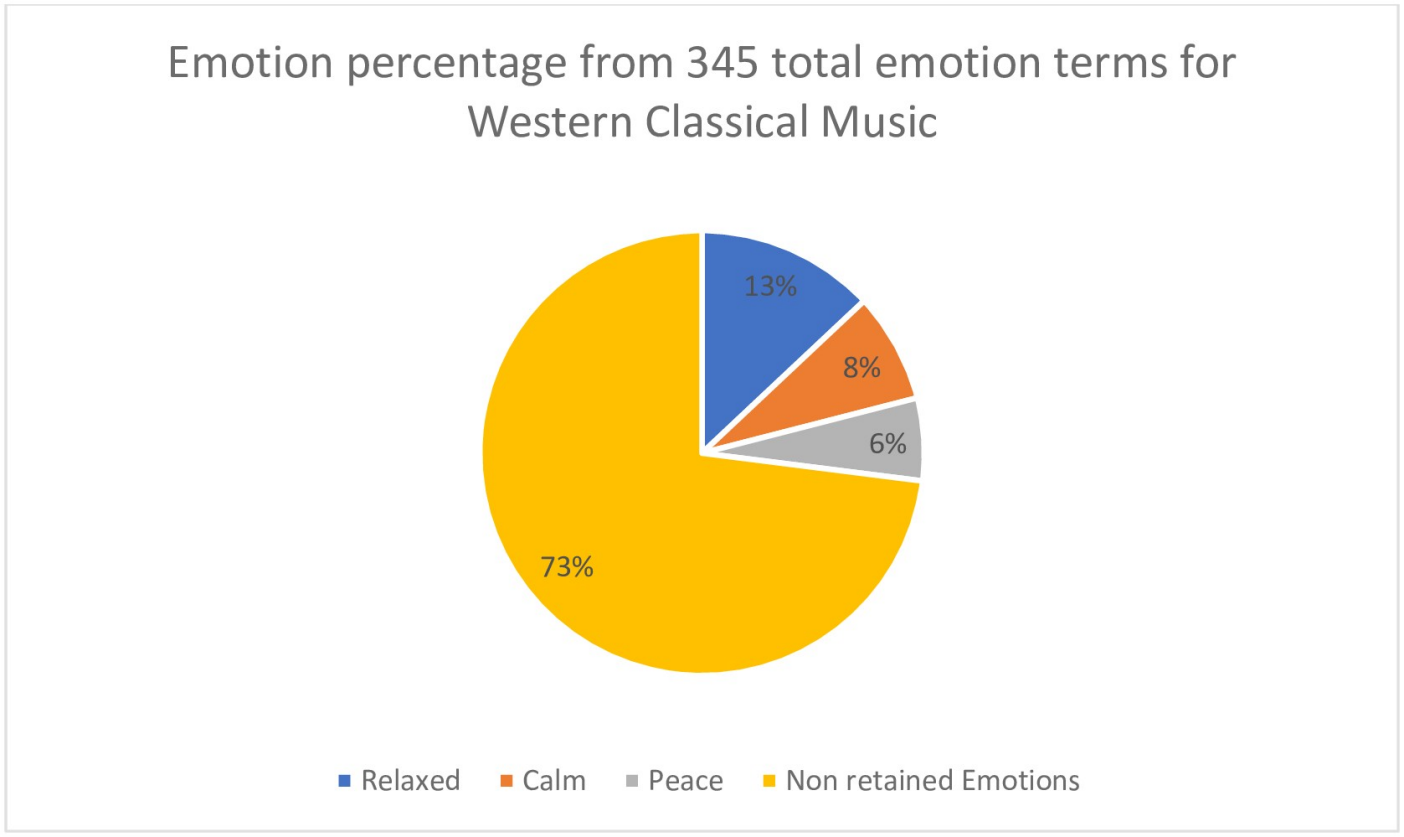
A visual represantation of the emotions reported with frequency above 5% of the total emotion words when the label Western Classical music was assigned to the lyrics.

Emotions words reported with a frequency above 5% of the total emotion words reported for each genre in phase 1 were analyzed. The 5% threshold is based on that used by Zentner, Grandjean, and Scherer [43] and Susino and Schubert [26]. Table 2 reports these results and Fig 3 provides a more detailed explanation of the source of the counts for the case of when the label Western Classical is used as the priming label. According to the 5% criterion, a total of 14 emotions were reported across genres. The most frequently emotion response was “happy”, reported 123 times across *Son*, Pop, and *Bossa Nova*. Notably, the term ‘flavourful’ was treated as an emotion, consistent with how Cubans interpret the expression, and in line with previous studies on flavour and music-induced emotions [44, 45].

**Table 2:** Emotions and respective percentages reported above 5% of the total emotion words reported for each genre label in phase 1.

Genre							
Western Classical	*Fado*	Heavy Metal	Hip Hop	*Son*	*Gagaku*	Pop	*Bossa Nova*
TOT = 345 relaxed (13%), calm (8%), peace (6%)	TOT = 352 sadness (13%), calm (7%), love (6%), relaxed (6%), romantic (5%)	TOT = 346 madness (7%), anger (5%)	TOT = 398 anger (15%), dancing (8%),	TOT = 354 happy (7%), love (7%), joy (6%), dancing (5%), flavourful (5%),	TOT = 332 peace (7%), meditation (5%)	TOT = 349 happy (12%), joy (9%),	TOT = 358 joy (16%), happy (11%), excited (5%), dancing (5%)

TOT = Total emotion words reported for each respective genre label.

See Fig 3 for a visualisation of how the words are distributed in the case when the Western Classical genre label is applied to the lyrics.

The emotions reported in the genre awareness phase 1 for each genre were compared with those observed for the same or similar genres (explained below) in a previous study [26]. For Western Classical, relaxed, calm, and peace observed in the present study (Fig 3) were also the three most observed emotions in [26]. For *Fado*, sadness, calm, romantic and love were also observed in both the present research and also [26]. Anger was the most reported emotion in both studies for Heavy Metal and Hip Hop, and so was dancing for Hip Hop and dancing and happy for Pop. The emotions reported for *Bossa Nova*, *Son*, and *Gagaku* in the present study were respectively compared with *Samba*, *Bolero*, and *Koto* in [26]. Both *Bossa Nova* and *Samba* generated high counts of dancing and happy. *Son* compared to *Bolero* produced mixed results. While in both studies, dancing, love and relaxed were three of the most frequently reported emotions reported for both *Son* and *Bolero*, romantic sad and love reported for Bolero were not observed in the present study. Instead, joy and flavourful were observed for *Son*. Lastly, a comparison between *Gagaku* and *Koto* revealed peace as a common response in both genres. However, the emotions calm, sad and tranquil observed in *Koto* were not replicated for *Gagagku*. Instead, meditation was observed in the present study. Only for *Son* was there no replication of emotions. Seven out of eight genres compared had identical or consistent emotion categories reported.

### Exploration of culture effects

To explore the effects of culture, lyric-condition combination phase 3 emotion words within and across the two cultures were inspected for each genre. S4 Table presents these results. Notable differences in priming conditions were observed in comparison to unprimed conditions for Western Opera, Heavy Metal and *Gagaku* for both cultural group, and Hip Hop for Cuban culture. Taking Heavy Metal as an example, both cultures reported negative valance, high arousal emotions in the OP condition. Australians reported anger for both primed (14%) and unprimed (15%) conditions, while Cubans reported violence (10%) and death (22%). However, no notable distinctions between cultures were observed for *Fado*, *Son*, Pop, and *Bossa Nova*.

Across cultures, differences were observed for all genres when comparing emotion words and broad emotion categories. For example, for Hip Hop, Cubans frequently reported violence (14%) while Australians frequently reported sadness (18%). Furthermore, the frequency of some emotion terms was at times inconsistent. Cuban participants reported higher counts of love for Western Opera (37%), *Son* (42%) and *Bossa Nova* (9%) and spiritual for *Gagaku* (43%), compared to Australian participants. Australian participants reported higher counts of sadness compared to Cubans, for Hip Hop (18%), *Son* (21%) and *Bossa Nova* (19%). These results support the notion that cultural differences are observed because stereotypes are culturally learned.

### An exploration of emotion responses based on fandom

To explore the proposed effects of fandom, the characteristic emotions for each genre were broken into four distinct fandom categories using responses gathered in the fandom rating phase 2: Low Fandom (fandom ratings of 1 to 3); Average Fandom (fandom ratings of 4); High Fandom (fandom ratings of 5 to 7) and No Fandom (I don’t know this music). No Fandom responses were ignored when calculating mean and standard deviation of ratings. Pop had the highest fandom rating (*M* = 5.45, *SD* = 1.66), while Heavy Metal had the lowest (*M* = 2.38, *SD* = 1.74). Emotions word frequency reported above 5% of the total emotion words for each genre in phase 1 were analyzed according to fandom scores. Table 3 reports these results.

**Table 3:** Emotions reported above 5% of the total emotion words reported for each genre label in phase 1 by fandom rating category from phase 2.

Fandom category	Genres and Respective Fandom Mean and Standard Deviation							
	*Western Classical (M = 5*.*17*, *SD = 1*.*58)*	*Fado (M = 4*.*59*, *SD = 2*.*21)*	*Heavy metal (M = 2*.*38*, *SD = 1*.*74)*	*Hip hop (M = 4*.*96*, *SD = 1*.*82)*	*Son (M = 5*.*02*, *SD = 2*.*21)*	*Gagaku (M = 2*.*47*, *SD = 2*.*62)*	*Pop (M = 5*.*43*, *SD = 1*.*61)*	*Bossa Nova (M = 4*.*49*, *SD = 1*.*76)*
*Low Fandom* ^a^	TOT: 21 calm (23.8%), bored (19%)	TOT: 72 sadness (18%), relaxed (16.6%),	TOT: 177 anger (18.6%), madness (7.3%), fear (7.3%)	TOT: 36 2**	TOT: 39 dancing (12.8%)	TOT: 66 meditation (12.1%)	TOT: 25 bored (28%)	TOT: 52 happy (15.4%), joy (9.6%)
*Average Fandom* ^b^	TOT: 17 relax (23.5%)	TOT: 40 peace (10%), nostalgia (10%), sadness (10%)	TOT: 17 anger (17.6%), excitement (11.8%)	TOT: 14 dancing (28.6%)	TOT: 29 happy (17.2%)	TOT: 22 peace (22.7%),	TOT: 21 happy (19%), (bored 13.4%)	TOT: 23 happy (21.7%),
*High Fandom* ^c^	TOT: 124 relax (21%), calm (13.7%), peace (9.7%),	TOT: 87 sadness (20.7%), calm (12.6%), love (11.5%), nostalgia (8%)	TOT: 36 excitement (25%), anger (13.9%)	TOT: 97 relax (10%), happy (10%), dancing (7.2%)	TOT: 110 joy (10.9%), dancing (10.9%), happy (10%), flavourful (8.1%)	TOT: 35 reflexive (14.2%), peace (14.2%),	TOT: 112 happy (26.4%), joy (13.2%), dancing (8%), energetic (8%), excitement* (8%)	TOT: 130 joy (28.5%), happy (9.2%), dancing (9.2%)
*No Fandom* ^d^	TOT: 2**	TOT: 25 calm (16%), sadness (12%)	TOT: 10 relief (20%), beautiful (20%), anger* (20%)	TOT: 1 2**	TOT: 18 relax* (38.8%)	TOT: 38 peace* (23.7%), reflexive (13.1%), spiritual (10.5%)	TOT: 3 2**	TOT: 10 happy* (10%), dancing* (10%)

^a^ Fandom ratings of 1 to 3 = low fandom score (strongly disagree (1), moderately disagree (2), slightly disagree (3)).

^b^ Fandom ratings of 4 (neither agree nor disagree).

^c^ Fandom ratings of 5 to 7 = high fandom (slightly agree (5), moderately agree (6), strongly agree (7)).

^d^ No Fandom = I don’t know this music.

TOT = Total emotion words reported for each respective genre.

** Small number of emotion words reported or no terms above the 5% criterion.

For participants reporting no fandom, emotion word counts reported were too low to allow analysis for Western Classical, Hip Hop, and Pop. For low fandom of Hip Hop, 36 emotions were reported though no single one was reported above threshold. Comparing high fandom with low fandom, which each cover three points of the fandom rating scale, and have a comparable number of emotion words reported across the genres, we see a total of 26 above threshold emotions reported in the high fandom group, whereas for the low fandom group only 12 emotions were reported above threshold. For example, happy was the only above 5% threshold emotion reported from average fandom listeners for *Son*, compared to joy, dancing, happy and flavourful from high fandom listeners. It is also noticeable that high fandom responses for some genres produced contrasting emotions. For example, fans of Heavy Metal reported both excitement (25%) and anger (13.9%). Similarly, fans of Hip Hop reported relax (10%), happy (10%) and dancing (7.2%). The same was observed in no fandom responses. However, this range of emotions is more limited in low and average fans responses. These observations suggest that participants with high fandom and no fandom of a genre report more varied emotions than participants with respectively low and average fandom for the same genre, lending support to the proposal that people with low and average fandom will stereotype more than people with high fandom or no knowledge.

## Discussion

### Extra-musical cues effect

Lyric-condition combination phase 3 eliminated psychoacoustic features from the emotion elicitation process through the use of the genre label priming paradigm. That is, emotion responses were triggered by extra-musical cues. Emotion stereotyping provides a logical explanation of these results. According to STEM, information about music can automatically trigger categorical, emotional expectations that are formed through a cultural interaction that is not be informed by music features. Within a specific emotion space quadrant, like the negative valance, high arousal emotions for Heavy Metal primed stimuli, or negative valance, low arousal emotions for *Fado* are systematically communicated. The most-reported emotion in phase 3 for *Fado* (sadness– 13% of 352) and Heavy Metal (anger– 5% of 346) belong to the over-represented emotion quadrants in Table 3, Q2 and Q3 respectively. Sadness is a common emotion response to *Fado* music [46] and so is anger to Heavy Metal [47]. The same applies to anger–(15% of 398) for Hip Hop, overly represented in Q2 emotions in Table 1. However, *Son* was represented in Q2 and Q3 quadrants, which are not representative of its expected Q1 (happy) emotions as observed in previous studies with similar music genres, indicating that the emotions were not stereotyped. Overall, the differences between the primed and unprimed stimuli in phase 3 suggest the genre label triggered an emotional stereotype. Four genre conditions did not produce statistically significant differences compared to their lyric-matching, unprimed stimuli. This means that in these instances, the sentiment of the lyrics in both primed and unprimed condition triggered the emotion responses, suggesting that emotion stereotyping due to genre label did not take place. Hence, it is possible that stereotyping might be more prominent for some music genres, such as Heavy Metal, Western Opera and *Gagaku* music in comparison to others, like Pop, or that other factors might be involved, such as fandom, which we discuss below.

### Genre characteristic emotions

For all of the eight genres investigated but *Son*, emotions observed in [26], compared to emotions reported in phase 1 of the current study were either identical or were broadly part of the same emotion category (for example, Western Classical represented by the emotions calm, peace, and relax). The replication occurred even though the music stimuli played to exemplify a genre across the two studies were different, thus differing in their harmonic, rhythmic and melodic features (for example, Gustav Holst’s ‘The Planets’ compared to Philip Glass’ ‘Morning Passages’ for the Western Classical stimuli). This suggests that genre information alone can reliably evoke emotions systematically, building evidence that extra-musical cues, rather than just psychoacoustic features, play a role in the communication of emotion in music. According to this scenario, Pop and *Samba*, for example, are expected to communicate positive valence, high arousal emotions in part because of the emotion stereotype they evoke; Heavy Metal and Hip Hop will characteristically evoke negative valence, high arousal emotions; *Fado* would be expected to evoke negative valance, low arousal emotions and, Western Classical and *Gagaku* are expected to evoke positive valence, low arousal emotions, overall consistent with the results in this study. The mixed results generated by comparing *Son* with *Bolero* are likely because of the cultural difference between participants in this study and previous research. Unlike participants in previous research [26, 43] which investigated emotion responses to music genres from a single culture, the current study investigated responses from two distinct cultures in which both test stimuli and, importantly, participants were recruited from the cultures from which the stereotypes are present. Based on the cue-redundancy model [4], Cuban listeners in this study would attend to culture-specific cues when listening to *Son*, which in turn facilitated the emotional recognition for the music of their own culture. This would explain why in the current study the emotions flavourful and joy collectively made up 11% of the total responses to *Son*, emotions which were not observed in Susino and Schubert [26] which investigated Australian listeners’responses only (who may not have been privy to these culture-specific cues). It is likely that Cubans relied more on culture-specific cues to respond to *Son* music, while non-Cuban participants relied on psychoacoustic cues deduced from the exemplars presented in the genre awareness phase 1. Hence, Australians might simply not have known *Son*, and so had no stereotype, but instead determined their responses by means of the psychoacoustic cues. Another explanation might be the discrepancy in fandom between Cuban and Australian listeners for *Son*, discussed below. Participants with different fandom levels have been observed to report different emotions to the same music [26, 31].

### Stereotyping as a psychological mechanism

Emotion stereotyping (and STEM) explain *how* emotion responses differ within and across cultures as a psychological process which may change over time, solely influenced by culture-dependent variables, (e.g., knowledge of the culture or fandom of a music genre from that culture). Thus, it is possible to view emotion stereotyping of music as three psychological mechanisms: evaluative conditioning, episodic memory, and aesthetic judgement of music [48–50].

Evaluative conditioning occurs when an emotion is induced by music as a result of previous pairing with other stimuli. Take one example, a calm response to Japanese *Gagaku* music because it has been previously paired with a meditative experience which made the listener feel calm. Subsequently, *Gagaku* music will trigger calmness, even in the absence of the mediation experience. Applying evaluative condition to our data, listeners were conditioned by a genre label which at times triggerred a stereotyped emotion [51–54].

Stereotyping can also occur as a result of the episodic memory mechanism. This mechanism involves an emotion response triggered by music because of a consciously accessible recollection of a past event. Unlike evaluative conditioning which can be triggered subconsciously, episodic memories are always conscious recollections of a previous event.

Juslin [48] extended the BRECVEM framework by including aesthetic judgement as a separate mechanism. In this framework, aesthetic judgements refer to a listener’s evaluation based on one or more of their individual aesthetic criteria reaching a certain level. For example, music judged as ‘beautiful’ evokes an aesthetic judgement. One of the factors on which aesthetic judgment depends is knowledge, which can change over time and context. Aesthetic orientations influence our cultural schemas and thus, our decisions and view of the world [66]. For example, much European writing on aesthetics refers to Western elite society, making assumptions about the experience that do not necessarily apply to all other cultures [67].

### Culture effect

We compared responses from two diverse cultures to identify similarities and differences in emotional responses to music lyrics. A number of differences were expected, considering these two cultures are evidently different [55, 56]. When primed with a genre label (OP and LP), similar emotion responses were observed between the two cultures for Western Opera, *Fado*, Heavy Metal, and *Gagaku*. This is consistent with cross-cultural research in music and emotion that provides empirical evidence for the recognition of certain emotions [3, 4, 13, 57–60]. The absence of sounded music in the current design suggests that emotion stereotyping is a cross-cultural phenomenon and that distinct cultures are susceptible to stereotyping. Differing emotion responses were also observed between cultures for Hip Hop, *Son*, Pop, and *Bossa Nova*. Taking Hip Hop as an example, Australians reported sadness, betrayal, and longing, while Cubans reported violence and sadness. Furthermore, differences between the two cultures were observed when participants responded to unprimed stimuli, such as for *Son* and *Bossa Nova*. Therefore, some lyrics are interpreted differently across cultures. This finding is likely not due to linguistic or expressive differences, as all stimuli and responses were professionally back-translated. Instead, the result can be explained by cultural differences between the two participant groups. To give meaning to the lyrics both Australians and Cubans undergo the same psychological processes but within different cultural frameworks [61–63]. The more cultural variation between the two cultures, the less convergence between emotion elicitation [64, 65]. However, when both cultures are primed with a genre label cue, and emotions converge more, this is taken as evidence that both cultures hold the same emotion stereotypes about the genre.

As both cultures and their music become more hybridized as an effect of western culture globalisation [35, 66], these data provide a unique insight in emotion responses from a culture which has been little influenced by westernisation, helping our understanding of cultural differences in music perception [67, 68].

### Fandom effect

An exploratory investigation was to measure the effects of fandom on emotion stereotyping, particularly that listeners with low and average fandom would likely respond using more stereotypical emotions than participants with high fandom. The higher number of reported emotions in genres with overall high fandom (Pop, 5 emotions) compared to low (*Gagaku*, 1 emotion), to average (Heavy Metal, 2 emotions) can be explained by STEM, because the theory predicts that highly familiar listeners are less likely to emotion stereotype music and thus attribute other, non-stereotyped emotions more flexibly. Although one can be familiar with a genre and not a fan, a fan is likely to be highly familiar with the music. High fandom listeners reported the highest number of above threshold emotion words, compared to low fandom and average fandom. Participants with different fandom levels have been observed to report different emotions to the same music [16, 26, 31, 69]. This explanation might also illustrate the anomaly observed in responses to *Son* (mentioned above) because Cuban listeners were likely to have high fandom for *Son*. Both joy and flavourful were reported in this study by high fandom listeners. In contrast, no high fandom listeners were self-identified for *Bolero* in our 2019 study, which can explain why joy and flavourful were not reported in the latter research. It also explains the contrasting emotion words reported by participants with high fandom as they are less likely to stereotype and hence use varied, sometimes even contrasting emotions, compared to low and average fans who frequently reported emotions from the same category for the same music genre. Psychoacoustic cues could aid the systematic responses for no fandom listeners [1]. While no firm conclusions can be drawn about the effect of fandom and stereotyping of emotion in music the results affirm the need of further research. Despite much work being conducted on the effect of familiarity and emotion in music [e.g., 70–72], fandom differs sublty from familiarity, and separating these two variables can help to tease apart the more specific predictions about fandom (not familiarity) made by STEM.

### Limitations and future directions

This study investigated 8 genres and 3 ways of labelling each of those genres. This design allowed participants to complete any of the three priming conditions using the one set of lyrics. Participants could not complete more than one of the priming levels with this design because they same lyrics would be presented with different contextualising priming information, drawing the participants’ attention to the hypothesis being tested. Some lyrics may have been more susceptible to the stereotypes exerted by music genre cues that we did not use for reasons of experimental efficiency, and so the data available from the design is necessarily limited [for further theoretical grounding on this issue, see 73]. A more ideal design might be to test participants in all conditions, and this could be achieved by having 32 sets of lyrics, covering the 8 genres by 3 priming conditions. The lyrics would then need to be repeated in many combinations so that they could appear at every level of genre-priming combination as a between-subject variable, but also probably revealing the purpose of the study, as people see the same lyrics used with different music genre labels as cues, or a between-subjects (preferably matched groups) design would be required. This would be a large-scale study that could be looked at in the future, or a smaller scale study could be conducted with fewer genres.

It is also possible that participants’ responses in the genre label priming test phase 3 could have been influenced by the sounded examples they heard in the genre awareness phase 1, or prior implicit or explicit experience with the genre, activating an internal representation of the genre different to the understanding the participant otherwise had (if they had one at all). That is, the participants may have a sound world of a genre in their minds that is triggered by the presentation of the music genre label, meaning that we cannot be certain that our method completely eliminated their sound world of a genre. However, the finding that the emotions triggered within a genre were consistent with a previous study [26] using a similar design but with different exemplars suggests that this problem is unlikely to have had a major impact on the present results. An alternative approach could be to ask participants to report emotion after being presented with a genre label alone without any exemplars of the genre presented. This, however, runs the risk of not knowing what the participant’s understanding is of the genre in question, which could pose problems particularly for genres that are not at least reasonably familiar to the participant.

Another limitation of the study is the use a convenience sample. Given the desire to recruit participants from diverse cultures, the design was more restricted for the Cuban participants who, for example, did not have easy access to computers, forcing data collection to take place using pen and paper. The opportunity to investigate diverse cultures justified this limitation.

Finally, this research examined the emotional content of musical lyrics, separate from any psychoacoustic influence. Overall, as shown in S1 Table, the sentiment analyses of both English and Spanish translations were very similar. However, lyrics for Western Classical have overall positively valanced lyrics, while both Portuguese *Fado* and Heavy Metal excerpts lean more towards negatively valanced sentiment. The results in Table 1 show similar trends, in that when participants were primed with lyrics from Western Opera a higher number of high valence/high arousal emotions were reported, and when primed with Portuguese *Fado* participants reported a higher number of low valence/low arousal emotions, and low valence/high arousal terms for Heavy Metal. Although a particular sentiment expressed by a lyric was identical regardless of the music genre label with which it was presented, it should be acknowledged that these inconsistencies in sentiment across the lyrics may have influenced the results, and should be more carefully controlled in the future.

## Conclusions

The overall aim of this study was to investigate the influence of extra-musical cues on emotional responses to music, and to investigate culture and fandom effects. Responses were gathered from two diverse cultures ensuring a range of responses to eight varied music genres, and diverse social cultures. The study demonstrated strong evidence of the prevalence of systematic emotional responses to music genres even in the absence of the music signal (the elimination of overt musical cues). Characteristic (expected) emotion responses to music genres were frequently reported.

Previous research has not been able to determine whether musical emotions can be triggered without an acoustic signal and to what extent extra-musical effects are responsible. The current study found evidence that a listener can experience musical emotions with a genre label cue, and without sounded music. This research primed music lyrics by labelling them as a particular genre which allowed the entire removal of the psychoacoustic cues, and overall, systematic changes were observed based on the genre label, that could not be attribtued to the sentiment of the lyrics. This led to the conclusion that emotion in music is partly dependent on previously held, culturally bound beliefs. In particular, Hip Hop, Heavy Metal, *Bossa Nova* and *Gagaku* music genres exhibited these systematic differences. Further to this, it appears that the contribution of culture and of fandom in emotion expectation has important implications considering the observed response differences between the cultures investigated, and because participants who are fans of a music genre emotion expect less than non-fans.

These differences were explained by the Stereotype Theory of Emotion in Music (STEM). STEM [18] provides a more refined explanation of how extra-musical and culture-specific cues impact on the communication of emotion in music because it not only proposes an effect of culture but explains the source of this effect as a preconceived belief.

The evidence in this study presents an important step forward in research on music and emotion, and points to novel and important developments in cross-cultural and music familiarity research. The study isolated the role of extra-musical cues and culture in emotional response to music by drawing listener attention to culturally based information using genre labels rather than using music signals. The significant role that extra-musical cues can play in responses to music was quantitatively demonstrated. Music research has only just begun to better understand the effects of extra-musical and culture-specific variables, and their impact on emotion communication needs to be considered for future research in this field.

## Supporting information

S1 Table Lyric transcripts and translations, genre origin, and genre of lure condition presented to participants in phase 3. (DOCX)

S2 Table Music excerpts (exemplars) used in phase 1 to elicit spontaneous emotion responses to a given genre. (DOCX)

S3 Table Terms and synonyms identified through the english oxford living dictionary and matched in ANEW 2017. (DOCX)

S4 Table Emotions and respective percentages reported above 5% of the total emotion words for each genre by culture for phase 3 responses. (DOCX)

S1 Database (HTM)
