# Supplementary material for: Musical emotions in the absence of music: A cross-cultural investigation of emotion communication in music by extra-musical cues
Source: PLoS One. 2020 Nov 18;15(11):e0241196. doi: 10.1371/journal.pone.0241196 (PMC7673536; doi:10.1371/journal.pone.0241196)
Supplement: S1 Table — (DOCX) [file pone.0241196.s001.docx]

**S1 Table. Lyric transcripts and translations, genre origin, and genre of lure condition presented to participants in Phase 3.**

| **Music piece** | **Order of Presentation** | **Original Music Genre** | **Lure Music Genre** | **English Transcript** | **iFeel Emotion Polarity Frequency (N)** | **Spanish Translation** | **iFeel Emotion Polarity Frequency (N)** |
| --- | --- | --- | --- | --- | --- | --- | --- |
| *La bohème*, Act II, Musetta’s Waltz by Giacomo Puccini | Australians: Randomised  Cubans: 1^st^ and 8^th^ | *Western Opera* | Pop | As I walk through the streets people turn  to look at me They inspect my beauty from head to toe I feel their eyes longing for my hidden charms | Positive (7)  Neutral (5)  Negative (4) | Cuando voy solita por la calle, la gente se para y mira y buscan mi belleza de la cabeza a los pies  Y saboreo entonces el anhelo sutil que sus ojos traspiran, y en esos encantos visibles intuyen mis bellezas ocultas | Positive (10)  Neutral (3)  Negative (3) |
| *Loucura (Sou do Fado)* by Carlos do Carmo | Australians: Randomised  Cubans: 2^nd^ and 7^th^ | *Fado* | *Western Opera* | Blessed is this madness To sing and suffer Cry, cry, poets from my country | Positive (2)  Neutral (4)  Negative (10) | Mas bendita esta locura de cantar y de sufrir  Llorad, llorad, poetas de mi país | Positive (2)  Neutral (5)  Negative (9) |
| *God id Dead?* by  Black Sabbath | Australians: Randomised  Cubans: 3^rd^ and 6^th^ | Heavy Metal | *Son* | Blood on my conscious And murder on mind Out of the gloom I rise up from my tomb into impending doom Now my body is my shrine | Positive (2)  Neutral (3)  Negative (11) | Sangre en mi conciencia Y asesinato en mi mente De las tinieblas surjo de mi tumba a un final inminente Ahora mi cuerpo es mi santuario | Positive (3)  Neutral (5)  Negative (8) |
| *The Isle of Arran* by Loyle Carner | Australians: Randomised  Cubans: 4^th^ and 5^th^ | Hip Hop | *Bossa Nova* | Know that I've been holding out, hoping to receive him I've been holding out for G but he was nowhere to be seen When I was bleeding | Positive (6)  Neutral (5)  Negative (5) | Debes saber que he estado esperando, esperando recibirlo He estado esperando a D pero no lo vi en ningún lado Cuando me desangraba | Positive (3)  Neutral (8)  Negative (5) |
| *Dos Gardenias* by Isolina Carrillo | Australians: Randomised  Cubans: 5^th^ and 4^th^ | *Son* | *Gagaku* | The Gardenias of my love will die It's because they have guessed that your love has betrayed me Because there is another will **^a^** | Positive (7)  Neutral (5)  Negative (4) | Las gardenias de mi amor se mueren Es que han adivinado que tu amor me ha traicionado porque existe otro querer | Positive (5)  Neutral (6)  Negative (4) |
| Lyrics by  Unknown | Australians: Randomised  Cubans: 6^th^ and 3rd | *Gagaku* | Heavy Metal | That all the various defiling things Sins and impurities to be cleansed and to be made pure Proclaim and request of heavenly spirits Of celestial realms **^a^** | Positive (7)  Neutral (4)  Negative (5) | Que todas las deshonras los pecados e impurezas por limpiar y purificar proclaman y piden a los espíritus divinos De los reinos celestiales | Positive (7)  Neutral (4)  Negative (6) |
| *How??* by  The Flaming Lips | Australians: Randomised  Cubans: 7^th^ and 2^nd^ | Pop | *Fado* | White trash rednecks, earthworms eat the ground Legalize it, every drug right now Are you with us or are you burnin’ out? | Positive (6)  Neutral (6)  Negative (4) | Catetos basura blanca, lombrices que comen tierra Legalícenlas, todas las drogas ya mismo ¿Estás con nosotros o te estás entregando? | Positive (5)  Neutral (8)  Negative (3) |
| *Água de Beber*, by Antonio Carlos Jobim | Australians: Randomised  Cubans: 8^th^ and 1^st^ | *Bossa Nova* | Hip Hop | I wanted to love but I was afraid and wanted to save my heart But love knows a secret fear that can kill your heart **^a^** | Positive (6)  Neutral (4)  Negative (6) | Quise amar pero tuve miedo, y quise salvar mi corazón Mas el amor sabe un secreto, el miedo puede matar tu corazón | Positive (6)  Neutral (5)  Negative (5) |
