# Supplementary material for: Musical emotions in the absence of music: A cross-cultural investigation of emotion communication in music by extra-musical cues
Source: PLoS One. 2020 Nov 18;15(11):e0241196. doi: 10.1371/journal.pone.0241196 (PMC7673536; doi:10.1371/journal.pone.0241196)
Supplement: S2 Table — (DOCX) [file pone.0241196.s002.docx]

**S2 Table. Music Excerpts (exemplars) used in Phase 1 to Elicit Spontaneous Emotion Responses to a given genre.**

*Music excerpts (exemplars) used in Phase 1 to elicit spontaneous emotion responses to a given genre*

| **Genre** | **Description** | **Stimuli** |
| --- | --- | --- |
| Western Classical | Art music written in the musical tradition of Western culture | 1. Holst, Gustav. *The Planets*. Op.31-1.Mars. [Recorded by Charles Dutoit & Orchestre Symphonique de Montréal], (2016). 2. Glass, Philip. *Morning Passages*. The Hours [OST], (2002). |
| *Fado* | A popular Portuguese music tradition often characterised by singing about working- class people and the sea | 1. Dos Reis Nunes, Marisa. *Oxalá*, (2001). 2. Camané. *Sei De Um Rio*, (2008). |
| Heavy Metal | A genre of rock music characteristically employing highly amplified distortion, loudness, and long guitar solos | 1. Korn. *Shoots and Ladders*, (1995). 2. Dream Theater. *The Gift of Music*, (2015). |
| Hip Hop | A genre of music characteristically employing rapping, djaying and beatboxing | 1. Coyle-Larner, Benjamin. *The Isle of Arran*, (2017). 2. Missy Elliot. *I’m Better*, (2017). |
| *Son* | A Cuban genre of music characteristically distinct by its use of a lyrical meter, five-stroke clave rhythm, *tres* guitar and call and response structure | 1. Rodríguez, Arsenio. *No Me Llores Más* [Recorded by Omara Portuondo], (2000). 2. Félix, Altuna. *Si Te Contera* [Recorded by Ibrahim Ferrer], (2005). |
| *Gagaku* | A Japanese Classical music tradition typically performed in imperial courts in Japan | 1. Katsutaro, Kouta. *Sangai-bushi/Sado Okesa,* (2017). 2. I Jiyushi & T Shikebu, *Joriri-Banraku,* (1964). |
| Pop | A genre of music characteristically employing short to medium length (two to four minutes) songs, repeated choruses and a simple structure, with an aim to appeal to a general audience | 1. Rogers, Maggie. *On/Off*, (2015). 2. William, Pharell. *Marilyn Monroe*, (2014). |
| *Bossa Nova* | A Brazilian genre of music typically employing a common signature with emphasis on the second beat, usually containing syncopation in the second measure | 1. Alcione. *Não Deixe O Samba Morrer*, (1975). 2. Sargento, Nelson. *Agoniza mas não morre*, (1979). |
