# Supplementary material for: Musical emotions in the absence of music: A cross-cultural investigation of emotion communication in music by extra-musical cues
Source: PLoS One. 2020 Nov 18;15(11):e0241196. doi: 10.1371/journal.pone.0241196 (PMC7673536; doi:10.1371/journal.pone.0241196)
Supplement: S3 Table — (DOCX) [file pone.0241196.s003.docx]

**S3 Table. Terms and Synonyms Identified Through The English Oxford Living Dictionary and Matched in ANEW 2017.**

| **The culture(s) which identified the emotion** | **Original term** | **Synonym found in ANEW 2017^a b^** |
| --- | --- | --- |
| Australian, Cuban | Nostalgia | Sentiment |
| Australian, Cuban | Sensual | Sexual |
| Australian, Cuban | Meditation | Reflection |
| Australian | Gory | Gruesome |
| Australian | Sombre | Dark |
| Australian | Longing | Wish |
| Australian | Anguish | Agony |
| Australian | Negative | Pessimistic |
| Australian | Soothing | Relax |
| Australian | Uplifted | Cheery |
| Australian | Chill | Relax |
| Australian | Pleasant | Enjoyment |
| Australian | Expressive | Emotional |
| Australian | Jovial | Jolly |
| Australian | Mellow | Sweet |
| Australian | Captivated | Attract |
| Australian | Distaste | Dislike |
| Australian | Liberation | Freedom |
| Australian | Angst | Anxiety |
| Australian | Feral | Savage |
| Australian | Pumped | Energy |
| Australian | Affronted | Outrage |
| Australian | Content | Peace |
| Australian | Laidback | Relaxed |
| Australian | Upbeat | Positive |
| Australian | Pissed Off | Annoyance |
| Australian | Animated | Lively |
| Australian | Agitated | Nervous |
| Australian | Enchanted | Charm |
| Australian | Harmonious | Peaceful |
| Australian | Vengeful | Resent |
| Australian | Sprightly | Lively |
| Cuban | Lament | Cry |
| Cuban | Yearning | Desire |
| Cuban | Loveless | Cold |
| Cuban | Divine | Holy |
| Cuban | Tranquillity | Peace |
| Cuban | Light-hearted | Cheerful |
| Cuban | Spirited | Lively |
| Cuban | Sultry | Sexy |
| Cuban | Euphoria | Exhilaration |
| Cuban | Out of Control | Wild |
| Cuban | Bewilderment | Confused |
| Cuban | Torment | Agony |
| Cuban | Unbearable | Annoyance |
| Cuban | Goosebumps | Excitement |
| Cuban | Liberation | Free |
| Cuban | Sublime | Awe |

*Note* ^a^ Synonym Found in ANEW 2017 through the English Oxford Living Dictionary, https://en.oxforddictionaries.com.

^b^ Some synonyms were used for more than one original emotion term, such as ‘lively’ used as a synonym for both ‘sprightly’ and ‘spirited’.
