# Supplementary material for: Musical emotions in the absence of music: A cross-cultural investigation of emotion communication in music by extra-musical cues
Source: PLoS One. 2020 Nov 18;15(11):e0241196. doi: 10.1371/journal.pone.0241196 (PMC7673536; doi:10.1371/journal.pone.0241196)
Supplement: S4 Table — (DOCX) [file pone.0241196.s004.docx]

**S4 Table. Emotions and Respective Percentages Reported above 5% of the Total Emotion Words for Each Genre by Culture for Phase 3 Responses.**

| **Culture** | | | | |  |  |  |
| --- | --- | --- | --- | --- | --- | --- | --- |
| Genre | **Primed** | | **Unprimed** | | **Lure** | |  |
|  | **Australian** | **Cuban** | **Australian** | **Cuban** | **Australian** | **Cuban** | |
| **Western Opera [Pop]** | TOT=46 pride (7%) | TOT=36 pride (14%) | TOT=52 lust (8%) | TOT=25 seduction (16%), vanity (8%), sensual (8%) | TOT=51 happy (6%) | TOT=38 love (37%), sensual (11%), pride (8%), vanity (5%) | |
| ***Fado* [Western Opera]** | TOT=50 sadness (17%) | TOT=30 patriotism (23%), sadness (23%), passion (10%) | TOT=55 sadness (36%) | TOT=31 sadness (16%), patriotic (10%), passion (10%), yearning (6%) | TOT=58 sadness (31%) | TOT=30 patriotic (30%), sadness (23%), suffering (23%) | |
| **Heavy Metal [*Son*]** | TOT=52 anger (14%) | TOT=29 madness (10%), violence (10%), tragedy (2%), fear (2%) | TOT=48 anger (15%), disgust (6%) | TOT=25 death (22%) | TOT=52 fear (6%) | TOT=40 sadness (45%), death (5%), passion (5%), peace (5%), violent (5%) | |
| **Hip Hop [*Bossa Nova]*** | TOT=53 sadness (18%), betrayal (8%), longing (6%) | TOT=28 violence (14%), sad (11%) | TOT=51 sadness (18%), betrayal (8%) | TOT=25 desperation (20%), madness (8%), pain (8%) | TOT=49 sadness (33%), sorrow (6%) | TOT=34 passion (29%), sadness (24%), desperation (12%), hope (6%) | |
| ***Son* [*Gagaku*]** | TOT=49 sadness (21%), betrayal (10%) | TOT=37 love (37%), attraction (16%), sadness (14%), betrayal (8%) | TOT=51 sadness (36%), betrayal (6%), love (6%) | TOT=28 love (32%), attraction (21%), sorrow (7%), nostalgia (7%) | TOT=56 sadness (45%) | TOT=31 love (42%), sadness (19%), attraction (10%), heartache (6%) | |
| ***Gagaku* [Heavy Metal]** | TOT=48 spiritual (29%) | TOT=28 spiritual (43%) | TOT=54 religion (6%), hope (6%) | TOT=24 religion (19%), shame (6%) | TOT=56 anger (6%), fear (6%), spiritual (6%) | TOT=32 torture (13%), madness (13%), evil (10%), spiritual (10%), fury (6%) | |
| **Pop [*Fado*]** | TOT=48 anger (14%), confused (14%), excited (6%) | TOT=30 freedom (7%), violence (7%) | TOT=53 anger (29%) | TOT=23 protest (13%) | TOT=56 anger (20%), rebellious (6%) | TOT=30 freedom (14%) | |
| ***Bossa Nova* [Hip Hop]** | TOT=50 sadness (22%), love (10%), fear (6%) | TOT=32 love (32%), fear (19%), insecure (10%), passion (6%) | TOT=47 sadness (28%) | TOT=25 fear (24%), love (20%), doubtful (8%) | TOT=54 sadness (19%), fear (7%) | TOT=33 love (9%), sadness (9%), regret (6%), fear (6%), loveless (6%) | |

*Note.* TOT = Total emotion words reported for each respective genre

[ ] = Genre presented in Lure condition
