# Supplementary material for: Musical emotions in the absence of music: A cross-cultural investigation of emotion communication in music by extra-musical cues
Source: PLoS One. 2020 Nov 18;15(11):e0241196. doi: 10.1371/journal.pone.0241196 (PMC7673536; doi:10.1371/journal.pone.0241196)
Supplement: S1 Database — (HTM) [file pone.0241196.s005.htm]

| STUDY LOCATION & DOCUMENTATION | RESPONDENT ID | NOTES: 1. BLACK ROWS = no data or jibberish. 2. BLANK CELLS = No data 3. In CONDITION section font colour means: 'White' = Primed with Original lyrics, 'Yellow' Unprimed, 'Black' primed incongruent'. 4. RED HIGHLIGHTED CELL = Error found AND CORRECTED in second spot check. 5. CN | SAMBA | FADO | SON/BOLEIRO | SHAMISEN/KOTO | HEAVY METAL | HIP HOP | POP | WESTERN CLASSICAL | FANDOM SAMBA | | FANDOM HEAVY METAL | | FANDOM POP |  | FANDOM HIP HOP | | FANDOM SHAMISEN/KOTO | | FANDOM WESTERN CLASSICAL | | FANDOM FADO | | FANDOM SON/BOLEIRO | | Participant Code | TYPE OF PRIMING FOR OPERA LYRICS | EMOTION RESPONSE "Below are the lyrics from an Classical Opera music excerpt. ""As I walk through the streets people turn  to look at me They inspect my beauty from head to toe I feel their eY longing for my hidden charms""  What is the first emotion that comes to your mind when you read these lyrics? Please provide just one word. There is no right or wrong answer.   [BLANK = NO ANSWER OR NO DATA]" | VLOOKUP | ValenceMeanFromANEW2017 | ArousalMeanFromANEW2017 | DominanceMeanFromANEW2017 | TYPE OF PRIMING FOR FADO LYRICS | EMOTION RESPONSE Below are the lyrics from a Portuguese Fado music excerpt. "Blessed is this madness To sing and suffer Cry, Cry, poets from my country" What is the first emotion that comes to your mind when you read these lyrics? Please provide just one word. There is no right or wrong answer. | VLOOKUP | ValenceMeanFromANEW2017 | ArousalMeanFromANEW2017 | DominanceMeanFromANEW2017 | TYPE OF PRIMING FOR HEAVY METAL LYRICS | EMOTION RESPONSE Below are the lyrics from a Heavy Metal excerpt. "Blood on my conscious And murder in mind Out of the gloom I rise up from my tomb into impending doom Now my body is my shrine"  What is the first emotion that comes to your mind when you read these lyrics? Please provide just one word. There is no right or wrong answer. | VLOOKUP | ValenceMeanFromANEW2017 | ArousalMeanFromANEW2017 | DominanceMeanFromANEW2017 | TYPE OF PRIMING FOR HIP HOP LYRICS | EMOTION RESPONSE Below are the lyrics from a Hip Hop excerpt. "Know that I've been holding out, hoping to receive him I've been holding out for G but he was nowhere to be seen When I was bleeding"  What is the first emotion that comes to your mind when you read these lyrics? Please provide just one word. There is no right or wrong answer. | VLOOKUP | ValenceMeanFromANEW2017 | ArousalMeanFromANEW2017 | DominanceMeanFromANEW2017 | TYPE OF PRIMING FOR BOLERO LYRICS | EMOTION RESPONSE Below are the lyrics from a Cuban Bolero music excerpt. "The Gardenias of my love will die It's because they have guessed that your love has betrayed me Because there is another will" What is the first emotion that comes to your mind when you read these lyrics? Please provide just one word. There is no right or wrong answer. | VLOOKUP | ValenceMeanFromANEW2017 | ArousalMeanFromANEW2017 | DominanceMeanFromANEW2017 | TYPE OF PRIMING FOR KOTO LYRICS | EMOTION RESPONSE Below are the lyrics from a Japanese traditional music excerpt. "That all the various defiling things Sins and impurities to be cleansed and to be madnesse pure Proclaim and request of heavinly spirits Of celestial realms" What is the first emotion that comes to your mind when you read these lyrics? Please provide just one word. There is no right or wrong answer. | VLOOKUP | ValenceMeanFromANEW2017 | ArousalMeanFromANEW2017 | DominanceMeanFromANEW2017 | TYPE OF PRIMING FOR POP LYRICS | EMOTION RESPONSE Below are the lyrics from a Pop music excerpt. "White trash rednecks, earthworms eat the ground Legalize it, every drug right now Are you with us or are you burnin’ out?" What is the first emotion that comes to your mind when you read these lyrics? Please provide just one word. There is no right or wrong answer. | VLOOKUP | ValenceMeanFromANEW2017 | ArousalMeanFromANEW2017 | DominanceMeanFromANEW2017 | TYPE OF PRIMING FOR SAMBA LYRICS | EMOTION RESPONSE Below are the lyrics from a Brazilian Samba music excerpt. "I wanted to love but I was afraid and wanted to save my heart But love knows a secret fear that can kill your heart" What is the first emotion that comes to your mind when you read these lyrics? Please provide just one word. There is no right or wrong answer. | VLOOKUP | ValenceMeanFromANEW2017 | ArousalMeanFromANEW2017 | DominanceMeanFromANEW2017 | | |
|  |  |  | Please listen to these short excerpts EXACTLY ONCE EACH.  The excerpts are examples of Brazilian Samba music.     <source src="//www.surveys.unsw.edu.au/User/66/66499/media/31/3179.mp3?autoplay=1" type="audio/ogg">   <source src="//www.surveys.unsw.edu.au/User/66/66499/media/31/3179.mp3?autoplay=1" type="audio/mpeg">   Your browser does not support the audio tag.       <source src="//www.surveys.unsw.edu.au/User/66/66499/media/31/3180.mp3?autoplay=1" type="audio/ogg">   <source src="//www.surveys.unsw.edu.au/User/66/66499/media/31/3180.mp3?autoplay=1"audio/mpeg">   Your browser does not support the audio tag.   What is the first emotion that comes to your mind when you hear this music genre (not necessarily these two pieces)? Please provide just one word. There is no right or wrong answer.  </div></div> | Please listen to these short excerpts EXACTLY ONCE EACH.  The excerpts are examples of Portuguese Fado music.     <source src="//www.surveys.unsw.edu.au/User/66/66499/media/31/3181.mp3?autoplay=1" type="audio/ogg">   <source src="//www.surveys.unsw.edu.au/User/66/66499/media/31/3181.mp3?autoplay=1" type="audio/mpeg">   Your browser does not support the audio tag.       <source src="//www.surveys.unsw.edu.au/User/66/66499/media/31/3182.mp3?autoplay=1" type="audio/ogg">   <source src="//www.surveys.unsw.edu.au/User/66/66499/media/31/3182.mp3?autoplay=1"audio/mpeg">   Your browser does not support the audio tag.   What is the first emotion that comes to your mind when you hear this music genre (not necessarily these two pieces)? Please provide just one word. There is no right or wrong answer.  </div></div> | Please listen to these short excerpts EXACTLY ONCE EACH.  The excerpts are examples of Cuban Son music.     <source src="//www.surveys.unsw.edu.au/User/66/66499/media/31/3183.mp3?autoplay=1" type="audio/ogg">   <source src="//www.surveys.unsw.edu.au/User/66/66499/media/31/3183.mp3?autoplay=1" type="audio/mpeg">   Your browser does not support the audio tag.       <source src="//www.surveys.unsw.edu.au/User/66/66499/media/31/3184.mp3?autoplay=1" type="audio/ogg">   <source src="//www.surveys.unsw.edu.au/User/66/66499/media/31/3184.mp3?autoplay=1"audio/mpeg">   Your browser does not support the audio tag.   What is the first emotion that comes to your mind when you hear this music genre (not necessarily these two pieces)? Please provide just one word. There is no right or wrong answer.  </div></div> | Please listen to these short excerpts EXACTLY ONCE EACH.  The excerpts are examples of Japanese Gagaku music.     <source src="//www.surveys.unsw.edu.au/User/66/66499/media/31/3185.mp3?autoplay=1" type="audio/ogg">   <source src="//www.surveys.unsw.edu.au/User/66/66499/media/31/3185.mp3?autoplay=1" type="audio/mpeg">   Your browser does not support the audio tag.       <source src="//www.surveys.unsw.edu.au/User/66/66499/media/31/3186.mp3?autoplay=1" type="audio/ogg">   <source src="//www.surveys.unsw.edu.au/User/66/66499/media/31/3186.mp3?autoplay=1"audio/mpeg">   Your browser does not support the audio tag.   What is the first emotion that comes to your mind when you hear this music genre (not necessarily these two pieces)? Please provide just one word. There is no right or wrong answer.  </div></div> | Please listen to these short excerpts EXACTLY ONCE EACH.  The excerpts are examples of Heavy Metal music.     <source src="//www.surveys.unsw.edu.au/User/66/66499/media/31/3187.mp3?autoplay=1" type="audio/ogg">   <source src="//www.surveys.unsw.edu.au/User/66/66499/media/31/3187.mp3?autoplay=1" type="audio/mpeg">   Your browser does not support the audio tag.       <source src="//www.surveys.unsw.edu.au/User/66/66499/media/31/3188.mp3?autoplay=1" type="audio/ogg">   <source src="//www.surveys.unsw.edu.au/User/66/66499/media/31/3188.mp3?autoplay=1"audio/mpeg">   Your browser does not support the audio tag.   What is the first emotion that comes to your mind when you hear this music genre (not necessarily these two pieces)? Please provide just one word. There is no right or wrong answer.  </div></div> | Please listen to these short excerpts EXACTLY ONCE EACH.  The excerpts are examples of Hip Hop music.      <source src="//www.surveys.unsw.edu.au/User/66/66499/media/31/3189.mp3?autoplay=1" type="audio/ogg">   <source src="//www.surveys.unsw.edu.au/User/66/66499/media/31/3189.mp3?autoplay=1" type="audio/mpeg">   Your browser does not support the audio tag.       <source src="//www.surveys.unsw.edu.au/User/66/66499/media/31/3190.mp3?autoplay=1" type="audio/ogg">   <source src="//www.surveys.unsw.edu.au/User/66/66499/media/31/3190.mp3?autoplay=1"audio/mpeg">   Your browser does not support the audio tag.   What is the first emotion that comes to your mind when you hear this music genre (not necessarily these two pieces)? Please provide just one word. There is no right or wrong answer.  </div></div> | Please listen to these short excerpts EXACTLY ONCE EACH.  The excerpts are examples of Pop music.     <source src="//www.surveys.unsw.edu.au/User/66/66499/media/31/3191.mp3?autoplay=1" type="audio/ogg">   <source src="//www.surveys.unsw.edu.au/User/66/66499/media/31/3191.mp3?autoplay=1" type="audio/mpeg">   Your browser does not support the audio tag.       <source src="//www.surveys.unsw.edu.au/User/66/66499/media/31/3192.mp3?autoplay=1" type="audio/ogg">   <source src="//www.surveys.unsw.edu.au/User/66/66499/media/31/3192.mp3?autoplay=1"audio/mpeg">   Your browser does not support the audio tag.   What is the first emotion that comes to your mind when you hear this music genre (not necessarily these two pieces)? Please provide just one word. There is no right or wrong answer.  </div></div> | Please listen to these short excerpts EXACTLY ONCE EACH.  The excerpts are examples of Western Classical music.     <source src="//www.surveys.unsw.edu.au/User/66/66499/media/31/3193.mp3?autoplay=1" type="audio/ogg">   <source src="//www.surveys.unsw.edu.au/User/66/66499/media/31/3193.mp3?autoplay=1" type="audio/mpeg">   Your browser does not support the audio tag.       <source src="//www.surveys.unsw.edu.au/User/66/66499/media/31/3194.mp3?autoplay=1" type="audio/ogg">   <source src="//www.surveys.unsw.edu.au/User/66/66499/media/31/3194.mp3?autoplay=1"audio/mpeg">   Your browser does not support the audio tag.   What is the first emotion that comes to your mind when you hear this music genre (not necessarily these two pieces)? Please provide just one word. There is no right or wrong answer.  </div></div> | Please indicate the extent to which you agree that you are a fan (that is, a keen follower) of the music listed on each row below: | | | | |  |  |  |  |  |  |  |  |  |  |  | 0 |  |  |  |  |  |  |  |  |  |  |  |  |  |  |  |  |  |  |  |  |  |  |  |  |  |  |  |  |  |  |  |  |  |  |  |  |  |  |  |  |  |  |  |  |  |  |  |  |  |  |
| SYDNEY | 1 |  |  |  |  |  |  |  |  |  |  |  |  |  |  |  |  |  |  |  |  |  |  |  |  |  | 1 |  |  |  |  |  |  |  |  |  |  |  |  |  |  |  |  |  |  |  |  |  |  |  |  |  |  |  |  |  |  |  |  |  |  |  |  |  |  |  |  |  |  |  |  |  |  |  |  |  |  |
| SYDNEY | 12345 |  | q | q | q | q | q | q | q | q |  |  |  |  |  |  |  |  |  |  |  |  |  |  |  |  | 12345 |  |  |  |  |  |  |  |  |  |  |  |  |  |  |  |  |  |  |  |  |  |  |  |  |  |  |  |  |  |  |  |  |  |  |  |  |  |  |  |  |  |  |  |  |  |  |  |  |  |  |
| SYDNEY | 123456 |  | excited | happy | pumped | chill | angry | angry | excited | chill | Neither Agree nor Disagree | 4 | Neither Agree nor Disagree | 4 | Neither Agree nor Disagree | 4 | Neither Agree nor Disagree | 4 | Neither Agree nor Disagree | 4 | Neither Agree nor Disagree | 4 | Neither Agree nor Disagree | 4 | Neither Agree nor Disagree | 4 | 123456 |  |  |  |  |  |  |  |  |  |  |  |  |  |  |  |  |  |  |  |  |  |  |  |  |  |  |  |  |  |  |  |  |  |  |  |  |  |  |  |  |  |  |  |  |  |  |  |  |  |  |
| SYDNEY | 503344 |  | wdqwd | rarer | wcwc | lknlkn | onion | erferf | iohoih | efeqrf |  |  |  |  |  |  |  |  |  |  |  |  |  |  |  |  | 503344 |  |  |  |  |  |  |  |  |  |  |  |  |  |  |  |  |  |  |  |  |  |  |  |  |  |  |  |  |  |  |  |  |  |  |  |  |  |  |  |  |  |  |  |  |  |  |  |  |  |  |
| SYDNEY | z1234567 |  |  |  | a | a |  | b | n | n |  |  |  |  |  |  |  |  |  |  |  |  |  |  |  |  | 1234567 |  |  |  |  |  |  |  |  |  |  |  |  |  |  |  |  |  |  |  |  |  |  |  |  |  |  |  |  |  |  |  |  |  |  |  |  |  |  |  |  |  |  |  |  |  |  |  |  |  |  |
| SYDNEY | 3252269 |  | Relaxed, taking things easy; happiness | Calm, reflection | Relaxed, reflection | Anticipation/mystery | Extreme anger | Carefree, rebellious | Happiness/energy | Deep reflection, serious | Neither Agree nor Disagree | 4 | Strongly Disagree | 1 | Moderately Agree | 6 | Moderately Disagree | 2 | Moderately Disagree | 2 | Strongly Agree | 7 | Slightly Disagree | 3 | Slightly Disagree | 3 | 3252269 | PRIMED OPERA (ORIGINAL LYRICS OPERA) | Reflection | Reflection | 5.97 | 4.38 | 6.1 | PRIMED FADO (ORIGINAL LYRICS FADO) | Passionate | passion | 8.03 | 7.26 | 6.13 | PRIMED HEAVY METAL (ORIGINAL LYRICS HEAVY METAL) | Hatred | Hatred | 1.98 | 6.66 | 4.3 | PRIMED HIP HOP (ORIGINAL LYRICS HIP HOP) | Helplessness | Helplessness | #N/A | #N/A | #N/A | PRIMED BOLERO (ORIGINAL LYRICS BOLERO) | Sadness | sadness | 2.21 | 5.21 | 2.82 | PRIMED KOTO (ORIGINAL LYRICS KOTO) | Reflection | Reflection | 5.97 | 4.38 | 6.1 | PRIMED POP (ORIGINAL LYRICS POP) | Anger | anger | 2.34 | 7.63 | 5.5 | PRIMED SAMBA (ORIGINAL LYRICS SAMBA) | Fear | fear | 2.76 | 6.96 | 3.22 |  |  |
| SYDNEY | 3254314 |  | Calm | captivated | relaxed | Intrigue | Anger | Animated | amusement | Inspiration | Moderately Disagree | 2 | Moderately Agree | 6 | Moderately Agree | 6 | Strongly Agree | 7 | Moderately Disagree | 2 | Strongly Agree | 7 | Moderately Disagree | 2 | Moderately Disagree | 2 | 3254314 | UNPRIMED (ORIGINAL LYRICS OPERA) | anxiety | anxiety | 2.77 | 6.72 | 2.72 | UNPRIMED (ORIGINAL LYRICS FADO) | sorrow | sorrow | 2.32 | 4.48 | 3.67 | UNPRIMED (ORIGINAL LYRICS HEAVY METAL) | fear | fear | 2.76 | 6.96 | 3.22 | UNPRIMED (ORIGINAL LYRICS HIP HOP) | longing | Longing | #N/A | #N/A | #N/A | UNPRIMED (ORIGINAL LYRICS BOLERO) | sorrow | sorrow | 2.32 | 4.48 | 3.67 | UNPRIMED (ORIGINAL LYRICS KOTO) | fascination | Fascination | #N/A | #N/A | #N/A | UNPRIMED (ORIGINAL LYRICS POP) | animated | animated | #N/A | #N/A | #N/A | UNPRIMED (ORIGINAL LYRICS SAMBA) | inspiration | inspiration | 7.48 | 6.22 | 5.87 |  |  |
| SYDNEY | 3254314 |  |  |  |  |  |  |  |  |  |  |  |  |  |  |  |  |  |  |  |  |  |  |  |  |  | 3254314 |  |  | #N/A | #N/A | #N/A | #N/A |  |  | #N/A | #N/A | #N/A | #N/A |  |  | #N/A | #N/A | #N/A | #N/A |  |  | #N/A | #N/A | #N/A | #N/A |  |  | #N/A | #N/A | #N/A | #N/A |  |  | #N/A | #N/A | #N/A | #N/A |  |  | #N/A | #N/A | #N/A | #N/A |  |  | #N/A | #N/A | #N/A | #N/A |  |  |
| SYDNEY | 3275760 |  | Excited | Glad | Longing | Sad | angry | Angry | Happy | classic | Strongly Agree | 7 | I don't know this music | 8 | Strongly Agree | 7 | Strongly Agree | 7 | I don't know this music | 8 | Strongly Agree | 7 | I don't know this music | 8 | I don't know this music | 8 | 3275760 |  |  | #N/A | #N/A | #N/A | #N/A |  |  | #N/A | #N/A | #N/A | #N/A |  |  | #N/A | #N/A | #N/A | #N/A |  |  | #N/A | #N/A | #N/A | #N/A |  |  | #N/A | #N/A | #N/A | #N/A |  |  | #N/A | #N/A | #N/A | #N/A |  |  | #N/A | #N/A | #N/A | #N/A |  |  | #N/A | #N/A | #N/A | #N/A |  |  |
| SYDNEY | 3332513 |  | joyful | inspired | excited | Indifferent | Rage | provocative | playful | ecstatic | Slightly Agree | 5 | Moderately Disagree | 2 | Moderately Agree | 6 | Strongly Agree | 7 | Moderately Disagree | 2 | Moderately Agree | 6 | Moderately Agree | 6 | Moderately Agree | 6 | 3332513 | UNPRIMED (ORIGINAL LYRICS OPERA) | confident | confident | 7.98 | 6.22 | 7.68 | UNPRIMED (ORIGINAL LYRICS FADO) | Poignant | Poignant | #N/A | #N/A | #N/A | UNPRIMED (ORIGINAL LYRICS HEAVY METAL) | isolated | isolated | #N/A | #N/A | #N/A | UNPRIMED (ORIGINAL LYRICS HIP HOP) | Sadness | sadness | 2.21 | 5.21 | 2.82 | UNPRIMED (ORIGINAL LYRICS BOLERO) | lonely | lonely | 2.17 | 4.51 | 2.95 | UNPRIMED (ORIGINAL LYRICS BOLERO) | peaceful | peaceful | 7.77 | 2.87 | 5.7 | UNPRIMED (ORIGINAL LYRICS POP) | aggressive | aggressive | 5.1 | 5.83 | 5.59 | UNPRIMED (ORIGINAL LYRICS SAMBA) | hurt | hurt | 1.9 | 5.85 | 3.33 |  |  |
| SYDNEY | 3373251 |  | calm | sentimental | excitement | confused | irritated | no emotions | no emotions | touched | I don't know this music | 8 | Strongly Disagree | 1 | Strongly Agree | 7 | Slightly Agree | 5 | Neither Agree nor Disagree | 4 | Strongly Agree | 7 | Neither Agree nor Disagree | 4 | Neither Agree nor Disagree | 4 | 3373251 |  |  | #N/A | #N/A | #N/A | #N/A |  |  | #N/A | #N/A | #N/A | #N/A |  |  | #N/A | #N/A | #N/A | #N/A |  |  | #N/A | #N/A | #N/A | #N/A |  |  | #N/A | #N/A | #N/A | #N/A |  |  | #N/A | #N/A | #N/A | #N/A |  |  | #N/A | #N/A | #N/A | #N/A |  |  | #N/A | #N/A | #N/A | #N/A |  |  |
| SYDNEY | z3374318 |  | Romantic | Romantic | Tense | Tense | Angsty | Cringe | Uninspired | Epiphany | Slightly Agree | 5 | Moderately Disagree | 2 | Strongly Agree | 7 | Strongly Agree | 7 | Slightly Agree | 5 | Neither Agree nor Disagree | 4 | Slightly Agree | 5 | Slightly Agree | 5 | 3374318 | PRIMED OPERA (ORIGINAL LYRICS OPERA) | Cringe | Cringe | #N/A | #N/A | #N/A | PRIMED FADO (ORIGINAL LYRICS FADO) | Melodramatic | Melodramatic | #N/A | #N/A | #N/A | PRIMED HEAVY METAL (ORIGINAL LYRICS HEAVY METAL) | Embarassed | embarrassed | 3.03 | 5.87 | 2.87 | PRIMED HIP HOP (ORIGINAL LYRICS HIP HOP) | Unaffected | Unaffected | #N/A | #N/A | #N/A | PRIMED BOLERO (ORIGINAL LYRICS BOLERO) | Sympathetic | sympathetic | 5.74 | 3.99 | 5.59 | PRIMED KOTO (ORIGINAL LYRICS KOTO) | Sad | sad | 1.61 | 4.13 | 3.45 | PRIMED POP (ORIGINAL LYRICS POP) | Unimpressed | Unimpressed | #N/A | #N/A | #N/A | PRIMED SAMBA (ORIGINAL LYRICS SAMBA) | Unsympathetic | Unsympathetic | #N/A | #N/A | #N/A |  |  |
| SYDNEY | 3376421 |  | Excitement | Boredom | Nostalgic | intrigued | Annoyed | Defiance | pleased | Triumphant | Moderately Disagree | 2 | Strongly Disagree | 1 | Moderately Agree | 6 | Slightly Agree | 5 | I don't know this music | 8 | Slightly Disagree | 3 | I don't know this music | 8 | Slightly Disagree | 3 | 3376421 | PRIMED OPERA (ORIGINAL LYRICS OPERA) | scepticism | scepticism | #N/A | #N/A | #N/A | PRIMED FADO (ORIGINAL LYRICS FADO) | anticipation | anticipation | #N/A | #N/A | #N/A | PRIMED HEAVY METAL (ORIGINAL LYRICS HEAVY METAL) | amusement | amusement | 7.96 | 6.22 | 5.74 | PRIMED HIP HOP (ORIGINAL LYRICS HIP HOP) | sadness | sadness | 2.21 | 5.21 | 2.82 | PRIMED BOLERO (ORIGINAL LYRICS BOLERO) | sadness | sadness | 2.21 | 5.21 | 2.82 | PRIMED KOTO (ORIGINAL LYRICS KOTO) | confused | confused | 3.21 | 6.03 | 4.24 | PRIMED POP (ORIGINAL LYRICS POP) | resignation | resign | 3.47 | 4.21 | 4.57 | PRIMED SAMBA (ORIGINAL LYRICS SAMBA) | amusement | amusement | 7.96 | 6.22 | 5.74 |  |  |
| SYDNEY | z3413806 |  | Old | sweet | funky | deceit | angry | cool | modern | mystery | Neither Agree nor Disagree | 4 | Strongly Disagree | 1 | Strongly Agree | 7 | Strongly Agree | 7 | Moderately Disagree | 2 | Strongly Agree | 7 | Slightly Disagree | 3 | Strongly Disagree | 1 | 3413806 | UNPRIMED (ORIGINAL LYRICS OPERA) | young | young | 6.89 | 5.64 | 5.3 | UNPRIMED (ORIGINAL LYRICS FADO) | sad | sad | 1.61 | 4.13 | 3.45 | UNPRIMED (ORIGINAL LYRICS HEAVY METAL) | danger | danger | 2.95 | 7.32 | 3.59 | UNPRIMED (ORIGINAL LYRICS HIP HOP) | religion | 0 | #N/A | #N/A | #N/A | UNPRIMED (ORIGINAL LYRICS BOLERO) | sad | sad | 1.61 | 4.13 | 3.45 | UNPRIMED (ORIGINAL LYRICS BOLERO) | religion | 0 | #N/A | #N/A | #N/A | UNPRIMED (ORIGINAL LYRICS POP) | dangerous | dangerous | 3.69 | 6.29 | 4.93 | UNPRIMED (ORIGINAL LYRICS SAMBA) | Introverted | Introverted | #N/A | #N/A | #N/A |  |  |
| SYDNEY | z3416693 |  | light hearted | dreamy | relaxed | nostalgic | confused | energetic | happy | calm | Slightly Agree | 5 | Strongly Disagree | 1 | Strongly Agree | 7 | Strongly Agree | 7 | Slightly Agree | 5 | Neither Agree nor Disagree | 4 | Moderately Agree | 6 | Moderately Agree | 6 | 3416693 | PRIMED OPERA (ORIGINAL LYRICS OPERA) | insecure | insecure | 2.36 | 5.56 | 2.33 | PRIMED FADO (ORIGINAL LYRICS FADO) | sadness | sadness | 2.21 | 5.21 | 2.82 | PRIMED HEAVY METAL (ORIGINAL LYRICS HEAVY METAL) | anger | anger | 2.34 | 7.63 | 5.5 | PRIMED HIP HOP (ORIGINAL LYRICS HIP HOP) | anger | anger | 2.34 | 7.63 | 5.5 | PRIMED BOLERO (ORIGINAL LYRICS BOLERO) | sadness | sadness | 2.21 | 5.21 | 2.82 | PRIMED KOTO (ORIGINAL LYRICS KOTO) | dark | Dark | 4.71 | 4.28 | 4.84 | PRIMED POP (ORIGINAL LYRICS POP) | anger | anger | 2.34 | 7.63 | 5.5 | PRIMED SAMBA (ORIGINAL LYRICS SAMBA) | mystery | mystery | #N/A | #N/A | #N/A |  |  |
| SYDNEY | z3417645 |  | relaxed | love | love | excited | angry | chill | happy | tragic | Neither Agree nor Disagree | 4 | Strongly Disagree | 1 | Strongly Agree | 7 | Strongly Agree | 7 | Neither Agree nor Disagree | 4 | Slightly Agree | 5 | Moderately Agree | 6 | Neither Agree nor Disagree | 4 | 3417645 | PRIMED POP (ORIGINAL LYRICS OPERA) | confident | confident | 7.98 | 6.22 | 7.68 | PRIMED OPERA (ORIGINAL LYRICS FADO) | sad | sad | 1.61 | 4.13 | 3.45 | PRIMED CUBAN SON (ORIGINAL LYRICS HEAVY METAL) | scared | scared | 2.78 | 6.82 | 2.94 | PRIMED SAMBA (ORIGINAL LYRICS HIP HOP) | upset | upset | 2 | 5.86 | 4.08 | PRIMED KOTO (ORIGINAL LYRICS BOLERO) | beauty and sadness | sad | 1.61 | 4.13 | 3.45 | PRIMED HEAVY METAL (ORIGINAL LYRICS KOTO) | spiritual | spirit | 7 | 5.56 | 5.82 | PRIMED FADO (ORIGINAL LYRICS POP) | anger | anger | 2.34 | 7.63 | 5.5 | PRIMED HIP HOP (ORIGINAL LYRICS SAMBA) | sympathetic | sympathetic | 5.74 | 3.99 | 5.59 |  |  |
| SYDNEY | 3420952 |  | hungry | sad | energetic | confused | tired | soulful | happy | musical | Moderately Disagree | 2 | Moderately Disagree | 2 | Slightly Agree | 5 | Slightly Disagree | 3 | Moderately Disagree | 2 | Slightly Agree | 5 | Moderately Disagree | 2 | Moderately Disagree | 2 | 3420952 | PRIMED POP (ORIGINAL LYRICS OPERA) | annoyed | annoy | 2.96 | 5.52 | 4.44 | PRIMED OPERA (ORIGINAL LYRICS FADO) | confused | confused | 3.21 | 6.03 | 4.24 | PRIMED CUBAN SON (ORIGINAL LYRICS HEAVY METAL) | strong | strong | 7.11 | 5.92 | 6.92 | PRIMED SAMBA (ORIGINAL LYRICS HIP HOP) | sympathetic | sympathetic | 5.74 | 3.99 | 5.59 | PRIMED KOTO (ORIGINAL LYRICS BOLERO) | sad | sad | 1.61 | 4.13 | 3.45 | PRIMED HEAVY METAL (ORIGINAL LYRICS KOTO) | confused | confused | 3.21 | 6.03 | 4.24 | PRIMED FADO (ORIGINAL LYRICS POP) | curious | Curious | 6.08 | 5.82 | 5.42 | PRIMED HIP HOP (ORIGINAL LYRICS SAMBA) | fear | fear | 2.76 | 6.96 | 3.22 |  |  |
| SYDNEY | 3441031 |  | calm | peace | Serenity | alert | anger | excitement | irritation | awestruck | Neither Agree nor Disagree | 4 | Strongly Disagree | 1 | Slightly Disagree | 3 | Moderately Disagree | 2 | Neither Agree nor Disagree | 4 | Moderately Agree | 6 | Neither Agree nor Disagree | 4 | Neither Agree nor Disagree | 4 | 3441031 | PRIMED POP (ORIGINAL LYRICS OPERA) | deceit | deceit | 2.9 | 5.68 | 3.95 | PRIMED OPERA (ORIGINAL LYRICS FADO) | hopelessness | hopeless | 2.27 | 4.28 | 2.96 | PRIMED CUBAN SON (ORIGINAL LYRICS HEAVY METAL) | evil | evil | 3.23 | 6.39 | 5.25 | PRIMED SAMBA (ORIGINAL LYRICS HIP HOP) | sadness | sadness | 2.21 | 5.21 | 2.82 | PRIMED KOTO (ORIGINAL LYRICS BOLERO) | sadness | sadness | 2.21 | 5.21 | 2.82 | PRIMED HEAVY METAL (ORIGINAL LYRICS KOTO) | fear | fear | 2.76 | 6.96 | 3.22 | PRIMED FADO (ORIGINAL LYRICS POP) | anger | anger | 2.34 | 7.63 | 5.5 | PRIMED HIP HOP (ORIGINAL LYRICS SAMBA) | Annoyed | annoy | 2.96 | 5.52 | 4.44 |  |  |
| SYDNEY | z3441031 |  | serenity | calm | calm | alert | anger | upbeat | irritated | peaceful | Neither Agree nor Disagree | 4 | Strongly Disagree | 1 | Slightly Disagree | 3 | Moderately Disagree | 2 | Neither Agree nor Disagree | 4 | Moderately Agree | 6 | Neither Agree nor Disagree | 4 | Neither Agree nor Disagree | 4 | 3441031 | UNPRIMED (ORIGINAL LYRICS OPERA) | Pleased | Pleased | 7.79 | 5.68 | 6.29 | UNPRIMED (ORIGINAL LYRICS FADO) | pleased | Pleased | 7.79 | 5.68 | 6.29 | UNPRIMED (ORIGINAL LYRICS HEAVY METAL) | Dark | Dark | 4.71 | 4.28 | 4.84 | UNPRIMED (ORIGINAL LYRICS HEAVY METAL) | unpleased | unpleased | #N/A | #N/A | #N/A | UNPRIMED (ORIGINAL LYRICS BOLERO) | | #N/A | #N/A | #N/A | #N/A | UNPRIMED (ORIGINAL LYRICS BOLERO) | weird | weird | 5.17 | 4.82 | 5.13 | UNPRIMED (ORIGINAL LYRICS POP) | Stupid | stupid | 2.31 | 4.72 | 2.98 | UNPRIMED (ORIGINAL LYRICS SAMBA) | | #N/A | #N/A | #N/A | #N/A |  |  |
| SYDNEY | 3458971 |  | expectation | nostalgic | happy | scared | angry | pumped | excitement | calm | Slightly Agree | 5 | Strongly Disagree | 1 | Strongly Agree | 7 | Strongly Agree | 7 | Slightly Disagree | 3 | Slightly Agree | 5 | Slightly Disagree | 3 | Slightly Agree | 5 | 3458971 |  |  | #N/A | #N/A | #N/A | #N/A |  |  | #N/A | #N/A | #N/A | #N/A |  |  | #N/A | #N/A | #N/A | #N/A |  |  | #N/A | #N/A | #N/A | #N/A |  |  | #N/A | #N/A | #N/A | #N/A |  |  | #N/A | #N/A | #N/A | #N/A |  |  | #N/A | #N/A | #N/A | #N/A |  |  | #N/A | #N/A | #N/A | #N/A |  |  |
| SYDNEY | 3458971 |  | good | good | good | good | good | good | good | good |  |  |  |  |  |  |  |  |  |  |  |  |  |  |  |  | 3458971 | PRIMED POP (ORIGINAL LYRICS OPERA) | insecurity | insecure | 2.36 | 5.56 | 2.33 | PRIMED OPERA (ORIGINAL LYRICS FADO) | madness | madness | 4.37 | 5.8 | 4.53 | PRIMED CUBAN SON (ORIGINAL LYRICS HEAVY METAL) | fear | fear | 2.76 | 6.96 | 3.22 | PRIMED SAMBA (ORIGINAL LYRICS HIP HOP) | Sad | sad | 1.61 | 4.13 | 3.45 | PRIMED KOTO (ORIGINAL LYRICS BOLERO) | sadness | sadness | 2.21 | 5.21 | 2.82 | PRIMED HEAVY METAL (ORIGINAL LYRICS KOTO) | anger | anger | 2.34 | 7.63 | 5.5 | PRIMED FADO (ORIGINAL LYRICS POP) | shock | shock | 4.03 | 7.45 | 3.34 | PRIMED HIP HOP (ORIGINAL LYRICS SAMBA) | confusion | confusion | 3.46 | 6.07 | 3.04 |  |  |
| SYDNEY | z3459608 |  | Dancy | Relaxed | Sexy | Annoyed | Feral | Angry | Happy | Bored | Moderately Agree | 6 | Strongly Disagree | 1 | Moderately Agree | 6 | Moderately Disagree | 2 | Moderately Disagree | 2 | Moderately Disagree | 2 | Neither Agree nor Disagree | 4 | Moderately Agree | 6 | 3459608 | PRIMED OPERA (ORIGINAL LYRICS OPERA) | Romantic | Romantic | 8.32 | 7.59 | 6.08 | PRIMED FADO (ORIGINAL LYRICS FADO) | Emotional | Emotional | 4.36 | 5.75 | 4.29 | PRIMED HEAVY METAL (ORIGINAL LYRICS HEAVY METAL) | Gorey | Gorey | #N/A | #N/A | #N/A | PRIMED HIP HOP (ORIGINAL LYRICS HIP HOP) | | #N/A | #N/A | #N/A | #N/A | PRIMED BOLERO (ORIGINAL LYRICS BOLERO) | Betrayal | Betrayal | 2.28 | 5.37 | 4.18 | PRIMED KOTO (ORIGINAL LYRICS KOTO) | Religious | religion | 5.07 | 5.85 | 5.3 | PRIMED POP (ORIGINAL LYRICS POP) | Nasty | Nasty | 3.58 | 4.89 | 5 | PRIMED SAMBA (ORIGINAL LYRICS SAMBA) | Romantic | Romantic | 8.32 | 7.59 | 6.08 |  |  |
| SYDNEY | z3459931 |  | Entertaining | Lovely | Fun | Interesting | Disgusting | Annoying | Satisfactory | Beautiful | Neither Agree nor Disagree | 4 | Strongly Disagree | 1 | Slightly Disagree | 3 | Strongly Disagree | 1 | I don't know this music | 8 | Strongly Agree | 7 | Neither Agree nor Disagree | 4 | Neither Agree nor Disagree | 4 | 3459931 |  |  | #N/A | #N/A | #N/A | #N/A |  |  | #N/A | #N/A | #N/A | #N/A |  |  | #N/A | #N/A | #N/A | #N/A |  |  | #N/A | #N/A | #N/A | #N/A |  |  | #N/A | #N/A | #N/A | #N/A |  |  | #N/A | #N/A | #N/A | #N/A |  |  | #N/A | #N/A | #N/A | #N/A |  |  | #N/A | #N/A | #N/A | #N/A |  |  |
| SYDNEY | z3460664 |  | body shaking | gliding | relaxation | foreign | agression | inquisitive | dance | boredom | Moderately Agree | 6 | Neither Agree nor Disagree | 4 | Moderately Agree | 6 | Slightly Agree | 5 | Neither Agree nor Disagree | 4 | Neither Agree nor Disagree | 4 | Moderately Agree | 6 | Moderately Agree | 6 | 3460664 | PRIMED POP (ORIGINAL LYRICS OPERA) | arrogance | arrogance | 3.15 | 4.85 | 5.32 | PRIMED OPERA (ORIGINAL LYRICS FADO) | confusion | confusion | 3.46 | 6.07 | 3.04 | PRIMED CUBAN SON (ORIGINAL LYRICS HEAVY METAL) | aggression | aggression | 3.54 | 5.73 | 5.04 | PRIMED SAMBA (ORIGINAL LYRICS HIP HOP) | lust | lust | 7.12 | 6.88 | 5.49 | PRIMED KOTO (ORIGINAL LYRICS BOLERO) | love | love | 8.72 | 6.44 | 7.11 | PRIMED HEAVY METAL (ORIGINAL LYRICS KOTO) | holy | holy | 6.36 | 5.36 | 4.68 | PRIMED FADO (ORIGINAL LYRICS POP) | agreeance | agreeance | #N/A | #N/A | #N/A | PRIMED HIP HOP (ORIGINAL LYRICS SAMBA) | rhythm | rhythm | 7.06 | 5.73 | 6.23 |  |  |
| SYDNEY | z3461130 |  | connected | relaxed | carefree | tranquil | frustration | upbeat | joy | aware | Moderately Disagree | 2 | Strongly Disagree | 1 | Strongly Agree | 7 | Moderately Agree | 6 | Strongly Disagree | 1 | Strongly Disagree | 1 | Strongly Disagree | 1 | Strongly Disagree | 1 | 3461130 | PRIMED POP (ORIGINAL LYRICS OPERA) | admiration | admiration | #N/A | #N/A | #N/A | PRIMED OPERA (ORIGINAL LYRICS FADO) | troubled | troubled | 2.17 | 5.94 | 3.91 | PRIMED CUBAN SON (ORIGINAL LYRICS HEAVY METAL) | powerful | Powerful | 6.84 | 5.83 | 7.19 | PRIMED SAMBA (ORIGINAL LYRICS HIP HOP) | hurt | hurt | 1.9 | 5.85 | 3.33 | PRIMED KOTO (ORIGINAL LYRICS BOLERO) | heartbroken | heartbreak | 1.93 | 5.8 | 3.11 | PRIMED HEAVY METAL (ORIGINAL LYRICS KOTO) | powerful | Powerful | 6.84 | 5.83 | 7.19 | PRIMED FADO (ORIGINAL LYRICS POP) | frustrated | Frustrated | 2.48 | 5.61 | 3.5 | PRIMED HIP HOP (ORIGINAL LYRICS SAMBA) | apprehensive | apprehensive | #N/A | #N/A | #N/A |  |  |
| SYDNEY | 3462165 |  | cruising | calm | sassy | calm | irritated | Chill | no emotion | happy, calm | I don't know this music | 8 | Strongly Disagree | 1 | Neither Agree nor Disagree | 4 | Strongly Agree | 7 | Moderately Agree | 6 | Strongly Agree | 7 | Moderately Agree | 6 | I don't know this music | 8 | 3462165 | PRIMED POP (ORIGINAL LYRICS OPERA) | nothing | nothing | #N/A | #N/A | #N/A | PRIMED OPERA (ORIGINAL LYRICS FADO) | sad | sad | 1.61 | 4.13 | 3.45 | PRIMED CUBAN SON (ORIGINAL LYRICS HEAVY METAL) | revenge | revenge | #N/A | #N/A | #N/A | PRIMED SAMBA (ORIGINAL LYRICS HIP HOP) | confusion | confusion | 3.46 | 6.07 | 3.04 | PRIMED KOTO (ORIGINAL LYRICS BOLERO) | sad | sad | 1.61 | 4.13 | 3.45 | PRIMED HEAVY METAL (ORIGINAL LYRICS KOTO) | spiritual | spirit | 7 | 5.56 | 5.82 | PRIMED FADO (ORIGINAL LYRICS POP) | confusion | confusion | 3.46 | 6.07 | 3.04 | PRIMED HIP HOP (ORIGINAL LYRICS SAMBA) | selfish love | selfish | 2.42 | 5.5 | 4.64 |  |  |
| SYDNEY | z3463563 |  | Relaxed | Romantic | Happy | Nothing | Angst-y | Agitated | Energised | Grand | Slightly Disagree | 3 | Strongly Disagree | 1 | Slightly Agree | 5 | Moderately Disagree | 2 | Moderately Disagree | 2 | Neither Agree nor Disagree | 4 | Moderately Disagree | 2 | Neither Agree nor Disagree | 4 | 3463563 | UNPRIMED (ORIGINAL LYRICS OPERA) | Nothing | nothing | #N/A | #N/A | #N/A | UNPRIMED (ORIGINAL LYRICS FADO) | Confusion | confusion | 3.46 | 6.07 | 3.04 | UNPRIMED (ORIGINAL LYRICS HEAVY METAL) | Disgust | disgusting | 2.96 | 5.18 | 3.64 | UNPRIMED (ORIGINAL LYRICS HIP HOP) | Sympathy | Sympathy | 5.33 | 5.03 | 4.73 | UNPRIMED (ORIGINAL LYRICS BOLERO) | Loss | Loss | 1.89 | 5.78 | 2.38 | UNPRIMED (ORIGINAL LYRICS BOLERO) | Hopeful | Hopeful | 7.1 | 5.78 | 5.41 | UNPRIMED (ORIGINAL LYRICS POP) | Repulsion | repulsive | 2.86 | 4.84 | 4.76 | UNPRIMED (ORIGINAL LYRICS SAMBA) | Anxious | Anxious | 4.81 | 6.92 | 5.33 |  |  |
| SYDNEY | z3463641 |  | state of entrainment | Upset | Happy | Peaceful | Frustrated | melancholy | Cheerful | Anxious | Slightly Agree | 5 | Strongly Disagree | 1 | Moderately Agree | 6 | Strongly Agree | 7 | Neither Agree nor Disagree | 4 | Slightly Agree | 5 | Slightly Agree | 5 | Slightly Agree | 5 | 3463641 | UNPRIMED (ORIGINAL LYRICS OPERA) | disgusted | disgusted | 2.45 | 5.42 | 4.34 | UNPRIMED (ORIGINAL LYRICS FADO) | sombre | sombre | #N/A | #N/A | #N/A | UNPRIMED (ORIGINAL LYRICS HEAVY METAL) | Frightened | Frightened | 2.59 | 6.9 | 3.21 | UNPRIMED (ORIGINAL LYRICS HIP HOP) | sympathetic | sympathetic | 5.74 | 3.99 | 5.59 | UNPRIMED (ORIGINAL LYRICS BOLERO) | Sad | sad | 1.61 | 4.13 | 3.45 | UNPRIMED (ORIGINAL LYRICS BOLERO) | Relaxed | Relaxed | 7 | 2.39 | 5.55 | UNPRIMED (ORIGINAL LYRICS POP) | annoyed | annoy | 2.96 | 5.52 | 4.44 | UNPRIMED (ORIGINAL LYRICS SAMBA) | Anxious | Anxious | 4.81 | 6.92 | 5.33 |  |  |
| SYDNEY | z3463641 |  | Entrainment | Happy | Cheerful | Calm | Frustrated | Melancholy | Entrainment | Relaxed |  |  |  |  |  |  |  |  |  |  |  |  |  |  |  |  | 3463641 |  |  | #N/A | #N/A | #N/A | #N/A |  |  | #N/A | #N/A | #N/A | #N/A |  |  | #N/A | #N/A | #N/A | #N/A |  |  | #N/A | #N/A | #N/A | #N/A |  |  | #N/A | #N/A | #N/A | #N/A |  |  | #N/A | #N/A | #N/A | #N/A |  |  | #N/A | #N/A | #N/A | #N/A |  |  | #N/A | #N/A | #N/A | #N/A |  |  |
| SYDNEY | z3463641 |  | entrainment | happy | cheerful | calm | frustrated | melancholy | Entrainment | relaxed | Strongly Agree | 7 | Strongly Disagree | 1 | Strongly Agree | 7 | Strongly Agree | 7 | Slightly Agree | 5 | Neither Agree nor Disagree | 4 | Slightly Agree | 5 | Slightly Agree | 5 | 3463641 |  |  | #N/A | #N/A | #N/A | #N/A |  |  | #N/A | #N/A | #N/A | #N/A |  |  | #N/A | #N/A | #N/A | #N/A |  |  | #N/A | #N/A | #N/A | #N/A |  |  | #N/A | #N/A | #N/A | #N/A |  |  | #N/A | #N/A | #N/A | #N/A |  |  | #N/A | #N/A | #N/A | #N/A |  |  | #N/A | #N/A | #N/A | #N/A |  |  |
| SYDNEY | z3464531 |  | Excited | Romantic | Happy | Nothing | Hate | Confident | Joy | Triumph | Strongly Agree | 7 | Strongly Disagree | 1 | Strongly Agree | 7 | Moderately Agree | 6 | Slightly Disagree | 3 | Strongly Agree | 7 | Strongly Agree | 7 | Strongly Agree | 7 | 3464531 | UNPRIMED (ORIGINAL LYRICS OPERA) | Anxious | Anxious | 4.81 | 6.92 | 5.33 | UNPRIMED (ORIGINAL LYRICS FADO) | Sad | sad | 1.61 | 4.13 | 3.45 | UNPRIMED (ORIGINAL LYRICS HEAVY METAL) | Reflection | Reflection | 5.97 | 4.38 | 6.1 | UNPRIMED (ORIGINAL LYRICS HIP HOP) | Death | Death | 1.61 | 4.59 | 3.47 | UNPRIMED (ORIGINAL LYRICS BOLERO) | Sad | sad | 1.61 | 4.13 | 3.45 | UNPRIMED (ORIGINAL LYRICS BOLERO) | Forgiveness | Forgiveness | #N/A | #N/A | #N/A | UNPRIMED (ORIGINAL LYRICS POP) | Anger | anger | 2.34 | 7.63 | 5.5 | UNPRIMED (ORIGINAL LYRICS SAMBA) | Love | love | 8.72 | 6.44 | 7.11 |  |  |
| SYDNEY | z3464751 |  | Uplifted | Humble | passionate | Curious | Aggressive | Excited | pretentious | MeloDramatic | Neither Agree nor Disagree | 4 | Slightly Agree | 5 | Neither Agree nor Disagree | 4 | Slightly Agree | 5 | Slightly Agree | 5 | Neither Agree nor Disagree | 4 | Neither Agree nor Disagree | 4 | Neither Agree nor Disagree | 4 | 3464751 | UNPRIMED (ORIGINAL LYRICS OPERA) | sultry | sultry | #N/A | #N/A | #N/A | UNPRIMED (ORIGINAL LYRICS FADO) | pride | Pride | 7 | 5.83 | 7.06 | UNPRIMED (ORIGINAL LYRICS HEAVY METAL) | vicious | vicious | 3.47 | 5.43 | 5.01 | UNPRIMED (ORIGINAL LYRICS HIP HOP) | pity | pity | 3.37 | 3.72 | 4.12 | UNPRIMED (ORIGINAL LYRICS BOLERO) | heartbroken | heartbreak | 1.93 | 5.8 | 3.11 | UNPRIMED (ORIGINAL LYRICS BOLERO) | heavy | Heavy | 3.69 | 4.58 | 4.1 | UNPRIMED (ORIGINAL LYRICS POP) | confused | confused | 3.21 | 6.03 | 4.24 | UNPRIMED (ORIGINAL LYRICS SAMBA) | introspective | Introspective | #N/A | #N/A | #N/A |  |  |
| SYDNEY | 3464925 |  | excited | relaxed | Happy | Mournful | Angry | strong | happy | enchanted | Strongly Agree | 7 | Strongly Disagree | 1 | Strongly Agree | 7 | Strongly Agree | 7 | Neither Agree nor Disagree | 4 | Strongly Agree | 7 | Strongly Agree | 7 | Strongly Agree | 7 | 3464925 | PRIMED OPERA (ORIGINAL LYRICS OPERA) | confident | confident | 7.98 | 6.22 | 7.68 | PRIMED FADO (ORIGINAL LYRICS FADO) | sad | sad | 1.61 | 4.13 | 3.45 | PRIMED HEAVY METAL (ORIGINAL LYRICS HEAVY METAL) | distress | distress | 2.67 | 5.7 | 3.03 | PRIMED HIP HOP (ORIGINAL LYRICS HIP HOP) | sad | sad | 1.61 | 4.13 | 3.45 | PRIMED BOLERO (ORIGINAL LYRICS BOLERO) | betrayal | Betrayal | 2.28 | 5.37 | 4.18 | PRIMED KOTO (ORIGINAL LYRICS KOTO) | Religious | religion | 5.07 | 5.85 | 5.3 | PRIMED POP (ORIGINAL LYRICS POP) | aggressive | aggressive | 5.1 | 5.83 | 5.59 | PRIMED SAMBA (ORIGINAL LYRICS SAMBA) | sad | sad | 1.61 | 4.13 | 3.45 |  |  |
| SYDNEY | z3465132 |  | Lively | Relaxed | Chill | Meditative | Aggravated | Agitated | Energised | Calm | Moderately Agree | 6 | Slightly Disagree | 3 | Strongly Agree | 7 | Slightly Disagree | 3 | Moderately Agree | 6 | Strongly Agree | 7 | Moderately Agree | 6 | Slightly Disagree | 3 | 3465132 | PRIMED POP (ORIGINAL LYRICS OPERA) | Uncomfortable | Uncomfortable | 2.97 | 6.06 | 3.42 | PRIMED OPERA (ORIGINAL LYRICS FADO) | Empowered | power | 6.54 | 6.67 | 7.28 | PRIMED CUBAN SON (ORIGINAL LYRICS HEAVY METAL) | Agitated | Agitated | #N/A | #N/A | #N/A | PRIMED SAMBA (ORIGINAL LYRICS HIP HOP) | contemplative | contemplative | #N/A | #N/A | #N/A | PRIMED KOTO (ORIGINAL LYRICS BOLERO) | Pensive | Pensive | #N/A | #N/A | #N/A | PRIMED HEAVY METAL (ORIGINAL LYRICS KOTO) | Reflective | Reflective | #N/A | #N/A | #N/A | PRIMED FADO (ORIGINAL LYRICS POP) | Angry | angry | 2.85 | 7.17 | 5.55 | PRIMED HIP HOP (ORIGINAL LYRICS SAMBA) | regret | Regret | 2.26 | 5.67 | 3.23 |  |  |
| SYDNEY | z3465237 |  | relaxed | nostalgic | loving | introspective | angry | pissed off | energetic, confident, sassy, happy | amazed | Moderately Agree | 6 | Strongly Agree | 7 | Strongly Agree | 7 | Strongly Agree | 7 | I don't know this music | 8 | Moderately Agree | 6 | Strongly Agree | 7 | Strongly Agree | 7 | 3465237 | PRIMED POP (ORIGINAL LYRICS OPERA) | fear | fear | 2.76 | 6.96 | 3.22 | PRIMED OPERA (ORIGINAL LYRICS FADO) | upset | upset | 2 | 5.86 | 4.08 | PRIMED CUBAN SON (ORIGINAL LYRICS HEAVY METAL) | lonely | lonely | 2.17 | 4.51 | 2.95 | PRIMED SAMBA (ORIGINAL LYRICS HIP HOP) | betrayel | Betrayal | 2.28 | 5.37 | 4.18 | PRIMED KOTO (ORIGINAL LYRICS BOLERO) | discouraged | discouraged | 3 | 4.53 | 3.61 | PRIMED HEAVY METAL (ORIGINAL LYRICS KOTO) | sad | sad | 1.61 | 4.13 | 3.45 | PRIMED FADO (ORIGINAL LYRICS POP) | no special feeling | no special feeling | #N/A | #N/A | #N/A | PRIMED HIP HOP (ORIGINAL LYRICS SAMBA) | sperturbed | sperturbed | #N/A | #N/A | #N/A |  |  |
| SYDNEY | 3465491 |  | high spirited | Sad | excitment | Nostalgia | anger / aggression | bored | boredom | excitement | Slightly Agree | 5 | Slightly Agree | 5 | Slightly Disagree | 3 | Neither Agree nor Disagree | 4 | Strongly Disagree | 1 | Moderately Agree | 6 | Moderately Disagree | 2 | Slightly Agree | 5 | 3465491 | PRIMED OPERA (ORIGINAL LYRICS OPERA) | laugher | laugher | #N/A | #N/A | #N/A | PRIMED FADO (ORIGINAL LYRICS FADO) | sadness | sadness | 2.21 | 5.21 | 2.82 | PRIMED HEAVY METAL (ORIGINAL LYRICS HEAVY METAL) | scared | scared | 2.78 | 6.82 | 2.94 | PRIMED HIP HOP (ORIGINAL LYRICS HIP HOP) | contemplative | contemplative | #N/A | #N/A | #N/A | PRIMED BOLERO (ORIGINAL LYRICS BOLERO) | faith | faith | 6.57 | 5.73 | 5.6 | PRIMED KOTO (ORIGINAL LYRICS KOTO) | cleansing | cleansing | #N/A | #N/A | #N/A | PRIMED POP (ORIGINAL LYRICS POP) | angry | angry | 2.85 | 7.17 | 5.55 | PRIMED SAMBA (ORIGINAL LYRICS SAMBA) | estranged | estranged | #N/A | #N/A | #N/A |  |  |
| SYDNEY | 3465491 |  | sexy | LONGING, NOSTALGIA | cool | strange | angry | angry | annoyed | excited | Moderately Agree | 6 | Moderately Disagree | 2 | Moderately Disagree | 2 | Slightly Agree | 5 | Strongly Disagree | 1 | Moderately Agree | 6 | Neither Agree nor Disagree | 4 | Slightly Agree | 5 | 3465491 | UNPRIMED (ORIGINAL LYRICS OPERA) | diadain | disdain | #N/A | #N/A | #N/A | UNPRIMED (ORIGINAL LYRICS FADO) | low spirits | low spirits | #N/A | #N/A | #N/A | UNPRIMED (ORIGINAL LYRICS HEAVY METAL) | hatred | Hatred | 1.98 | 6.66 | 4.3 | UNPRIMED (ORIGINAL LYRICS HIP HOP) | sadness | sadness | 2.21 | 5.21 | 2.82 | UNPRIMED (ORIGINAL LYRICS BOLERO) | love broken | heartbreak | 1.93 | 5.8 | 3.11 | UNPRIMED (ORIGINAL LYRICS BOLERO) | despair | Despair | 2.99 | 4.49 | 4.3 | UNPRIMED (ORIGINAL LYRICS POP) | determination | determination | #N/A | #N/A | #N/A | UNPRIMED (ORIGINAL LYRICS SAMBA) | Insecure | insecure | 2.36 | 5.56 | 2.33 |  |  |
| SYDNEY | 3466532 |  | comfortable | relax | happy | strange | anxious | excited | happy | relax | Neither Agree nor Disagree | 4 | I don't know this music | 8 | Strongly Agree | 7 | Strongly Agree | 7 | Moderately Agree | 6 | Strongly Agree | 7 | I don't know this music | 8 | Slightly Agree | 5 | 3466532 |  |  | #N/A | #N/A | #N/A | #N/A |  |  | #N/A | #N/A | #N/A | #N/A |  |  | #N/A | #N/A | #N/A | #N/A |  |  | #N/A | #N/A | #N/A | #N/A |  |  | #N/A | #N/A | #N/A | #N/A |  |  | #N/A | #N/A | #N/A | #N/A |  |  | #N/A | #N/A | #N/A | #N/A |  |  | #N/A | #N/A | #N/A | #N/A |  |  |
| SYDNEY | z3484164 |  | Happy | Sad | Relaxed | Weird/Eerie | Scared | Confident | Happy | Content | Slightly Disagree | 3 | Strongly Disagree | 1 | Strongly Agree | 7 | Slightly Agree | 5 | Strongly Disagree | 1 | Slightly Agree | 5 | Slightly Disagree | 3 | Slightly Disagree | 3 | 3484164 | PRIMED OPERA (ORIGINAL LYRICS OPERA) | Insecure | insecure | 2.36 | 5.56 | 2.33 | PRIMED FADO (ORIGINAL LYRICS FADO) | Sad | sad | 1.61 | 4.13 | 3.45 | PRIMED HEAVY METAL (ORIGINAL LYRICS HEAVY METAL) | Antisocial | Antisocial | #N/A | #N/A | #N/A | PRIMED HIP HOP (ORIGINAL LYRICS HIP HOP) | Longing | Longing | #N/A | #N/A | #N/A | PRIMED BOLERO (ORIGINAL LYRICS BOLERO) | Unfaithfulness | Unfaithfulness | #N/A | #N/A | #N/A | PRIMED KOTO (ORIGINAL LYRICS KOTO) | Spiritual | spirit | 7 | 5.56 | 5.82 | PRIMED POP (ORIGINAL LYRICS POP) | Reckless | Reckless | 4.37 | 5.74 | 5.22 | PRIMED SAMBA (ORIGINAL LYRICS SAMBA) | Sad | sad | 1.61 | 4.13 | 3.45 |  |  |
| SYDNEY | z3484164 |  | Romantic | Longing | Romantic | Eerie | Scared | Confident | Groovy | Dramatic | Moderately Agree | 6 | Strongly Disagree | 1 | Strongly Agree | 7 | Strongly Agree | 7 | Moderately Disagree | 2 | Moderately Agree | 6 | Slightly Agree | 5 | Slightly Agree | 5 | 3484164 | UNPRIMED (ORIGINAL LYRICS OPERA) | Sexual | Sexual | 6.7 | 6.33 | 5.73 | UNPRIMED (ORIGINAL LYRICS FADO) | Sorrow | sorrow | 2.32 | 4.48 | 3.67 | UNPRIMED (ORIGINAL LYRICS HEAVY METAL) | Ruthless | Ruthless | #N/A | #N/A | #N/A | UNPRIMED (ORIGINAL LYRICS HIP HOP) | Betrayed | Betrayal | 2.28 | 5.37 | 4.18 | UNPRIMED (ORIGINAL LYRICS BOLERO) | Unfaithfulness | Unfaithfulness | #N/A | #N/A | #N/A | UNPRIMED (ORIGINAL LYRICS BOLERO) | Hopeful | Hopeful | 7.1 | 5.78 | 5.41 | UNPRIMED (ORIGINAL LYRICS POP) | Angry | angry | 2.85 | 7.17 | 5.55 | UNPRIMED (ORIGINAL LYRICS SAMBA) | | #N/A | #N/A | #N/A | #N/A |  |  |
| SYDNEY | z3485826 |  | Lust | Romantic | Love | relaxation | Pumped | Angst | relaxation | anticipation | Strongly Agree | 7 | Strongly Agree | 7 | Neither Agree nor Disagree | 4 | Strongly Disagree | 1 | Strongly Agree | 7 | Strongly Agree | 7 | Strongly Agree | 7 | Strongly Agree | 7 | 3485826 | UNPRIMED (ORIGINAL LYRICS OPERA) | lust | lust | 7.12 | 6.88 | 5.49 | UNPRIMED (ORIGINAL LYRICS FADO) | sadness | sadness | 2.21 | 5.21 | 2.82 | UNPRIMED (ORIGINAL LYRICS HEAVY METAL) | pumped | pumped | #N/A | #N/A | #N/A | UNPRIMED (ORIGINAL LYRICS HIP HOP) | boredom | boring | 3.38 | 2.29 | 4.18 | UNPRIMED (ORIGINAL LYRICS BOLERO) | sadness | sadness | 2.21 | 5.21 | 2.82 | UNPRIMED (ORIGINAL LYRICS BOLERO) | anticipation | anticipation | #N/A | #N/A | #N/A | UNPRIMED (ORIGINAL LYRICS POP) | disgust | disgusting | 2.96 | 5.18 | 3.64 | UNPRIMED (ORIGINAL LYRICS SAMBA) | sadness | sadness | 2.21 | 5.21 | 2.82 |  |  |
| SYDNEY | z3489357 |  | relaxing | expressive | uplifting | meditative | grudge | expressive | fun | intense | Slightly Agree | 5 | Slightly Disagree | 3 | Moderately Agree | 6 | Moderately Agree | 6 | Moderately Disagree | 2 | Strongly Agree | 7 | Slightly Agree | 5 | Moderately Agree | 6 | 3489357 | PRIMED OPERA (ORIGINAL LYRICS OPERA) | magical | magical | 7.46 | 5.95 | 5.73 | PRIMED FADO (ORIGINAL LYRICS FADO) | moving | moving | #N/A | #N/A | #N/A | PRIMED HEAVY METAL (ORIGINAL LYRICS HEAVY METAL) | unbreakable | unbreakable | #N/A | #N/A | #N/A | PRIMED HIP HOP (ORIGINAL LYRICS HIP HOP) | strong | strong | 7.11 | 5.92 | 6.92 | PRIMED BOLERO (ORIGINAL LYRICS BOLERO) | heartbroken | heartbreak | 1.93 | 5.8 | 3.11 | PRIMED KOTO (ORIGINAL LYRICS KOTO) | calm | calm | 6.73 | 3.6 | 6.37 | PRIMED POP (ORIGINAL LYRICS POP) | angry | angry | 2.85 | 7.17 | 5.55 | PRIMED SAMBA (ORIGINAL LYRICS SAMBA) | emotional | Emotional | 4.36 | 5.75 | 4.29 |  |  |
| SYDNEY | z3493350 |  | mess | peace | happy | strange | heavy | active | happy | comfortable | Neither Agree nor Disagree | 4 | Neither Agree nor Disagree | 4 | Strongly Agree | 7 | Moderately Agree | 6 | Slightly Disagree | 3 | Neither Agree nor Disagree | 4 | Neither Agree nor Disagree | 4 | Neither Agree nor Disagree | 4 | 3493350 | UNPRIMED (ORIGINAL LYRICS OPERA) | peace | peace | 7.72 | 2.95 | 5.45 | UNPRIMED (ORIGINAL LYRICS FADO) | desperate | desperate | 2.77 | 5.03 | 3.51 | UNPRIMED (ORIGINAL LYRICS HEAVY METAL) | clam | clam | #N/A | #N/A | #N/A | UNPRIMED (ORIGINAL LYRICS HIP HOP) | sad | sad | 1.61 | 4.13 | 3.45 | UNPRIMED (ORIGINAL LYRICS BOLERO) | heavy | Heavy | 3.69 | 4.58 | 4.1 | UNPRIMED (ORIGINAL LYRICS BOLERO) | clean | clean | 7.23 | 4.8 | 6.9 | UNPRIMED (ORIGINAL LYRICS POP) | strong | strong | 7.11 | 5.92 | 6.92 | UNPRIMED (ORIGINAL LYRICS SAMBA) | sad | sad | 1.61 | 4.13 | 3.45 |  |  |
| SYDNEY | 3509020 |  | Joy and energy | Relaxed | Festive (the first piece)  Relaxed (the second one) | Confusion | Couldn't finish the piece, it really annoys me | it makes me feel like dancing to their rythm | careless, positive | Dreamy and disconnected | Moderately Disagree | 2 | Strongly Disagree | 1 | Moderately Agree | 6 | Neither Agree nor Disagree | 4 | Strongly Disagree | 1 | Slightly Agree | 5 | Neither Agree nor Disagree | 4 | Moderately Agree | 6 | 3509020 | UNPRIMED (ORIGINAL LYRICS OPERA) | Curiosity | Curiosity | 6.74 | 6.08 | 5.46 | UNPRIMED (ORIGINAL LYRICS FADO) | Quest | Quest | #N/A | #N/A | #N/A | UNPRIMED (ORIGINAL LYRICS HEAVY METAL) | Pain | pain | 2.13 | 6.5 | 3.71 | UNPRIMED (ORIGINAL LYRICS HIP HOP) | Hopelessness | hopeless | 2.27 | 4.28 | 2.96 | UNPRIMED (ORIGINAL LYRICS BOLERO) | Sadness | sadness | 2.21 | 5.21 | 2.82 | UNPRIMED (ORIGINAL LYRICS BOLERO) | Prayer | Prayer | 6.24 | 5.14 | 5.64 | UNPRIMED (ORIGINAL LYRICS POP) | Anger | anger | 2.34 | 7.63 | 5.5 | UNPRIMED (ORIGINAL LYRICS SAMBA) | Revelation | Revelation | #N/A | #N/A | #N/A |  |  |
| SYDNEY | 3509020 |  |  |  |  |  |  |  |  |  |  |  |  |  |  |  |  |  |  |  |  |  |  |  |  |  | 3509020 |  |  | #N/A | #N/A | #N/A | #N/A |  |  | #N/A | #N/A | #N/A | #N/A |  |  | #N/A | #N/A | #N/A | #N/A |  |  | #N/A | #N/A | #N/A | #N/A |  |  | #N/A | #N/A | #N/A | #N/A |  |  | #N/A | #N/A | #N/A | #N/A |  |  | #N/A | #N/A | #N/A | #N/A |  |  | #N/A | #N/A | #N/A | #N/A |  |  |
| SYDNEY | Z5011742 |  | Joyful | Immersed | Passionate | Grave | Whiny | Energetic | Active | Excited | Slightly Agree | 5 | Moderately Disagree | 2 | Moderately Agree | 6 | Slightly Agree | 5 | Neither Agree nor Disagree | 4 | Slightly Agree | 5 | Neither Agree nor Disagree | 4 | Neither Agree nor Disagree | 4 | 5011742 | UNPRIMED (ORIGINAL LYRICS OPERA) | Proud | Proud | 8.03 | 5.56 | 6.74 | UNPRIMED (ORIGINAL LYRICS FADO) | Miss | Miss | #N/A | #N/A | #N/A | UNPRIMED (ORIGINAL LYRICS HEAVY METAL) | Anger | anger | 2.34 | 7.63 | 5.5 | UNPRIMED (ORIGINAL LYRICS HIP HOP) | Sad | sad | 1.61 | 4.13 | 3.45 | UNPRIMED (ORIGINAL LYRICS BOLERO) | Depressed | Depressed | 1.83 | 4.72 | 2.74 | UNPRIMED (ORIGINAL LYRICS BOLERO) | Down | Down | #N/A | #N/A | #N/A | UNPRIMED (ORIGINAL LYRICS POP) | Strong | strong | 7.11 | 5.92 | 6.92 | UNPRIMED (ORIGINAL LYRICS SAMBA) | Weak | Weak | 2.64 | 3.78 | 3.44 |  |  |
| SYDNEY | z5011742 |  |  | fun |  |  |  |  |  |  |  |  |  |  |  |  |  |  |  |  |  |  |  |  |  |  | 5011742 |  |  | #N/A | #N/A | #N/A | #N/A |  |  | #N/A | #N/A | #N/A | #N/A |  |  | #N/A | #N/A | #N/A | #N/A |  |  | #N/A | #N/A | #N/A | #N/A |  |  | #N/A | #N/A | #N/A | #N/A |  |  | #N/A | #N/A | #N/A | #N/A |  |  | #N/A | #N/A | #N/A | #N/A |  |  | #N/A | #N/A | #N/A | #N/A |  |  |
| SYDNEY | z5014763 |  | energetic | romantic | groovy | nostalgia | raging | determined | sprightly | peaceful, joyous and hopeful | Slightly Agree | 5 | Moderately Disagree | 2 | Moderately Agree | 6 | Moderately Agree | 6 | Neither Agree nor Disagree | 4 | Moderately Agree | 6 | Neither Agree nor Disagree | 4 | Neither Agree nor Disagree | 4 | 5014763 | UNPRIMED (ORIGINAL LYRICS OPERA) | narcisism | narcisism | #N/A | #N/A | #N/A | UNPRIMED (ORIGINAL LYRICS FADO) | agony | agony | 2.43 | 6.06 | 4.02 | UNPRIMED (ORIGINAL LYRICS HEAVY METAL) | rage | rage | 2.41 | 8.17 | 5.68 | UNPRIMED (ORIGINAL LYRICS HIP HOP) | tragedy | tragedy | 1.78 | 6.24 | 3.5 | UNPRIMED (ORIGINAL LYRICS BOLERO) | heartbreak | heartbreak | 1.93 | 5.8 | 3.11 | UNPRIMED (ORIGINAL LYRICS BOLERO) | righteousness | righteousness | #N/A | #N/A | #N/A | UNPRIMED (ORIGINAL LYRICS POP) | anarchy | anarchy | 3.58 | 5.48 | 3.65 | UNPRIMED (ORIGINAL LYRICS SAMBA) | conflicted | conflicted | #N/A | #N/A | #N/A |  |  |
| SYDNEY | z5014870 |  | relaxed | Jovial | relaxed | nostalgic | uncomfortable | uncomfortable | excited | relaxed | Neither Agree nor Disagree | 4 | Strongly Disagree | 1 | Moderately Agree | 6 | Moderately Agree | 6 | Moderately Agree | 6 | Strongly Agree | 7 | I don't know this music | 8 | I don't know this music | 8 | 5014870 | PRIMED OPERA (ORIGINAL LYRICS OPERA) | nonplussed | nonplussed | #N/A | #N/A | #N/A | PRIMED FADO (ORIGINAL LYRICS FADO) | sombre | sombre | #N/A | #N/A | #N/A | PRIMED HEAVY METAL (ORIGINAL LYRICS HEAVY METAL) | moved | moved | #N/A | #N/A | #N/A | PRIMED HIP HOP (ORIGINAL LYRICS HIP HOP) | sad | sad | 1.61 | 4.13 | 3.45 | PRIMED BOLERO (ORIGINAL LYRICS BOLERO) | sombre | sombre | #N/A | #N/A | #N/A | PRIMED KOTO (ORIGINAL LYRICS KOTO) | confused | confused | 3.21 | 6.03 | 4.24 | PRIMED POP (ORIGINAL LYRICS POP) | offended | offended | #N/A | #N/A | #N/A | PRIMED SAMBA (ORIGINAL LYRICS SAMBA) | shaken | shaken | #N/A | #N/A | #N/A |  |  |
| SYDNEY | z5015219 |  | slightly irritated because of the minor keys | distaste | interest | wonder | impressed but intensity | relaxed | frustrated | Appreciation | Slightly Disagree | 3 | Moderately Disagree | 2 | Slightly Agree | 5 | Moderately Agree | 6 | Slightly Agree | 5 | Slightly Agree | 5 | Moderately Disagree | 2 | Moderately Disagree | 2 | 5015219 | UNPRIMED (ORIGINAL LYRICS OPERA) | laughter | laughter | 8.45 | 6.75 | 6.45 | UNPRIMED (ORIGINAL LYRICS FADO) | yearning | yearning | #N/A | #N/A | #N/A | UNPRIMED (ORIGINAL LYRICS HEAVY METAL) | interest | interest | 6.97 | 5.66 | 5.89 | UNPRIMED (ORIGINAL LYRICS HIP HOP) | sorrow | sorrow | 2.32 | 4.48 | 3.67 | UNPRIMED (ORIGINAL LYRICS BOLERO) | annoyance | annoyance | 2.97 | 5.18 | 4.21 | UNPRIMED (ORIGINAL LYRICS BOLERO) | disinterested | disinterested | #N/A | #N/A | #N/A | UNPRIMED (ORIGINAL LYRICS POP) | anger | anger | 2.34 | 7.63 | 5.5 | UNPRIMED (ORIGINAL LYRICS SAMBA) | apprehensive | apprehensive | #N/A | #N/A | #N/A |  |  |
| SYDNEY | z5015594 |  | calm | relaxed | joy | confused | anxious | upbeat | upbeat | courageous | Moderately Agree | 6 | Strongly Disagree | 1 | Moderately Agree | 6 | Strongly Agree | 7 | I don't know this music | 8 | Neither Agree nor Disagree | 4 | Slightly Agree | 5 | Moderately Agree | 6 | 5015594 | PRIMED POP (ORIGINAL LYRICS OPERA) | pretentious | pretentious | #N/A | #N/A | #N/A | PRIMED OPERA (ORIGINAL LYRICS FADO) | dispair | despair | 2.99 | 4.49 | 4.3 | PRIMED CUBAN SON (ORIGINAL LYRICS HEAVY METAL) | curious | Curious | 6.08 | 5.82 | 5.42 | PRIMED SAMBA (ORIGINAL LYRICS HIP HOP) | sad | sad | 1.61 | 4.13 | 3.45 | PRIMED KOTO (ORIGINAL LYRICS BOLERO) | upset | upset | 2 | 5.86 | 4.08 | PRIMED HEAVY METAL (ORIGINAL LYRICS KOTO) | prophetic | prophetic | #N/A | #N/A | #N/A | PRIMED FADO (ORIGINAL LYRICS POP) | inspired | Inspired | 7.15 | 6.02 | 6.67 | PRIMED HIP HOP (ORIGINAL LYRICS SAMBA) | sperturbed | sperturbed | #N/A | #N/A | #N/A |  |  |
| SYDNEY | 5015657 |  | jo | bj | bj | bj | nk | jo | bj | bj |  |  |  |  |  |  |  |  |  |  |  |  |  |  |  |  | 5015657 |  |  | #N/A | #N/A | #N/A | #N/A |  |  | #N/A | #N/A | #N/A | #N/A |  |  | #N/A | #N/A | #N/A | #N/A |  |  | #N/A | #N/A | #N/A | #N/A |  |  | #N/A | #N/A | #N/A | #N/A |  |  | #N/A | #N/A | #N/A | #N/A |  |  | #N/A | #N/A | #N/A | #N/A |  |  | #N/A | #N/A | #N/A | #N/A |  |  |
| SYDNEY | 5016005 |  | happy and relaxed | sentiment | confused but amused | interested | annoyed | no particular emotion, happy? | happy | relaxed and exciting, depending on what the music is | Moderately Disagree | 2 | Strongly Disagree | 1 | Slightly Agree | 5 | Slightly Agree | 5 | Slightly Disagree | 3 | Strongly Agree | 7 | Slightly Disagree | 3 | Moderately Disagree | 2 | 5016005 | PRIMED POP (ORIGINAL LYRICS OPERA) | proud | Proud | 8.03 | 5.56 | 6.74 | PRIMED OPERA (ORIGINAL LYRICS FADO) | confused | confused | 3.21 | 6.03 | 4.24 | PRIMED CUBAN SON (ORIGINAL LYRICS HEAVY METAL) | fear | fear | 2.76 | 6.96 | 3.22 | PRIMED SAMBA (ORIGINAL LYRICS HIP HOP) | lost | lost | 2.82 | 5.82 | 2.86 | PRIMED KOTO (ORIGINAL LYRICS BOLERO) | confused | confused | 3.21 | 6.03 | 4.24 | PRIMED HEAVY METAL (ORIGINAL LYRICS KOTO) | confused | confused | 3.21 | 6.03 | 4.24 | PRIMED FADO (ORIGINAL LYRICS POP) | confused | confused | 3.21 | 6.03 | 4.24 | PRIMED HIP HOP (ORIGINAL LYRICS SAMBA) | sad | sad | 1.61 | 4.13 | 3.45 |  |  |
| SYDNEY | z5016297 |  | Happy | Calm | Happy | Anxious | Agitated | Excited | Excited | Excitement | Moderately Agree | 6 | Slightly Disagree | 3 | Strongly Agree | 7 | Slightly Agree | 5 | I don't know this music | 8 | Strongly Agree | 7 | I don't know this music | 8 | I don't know this music | 8 | 5016297 | UNPRIMED (ORIGINAL LYRICS OPERA) | Neutral | Neutral | #N/A | #N/A | #N/A | UNPRIMED (ORIGINAL LYRICS FADO) | Neutral | Neutral | #N/A | #N/A | #N/A | UNPRIMED (ORIGINAL LYRICS HEAVY METAL) | Sadness | sadness | 2.21 | 5.21 | 2.82 | UNPRIMED (ORIGINAL LYRICS HIP HOP) | Neutral | Neutral | #N/A | #N/A | #N/A | UNPRIMED (ORIGINAL LYRICS BOLERO) | Sadness | sadness | 2.21 | 5.21 | 2.82 | UNPRIMED (ORIGINAL LYRICS BOLERO) | Neutral | Neutral | #N/A | #N/A | #N/A | UNPRIMED (ORIGINAL LYRICS POP) | Annoyed | annoy | 2.96 | 5.52 | 4.44 | UNPRIMED (ORIGINAL LYRICS SAMBA) | Confused | confused | 3.21 | 6.03 | 4.24 |  |  |
| SYDNEY | z5016456 |  | Active | Passion | Energy | Eclectic | Chaos | Passion | Stimulation | Granduer | Moderately Disagree | 2 | Moderately Disagree | 2 | Strongly Agree | 7 | Strongly Agree | 7 | Strongly Disagree | 1 | Slightly Agree | 5 | Moderately Disagree | 2 | Moderately Disagree | 2 | 5016456 | PRIMED OPERA (ORIGINAL LYRICS OPERA) | Pride | Pride | 7 | 5.83 | 7.06 | PRIMED FADO (ORIGINAL LYRICS FADO) | Mourning | mourn | 1.8 | 5.87 | 2.87 | PRIMED HEAVY METAL (ORIGINAL LYRICS HEAVY METAL) | Hate | Hate | 2.12 | 6.95 | 5.05 | PRIMED HIP HOP (ORIGINAL LYRICS HIP HOP) | Hopelessness | hopeless | 2.27 | 4.28 | 2.96 | PRIMED BOLERO (ORIGINAL LYRICS BOLERO) | Bittnerness | bitter | 3.31 | 4.4 | 5.17 | PRIMED KOTO (ORIGINAL LYRICS KOTO) | Faith | faith | 6.57 | 5.73 | 5.6 | PRIMED POP (ORIGINAL LYRICS POP) | Anarchy | anarchy | 3.58 | 5.48 | 3.65 | PRIMED SAMBA (ORIGINAL LYRICS SAMBA) | Vulnerability | Vulnerability | #N/A | #N/A | #N/A |  |  |
| SYDNEY | 5017184 |  | bliss | solemn | festive | meditative | angry | swag | happy | calm | Slightly Disagree | 3 | Strongly Disagree | 1 | Moderately Agree | 6 | Moderately Agree | 6 | Slightly Disagree | 3 | Slightly Disagree | 3 | Moderately Disagree | 2 | Moderately Disagree | 2 | 5017184 | UNPRIMED (ORIGINAL LYRICS OPERA) | insecurity | insecure | 2.36 | 5.56 | 2.33 | UNPRIMED (ORIGINAL LYRICS FADO) | suffering | suffer | 1.72 | 6.13 | 2.54 | UNPRIMED (ORIGINAL LYRICS HEAVY METAL) | torment | torment | #N/A | #N/A | #N/A | UNPRIMED (ORIGINAL LYRICS HIP HOP) | bitter | bitter | 3.31 | 4.4 | 5.17 | UNPRIMED (ORIGINAL LYRICS BOLERO) | love | love | 8.72 | 6.44 | 7.11 | UNPRIMED (ORIGINAL LYRICS BOLERO) | religious | religion | 5.07 | 5.85 | 5.3 | UNPRIMED (ORIGINAL LYRICS POP) | anger | anger | 2.34 | 7.63 | 5.5 | UNPRIMED (ORIGINAL LYRICS SAMBA) | afraid | Afraid | 2 | 6.67 | 3.98 |  |  |
| SYDNEY | z5017802 |  | Festive, energetic | Calm | Festive | Solemn | Aggression | Frustration | Happy | Powerful | Strongly Disagree | 1 | Moderately Agree | 6 | Moderately Agree | 6 | Slightly Agree | 5 | Strongly Disagree | 1 | Slightly Agree | 5 | Strongly Disagree | 1 | Strongly Disagree | 1 | 5017802 | UNPRIMED (ORIGINAL LYRICS OPERA) | Inspired | Inspired | 7.15 | 6.02 | 6.67 | UNPRIMED (ORIGINAL LYRICS FADO) | Sad | sad | 1.61 | 4.13 | 3.45 | UNPRIMED (ORIGINAL LYRICS HEAVY METAL) | Anger | anger | 2.34 | 7.63 | 5.5 | UNPRIMED (ORIGINAL LYRICS HIP HOP) | Longing | Longing | #N/A | #N/A | #N/A | UNPRIMED (ORIGINAL LYRICS BOLERO) | Sadness | sadness | 2.21 | 5.21 | 2.82 | UNPRIMED (ORIGINAL LYRICS BOLERO) | Spiritual | spirit | 7 | 5.56 | 5.82 | UNPRIMED (ORIGINAL LYRICS POP) | Frustrated | Frustrated | 2.48 | 5.61 | 3.5 | UNPRIMED (ORIGINAL LYRICS SAMBA) | Submission | Submission | #N/A | #N/A | #N/A |  |  |
| SYDNEY | z5017908 |  | Joy | Reflective | Festive | Meditative/trance like | Aggression | Determined | Energetic | Adventuring | Slightly Disagree | 3 | Strongly Disagree | 1 | Slightly Agree | 5 | Strongly Agree | 7 | Strongly Disagree | 1 | Strongly Disagree | 1 | Moderately Disagree | 2 | Neither Agree nor Disagree | 4 | 5017908 | PRIMED OPERA (ORIGINAL LYRICS OPERA) | Superior | Superior | 6.29 | 5.57 | 6.7 | PRIMED FADO (ORIGINAL LYRICS FADO) | Loss | Loss | 1.89 | 5.78 | 2.38 | PRIMED HEAVY METAL (ORIGINAL LYRICS HEAVY METAL) | Disgust | disgusting | 2.96 | 5.18 | 3.64 | PRIMED HIP HOP (ORIGINAL LYRICS HIP HOP) | Abandoned | Abandoned | #N/A | #N/A | #N/A | PRIMED BOLERO (ORIGINAL LYRICS BOLERO) | Loathing | Loathing | #N/A | #N/A | #N/A | PRIMED KOTO (ORIGINAL LYRICS KOTO) | Relieved | relief | 6.81 | 4.17 | 5.6 | PRIMED POP (ORIGINAL LYRICS POP) | Skepticism | Skepticism | #N/A | #N/A | #N/A | PRIMED SAMBA (ORIGINAL LYRICS SAMBA) | Sadness | sadness | 2.21 | 5.21 | 2.82 |  |  |
| SYDNEY | 5018068 |  | Joyful | Sad | romantic | Artificial Contentedness | Anger | Frustration | love | Courageous | I don't know this music | 8 | Strongly Disagree | 1 | Slightly Agree | 5 | Neither Agree nor Disagree | 4 | I don't know this music | 8 | Moderately Agree | 6 | I don't know this music | 8 | I don't know this music | 8 | 5018068 | UNPRIMED (ORIGINAL LYRICS OPERA) | lust | lust | 7.12 | 6.88 | 5.49 | UNPRIMED (ORIGINAL LYRICS FADO) | Despair | Despair | 2.99 | 4.49 | 4.3 | UNPRIMED (ORIGINAL LYRICS HEAVY METAL) | despair | Despair | 2.99 | 4.49 | 4.3 | UNPRIMED (ORIGINAL LYRICS HIP HOP) | Disappointment | Disappointment | 2.37 | 4.6 | 3.2 | UNPRIMED (ORIGINAL LYRICS BOLERO) | depressed | Depressed | 1.83 | 4.72 | 2.74 | UNPRIMED (ORIGINAL LYRICS BOLERO) | liberated | liberated | #N/A | #N/A | #N/A | UNPRIMED (ORIGINAL LYRICS POP) | aggressive | aggressive | 5.1 | 5.83 | 5.59 | UNPRIMED (ORIGINAL LYRICS SAMBA) | pain | pain | 2.13 | 6.5 | 3.71 |  |  |
| SYDNEY | 5018164 |  | Excited | Love | Celebratory | Focussed | Angry | Energised | Joy | Uplifted | Moderately Agree | 6 | Strongly Disagree | 1 | Strongly Agree | 7 | Strongly Agree | 7 | Neither Agree nor Disagree | 4 | Moderately Agree | 6 | Slightly Agree | 5 | Moderately Agree | 6 | 5018164 | PRIMED OPERA (ORIGINAL LYRICS OPERA) | Suspicious | Suspicious | 3.76 | 6.25 | 4.47 | PRIMED FADO (ORIGINAL LYRICS FADO) | Intrigued | Intrigued | #N/A | #N/A | #N/A | PRIMED HEAVY METAL (ORIGINAL LYRICS HEAVY METAL) | Disgusted | disgusted | 2.45 | 5.42 | 4.34 | PRIMED HIP HOP (ORIGINAL LYRICS HIP HOP) | Confused | confused | 3.21 | 6.03 | 4.24 | PRIMED BOLERO (ORIGINAL LYRICS BOLERO) | Sad | sad | 1.61 | 4.13 | 3.45 | PRIMED KOTO (ORIGINAL LYRICS KOTO) | Cleansed | Cleansed | #N/A | #N/A | #N/A | PRIMED POP (ORIGINAL LYRICS POP) | Angry | angry | 2.85 | 7.17 | 5.55 | PRIMED SAMBA (ORIGINAL LYRICS SAMBA) | Excited | excitement | 7.5 | 7.67 | 6.18 |  |  |
| SYDNEY | z5019420 |  | happy | sad | happy | excitement | overwhelmed | interested | happy | calm | Moderately Agree | 6 | Strongly Disagree | 1 | Moderately Agree | 6 | Neither Agree nor Disagree | 4 | Strongly Agree | 7 | Strongly Agree | 7 | Moderately Agree | 6 | Strongly Agree | 7 | 5019420 | PRIMED POP (ORIGINAL LYRICS OPERA) | vulnerable | vulnerable | 3.14 | 4.53 | 4.02 | PRIMED OPERA (ORIGINAL LYRICS FADO) | excited | excitement | 7.5 | 7.67 | 6.18 | PRIMED CUBAN SON (ORIGINAL LYRICS HEAVY METAL) | angry | angry | 2.85 | 7.17 | 5.55 | PRIMED SAMBA (ORIGINAL LYRICS HIP HOP) | betrayed | Betrayal | 2.28 | 5.37 | 4.18 | PRIMED KOTO (ORIGINAL LYRICS BOLERO) | sadness | sadness | 2.21 | 5.21 | 2.82 | PRIMED HEAVY METAL (ORIGINAL LYRICS KOTO) | angry | angry | 2.85 | 7.17 | 5.55 | PRIMED FADO (ORIGINAL LYRICS POP) | angry | angry | 2.85 | 7.17 | 5.55 | PRIMED HIP HOP (ORIGINAL LYRICS SAMBA) | afraid | Afraid | 2 | 6.67 | 3.98 |  |  |
| SYDNEY | z5019650 |  | drunken | suave | sambatastic | content | ecstatic | chyeaaaaah | Chill | wanderlust | Neither Agree nor Disagree | 4 | Neither Agree nor Disagree | 4 | Moderately Agree | 6 | Moderately Agree | 6 | Slightly Agree | 5 | Slightly Agree | 5 | Slightly Disagree | 3 | Neither Agree nor Disagree | 4 | 5019650 | PRIMED OPERA (ORIGINAL LYRICS OPERA) | superficial | superficial | #N/A | #N/A | #N/A | PRIMED FADO (ORIGINAL LYRICS FADO) | patriotic | patriotic | 6.62 | 5.54 | 6.35 | PRIMED HEAVY METAL (ORIGINAL LYRICS HEAVY METAL) | emotional | Emotional | 4.36 | 5.75 | 4.29 | PRIMED HIP HOP (ORIGINAL LYRICS HIP HOP) | uncomfortable | Uncomfortable | 2.97 | 6.06 | 3.42 | PRIMED BOLERO (ORIGINAL LYRICS BOLERO) | sombre | sombre | #N/A | #N/A | #N/A | PRIMED KOTO (ORIGINAL LYRICS KOTO) | holy | holy | 6.36 | 5.36 | 4.68 | PRIMED POP (ORIGINAL LYRICS POP) | agressive | aggressive | 5.1 | 5.83 | 5.59 | PRIMED SAMBA (ORIGINAL LYRICS SAMBA) | smitten | smitten | #N/A | #N/A | #N/A |  |  |
| SYDNEY | z5020320 |  | Thrilled | Soothe | Carefree | Anxious | Energised | Neutral | annoyed | Indifferent | Moderately Disagree | 2 | Neither Agree nor Disagree | 4 | Neither Agree nor Disagree | 4 | Neither Agree nor Disagree | 4 | Slightly Disagree | 3 | Slightly Disagree | 3 | Slightly Disagree | 3 | Slightly Disagree | 3 | 5020320 | UNPRIMED (ORIGINAL LYRICS OPERA) | Dramatic | Dramatic | #N/A | #N/A | #N/A | UNPRIMED (ORIGINAL LYRICS FADO) | Intrinsic | Intrinsic | #N/A | #N/A | #N/A | UNPRIMED (ORIGINAL LYRICS HEAVY METAL) | Introspective | Introspective | #N/A | #N/A | #N/A | UNPRIMED (ORIGINAL LYRICS HIP HOP) | Indifferent | indifferent | 4.61 | 3.18 | 4.84 | UNPRIMED (ORIGINAL LYRICS BOLERO) | Pure | Pure | #N/A | #N/A | #N/A | UNPRIMED (ORIGINAL LYRICS BOLERO) | Confused | confused | 3.21 | 6.03 | 4.24 | UNPRIMED (ORIGINAL LYRICS POP) | Indifferent | indifferent | 4.61 | 3.18 | 4.84 | UNPRIMED (ORIGINAL LYRICS SAMBA) | Amused | Amused | #N/A | #N/A | #N/A |  |  |
| SYDNEY | 5020393 |  | Happy/dance | Desire | Exciting | Wonder | Power | Angst | Neediness | Relaxing/neutral | Neither Agree nor Disagree | 4 | Strongly Disagree | 1 | Strongly Disagree | 1 | Slightly Disagree | 3 | I don't know this music | 8 | Neither Agree nor Disagree | 4 | I don't know this music | 8 | I don't know this music | 8 | 5020393 | PRIMED POP (ORIGINAL LYRICS OPERA) | Disbelief | Disbelief | #N/A | #N/A | #N/A | PRIMED OPERA (ORIGINAL LYRICS FADO) | Sad | sad | 1.61 | 4.13 | 3.45 | PRIMED CUBAN SON (ORIGINAL LYRICS HEAVY METAL) | Disbelief | Disbelief | #N/A | #N/A | #N/A | PRIMED SAMBA (ORIGINAL LYRICS HIP HOP) | | #N/A | #N/A | #N/A | #N/A | PRIMED KOTO (ORIGINAL LYRICS BOLERO) | Sad | sad | 1.61 | 4.13 | 3.45 | PRIMED HEAVY METAL (ORIGINAL LYRICS KOTO) | Awe | Awe | 6.48 | 5.5 | 4.43 | PRIMED FADO (ORIGINAL LYRICS POP) | Laughter | laughter | 8.45 | 6.75 | 6.45 | PRIMED HIP HOP (ORIGINAL LYRICS SAMBA) | Disbelief | Disbelief | #N/A | #N/A | #N/A |  |  |
| SYDNEY | 5020393 |  | Happy | Longing | Happy | Relaxed | Energetic | Boredom | Boredom | Relaxed | Strongly Disagree | 1 | Strongly Disagree | 1 | Strongly Disagree | 1 | Moderately Disagree | 2 | I don't know this music | 8 | Moderately Disagree | 2 | Strongly Disagree | 1 | Strongly Disagree | 1 | 5020393 |  |  | #N/A | #N/A | #N/A | #N/A | PRIMED FADO (ORIGINAL LYRICS FADO) | Sad | sad | 1.61 | 4.13 | 3.45 | PRIMED HEAVY METAL (ORIGINAL LYRICS HEAVY METAL) | Awe | Awe | 6.48 | 5.5 | 4.43 | PRIMED HIP HOP (ORIGINAL LYRICS HIP HOP) | Worry | Worry | 2.31 | 6 | 2.96 | PRIMED BOLERO (ORIGINAL LYRICS BOLERO) | Sympathy | Sympathy | 5.33 | 5.03 | 4.73 | PRIMED KOTO (ORIGINAL LYRICS KOTO) | Surprise | Surprise | 7.73 | 7.07 | 3.87 | PRIMED POP (ORIGINAL LYRICS POP) | Humoured | Humoured | #N/A | #N/A | #N/A | PRIMED SAMBA (ORIGINAL LYRICS SAMBA) | Sad | sad | 1.61 | 4.13 | 3.45 |  |  |
| SYDNEY | z5020459 |  |  | Longing |  | serenity |  |  |  |  |  |  |  |  |  |  |  |  |  |  |  |  |  |  |  |  | 5020459 | PRIMED POP (ORIGINAL LYRICS OPERA) | Nervous | Nervous | 3.29 | 6.59 | 3.56 | PRIMED OPERA (ORIGINAL LYRICS FADO) | Sadness | sadness | 2.21 | 5.21 | 2.82 | PRIMED CUBAN SON (ORIGINAL LYRICS HEAVY METAL) | Fear | fear | 2.76 | 6.96 | 3.22 | PRIMED SAMBA (ORIGINAL LYRICS HIP HOP) | Longing | Longing | #N/A | #N/A | #N/A | PRIMED KOTO (ORIGINAL LYRICS BOLERO) | Sadness | sadness | 2.21 | 5.21 | 2.82 | PRIMED HEAVY METAL (ORIGINAL LYRICS KOTO) | Fear | fear | 2.76 | 6.96 | 3.22 | PRIMED FADO (ORIGINAL LYRICS POP) | Surprise | Surprise | 7.73 | 7.07 | 3.87 | PRIMED HIP HOP (ORIGINAL LYRICS SAMBA) | Sadness | sadness | 2.21 | 5.21 | 2.82 |  |  |
| SYDNEY | z5020459 |  | Lively | Longing | Love | Serenity | Fear | Empowered | Happy | Excited | Slightly Agree | 5 | Strongly Disagree | 1 | Strongly Agree | 7 | Strongly Agree | 7 | Neither Agree nor Disagree | 4 | Strongly Agree | 7 | Slightly Agree | 5 | Slightly Agree | 5 | 5020459 |  |  | #N/A | #N/A | #N/A | #N/A |  |  | #N/A | #N/A | #N/A | #N/A |  |  | #N/A | #N/A | #N/A | #N/A |  |  | #N/A | #N/A | #N/A | #N/A |  |  | #N/A | #N/A | #N/A | #N/A |  |  | #N/A | #N/A | #N/A | #N/A |  |  | #N/A | #N/A | #N/A | #N/A |  |  | #N/A | #N/A | #N/A | #N/A |  |  |
| SYDNEY | z5020459 |  |  |  |  |  |  |  |  |  |  |  |  |  |  |  |  |  |  |  |  |  |  |  |  |  | 5020459 |  |  | #N/A | #N/A | #N/A | #N/A |  |  | #N/A | #N/A | #N/A | #N/A |  |  | #N/A | #N/A | #N/A | #N/A |  |  | #N/A | #N/A | #N/A | #N/A |  |  | #N/A | #N/A | #N/A | #N/A |  |  | #N/A | #N/A | #N/A | #N/A |  |  | #N/A | #N/A | #N/A | #N/A |  |  | #N/A | #N/A | #N/A | #N/A |  |  |
| SYDNEY | z5020459 |  |  |  |  |  |  |  |  |  |  |  |  |  |  |  |  |  |  |  |  |  |  |  |  |  | 5020459 |  |  | #N/A | #N/A | #N/A | #N/A |  |  | #N/A | #N/A | #N/A | #N/A |  |  | #N/A | #N/A | #N/A | #N/A |  |  | #N/A | #N/A | #N/A | #N/A |  |  | #N/A | #N/A | #N/A | #N/A |  |  | #N/A | #N/A | #N/A | #N/A |  |  | #N/A | #N/A | #N/A | #N/A |  |  | #N/A | #N/A | #N/A | #N/A |  |  |
| SYDNEY | z5020459 |  | Excited | melancholy | Relaxed | Peace | Fear | Empowered | Excited | Calm | Slightly Agree | 5 | Strongly Disagree | 1 | Strongly Agree | 7 | Strongly Agree | 7 | Neither Agree nor Disagree | 4 | Strongly Agree | 7 | Slightly Agree | 5 | Slightly Agree | 5 | 5020459 |  |  | #N/A | #N/A | #N/A | #N/A |  |  | #N/A | #N/A | #N/A | #N/A |  |  | #N/A | #N/A | #N/A | #N/A |  |  | #N/A | #N/A | #N/A | #N/A |  |  | #N/A | #N/A | #N/A | #N/A |  |  | #N/A | #N/A | #N/A | #N/A |  |  | #N/A | #N/A | #N/A | #N/A |  |  | #N/A | #N/A | #N/A | #N/A |  |  |
| SYDNEY | z5020625 |  | Nostalgia | Nostalgia | Nostalgia | Chilled | Anger | Chilled | Happiness | Happiness | Strongly Disagree | 1 | Moderately Disagree | 2 | Slightly Agree | 5 | Moderately Agree | 6 | Strongly Disagree | 1 | Slightly Disagree | 3 | Strongly Disagree | 1 | Strongly Disagree | 1 | 5020625 | UNPRIMED (ORIGINAL LYRICS OPERA) | Confidence | Confidence | 7.04 | 5.52 | 6.42 | UNPRIMED (ORIGINAL LYRICS FADO) | Amazement | Amazement | 7.23 | 6.09 | 5.53 | UNPRIMED (ORIGINAL LYRICS HEAVY METAL) | Guilt | Guilt | 2.14 | 5.36 | 2.96 | UNPRIMED (ORIGINAL LYRICS HIP HOP) | Betrayal | Betrayal | 2.28 | 5.37 | 4.18 | UNPRIMED (ORIGINAL LYRICS BOLERO) | Sadness | sadness | 2.21 | 5.21 | 2.82 | UNPRIMED (ORIGINAL LYRICS BOLERO) | Guilt | Guilt | 2.14 | 5.36 | 2.96 | UNPRIMED (ORIGINAL LYRICS POP) | Anger | anger | 2.34 | 7.63 | 5.5 | UNPRIMED (ORIGINAL LYRICS SAMBA) | Fear | fear | 2.76 | 6.96 | 3.22 |  |  |
| SYDNEY | z5022932 |  | Happiness | Love | Excitement | Annoyance | Irritation | Excitement | Excitement | Nostalgia | Neither Agree nor Disagree | 4 | Strongly Disagree | 1 | Strongly Agree | 7 | Strongly Agree | 7 | Strongly Disagree | 1 | Slightly Disagree | 3 | Strongly Disagree | 1 | Moderately Disagree | 2 | 5022932 | PRIMED OPERA (ORIGINAL LYRICS OPERA) | Fascination | Fascination | #N/A | #N/A | #N/A | PRIMED FADO (ORIGINAL LYRICS FADO) | Anguish | Anguish | #N/A | #N/A | #N/A | PRIMED HEAVY METAL (ORIGINAL LYRICS HEAVY METAL) | Anger | anger | 2.34 | 7.63 | 5.5 | PRIMED HIP HOP (ORIGINAL LYRICS HIP HOP) | Betrayal | Betrayal | 2.28 | 5.37 | 4.18 | PRIMED BOLERO (ORIGINAL LYRICS BOLERO) | Betrayal | Betrayal | 2.28 | 5.37 | 4.18 | PRIMED KOTO (ORIGINAL LYRICS KOTO) | Calm | calm | 6.73 | 3.6 | 6.37 | PRIMED POP (ORIGINAL LYRICS POP) | Anger | anger | 2.34 | 7.63 | 5.5 | PRIMED SAMBA (ORIGINAL LYRICS SAMBA) | Sadness | sadness | 2.21 | 5.21 | 2.82 |  |  |
| SYDNEY | z5023151 |  | Happiness, feelings when you are having a good day | Sounds like something from a foreign romance movie. For me personally, I felt like I was over seas on a holiday in Europe as it was very relaxing. | Happy, same as the song before, feeling relaxed and feeling as if something good is going to happen | Relaxing and could fall into deep sleep. Feeling content | the first piece made me feel agitated and angry whereas the 2nd piece made me feel abit more content . I guess it was because the first piece was more aggressive and messy. | Bouncy and wanting to dance. | personally i dont like pop music so I was happy nor angry I was just feeling content | sleepy and could fall into a deep sleep. | Neither Agree nor Disagree | 4 | Neither Agree nor Disagree | 4 | Neither Agree nor Disagree | 4 | Neither Agree nor Disagree | 4 | Slightly Agree | 5 | Moderately Agree | 6 | Slightly Agree | 5 | Slightly Agree | 5 | 5023151 | PRIMED POP (ORIGINAL LYRICS OPERA) | confidence | Confidence | 7.04 | 5.52 | 6.42 | PRIMED OPERA (ORIGINAL LYRICS FADO) | sad | sad | 1.61 | 4.13 | 3.45 | PRIMED CUBAN SON (ORIGINAL LYRICS HEAVY METAL) | horror | horror | 2.76 | 7.21 | 4.63 | PRIMED SAMBA (ORIGINAL LYRICS HIP HOP) | sad | sad | 1.61 | 4.13 | 3.45 | PRIMED KOTO (ORIGINAL LYRICS BOLERO) | sadness and heartbreak | sad | 1.61 | 4.13 | 3.45 | PRIMED HEAVY METAL (ORIGINAL LYRICS KOTO) | disgust | disgusting | 2.96 | 5.18 | 3.64 | PRIMED FADO (ORIGINAL LYRICS POP) | angry | angry | 2.85 | 7.17 | 5.55 | PRIMED HIP HOP (ORIGINAL LYRICS SAMBA) | sad | sad | 1.61 | 4.13 | 3.45 |  |  |
| SYDNEY | 5027332 |  | dislike | soft and relax | wired | strange | loud | energetic | bright | classic | Strongly Disagree | 1 | Neither Agree nor Disagree | 4 | Strongly Agree | 7 | Strongly Agree | 7 | Strongly Disagree | 1 | Strongly Disagree | 1 | Strongly Disagree | 1 | Strongly Disagree | 1 | 5027332 | PRIMED OPERA (ORIGINAL LYRICS OPERA) | proud | Proud | 8.03 | 5.56 | 6.74 | PRIMED FADO (ORIGINAL LYRICS FADO) | sad | sad | 1.61 | 4.13 | 3.45 | PRIMED HEAVY METAL (ORIGINAL LYRICS HEAVY METAL) | strong | strong | 7.11 | 5.92 | 6.92 | PRIMED HIP HOP (ORIGINAL LYRICS HIP HOP) | sad | sad | 1.61 | 4.13 | 3.45 | PRIMED BOLERO (ORIGINAL LYRICS BOLERO) | hurted | hurt | 1.9 | 5.85 | 3.33 | PRIMED KOTO (ORIGINAL LYRICS KOTO) | wired | wired | #N/A | #N/A | #N/A | PRIMED POP (ORIGINAL LYRICS POP) | happy | happy | 8.21 | 6.49 | 6.63 | PRIMED SAMBA (ORIGINAL LYRICS SAMBA) | afraid | Afraid | 2 | 6.67 | 3.98 |  |  |
| SYDNEY | 5033016 |  | exhilarated | relaxed | amused | serene | anger | energized | happiness | harmonious | Strongly Disagree | 1 | Neither Agree nor Disagree | 4 | Moderately Agree | 6 | Moderately Agree | 6 | Strongly Disagree | 1 | Moderately Disagree | 2 | Strongly Disagree | 1 | Strongly Disagree | 1 | 5033016 |  |  | #N/A | #N/A | #N/A | #N/A | PRIMED FADO (ORIGINAL LYRICS FADO) | sadness | sadness | 2.21 | 5.21 | 2.82 | PRIMED HEAVY METAL (ORIGINAL LYRICS HEAVY METAL) | malicious | malicious | 2.7 | 5.19 | 4.92 | PRIMED HIP HOP (ORIGINAL LYRICS HIP HOP) | sadness | sadness | 2.21 | 5.21 | 2.82 | PRIMED BOLERO (ORIGINAL LYRICS BOLERO) | betrayal | Betrayal | 2.28 | 5.37 | 4.18 | PRIMED KOTO (ORIGINAL LYRICS KOTO) | intrigued | Intrigued | #N/A | #N/A | #N/A | PRIMED POP (ORIGINAL LYRICS POP) | excited | excitement | 7.5 | 7.67 | 6.18 | PRIMED SAMBA (ORIGINAL LYRICS SAMBA) | heartbroken | heartbreak | 1.93 | 5.8 | 3.11 |  |  |
| SYDNEY | z5039171 |  | Nostalgia | Wistfulness | Happiness | Excitement | Anger | Disgust | Contempt | Boredom | Neither Agree nor Disagree | 4 | Moderately Agree | 6 | Moderately Disagree | 2 | Strongly Disagree | 1 | Slightly Agree | 5 | Slightly Disagree | 3 | I don't know this music | 8 | Slightly Agree | 5 | 5039171 | PRIMED POP (ORIGINAL LYRICS OPERA) | Scepticism | scepticism | #N/A | #N/A | #N/A | PRIMED OPERA (ORIGINAL LYRICS FADO) | Bitterness | Bitter | 3.31 | 4.4 | 5.17 | PRIMED CUBAN SON (ORIGINAL LYRICS HEAVY METAL) | Thrill | Thrill | 8.05 | 8.02 | 6.54 | PRIMED SAMBA (ORIGINAL LYRICS HIP HOP) | Fear | fear | 2.76 | 6.96 | 3.22 | PRIMED KOTO (ORIGINAL LYRICS BOLERO) | Sadness | sadness | 2.21 | 5.21 | 2.82 | PRIMED HEAVY METAL (ORIGINAL LYRICS KOTO) | Wrath | Wrath | 3.47 | 5.6 | 4.23 | PRIMED FADO (ORIGINAL LYRICS POP) | Excitement | excitement | 7.5 | 7.67 | 6.18 | PRIMED HIP HOP (ORIGINAL LYRICS SAMBA) | Empathy | empathy | 5.32 | 4.81 | 4.33 |  |  |
| SYDNEY | z5041329 |  | like a bird | I can image im in a relaxing place | I can image their enthuasiastic | luxury | crazy | for first one, I fell that am follow in the rhythm and high | feel relaxing | sense of belonging | Slightly Agree | 5 | Strongly Disagree | 1 | Neither Agree nor Disagree | 4 | I don't know this music | 8 | Strongly Agree | 7 | Strongly Agree | 7 | Strongly Agree | 7 | I don't know this music | 8 | 5041329 | PRIMED POP (ORIGINAL LYRICS OPERA) | sunshine | sunshine | 7.61 | 5.56 | 5.6 | PRIMED OPERA (ORIGINAL LYRICS FADO) | boyfriend | boyfriend | 5.74 | 5.73 | 4.67 | PRIMED CUBAN SON (ORIGINAL LYRICS HEAVY METAL) | future | future | 6.71 | 6.48 | 4.89 | PRIMED SAMBA (ORIGINAL LYRICS HIP HOP) | him | him | #N/A | #N/A | #N/A | PRIMED KOTO (ORIGINAL LYRICS BOLERO) | poor | poor | 2.28 | 5.21 | 2.68 | PRIMED HEAVY METAL (ORIGINAL LYRICS KOTO) | nothing | nothing | #N/A | #N/A | #N/A | PRIMED FADO (ORIGINAL LYRICS POP) | freedom | freedom | 7.58 | 5.52 | 6.76 | PRIMED HIP HOP (ORIGINAL LYRICS SAMBA) | sorry | sorry | 3.62 | 5.11 | 3.82 |  |  |
| SYDNEY | 5042629 |  | relaxed | gentle | relaxed | serious | anxious | noisy | anxious | spirited | Neither Agree nor Disagree | 4 | Strongly Disagree | 1 | Neither Agree nor Disagree | 4 | Moderately Disagree | 2 | Neither Agree nor Disagree | 4 | Slightly Agree | 5 | I don't know this music | 8 | I don't know this music | 8 | 5042629 | UNPRIMED (ORIGINAL LYRICS OPERA) | ridiculous | ridiculous | 4.93 | 5.12 | 5.03 | UNPRIMED (ORIGINAL LYRICS FADO) | sad | sad | 1.61 | 4.13 | 3.45 | UNPRIMED (ORIGINAL LYRICS HEAVY METAL) | crazy | crazy | 5.93 | 6.28 | 5.13 | UNPRIMED (ORIGINAL LYRICS HIP HOP) | poor | poor | 2.28 | 5.21 | 2.68 | UNPRIMED (ORIGINAL LYRICS BOLERO) | | #N/A | #N/A | #N/A | #N/A | UNPRIMED (ORIGINAL LYRICS BOLERO) | beautiful | beautiful | 7.6 | 6.17 | 6.29 | UNPRIMED (ORIGINAL LYRICS POP) | worried | worried | #N/A | #N/A | #N/A | UNPRIMED (ORIGINAL LYRICS SAMBA) | | #N/A | #N/A | #N/A | #N/A |  |  |
| SYDNEY | z5046509 |  | happy | nostalgic | happy | relaxed | scary | motivation | determined | bored | Slightly Disagree | 3 | Strongly Disagree | 1 | Slightly Agree | 5 | Slightly Agree | 5 | Slightly Disagree | 3 | Slightly Disagree | 3 | Slightly Agree | 5 | Slightly Disagree | 3 | 5046509 |  |  | #N/A | #N/A | #N/A | #N/A | PRIMED FADO (ORIGINAL LYRICS FADO) | sadness | sadness | 2.21 | 5.21 | 2.82 | PRIMED HEAVY METAL (ORIGINAL LYRICS HEAVY METAL) | anger | anger | 2.34 | 7.63 | 5.5 | PRIMED HIP HOP (ORIGINAL LYRICS HIP HOP) | sadness and anger | sad | 1.61 | 4.13 | 3.45 | PRIMED BOLERO (ORIGINAL LYRICS BOLERO) | hearthache | heartbreak | 1.93 | 5.8 | 3.11 | PRIMED KOTO (ORIGINAL LYRICS KOTO) | none | none | #N/A | #N/A | #N/A | PRIMED POP (ORIGINAL LYRICS POP) | none | none | #N/A | #N/A | #N/A | PRIMED SAMBA (ORIGINAL LYRICS SAMBA) | melancholy | melancholy | 3.82 | 3.6 | 4.65 |  |  |
| SYDNEY | 5055993 |  | delight | sad | relax | lonely | Noisy | excited | lively | peaceful | Moderately Disagree | 2 | Strongly Disagree | 1 | Strongly Agree | 7 | Slightly Agree | 5 | Neither Agree nor Disagree | 4 | Moderately Agree | 6 | Slightly Disagree | 3 | I don't know this music | 8 | 5055993 | PRIMED POP (ORIGINAL LYRICS OPERA) | confidence | Confidence | 7.04 | 5.52 | 6.42 | PRIMED OPERA (ORIGINAL LYRICS FADO) | patriotic | patriotic | 6.62 | 5.54 | 6.35 | PRIMED CUBAN SON (ORIGINAL LYRICS HEAVY METAL) | toughness | toughness | #N/A | #N/A | #N/A | PRIMED SAMBA (ORIGINAL LYRICS HIP HOP) | upset | upset | 2 | 5.86 | 4.08 | PRIMED KOTO (ORIGINAL LYRICS BOLERO) | heart－broken | heartbreak | 1.93 | 5.8 | 3.11 | PRIMED HEAVY METAL (ORIGINAL LYRICS KOTO) | freedom | freedom | 7.58 | 5.52 | 6.76 | PRIMED FADO (ORIGINAL LYRICS POP) | rebellious | rebellious | 4.86 | 5.82 | 6.28 | PRIMED HIP HOP (ORIGINAL LYRICS SAMBA) | love struggle | heartbreak | 1.93 | 5.8 | 3.11 |  |  |
| SYDNEY | z5056121 |  | light | soothing | relaxed | exotic | passionate | rhythmed | young | formal | Moderately Disagree | 2 | Slightly Disagree | 3 | Strongly Agree | 7 | Strongly Agree | 7 | I don't know this music | 8 | Neither Agree nor Disagree | 4 | I don't know this music | 8 | I don't know this music | 8 | 5056121 | UNPRIMED (ORIGINAL LYRICS OPERA) | happy | happy | 8.21 | 6.49 | 6.63 | UNPRIMED (ORIGINAL LYRICS FADO) | sad | sad | 1.61 | 4.13 | 3.45 | UNPRIMED (ORIGINAL LYRICS HEAVY METAL) | heavy | Heavy | 3.69 | 4.58 | 4.1 | UNPRIMED (ORIGINAL LYRICS HIP HOP) | regretful | regretful | 2.28 | 5.74 | 3.43 | UNPRIMED (ORIGINAL LYRICS BOLERO) | desperate | desperate | 2.77 | 5.03 | 3.51 | UNPRIMED (ORIGINAL LYRICS BOLERO) | saintly | saintly | #N/A | #N/A | #N/A | UNPRIMED (ORIGINAL LYRICS POP) | rebellious | rebellious | 4.86 | 5.82 | 6.28 | UNPRIMED (ORIGINAL LYRICS SAMBA) | desired | desire | 7.69 | 7.35 | 6.49 |  |  |
| SYDNEY | z5057300 |  | Hawaii vocation. The second one make me feel in India market. | Love romantic movie music | Samba dancing | Traditional singing in some special event. | Heavy, like game music. | American style music | Korea pop music | Swan Lake | Slightly Disagree | 3 | Moderately Disagree | 2 | Neither Agree nor Disagree | 4 | Slightly Disagree | 3 | Slightly Agree | 5 | Moderately Agree | 6 | Strongly Agree | 7 | Moderately Agree | 6 | 5057300 |  |  | #N/A | #N/A | #N/A | #N/A |  |  | #N/A | #N/A | #N/A | #N/A |  |  | #N/A | #N/A | #N/A | #N/A |  |  | #N/A | #N/A | #N/A | #N/A |  |  | #N/A | #N/A | #N/A | #N/A |  |  | #N/A | #N/A | #N/A | #N/A |  |  | #N/A | #N/A | #N/A | #N/A |  |  | #N/A | #N/A | #N/A | #N/A |  |  |
| SYDNEY | z5057300 |  |  |  |  |  |  |  |  |  |  |  |  |  |  |  |  |  |  |  |  |  |  |  |  |  | 5057300 | PRIMED OPERA (ORIGINAL LYRICS OPERA) | lost | lost | 2.82 | 5.82 | 2.86 | PRIMED FADO (ORIGINAL LYRICS FADO) | opera | opera | 4.84 | 3.97 | 5.07 | PRIMED HEAVY METAL (ORIGINAL LYRICS HEAVY METAL) | horrible | horrible | 2.28 | 5.75 | 3.04 | PRIMED HIP HOP (ORIGINAL LYRICS HIP HOP) | lost | lost | 2.82 | 5.82 | 2.86 | PRIMED BOLERO (ORIGINAL LYRICS BOLERO) | love triangle | love triangle | #N/A | #N/A | #N/A | PRIMED KOTO (ORIGINAL LYRICS KOTO) | holy | holy | 6.36 | 5.36 | 4.68 | PRIMED POP (ORIGINAL LYRICS POP) | hot | hot | 5.41 | 5.31 | 5.31 | PRIMED SAMBA (ORIGINAL LYRICS SAMBA) | love | love | 8.72 | 6.44 | 7.11 |  |  |
| SYDNEY | z5057309 |  | relax | little sad | relax | relax | exciting | exciting | happy | fantastic | Moderately Disagree | 2 | Slightly Agree | 5 | Strongly Agree | 7 | Moderately Disagree | 2 | Moderately Disagree | 2 | Moderately Disagree | 2 | Moderately Disagree | 2 | Moderately Disagree | 2 | 5057309 | PRIMED POP (ORIGINAL LYRICS OPERA) | happy | happy | 8.21 | 6.49 | 6.63 | PRIMED OPERA (ORIGINAL LYRICS FADO) | sad | sad | 1.61 | 4.13 | 3.45 | PRIMED CUBAN SON (ORIGINAL LYRICS HEAVY METAL) | sad | sad | 1.61 | 4.13 | 3.45 | PRIMED SAMBA (ORIGINAL LYRICS HIP HOP) | sad | sad | 1.61 | 4.13 | 3.45 | PRIMED KOTO (ORIGINAL LYRICS BOLERO) | sad | sad | 1.61 | 4.13 | 3.45 | PRIMED HEAVY METAL (ORIGINAL LYRICS KOTO) | mad | mad | 2.44 | 6.76 | 5.86 | PRIMED FADO (ORIGINAL LYRICS POP) | mad | mad | 2.44 | 6.76 | 5.86 | PRIMED HIP HOP (ORIGINAL LYRICS SAMBA) | sad | sad | 1.61 | 4.13 | 3.45 |  |  |
| SYDNEY | z5057309 |  |  |  |  |  |  |  |  |  |  |  |  |  |  |  |  |  |  |  |  |  |  |  |  |  | 5057309 |  |  | #N/A | #N/A | #N/A | #N/A |  |  | #N/A | #N/A | #N/A | #N/A |  |  | #N/A | #N/A | #N/A | #N/A |  |  | #N/A | #N/A | #N/A | #N/A |  |  | #N/A | #N/A | #N/A | #N/A |  |  | #N/A | #N/A | #N/A | #N/A |  |  | #N/A | #N/A | #N/A | #N/A |  |  | #N/A | #N/A | #N/A | #N/A |  |  |
| SYDNEY | z5057309 |  | happy | relax | happy | relax | exciting | exciting | exciting | joy | Moderately Disagree | 2 | Slightly Agree | 5 | Strongly Agree | 7 | Slightly Agree | 5 | Strongly Disagree | 1 | Strongly Disagree | 1 | Strongly Disagree | 1 | Strongly Disagree | 1 | 5057309 |  |  | #N/A | #N/A | #N/A | #N/A |  |  | #N/A | #N/A | #N/A | #N/A |  |  | #N/A | #N/A | #N/A | #N/A |  |  | #N/A | #N/A | #N/A | #N/A |  |  | #N/A | #N/A | #N/A | #N/A |  |  | #N/A | #N/A | #N/A | #N/A |  |  | #N/A | #N/A | #N/A | #N/A |  |  | #N/A | #N/A | #N/A | #N/A |  |  |
| SYDNEY | z5057358 |  | cheerful | sadness | noisy | like chanting | heavy rhythm | strange | quite loudly | immersive | I don't know this music | 8 | Moderately Disagree | 2 | Moderately Disagree | 2 | Neither Agree nor Disagree | 4 | I don't know this music | 8 | Strongly Disagree | 1 | I don't know this music | 8 | I don't know this music | 8 | 5057358 | UNPRIMED (ORIGINAL LYRICS OPERA) | confident | confident | 7.98 | 6.22 | 7.68 | UNPRIMED (ORIGINAL LYRICS FADO) | strange | strange | 4.79 | 5.09 | 5.25 | UNPRIMED (ORIGINAL LYRICS HEAVY METAL) | terrible | terrible | 1.93 | 6.27 | 3.58 | UNPRIMED (ORIGINAL LYRICS HIP HOP) | powerful | Powerful | 6.84 | 5.83 | 7.19 | UNPRIMED (ORIGINAL LYRICS BOLERO) | Negative Energy | Negative Energy | #N/A | #N/A | #N/A | UNPRIMED (ORIGINAL LYRICS BOLERO) | Solemnly | solemn | 4.32 | 3.56 | 4.61 | UNPRIMED (ORIGINAL LYRICS POP) | disgusting | disgusting | 2.96 | 5.18 | 3.64 | UNPRIMED (ORIGINAL LYRICS SAMBA) | negative | negative | #N/A | #N/A | #N/A |  |  |
| SYDNEY | z5057415 |  | annoyed | relaxed | humour | peaceful | funny | angery | happy | enlightening | Moderately Disagree | 2 | Slightly Agree | 5 | Slightly Agree | 5 | Neither Agree nor Disagree | 4 | Strongly Agree | 7 | Moderately Agree | 6 | Moderately Disagree | 2 | Strongly Disagree | 1 | 5057415 | PRIMED POP (ORIGINAL LYRICS OPERA) | ridiculous | ridiculous | 4.93 | 5.12 | 5.03 | PRIMED OPERA (ORIGINAL LYRICS FADO) | angery | anger | 2.34 | 7.63 | 5.5 | PRIMED CUBAN SON (ORIGINAL LYRICS HEAVY METAL) | horrible | horrible | 2.28 | 5.75 | 3.04 | PRIMED SAMBA (ORIGINAL LYRICS HIP HOP) | sad | sad | 1.61 | 4.13 | 3.45 | PRIMED KOTO (ORIGINAL LYRICS BOLERO) | sad | sad | 1.61 | 4.13 | 3.45 | PRIMED HEAVY METAL (ORIGINAL LYRICS KOTO) | sorrowful | sorrow | 2.32 | 4.48 | 3.67 | PRIMED FADO (ORIGINAL LYRICS POP) | satiric | satiric | #N/A | #N/A | #N/A | PRIMED HIP HOP (ORIGINAL LYRICS SAMBA) | fearful | Fearful | 2.25 | 6.33 | 3.64 |  |  |
| SYDNEY | z5057415 |  | happy | upset | happy | peaceful | angry | angry | relaxed | calm | Slightly Agree | 5 | Slightly Agree | 5 | Moderately Agree | 6 | Moderately Agree | 6 | Strongly Agree | 7 | Strongly Agree | 7 | Neither Agree nor Disagree | 4 | Neither Agree nor Disagree | 4 | 5057415 |  |  | #N/A | #N/A | #N/A | #N/A |  |  | #N/A | #N/A | #N/A | #N/A |  |  | #N/A | #N/A | #N/A | #N/A |  |  | #N/A | #N/A | #N/A | #N/A |  |  | #N/A | #N/A | #N/A | #N/A |  |  | #N/A | #N/A | #N/A | #N/A |  |  | #N/A | #N/A | #N/A | #N/A |  |  | #N/A | #N/A | #N/A | #N/A |  |  |
| SYDNEY | z5059949 |  | Happiness/Dance | Romantic | Exotic | Disciplined | Annoyed | gangsta | Happy/Energetic | Drama | Slightly Agree | 5 | Strongly Disagree | 1 | Strongly Agree | 7 | Moderately Agree | 6 | Strongly Disagree | 1 | Slightly Disagree | 3 | Slightly Disagree | 3 | Slightly Agree | 5 | 5059949 | PRIMED POP (ORIGINAL LYRICS OPERA) | judged | judged | #N/A | #N/A | #N/A | PRIMED OPERA (ORIGINAL LYRICS FADO) | pain | pain | 2.13 | 6.5 | 3.71 | PRIMED CUBAN SON (ORIGINAL LYRICS HEAVY METAL) | Guilt | Guilt | 2.14 | 5.36 | 2.96 | PRIMED SAMBA (ORIGINAL LYRICS HIP HOP) | sad | sad | 1.61 | 4.13 | 3.45 | PRIMED KOTO (ORIGINAL LYRICS BOLERO) | sad | sad | 1.61 | 4.13 | 3.45 | PRIMED HEAVY METAL (ORIGINAL LYRICS KOTO) | cleansing | cleansing | #N/A | #N/A | #N/A | PRIMED FADO (ORIGINAL LYRICS POP) | scared | scared | 2.78 | 6.82 | 2.94 | PRIMED HIP HOP (ORIGINAL LYRICS SAMBA) | heartbreak | heartbreak | 1.93 | 5.8 | 3.11 |  |  |
| SYDNEY | 5060014 |  | happy | relaxed | Groovy | Curious | confused | confused | nostalgic | relaxed | Moderately Disagree | 2 | Moderately Disagree | 2 | Moderately Agree | 6 | Slightly Disagree | 3 | Slightly Agree | 5 | Moderately Agree | 6 | Moderately Disagree | 2 | Moderately Disagree | 2 | 5060014 | UNPRIMED (ORIGINAL LYRICS OPERA) | disgust | disgusting | 2.96 | 5.18 | 3.64 | UNPRIMED (ORIGINAL LYRICS FADO) | uncertain | uncertain | 3.45 | 4.79 | 3.37 | UNPRIMED (ORIGINAL LYRICS HEAVY METAL) | empowered | power | 6.54 | 6.67 | 7.28 | UNPRIMED (ORIGINAL LYRICS HIP HOP) | confused | confused | 3.21 | 6.03 | 4.24 | UNPRIMED (ORIGINAL LYRICS BOLERO) | Sadness | sadness | 2.21 | 5.21 | 2.82 | UNPRIMED (ORIGINAL LYRICS BOLERO) | Confused | confused | 3.21 | 6.03 | 4.24 | UNPRIMED (ORIGINAL LYRICS POP) | angry | angry | 2.85 | 7.17 | 5.55 | UNPRIMED (ORIGINAL LYRICS SAMBA) | Sympathy | Sympathy | 5.33 | 5.03 | 4.73 |  |  |
| SYDNEY | z5060015 |  | delighted | confident | sleepy | bored, relaxed, meditated | uncomfortable, surprised | at ease | playful | pleased | Slightly Agree | 5 | Strongly Disagree | 1 | Strongly Agree | 7 | Slightly Agree | 5 | Neither Agree nor Disagree | 4 | Strongly Agree | 7 | Neither Agree nor Disagree | 4 | Neither Agree nor Disagree | 4 | 5060015 | PRIMED OPERA (ORIGINAL LYRICS OPERA) | content | content | #N/A | #N/A | #N/A | PRIMED FADO (ORIGINAL LYRICS FADO) | powerless | powerless | #N/A | #N/A | #N/A | PRIMED HEAVY METAL (ORIGINAL LYRICS HEAVY METAL) | furious | furious | 1.96 | 7.64 | 5.32 | PRIMED HIP HOP (ORIGINAL LYRICS HIP HOP) | hurtful | hurt | 1.9 | 5.85 | 3.33 | PRIMED BOLERO (ORIGINAL LYRICS BOLERO) | hatred | Hatred | 1.98 | 6.66 | 4.3 | PRIMED KOTO (ORIGINAL LYRICS KOTO) | content | content | #N/A | #N/A | #N/A | PRIMED POP (ORIGINAL LYRICS POP) | confused | confused | 3.21 | 6.03 | 4.24 | PRIMED SAMBA (ORIGINAL LYRICS SAMBA) | lonely | lonely | 2.17 | 4.51 | 2.95 |  |  |
| SYDNEY | z5060340 |  | Love | Soft music | Relaxing | Relaxing | cool | Youth | fun | relaxing | I don't know this music | 8 | Slightly Disagree | 3 | Neither Agree nor Disagree | 4 | I don't know this music | 8 | I don't know this music | 8 | I don't know this music | 8 | I don't know this music | 8 | I don't know this music | 8 | 5060340 | UNPRIMED (ORIGINAL LYRICS OPERA) | happy | happy | 8.21 | 6.49 | 6.63 | UNPRIMED (ORIGINAL LYRICS FADO) | sad | sad | 1.61 | 4.13 | 3.45 | UNPRIMED (ORIGINAL LYRICS HEAVY METAL) | empowered | power | 6.54 | 6.67 | 7.28 | UNPRIMED (ORIGINAL LYRICS HIP HOP) | sad | sad | 1.61 | 4.13 | 3.45 | UNPRIMED (ORIGINAL LYRICS BOLERO) | sad | sad | 1.61 | 4.13 | 3.45 | UNPRIMED (ORIGINAL LYRICS BOLERO) | sad | sad | 1.61 | 4.13 | 3.45 | UNPRIMED (ORIGINAL LYRICS POP) | Anger | anger | 2.34 | 7.63 | 5.5 | UNPRIMED (ORIGINAL LYRICS SAMBA) | Sad | sad | 1.61 | 4.13 | 3.45 |  |  |
| SYDNEY | z5060369 |  | excited | sadness | energetic | meditative | energetic | chilled | happy | calm | Slightly Agree | 5 | Slightly Agree | 5 | Slightly Disagree | 3 | Moderately Disagree | 2 | Moderately Disagree | 2 | Strongly Agree | 7 | Slightly Agree | 5 | Slightly Agree | 5 | 5060369 | uncomfortable | uncomfortable | Uncomfortable | 2.97 | 6.06 | 3.42 | PRIMED FADO (ORIGINAL LYRICS FADO) | sad | sad | 1.61 | 4.13 | 3.45 | PRIMED HEAVY METAL (ORIGINAL LYRICS HEAVY METAL) | uncomfortable | Uncomfortable | 2.97 | 6.06 | 3.42 | PRIMED HIP HOP (ORIGINAL LYRICS HIP HOP) | sad | sad | 1.61 | 4.13 | 3.45 | PRIMED BOLERO (ORIGINAL LYRICS BOLERO) | heartbroken | heartbreak | 1.93 | 5.8 | 3.11 | PRIMED KOTO (ORIGINAL LYRICS KOTO) | enlightened | enlightened | #N/A | #N/A | #N/A | PRIMED POP (ORIGINAL LYRICS POP) | confused | confused | 3.21 | 6.03 | 4.24 | PRIMED SAMBA (ORIGINAL LYRICS SAMBA) | sad | sad | 1.61 | 4.13 | 3.45 |  |  |
| SYDNEY | z5060369 |  | energetic | love | excited | intrigued | weird | uncomfortable | happy | calm | Slightly Agree | 5 | Slightly Agree | 5 | Slightly Disagree | 3 | Slightly Disagree | 3 | Moderately Disagree | 2 | Strongly Agree | 7 | Slightly Agree | 5 | Slightly Agree | 5 | 5060369 |  |  | #N/A | #N/A | #N/A | #N/A |  |  | #N/A | #N/A | #N/A | #N/A |  |  | #N/A | #N/A | #N/A | #N/A |  |  | #N/A | #N/A | #N/A | #N/A |  |  | #N/A | #N/A | #N/A | #N/A |  |  | #N/A | #N/A | #N/A | #N/A |  |  | #N/A | #N/A | #N/A | #N/A |  |  | #N/A | #N/A | #N/A | #N/A |  |  |
| SYDNEY | 5060478 |  | alertness | calmness | alertness | tense | annoyance | tension | Happiness | Clamness | Moderately Agree | 6 | Strongly Disagree | 1 | Slightly Agree | 5 | Slightly Agree | 5 | Slightly Agree | 5 | Moderately Agree | 6 | Slightly Agree | 5 | Slightly Agree | 5 | 5060478 |  |  | #N/A | #N/A | #N/A | #N/A |  |  | #N/A | #N/A | #N/A | #N/A |  |  | #N/A | #N/A | #N/A | #N/A |  |  | #N/A | #N/A | #N/A | #N/A |  |  | #N/A | #N/A | #N/A | #N/A |  |  | #N/A | #N/A | #N/A | #N/A |  |  | #N/A | #N/A | #N/A | #N/A |  |  | #N/A | #N/A | #N/A | #N/A |  |  |
| SYDNEY | z5060526 |  | excited | relaxed | dancelike | relaxed | angry | calm, not excited | excited, energized | excited | Moderately Agree | 6 | Moderately Disagree | 2 | Strongly Agree | 7 | Slightly Disagree | 3 | Neither Agree nor Disagree | 4 | Strongly Agree | 7 | Slightly Agree | 5 | Moderately Agree | 6 | 5060526 | PRIMED OPERA (ORIGINAL LYRICS OPERA) | confidence | Confidence | 7.04 | 5.52 | 6.42 | PRIMED FADO (ORIGINAL LYRICS FADO) | sadness | sadness | 2.21 | 5.21 | 2.82 | PRIMED HEAVY METAL (ORIGINAL LYRICS HEAVY METAL) | annoyance | annoyance | 2.97 | 5.18 | 4.21 | PRIMED HIP HOP (ORIGINAL LYRICS HIP HOP) | sadness | sadness | 2.21 | 5.21 | 2.82 | PRIMED BOLERO (ORIGINAL LYRICS BOLERO) | happiness | happiness | 7.96 | 6.56 | 6.7 | PRIMED KOTO (ORIGINAL LYRICS KOTO) | sadness | sadness | 2.21 | 5.21 | 2.82 | PRIMED POP (ORIGINAL LYRICS POP) | laughter | laughter | 8.45 | 6.75 | 6.45 | PRIMED SAMBA (ORIGINAL LYRICS SAMBA) | sadness | sadness | 2.21 | 5.21 | 2.82 |  |  |
| SYDNEY | 5060814 |  | Energetic | Peaceful | Groovy | Transcendental | Disgust | Indifference | Bored | Satisfied | Neither Agree nor Disagree | 4 | Strongly Disagree | 1 | Strongly Disagree | 1 | Strongly Disagree | 1 | Neither Agree nor Disagree | 4 | Strongly Agree | 7 | Neither Agree nor Disagree | 4 | Neither Agree nor Disagree | 4 | 5060814 | PRIMED OPERA (ORIGINAL LYRICS OPERA) | mysterious | mysterious | #N/A | #N/A | #N/A | PRIMED FADO (ORIGINAL LYRICS FADO) | confused | confused | 3.21 | 6.03 | 4.24 | PRIMED HEAVY METAL (ORIGINAL LYRICS HEAVY METAL) | selfish | selfish | 2.42 | 5.5 | 4.64 | PRIMED HIP HOP (ORIGINAL LYRICS HIP HOP) | anxious | Anxious | 4.81 | 6.92 | 5.33 | PRIMED BOLERO (ORIGINAL LYRICS BOLERO) | Confusion | confusion | 3.46 | 6.07 | 3.04 | PRIMED KOTO (ORIGINAL LYRICS KOTO) | philosophical | philosophical | #N/A | #N/A | #N/A | PRIMED POP (ORIGINAL LYRICS POP) | confused | confused | 3.21 | 6.03 | 4.24 | PRIMED SAMBA (ORIGINAL LYRICS SAMBA) | Sad | sad | 1.61 | 4.13 | 3.45 |  |  |
| SYDNEY | z5060869 |  | Fiesta | Romance | Romance | Travel | Anger | Confidence | pleasure | Awe | Strongly Disagree | 1 | Slightly Agree | 5 | Moderately Disagree | 2 | Neither Agree nor Disagree | 4 | Strongly Disagree | 1 | Slightly Agree | 5 | Strongly Disagree | 1 | Strongly Disagree | 1 | 5060869 | UNPRIMED (ORIGINAL LYRICS OPERA) | Lust | lust | 7.12 | 6.88 | 5.49 | UNPRIMED (ORIGINAL LYRICS FADO) | Indifference | indifference | #N/A | #N/A | #N/A | UNPRIMED (ORIGINAL LYRICS HEAVY METAL) | Power | Power | 6.54 | 6.67 | 7.28 | UNPRIMED (ORIGINAL LYRICS HIP HOP) | Confusion | confusion | 3.46 | 6.07 | 3.04 | UNPRIMED (ORIGINAL LYRICS BOLERO) | Sadness | sadness | 2.21 | 5.21 | 2.82 | UNPRIMED (ORIGINAL LYRICS BOLERO) | Funny | Funny | 8.56 | 7 | 6.15 | UNPRIMED (ORIGINAL LYRICS POP) | Funny | Funny | 8.56 | 7 | 6.15 | UNPRIMED (ORIGINAL LYRICS SAMBA) | Tragedy | tragedy | 1.78 | 6.24 | 3.5 |  |  |
| SYDNEY | 5060869 |  | Romance | Exoticism | Tragedy | Seriousness | Anger | Confidence | Fun | Superiority | Strongly Disagree | 1 | Slightly Agree | 5 | Slightly Disagree | 3 | Moderately Disagree | 2 | Strongly Disagree | 1 | Slightly Agree | 5 | Strongly Disagree | 1 | Strongly Disagree | 1 | 5060869 | PRIMED OPERA (ORIGINAL LYRICS OPERA) | Pride | Pride | 7 | 5.83 | 7.06 | PRIMED FADO (ORIGINAL LYRICS FADO) | Sadness | sadness | 2.21 | 5.21 | 2.82 | PRIMED HEAVY METAL (ORIGINAL LYRICS HEAVY METAL) | Hope | Hope | 7.05 | 5.44 | 5.52 | PRIMED HIP HOP (ORIGINAL LYRICS HIP HOP) | Betrayal | Betrayal | 2.28 | 5.37 | 4.18 | PRIMED BOLERO (ORIGINAL LYRICS BOLERO) | Sadness | sadness | 2.21 | 5.21 | 2.82 | PRIMED KOTO (ORIGINAL LYRICS KOTO) | Piety | Piety | #N/A | #N/A | #N/A | PRIMED POP (ORIGINAL LYRICS POP) | Rebellion | rebellious | 4.86 | 5.82 | 6.28 | PRIMED SAMBA (ORIGINAL LYRICS SAMBA) | romantic | Romantic | 8.32 | 7.59 | 6.08 |  |  |
| SYDNEY | 5060895 |  | Latino | Fado | Cuban | oriental | Thrash Metal | South London, American rap | pop/electronic | orchestra | Moderately Agree | 6 | I don't know this music | 8 | Slightly Agree | 5 | Slightly Agree | 5 | Moderately Agree | 6 | Slightly Agree | 5 | Neither Agree nor Disagree | 4 | Strongly Agree | 7 | 5060895 |  |  | #N/A | #N/A | #N/A | #N/A |  |  | #N/A | #N/A | #N/A | #N/A |  |  | #N/A | #N/A | #N/A | #N/A |  |  | #N/A | #N/A | #N/A | #N/A |  |  | #N/A | #N/A | #N/A | #N/A |  |  | #N/A | #N/A | #N/A | #N/A |  |  | #N/A | #N/A | #N/A | #N/A |  |  | #N/A | #N/A | #N/A | #N/A |  |  |
| SYDNEY | z5060991 |  | Happy | Beautiful | Sassy | meditating | Angry | Cool | Average | Concentrating | Slightly Agree | 5 | Slightly Disagree | 3 | Moderately Agree | 6 | Moderately Agree | 6 | Moderately Disagree | 2 | Neither Agree nor Disagree | 4 | Slightly Agree | 5 | Slightly Agree | 5 | 5060991 | PRIMED OPERA (ORIGINAL LYRICS OPERA) | self-centred | self-centred | #N/A | #N/A | #N/A | PRIMED FADO (ORIGINAL LYRICS FADO) | sorrow | sorrow | 2.32 | 4.48 | 3.67 | PRIMED HEAVY METAL (ORIGINAL LYRICS HEAVY METAL) | satire | satire | 6.23 | 5.34 | 5.62 | PRIMED HIP HOP (ORIGINAL LYRICS HIP HOP) | serious | serious | 5.08 | 4 | 5.12 | PRIMED BOLERO (ORIGINAL LYRICS BOLERO) | love | love | 8.72 | 6.44 | 7.11 | PRIMED KOTO (ORIGINAL LYRICS KOTO) | supersticious | supersticious | #N/A | #N/A | #N/A | PRIMED POP (ORIGINAL LYRICS POP) | humour | humour | #N/A | #N/A | #N/A | PRIMED SAMBA (ORIGINAL LYRICS SAMBA) | Beware | Beware | 3.52 | 6.38 | 3.69 |  |  |
| SYDNEY | z5060991 |  |  |  |  |  |  |  |  |  |  |  |  |  |  |  |  |  |  |  |  |  |  |  |  |  | 5060991 | UNPRIMED (ORIGINAL LYRICS OPERA) | scared | scared | 2.78 | 6.82 | 2.94 | UNPRIMED (ORIGINAL LYRICS FADO) | confusion | confusion | 3.46 | 6.07 | 3.04 | UNPRIMED (ORIGINAL LYRICS HEAVY METAL) | creepy | creepy | 2.71 | 5.49 | 3.95 | UNPRIMED (ORIGINAL LYRICS HIP HOP) | Sadness | sadness | 2.21 | 5.21 | 2.82 | UNPRIMED (ORIGINAL LYRICS BOLERO) | | #N/A | #N/A | #N/A | #N/A | UNPRIMED (ORIGINAL LYRICS BOLERO) | creepy | creepy | 2.71 | 5.49 | 3.95 | UNPRIMED (ORIGINAL LYRICS POP) | anger | anger | 2.34 | 7.63 | 5.5 | UNPRIMED (ORIGINAL LYRICS SAMBA) | | #N/A | #N/A | #N/A | #N/A |  |  |
| SYDNEY | z5061280 |  | joy | love | calm | anxiety | anger | pride | joy | relaxed | Strongly Disagree | 1 | Strongly Disagree | 1 | Slightly Agree | 5 | Strongly Agree | 7 | Strongly Disagree | 1 | Strongly Disagree | 1 | Strongly Disagree | 1 | Strongly Disagree | 1 | 5061280 | UNPRIMED (ORIGINAL LYRICS OPERA) | Vanity | Vanity | 4.3 | 4.98 | 4.8 | UNPRIMED (ORIGINAL LYRICS FADO) | Sad | sad | 1.61 | 4.13 | 3.45 | UNPRIMED (ORIGINAL LYRICS HEAVY METAL) | Determined | Determined | #N/A | #N/A | #N/A | UNPRIMED (ORIGINAL LYRICS HIP HOP) | Frustrated | Frustrated | 2.48 | 5.61 | 3.5 | UNPRIMED (ORIGINAL LYRICS BOLERO) | | #N/A | #N/A | #N/A | #N/A | UNPRIMED (ORIGINAL LYRICS BOLERO) | Fanatical | Fanatical | #N/A | #N/A | #N/A | UNPRIMED (ORIGINAL LYRICS POP) | Angry | angry | 2.85 | 7.17 | 5.55 | UNPRIMED (ORIGINAL LYRICS SAMBA) | | #N/A | #N/A | #N/A | #N/A |  |  |
| SYDNEY | 5061290 |  | Familial | Serenading - seeking love | Makes me want to do a slow - midtempo dance. Feels like it tells a story | Very strict and composed. Feels very serious | Enthusiastic and determined, pumped up | Confidence with a sense of vanity | Short term love | Triumphant and excited | Moderately Agree | 6 | Slightly Disagree | 3 | Slightly Agree | 5 | Moderately Agree | 6 | Slightly Disagree | 3 | Moderately Disagree | 2 | Slightly Agree | 5 | Slightly Agree | 5 | 5061290 | PRIMED OPERA (ORIGINAL LYRICS OPERA) | pride | Pride | 7 | 5.83 | 7.06 | PRIMED FADO (ORIGINAL LYRICS FADO) | anguish | Anguish | #N/A | #N/A | #N/A | PRIMED HEAVY METAL (ORIGINAL LYRICS HEAVY METAL) | anger | anger | 2.34 | 7.63 | 5.5 | PRIMED HIP HOP (ORIGINAL LYRICS HIP HOP) | betrayal | Betrayal | 2.28 | 5.37 | 4.18 | PRIMED BOLERO (ORIGINAL LYRICS BOLERO) | Betrayal | Betrayal | 2.28 | 5.37 | 4.18 | PRIMED KOTO (ORIGINAL LYRICS KOTO) | wisdom | wisdom | 7.34 | 5.25 | 6.61 | PRIMED POP (ORIGINAL LYRICS POP) | confusion | confusion | 3.46 | 6.07 | 3.04 | PRIMED SAMBA (ORIGINAL LYRICS SAMBA) | Regret | Regret | 2.26 | 5.67 | 3.23 |  |  |
| SYDNEY | 5061650 |  | Dancing | Relaxation | Dance | Peaceful | Fire, anger | Power | Energy | Sleepy | Neither Agree nor Disagree | 4 | Neither Agree nor Disagree | 4 | Moderately Agree | 6 | Moderately Disagree | 2 | Moderately Disagree | 2 | Neither Agree nor Disagree | 4 | Moderately Disagree | 2 | Moderately Disagree | 2 | 5061650 | PRIMED POP (ORIGINAL LYRICS OPERA) | Judgement | Judgement | #N/A | #N/A | #N/A | PRIMED OPERA (ORIGINAL LYRICS FADO) | Neutral | Neutral | #N/A | #N/A | #N/A | PRIMED CUBAN SON (ORIGINAL LYRICS HEAVY METAL) | Morbidity | Morbidity | #N/A | #N/A | #N/A | PRIMED SAMBA (ORIGINAL LYRICS HIP HOP) | Betrayal | Betrayal | 2.28 | 5.37 | 4.18 | PRIMED KOTO (ORIGINAL LYRICS BOLERO) | Betrayal | Betrayal | 2.28 | 5.37 | 4.18 | PRIMED HEAVY METAL (ORIGINAL LYRICS KOTO) | Purity | Purity | #N/A | #N/A | #N/A | PRIMED FADO (ORIGINAL LYRICS POP) | Horrid | Horrid | #N/A | #N/A | #N/A | PRIMED HIP HOP (ORIGINAL LYRICS SAMBA) | Pain | pain | 2.13 | 6.5 | 3.71 |  |  |
| SYDNEY | 5061675 |  | Bored | Calm | Flowing | Peaceful | Pumped | Skilled | Excited | Ready | I don't know this music | 8 | Moderately Disagree | 2 | Strongly Agree | 7 | Strongly Agree | 7 | Neither Agree nor Disagree | 4 | Slightly Agree | 5 | I don't know this music | 8 | I don't know this music | 8 | 5061675 |  |  | #N/A | #N/A | #N/A | #N/A |  |  | #N/A | #N/A | #N/A | #N/A |  |  | #N/A | #N/A | #N/A | #N/A |  |  | #N/A | #N/A | #N/A | #N/A |  |  | #N/A | #N/A | #N/A | #N/A |  |  | #N/A | #N/A | #N/A | #N/A |  |  | #N/A | #N/A | #N/A | #N/A |  |  | #N/A | #N/A | #N/A | #N/A |  |  |
| SYDNEY | 5061678 |  | carefree | nostalgic | playful | interested | irritated | motivated | relaxed | insightful | Slightly Agree | 5 | Strongly Disagree | 1 | I don't know this music | 8 | I don't know this music | 8 | Moderately Disagree | 2 | Slightly Agree | 5 | Neither Agree nor Disagree | 4 | Neither Agree nor Disagree | 4 | 5061678 | PRIMED OPERA (ORIGINAL LYRICS OPERA) | Curious | Curious | 6.08 | 5.82 | 5.42 | PRIMED FADO (ORIGINAL LYRICS FADO) | Poetic | Poetic | #N/A | #N/A | #N/A | PRIMED HEAVY METAL (ORIGINAL LYRICS HEAVY METAL) | Uninterested | Uninterested | #N/A | #N/A | #N/A | PRIMED HIP HOP (ORIGINAL LYRICS HIP HOP) | Skilled | Skilled | #N/A | #N/A | #N/A | PRIMED BOLERO (ORIGINAL LYRICS BOLERO) | Poetic | Poetic | #N/A | #N/A | #N/A | PRIMED KOTO (ORIGINAL LYRICS KOTO) | Interested | Interested | 6.58 | 5.42 | 5.95 | PRIMED POP (ORIGINAL LYRICS POP) | Annoyed | annoy | 2.96 | 5.52 | 4.44 | PRIMED SAMBA (ORIGINAL LYRICS SAMBA) | Sympathetic | sympathetic | 5.74 | 3.99 | 5.59 |  |  |
| SYDNEY | z5061982 |  | Sultry | Melancholy | Sensual | Meditative | Angry | Struggle | Light hearted | Intellectual | I don't know this music | 8 | Strongly Disagree | 1 | Strongly Agree | 7 | Slightly Agree | 5 | I don't know this music | 8 | Slightly Disagree | 3 | I don't know this music | 8 | I don't know this music | 8 | 5061982 | PRIMED POP (ORIGINAL LYRICS OPERA) | Judgement | Judgement | #N/A | #N/A | #N/A | PRIMED OPERA (ORIGINAL LYRICS FADO) | Spiritual | spirit | 7 | 5.56 | 5.82 | PRIMED CUBAN SON (ORIGINAL LYRICS HEAVY METAL) | Saddness | sad | 1.61 | 4.13 | 3.45 | PRIMED SAMBA (ORIGINAL LYRICS HIP HOP) | Longing | Longing | #N/A | #N/A | #N/A | PRIMED KOTO (ORIGINAL LYRICS BOLERO) | Suffering | suffer | 1.72 | 6.13 | 2.54 | PRIMED HEAVY METAL (ORIGINAL LYRICS KOTO) | Cleansing | cleansing | #N/A | #N/A | #N/A | PRIMED FADO (ORIGINAL LYRICS POP) | Anger | anger | 2.34 | 7.63 | 5.5 | PRIMED HIP HOP (ORIGINAL LYRICS SAMBA) | Fear | fear | 2.76 | 6.96 | 3.22 |  |  |
| SYDNEY | 5062194 |  |  |  |  |  |  |  |  |  |  |  |  |  |  |  |  |  |  |  |  |  |  |  |  |  | 5062194 | PRIMED POP (ORIGINAL LYRICS OPERA) | Revolt | Revolt | 4.13 | 6.56 | 6.18 | PRIMED OPERA (ORIGINAL LYRICS FADO) | Sad | sad | 1.61 | 4.13 | 3.45 | PRIMED CUBAN SON (ORIGINAL LYRICS HEAVY METAL) | Anger | anger | 2.34 | 7.63 | 5.5 | PRIMED SAMBA (ORIGINAL LYRICS HIP HOP) | Sorrow | sorrow | 2.32 | 4.48 | 3.67 | PRIMED KOTO (ORIGINAL LYRICS BOLERO) | Sad | sad | 1.61 | 4.13 | 3.45 | PRIMED HEAVY METAL (ORIGINAL LYRICS KOTO) | Confusion | confusion | 3.46 | 6.07 | 3.04 | PRIMED FADO (ORIGINAL LYRICS POP) | Anger | anger | 2.34 | 7.63 | 5.5 | PRIMED HIP HOP (ORIGINAL LYRICS SAMBA) | Sad | sad | 1.61 | 4.13 | 3.45 |  |  |
| SYDNEY | 5062194 |  | Joy | Calm | Happy | Confusion | Angry | Excited | Bored | Motivated | Slightly Agree | 5 | Moderately Disagree | 2 | Slightly Disagree | 3 | Strongly Agree | 7 | I don't know this music | 8 | Strongly Agree | 7 | I don't know this music | 8 | I don't know this music | 8 | 5062194 |  | Revulsion | Revulsion | #N/A | #N/A | #N/A |  |  | #N/A | #N/A | #N/A | #N/A |  |  | #N/A | #N/A | #N/A | #N/A |  |  | #N/A | #N/A | #N/A | #N/A |  |  | #N/A | #N/A | #N/A | #N/A |  |  | #N/A | #N/A | #N/A | #N/A |  |  | #N/A | #N/A | #N/A | #N/A |  |  | #N/A | #N/A | #N/A | #N/A |  |  |
| SYDNEY | 5062194 |  |  |  |  |  |  |  |  |  |  |  |  |  |  |  |  |  |  |  |  |  |  |  |  |  | 5062194 |  |  | #N/A | #N/A | #N/A | #N/A |  |  | #N/A | #N/A | #N/A | #N/A |  |  | #N/A | #N/A | #N/A | #N/A |  |  | #N/A | #N/A | #N/A | #N/A |  |  | #N/A | #N/A | #N/A | #N/A |  |  | #N/A | #N/A | #N/A | #N/A |  |  | #N/A | #N/A | #N/A | #N/A |  |  | #N/A | #N/A | #N/A | #N/A |  |  |
| SYDNEY | 5062194 |  | Happy | Sad | Happy | Confused | Angry | Excited | Bored | Motivated | I don't know this music | 8 | Moderately Disagree | 2 | Slightly Agree | 5 | Strongly Agree | 7 | I don't know this music | 8 | Strongly Agree | 7 | I don't know this music | 8 | I don't know this music | 8 | 5062194 |  |  | #N/A | #N/A | #N/A | #N/A |  |  | #N/A | #N/A | #N/A | #N/A |  |  | #N/A | #N/A | #N/A | #N/A |  |  | #N/A | #N/A | #N/A | #N/A |  |  | #N/A | #N/A | #N/A | #N/A |  |  | #N/A | #N/A | #N/A | #N/A |  |  | #N/A | #N/A | #N/A | #N/A |  |  | #N/A | #N/A | #N/A | #N/A |  |  |
| SYDNEY | z5062303 |  | Groovy | Lonely | Smooth | Bored | Rage | Cool | Indifferent | Calm | Moderately Agree | 6 | Neither Agree nor Disagree | 4 | Moderately Agree | 6 | Moderately Agree | 6 | Neither Agree nor Disagree | 4 | Moderately Agree | 6 | Moderately Agree | 6 | Moderately Agree | 6 | 5062303 |  |  | #N/A | #N/A | #N/A | #N/A |  |  | #N/A | #N/A | #N/A | #N/A |  |  | #N/A | #N/A | #N/A | #N/A |  |  | #N/A | #N/A | #N/A | #N/A |  |  | #N/A | #N/A | #N/A | #N/A |  |  | #N/A | #N/A | #N/A | #N/A |  |  | #N/A | #N/A | #N/A | #N/A |  |  | #N/A | #N/A | #N/A | #N/A |  |  |
| SYDNEY | z5062303 |  |  |  |  |  |  |  |  |  |  |  |  |  |  |  |  |  |  |  |  |  |  |  |  |  | 5062303 |  | Longing | Longing | #N/A | #N/A | #N/A | PRIMED FADO (ORIGINAL LYRICS FADO) | Longing | Longing | #N/A | #N/A | #N/A | PRIMED HEAVY METAL (ORIGINAL LYRICS HEAVY METAL) | Anger | anger | 2.34 | 7.63 | 5.5 | PRIMED HIP HOP (ORIGINAL LYRICS HIP HOP) | Longing | Longing | #N/A | #N/A | #N/A | PRIMED BOLERO (ORIGINAL LYRICS BOLERO) | Disdain | Disdain | #N/A | #N/A | #N/A | PRIMED KOTO (ORIGINAL LYRICS KOTO) | Hope | Hope | 7.05 | 5.44 | 5.52 | PRIMED POP (ORIGINAL LYRICS POP) | Rebellious | rebellious | 4.86 | 5.82 | 6.28 | PRIMED SAMBA (ORIGINAL LYRICS SAMBA) | Longing | Longing | #N/A | #N/A | #N/A |  |  |
| SYDNEY | z5062348 |  | Energised | Relaxed | Happy | Happy | Intense | Happy | Neutral | Tense | Moderately Agree | 6 | Moderately Disagree | 2 | Strongly Agree | 7 | Strongly Agree | 7 | Moderately Agree | 6 | Strongly Agree | 7 | Moderately Agree | 6 | Moderately Agree | 6 | 5062348 | PRIMED POP (ORIGINAL LYRICS OPERA) | Neutral | Neutral | #N/A | #N/A | #N/A | PRIMED OPERA (ORIGINAL LYRICS FADO) | Sad | sad | 1.61 | 4.13 | 3.45 | PRIMED CUBAN SON (ORIGINAL LYRICS HEAVY METAL) | Concern | Concern | #N/A | #N/A | #N/A | PRIMED SAMBA (ORIGINAL LYRICS HIP HOP) | Sorrow | sorrow | 2.32 | 4.48 | 3.67 | PRIMED KOTO (ORIGINAL LYRICS BOLERO) | Interest | interest | 6.97 | 5.66 | 5.89 | PRIMED HEAVY METAL (ORIGINAL LYRICS KOTO) | Concern | Concern | #N/A | #N/A | #N/A | PRIMED FADO (ORIGINAL LYRICS POP) | Concern | Concern | #N/A | #N/A | #N/A | PRIMED HIP HOP (ORIGINAL LYRICS SAMBA) | Relatable | Relatable | #N/A | #N/A | #N/A |  |  |
| SYDNEY | z5062348 |  |  |  |  |  |  |  |  |  |  |  |  |  |  |  |  |  |  |  |  |  |  |  |  |  | 5062348 |  |  | #N/A | #N/A | #N/A | #N/A |  |  | #N/A | #N/A | #N/A | #N/A |  |  | #N/A | #N/A | #N/A | #N/A |  |  | #N/A | #N/A | #N/A | #N/A |  |  | #N/A | #N/A | #N/A | #N/A |  |  | #N/A | #N/A | #N/A | #N/A |  |  | #N/A | #N/A | #N/A | #N/A |  |  | #N/A | #N/A | #N/A | #N/A |  |  |
| SYDNEY | 5062348 |  | excited | fun | fun | home | weird | hip | fine | posh | Neither Agree nor Disagree | 4 | Neither Agree nor Disagree | 4 | Neither Agree nor Disagree | 4 | Neither Agree nor Disagree | 4 | Neither Agree nor Disagree | 4 | Neither Agree nor Disagree | 4 | Neither Agree nor Disagree | 4 | Neither Agree nor Disagree | 4 | 5062348 |  |  | #N/A | #N/A | #N/A | #N/A |  |  | #N/A | #N/A | #N/A | #N/A |  |  | #N/A | #N/A | #N/A | #N/A |  |  | #N/A | #N/A | #N/A | #N/A |  |  | #N/A | #N/A | #N/A | #N/A |  |  | #N/A | #N/A | #N/A | #N/A |  |  | #N/A | #N/A | #N/A | #N/A |  |  | #N/A | #N/A | #N/A | #N/A |  |  |
| SYDNEY | 5062472 |  | Happiness | Longing | Sadness | Sadness | Anger | Anger | Longing | Happiness | Slightly Agree | 5 | Strongly Disagree | 1 | Slightly Agree | 5 | Slightly Agree | 5 | Strongly Disagree | 1 | Slightly Disagree | 3 | Slightly Disagree | 3 | Slightly Agree | 5 | 5062472 | PRIMED POP (ORIGINAL LYRICS OPERA) | Fear | fear | 2.76 | 6.96 | 3.22 | PRIMED OPERA (ORIGINAL LYRICS FADO) | Anger | anger | 2.34 | 7.63 | 5.5 | PRIMED CUBAN SON (ORIGINAL LYRICS HEAVY METAL) | Sadness | sadness | 2.21 | 5.21 | 2.82 | PRIMED SAMBA (ORIGINAL LYRICS HIP HOP) | Sadness | sadness | 2.21 | 5.21 | 2.82 | PRIMED KOTO (ORIGINAL LYRICS BOLERO) | Sadness | sadness | 2.21 | 5.21 | 2.82 | PRIMED HEAVY METAL (ORIGINAL LYRICS KOTO) | Happiness | happiness | 7.96 | 6.56 | 6.7 | PRIMED FADO (ORIGINAL LYRICS POP) | Anger | anger | 2.34 | 7.63 | 5.5 | PRIMED HIP HOP (ORIGINAL LYRICS SAMBA) | Sadness | sadness | 2.21 | 5.21 | 2.82 |  |  |
| SYDNEY | 5062472 |  | Happiness | Nostalgia | Happiness | Confusion | Anger | Rebellion | Happiness | Happiness | Slightly Agree | 5 | Strongly Disagree | 1 | Slightly Agree | 5 | Slightly Disagree | 3 | Strongly Disagree | 1 | Moderately Disagree | 2 | Slightly Disagree | 3 | Slightly Agree | 5 | 5062472 |  |  | #N/A | #N/A | #N/A | #N/A |  |  | #N/A | #N/A | #N/A | #N/A |  |  | #N/A | #N/A | #N/A | #N/A |  |  | #N/A | #N/A | #N/A | #N/A |  |  | #N/A | #N/A | #N/A | #N/A |  |  | #N/A | #N/A | #N/A | #N/A |  |  | #N/A | #N/A | #N/A | #N/A |  |  | #N/A | #N/A | #N/A | #N/A |  |  |
| SYDNEY | 5062565 |  |  |  |  |  |  |  |  |  |  |  |  |  |  |  |  |  |  |  |  |  |  |  |  |  | 5062565 | PRIMED POP (ORIGINAL LYRICS OPERA) | Delightful | Delightful | 7.8 | 5.58 | 5.69 | PRIMED OPERA (ORIGINAL LYRICS FADO) | Suffering | suffer | 1.72 | 6.13 | 2.54 | PRIMED CUBAN SON (ORIGINAL LYRICS HEAVY METAL) | Uneasy | Uneasy | 3.07 | 6.03 | 3.24 | PRIMED SAMBA (ORIGINAL LYRICS HIP HOP) | Devastated | Devastated | #N/A | #N/A | #N/A | PRIMED SAMBA (ORIGINAL LYRICS HIP HOP) | Sorrowful | sorrow | 2.32 | 4.48 | 3.67 | PRIMED HEAVY METAL (ORIGINAL LYRICS KOTO) | Insecure | insecure | 2.36 | 5.56 | 2.33 | PRIMED FADO (ORIGINAL LYRICS POP) | Unsure | Unsure | 3.03 | 5.97 | 3.6 | PRIMED HIP HOP (ORIGINAL LYRICS SAMBA) | Heartbroken | heartbreak | 1.93 | 5.8 | 3.11 |  |  |
| SYDNEY | z5062565 |  | Excited | Heartbroken | Amused | Unsure | Energised | Rageful | Bored | Sophisticated | Slightly Agree | 5 | Strongly Agree | 7 | Moderately Agree | 6 | Moderately Agree | 6 | Slightly Disagree | 3 | Moderately Agree | 6 | Slightly Agree | 5 | Slightly Agree | 5 | 5062565 |  |  | #N/A | #N/A | #N/A | #N/A |  |  | #N/A | #N/A | #N/A | #N/A |  |  | #N/A | #N/A | #N/A | #N/A |  |  | #N/A | #N/A | #N/A | #N/A |  |  | #N/A | #N/A | #N/A | #N/A |  |  | #N/A | #N/A | #N/A | #N/A |  |  | #N/A | #N/A | #N/A | #N/A |  |  | #N/A | #N/A | #N/A | #N/A |  |  |
| SYDNEY | z5062565 |  | Excited | Heartbroken | Relaxed | Travelled | Energised | Uneasy | Unsure | Sophisticated | Moderately Agree | 6 | Strongly Agree | 7 | Moderately Agree | 6 | Moderately Agree | 6 | Neither Agree nor Disagree | 4 | Slightly Agree | 5 | Slightly Agree | 5 | Slightly Agree | 5 | 5062565 |  |  | #N/A | #N/A | #N/A | #N/A |  |  | #N/A | #N/A | #N/A | #N/A |  |  | #N/A | #N/A | #N/A | #N/A |  |  | #N/A | #N/A | #N/A | #N/A |  |  | #N/A | #N/A | #N/A | #N/A |  |  | #N/A | #N/A | #N/A | #N/A |  |  | #N/A | #N/A | #N/A | #N/A |  |  | #N/A | #N/A | #N/A | #N/A |  |  |
| SYDNEY | 5062912 |  |  |  |  | Asian |  |  | Happiness |  |  |  |  |  |  |  |  |  |  |  |  |  |  |  |  |  | 5062912 |  | romantic | Romantic | 8.32 | 7.59 | 6.08 |  | tense | tense | 3.56 | 6.53 | 5.22 |  | scared | scared | 2.78 | 6.82 | 2.94 |  |  | #N/A | #N/A | #N/A | #N/A |  | sad | sad | 1.61 | 4.13 | 3.45 |  |  | #N/A | #N/A | #N/A | #N/A |  |  | #N/A | #N/A | #N/A | #N/A |  | depressive | depression | 1.85 | 4.54 | 2.91 |  |  |
| SYDNEY | 5062912 |  | happy | tender | intermate | tender | Irritated | energetic | happy | antsy | Slightly Disagree | 3 | Strongly Disagree | 1 | Strongly Agree | 7 | Strongly Agree | 7 | Neither Agree nor Disagree | 4 | Slightly Disagree | 3 | Slightly Disagree | 3 | Slightly Disagree | 3 | 5062912 |  |  | #N/A | #N/A | #N/A | #N/A |  |  | #N/A | #N/A | #N/A | #N/A |  |  | #N/A | #N/A | #N/A | #N/A |  |  | #N/A | #N/A | #N/A | #N/A |  |  | #N/A | #N/A | #N/A | #N/A |  |  | #N/A | #N/A | #N/A | #N/A |  |  | #N/A | #N/A | #N/A | #N/A |  |  | #N/A | #N/A | #N/A | #N/A |  |  |
| SYDNEY | 5062959 |  | sensual | sad | dance | confused | hatred | playful | bubbly | pleased | Slightly Disagree | 3 | Strongly Disagree | 1 | Strongly Agree | 7 | Moderately Agree | 6 | Strongly Disagree | 1 | Strongly Agree | 7 | Slightly Agree | 5 | Slightly Agree | 5 | 5062959 |  |  | #N/A | #N/A | #N/A | #N/A |  |  | #N/A | #N/A | #N/A | #N/A |  |  | #N/A | #N/A | #N/A | #N/A |  |  | #N/A | #N/A | #N/A | #N/A |  |  | #N/A | #N/A | #N/A | #N/A |  |  | #N/A | #N/A | #N/A | #N/A |  |  | #N/A | #N/A | #N/A | #N/A |  |  | #N/A | #N/A | #N/A | #N/A |  |  |
| SYDNEY | 5063035 |  | dancing | romantic | intimacy | weird | angry | I usually feel relaxed when I listen to hip hope (but not this type) there are different types within the hip hop genre | moving around | classy | Moderately Disagree | 2 | Slightly Disagree | 3 | Slightly Agree | 5 | Moderately Agree | 6 | Strongly Disagree | 1 | Slightly Agree | 5 | Neither Agree nor Disagree | 4 | Neither Agree nor Disagree | 4 | 5063035 | PRIMED OPERA (ORIGINAL LYRICS OPERA) | self resepct | self resepct | #N/A | #N/A | #N/A | PRIMED FADO (ORIGINAL LYRICS FADO) | sadness | sadness | 2.21 | 5.21 | 2.82 | PRIMED HEAVY METAL (ORIGINAL LYRICS HEAVY METAL) | distressed | distressed | 1.94 | 6.4 | 3.76 | PRIMED HIP HOP (ORIGINAL LYRICS HIP HOP) | dissapointed | disappointment | 2.37 | 4.6 | 3.2 | PRIMED BOLERO (ORIGINAL LYRICS BOLERO) | hurt | hurt | 1.9 | 5.85 | 3.33 | PRIMED KOTO (ORIGINAL LYRICS KOTO) | spiritual | spirit | 7 | 5.56 | 5.82 | PRIMED POP (ORIGINAL LYRICS POP) | confusion | confusion | 3.46 | 6.07 | 3.04 | PRIMED SAMBA (ORIGINAL LYRICS SAMBA) | heartbreak | heartbreak | 1.93 | 5.8 | 3.11 |  |  |
| SYDNEY | z5063196 |  | happiness | calmness | dancing | calmness | happiness | laidbackness | boredom | calmness | Slightly Disagree | 3 | Strongly Agree | 7 | Slightly Disagree | 3 | Slightly Disagree | 3 | Slightly Disagree | 3 | Strongly Agree | 7 | Slightly Disagree | 3 | Slightly Disagree | 3 | 5063196 | PRIMED OPERA (ORIGINAL LYRICS OPERA) | self-concious | self-concious | #N/A | #N/A | #N/A | PRIMED FADO (ORIGINAL LYRICS FADO) | suffering and patriotism | suffer | 1.72 | 6.13 | 2.54 | PRIMED HEAVY METAL (ORIGINAL LYRICS HEAVY METAL) | darkness | darkness | #N/A | #N/A | #N/A | PRIMED HIP HOP (ORIGINAL LYRICS HIP HOP) | longing and hurt | longing | #N/A | #N/A | #N/A | PRIMED BOLERO (ORIGINAL LYRICS BOLERO) | sadness | sadness | 2.21 | 5.21 | 2.82 | PRIMED KOTO (ORIGINAL LYRICS KOTO) | spiritual | spirit | 7 | 5.56 | 5.82 | PRIMED POP (ORIGINAL LYRICS POP) | anger | anger | 2.34 | 7.63 | 5.5 | PRIMED SAMBA (ORIGINAL LYRICS SAMBA) | fear of commitment | fear of commitment | #N/A | #N/A | #N/A |  |  |
| SYDNEY | 5074962 |  | Confused | Dreamy | Romantic | Sleepy | Energetic | Energetic | Annoyed | Powerful | Slightly Disagree | 3 | Neither Agree nor Disagree | 4 | Slightly Disagree | 3 | Moderately Agree | 6 | Neither Agree nor Disagree | 4 | Slightly Agree | 5 | Neither Agree nor Disagree | 4 | Neither Agree nor Disagree | 4 | 5074962 | PRIMED POP (ORIGINAL LYRICS OPERA) | empowered | power | 6.54 | 6.67 | 7.28 | PRIMED OPERA (ORIGINAL LYRICS FADO) | sAD | sad | 1.61 | 4.13 | 3.45 | PRIMED CUBAN SON (ORIGINAL LYRICS HEAVY METAL) | Fearful | Fearful | 2.25 | 6.33 | 3.64 | PRIMED SAMBA (ORIGINAL LYRICS HIP HOP) | Sad | sad | 1.61 | 4.13 | 3.45 | PRIMED KOTO (ORIGINAL LYRICS BOLERO) | sad | sad | 1.61 | 4.13 | 3.45 | PRIMED HEAVY METAL (ORIGINAL LYRICS KOTO) | Spiritual | spirit | 7 | 5.56 | 5.82 | PRIMED FADO (ORIGINAL LYRICS POP) | Humoured | Humoured | #N/A | #N/A | #N/A | PRIMED HIP HOP (ORIGINAL LYRICS SAMBA) | interest | interest | 6.97 | 5.66 | 5.89 |  |  |
| SYDNEY | z5075367 |  | Happy | Romantic | Romantic | Pensive | Energised | Cool | Happy | Relaxed | Strongly Agree | 7 | Slightly Agree | 5 | Strongly Agree | 7 | Moderately Agree | 6 | Neither Agree nor Disagree | 4 | Strongly Agree | 7 | Strongly Agree | 7 | Strongly Agree | 7 | 5075367 | UNPRIMED (ORIGINAL LYRICS OPERA) | Exposed | Exposed | #N/A | #N/A | #N/A | UNPRIMED (ORIGINAL LYRICS FADO) | Valued | Valued | #N/A | #N/A | #N/A | UNPRIMED (ORIGINAL LYRICS HEAVY METAL) | Angry | angry | 2.85 | 7.17 | 5.55 | UNPRIMED (ORIGINAL LYRICS HIP HOP) | Abandoned | Abandoned | #N/A | #N/A | #N/A | UNPRIMED (ORIGINAL LYRICS BOLERO) | Hurt | hurt | 1.9 | 5.85 | 3.33 | UNPRIMED (ORIGINAL LYRICS BOLERO) | Free | Free | 8.26 | 5.15 | 6.35 | UNPRIMED (ORIGINAL LYRICS POP) | Care-less | careless | 3.9 | 4.61 | 4.66 | UNPRIMED (ORIGINAL LYRICS SAMBA) | Sad | sad | 1.61 | 4.13 | 3.45 |  |  |
| SYDNEY | 5075385 |  | I feel like dancing | nostalgic | dancing | artistic | jumpy/agressive | chilling | It really depends on what song it is and who the artist is...If it's Adele - I feel nostagic and sad. If it's Taylor Swift, I feel empowered. If it's Kayne West- I get annoyed. etc | refreshed | I don't know this music | 8 | Moderately Disagree | 2 | Strongly Agree | 7 | Moderately Agree | 6 | Neither Agree nor Disagree | 4 | Strongly Agree | 7 | Slightly Agree | 5 | Slightly Disagree | 3 | 5075385 | UNPRIMED (ORIGINAL LYRICS OPERA) | empathy | empathy | 5.32 | 4.81 | 4.33 | UNPRIMED (ORIGINAL LYRICS FADO) | sympathetic | sympathetic | 5.74 | 3.99 | 5.59 | UNPRIMED (ORIGINAL LYRICS HEAVY METAL) | intense | intense | 5.44 | 6.32 | 5.72 | UNPRIMED (ORIGINAL LYRICS HIP HOP) | confused | confused | 3.21 | 6.03 | 4.24 | UNPRIMED (ORIGINAL LYRICS BOLERO) | cliche | cliche | #N/A | #N/A | #N/A | UNPRIMED (ORIGINAL LYRICS BOLERO) | secure | secure | 7.57 | 3.14 | 5.93 | UNPRIMED (ORIGINAL LYRICS POP) | confused | confused | 3.21 | 6.03 | 4.24 | UNPRIMED (ORIGINAL LYRICS SAMBA) | cliche | cliche | #N/A | #N/A | #N/A |  |  |
| SYDNEY | z5075493 |  | party | Romantic | Chill | Foreign | Angry | Celebration | Easy-going | Regal | I don't know this music | 8 | Strongly Disagree | 1 | Moderately Agree | 6 | Strongly Agree | 7 | I don't know this music | 8 | Moderately Agree | 6 | I don't know this music | 8 | I don't know this music | 8 | 5075493 |  |  | #N/A | #N/A | #N/A | #N/A |  |  | #N/A | #N/A | #N/A | #N/A |  |  | #N/A | #N/A | #N/A | #N/A |  |  | #N/A | #N/A | #N/A | #N/A |  |  | #N/A | #N/A | #N/A | #N/A |  |  | #N/A | #N/A | #N/A | #N/A |  |  | #N/A | #N/A | #N/A | #N/A |  |  | #N/A | #N/A | #N/A | #N/A |  |  |
| SYDNEY | 5075619 |  | excitement | calmness | calmness | Calmness | overwhelmed | exitement | Happy | calmness | Neither Agree nor Disagree | 4 | Strongly Disagree | 1 | Strongly Agree | 7 | Strongly Agree | 7 | I don't know this music | 8 | Slightly Agree | 5 | I don't know this music | 8 | I don't know this music | 8 | 5075619 | PRIMED OPERA (ORIGINAL LYRICS OPERA) | story | story | 6.63 | 5.37 | 5.47 | PRIMED FADO (ORIGINAL LYRICS FADO) | poetic | Poetic | #N/A | #N/A | #N/A | PRIMED HEAVY METAL (ORIGINAL LYRICS HEAVY METAL) | dark | Dark | 4.71 | 4.28 | 4.84 | PRIMED HIP HOP (ORIGINAL LYRICS HIP HOP) | drama | drama | 3.86 | 6.39 | 5.32 | PRIMED BOLERO (ORIGINAL LYRICS BOLERO) | emotional | Emotional | 4.36 | 5.75 | 4.29 | PRIMED KOTO (ORIGINAL LYRICS KOTO) | translated | translated | #N/A | #N/A | #N/A | PRIMED POP (ORIGINAL LYRICS POP) | generic | generic | #N/A | #N/A | #N/A | PRIMED SAMBA (ORIGINAL LYRICS SAMBA) | Amorous | Amorous | #N/A | #N/A | #N/A |  |  |
| SYDNEY | 5075713 |  | Makes me feel excited | Calmness | Love | reflection | Excitement | joy | tender feelings | Overwhelming awareness of my senses | Moderately Agree | 6 | Neither Agree nor Disagree | 4 | Strongly Agree | 7 | Strongly Agree | 7 | Slightly Agree | 5 | Slightly Agree | 5 | Moderately Agree | 6 | Moderately Agree | 6 | 5075713 | PRIMED POP (ORIGINAL LYRICS OPERA) | happiness | happiness | 7.96 | 6.56 | 6.7 | PRIMED OPERA (ORIGINAL LYRICS FADO) | sadness | sadness | 2.21 | 5.21 | 2.82 | PRIMED CUBAN SON (ORIGINAL LYRICS HEAVY METAL) | alarmed | alarmed | #N/A | #N/A | #N/A | PRIMED SAMBA (ORIGINAL LYRICS HIP HOP) | sadness | sadness | 2.21 | 5.21 | 2.82 | PRIMED KOTO (ORIGINAL LYRICS BOLERO) | low-spirited | low-spirited | #N/A | #N/A | #N/A | PRIMED HEAVY METAL (ORIGINAL LYRICS KOTO) | sulky | sulky | #N/A | #N/A | #N/A | PRIMED FADO (ORIGINAL LYRICS POP) | contempt | contempt | 3.85 | 5.28 | 5.13 | PRIMED HIP HOP (ORIGINAL LYRICS SAMBA) | sadness | sadness | 2.21 | 5.21 | 2.82 |  |  |
| SYDNEY | 5075717 |  |  |  |  |  |  |  |  |  |  |  |  |  |  |  |  |  |  |  |  |  |  |  |  |  | 5075717 |  |  | #N/A | #N/A | #N/A | #N/A |  |  | #N/A | #N/A | #N/A | #N/A |  |  | #N/A | #N/A | #N/A | #N/A |  |  | #N/A | #N/A | #N/A | #N/A |  |  | #N/A | #N/A | #N/A | #N/A |  |  | #N/A | #N/A | #N/A | #N/A |  |  | #N/A | #N/A | #N/A | #N/A |  |  | #N/A | #N/A | #N/A | #N/A |  |  |
| SYDNEY | 5075717 |  | Spirited | Melancholy | Energetic | Meditative | Anger | Frustration | Joyful | Lively | Neither Agree nor Disagree | 4 | Slightly Disagree | 3 | Strongly Agree | 7 | Slightly Agree | 5 | Moderately Disagree | 2 | Strongly Agree | 7 | Neither Agree nor Disagree | 4 | Neither Agree nor Disagree | 4 | 5075717 | PRIMED OPERA (ORIGINAL LYRICS OPERA) | Sensual | Sensual | #N/A | #N/A | #N/A | PRIMED FADO (ORIGINAL LYRICS FADO) | Nostalgic | Nostalgic | #N/A | #N/A | #N/A | PRIMED HEAVY METAL (ORIGINAL LYRICS HEAVY METAL) | Anger | anger | 2.34 | 7.63 | 5.5 | PRIMED HIP HOP (ORIGINAL LYRICS HIP HOP) | Sadness | sadness | 2.21 | 5.21 | 2.82 | PRIMED BOLERO (ORIGINAL LYRICS BOLERO) | Melancholy | melancholy | 3.82 | 3.6 | 4.65 | PRIMED KOTO (ORIGINAL LYRICS KOTO) | Spiritual | spirit | 7 | 5.56 | 5.82 | PRIMED POP (ORIGINAL LYRICS POP) | Resentful | resent | 3.76 | 4.47 | 4.46 | PRIMED SAMBA (ORIGINAL LYRICS SAMBA) | Passionate | passion | 8.03 | 7.26 | 6.13 |  |  |
| SYDNEY | z5075942 |  | dance | longing | high-spirited | unfamiliar | irritated | motivated | happy | relaxed | Strongly Disagree | 1 | Strongly Disagree | 1 | Strongly Agree | 7 | Moderately Agree | 6 | Strongly Disagree | 1 | Moderately Agree | 6 | Strongly Disagree | 1 | Strongly Disagree | 1 | 5075942 | PRIMED POP (ORIGINAL LYRICS OPERA) | pride | Pride | 7 | 5.83 | 7.06 | PRIMED OPERA (ORIGINAL LYRICS FADO) | sadness | sadness | 2.21 | 5.21 | 2.82 | PRIMED CUBAN SON (ORIGINAL LYRICS HEAVY METAL) | hate | Hate | 2.12 | 6.95 | 5.05 | PRIMED SAMBA (ORIGINAL LYRICS HIP HOP) | disappointment | Disappointment | 2.37 | 4.6 | 3.2 | PRIMED KOTO (ORIGINAL LYRICS BOLERO) | hate | Hate | 2.12 | 6.95 | 5.05 | PRIMED HEAVY METAL (ORIGINAL LYRICS KOTO) | hope | Hope | 7.05 | 5.44 | 5.52 | PRIMED FADO (ORIGINAL LYRICS POP) | hate | Hate | 2.12 | 6.95 | 5.05 | PRIMED HIP HOP (ORIGINAL LYRICS SAMBA) | scared | scared | 2.78 | 6.82 | 2.94 |  |  |
| SYDNEY | 5075942 |  |  |  |  |  |  |  |  |  |  |  |  |  |  |  |  |  |  |  |  |  |  |  |  |  | 5075942 |  |  | #N/A | #N/A | #N/A | #N/A |  |  | #N/A | #N/A | #N/A | #N/A |  |  | #N/A | #N/A | #N/A | #N/A |  |  | #N/A | #N/A | #N/A | #N/A |  |  | #N/A | #N/A | #N/A | #N/A |  |  | #N/A | #N/A | #N/A | #N/A |  |  | #N/A | #N/A | #N/A | #N/A |  |  | #N/A | #N/A | #N/A | #N/A |  |  |
| SYDNEY | z5076055 |  | lust | sadness | vengeful | sadness | calming | anger | joy | overwhelmed | Slightly Disagree | 3 | Slightly Agree | 5 | Strongly Agree | 7 | Slightly Agree | 5 | I don't know this music | 8 | Moderately Agree | 6 | Slightly Disagree | 3 | Slightly Disagree | 3 | 5076055 | UNPRIMED (ORIGINAL LYRICS OPERA) | satisfaction | satisfaction | 7.69 | 6.83 | 6.76 | UNPRIMED (ORIGINAL LYRICS FADO) | anger | anger | 2.34 | 7.63 | 5.5 | UNPRIMED (ORIGINAL LYRICS HEAVY METAL) | anger | anger | 2.34 | 7.63 | 5.5 | UNPRIMED (ORIGINAL LYRICS HIP HOP) | loneliness | loneliness | 1.61 | 4.56 | 2.51 | UNPRIMED (ORIGINAL LYRICS BOLERO) | betrayal | Betrayal | 2.28 | 5.37 | 4.18 | UNPRIMED (ORIGINAL LYRICS BOLERO) | calming | calm | 6.73 | 3.6 | 6.37 | UNPRIMED (ORIGINAL LYRICS POP) | pride | Pride | 7 | 5.83 | 7.06 | UNPRIMED (ORIGINAL LYRICS SAMBA) | heartbreak | heartbreak | 1.93 | 5.8 | 3.11 |  |  |
| SYDNEY | z5076068 |  | relaxing | relaxed | groovy | calm | intense | dancing | dancing | calm | I don't know this music | 8 | Strongly Disagree | 1 | Neither Agree nor Disagree | 4 | Neither Agree nor Disagree | 4 | I don't know this music | 8 | I don't know this music | 8 | I don't know this music | 8 | I don't know this music | 8 | 5076068 |  |  | #N/A | #N/A | #N/A | #N/A |  |  | #N/A | #N/A | #N/A | #N/A |  |  | #N/A | #N/A | #N/A | #N/A |  |  | #N/A | #N/A | #N/A | #N/A |  |  | #N/A | #N/A | #N/A | #N/A |  |  | #N/A | #N/A | #N/A | #N/A |  |  | #N/A | #N/A | #N/A | #N/A |  |  | #N/A | #N/A | #N/A | #N/A |  |  |
| SYDNEY | z5076137 |  | Delighted | Smooth Flowing | Confident | Forced | Rough | Tough and rigid | Joyous | Proud | Moderately Disagree | 2 | Strongly Disagree | 1 | Strongly Agree | 7 | Slightly Disagree | 3 | I don't know this music | 8 | Moderately Agree | 6 | I don't know this music | 8 | Strongly Disagree | 1 | 5076137 | PRIMED POP (ORIGINAL LYRICS OPERA) | love | love | 8.72 | 6.44 | 7.11 | PRIMED OPERA (ORIGINAL LYRICS FADO) | Despair | Despair | 2.99 | 4.49 | 4.3 | PRIMED CUBAN SON (ORIGINAL LYRICS HEAVY METAL) | intense | intense | 5.44 | 6.32 | 5.72 | PRIMED SAMBA (ORIGINAL LYRICS HIP HOP) | Sadness | sadness | 2.21 | 5.21 | 2.82 | PRIMED KOTO (ORIGINAL LYRICS BOLERO) | Empowering | power | 6.54 | 6.67 | 7.28 | PRIMED HEAVY METAL (ORIGINAL LYRICS KOTO) | evil | evil | 3.23 | 6.39 | 5.25 | PRIMED FADO (ORIGINAL LYRICS POP) | empowering | power | 6.54 | 6.67 | 7.28 | PRIMED HIP HOP (ORIGINAL LYRICS SAMBA) | fear | fear | 2.76 | 6.96 | 3.22 |  |  |
| SYDNEY | z5076137 |  |  |  |  |  |  |  |  |  |  |  |  |  |  |  |  |  |  |  |  |  |  |  |  |  | 5076137 |  |  | #N/A | #N/A | #N/A | #N/A |  |  | #N/A | #N/A | #N/A | #N/A |  |  | #N/A | #N/A | #N/A | #N/A |  |  | #N/A | #N/A | #N/A | #N/A |  |  | #N/A | #N/A | #N/A | #N/A |  |  | #N/A | #N/A | #N/A | #N/A |  |  | #N/A | #N/A | #N/A | #N/A |  |  | #N/A | #N/A | #N/A | #N/A |  |  |
| SYDNEY | 5076137 |  |  |  |  |  |  |  |  |  |  |  |  |  |  |  |  |  |  |  |  |  |  |  |  |  | 5076137 |  |  | #N/A | #N/A | #N/A | #N/A |  |  | #N/A | #N/A | #N/A | #N/A |  |  | #N/A | #N/A | #N/A | #N/A |  |  | #N/A | #N/A | #N/A | #N/A |  |  | #N/A | #N/A | #N/A | #N/A |  |  | #N/A | #N/A | #N/A | #N/A |  |  | #N/A | #N/A | #N/A | #N/A |  |  | #N/A | #N/A | #N/A | #N/A |  |  |
| SYDNEY | z5076351 |  | Excited | Calm | Excited | Peaceful | Annoyed | groovy | Happy | Peaceful | Strongly Disagree | 1 | Strongly Disagree | 1 | Strongly Agree | 7 | Strongly Agree | 7 | Strongly Agree | 7 | Strongly Agree | 7 | Slightly Disagree | 3 | Slightly Disagree | 3 | 5076351 | PRIMED POP (ORIGINAL LYRICS OPERA) | Powerful | Powerful | 6.84 | 5.83 | 7.19 | PRIMED OPERA (ORIGINAL LYRICS FADO) | sad | sad | 1.61 | 4.13 | 3.45 | PRIMED CUBAN SON (ORIGINAL LYRICS HEAVY METAL) | badass | badass | #N/A | #N/A | #N/A | PRIMED SAMBA (ORIGINAL LYRICS HIP HOP) | angry | angry | 2.85 | 7.17 | 5.55 | PRIMED KOTO (ORIGINAL LYRICS BOLERO) | Badass | badass | #N/A | #N/A | #N/A | PRIMED HEAVY METAL (ORIGINAL LYRICS KOTO) | badass | badass | #N/A | #N/A | #N/A | PRIMED FADO (ORIGINAL LYRICS POP) | Badass | badass | #N/A | #N/A | #N/A | PRIMED HIP HOP (ORIGINAL LYRICS SAMBA) | Sad | sad | 1.61 | 4.13 | 3.45 |  |  |
| SYDNEY | z5076351 |  | Happy | jnj | Groovey | Angry | LOL | badass | annoyed | Calm | Strongly Disagree | 1 | Strongly Disagree | 1 | Strongly Disagree | 1 | Strongly Disagree | 1 | Strongly Disagree | 1 | Strongly Disagree | 1 | Strongly Disagree | 1 | Strongly Disagree | 1 | 5076351 | UNPRIMED (ORIGINAL LYRICS OPERA) | LOL? | LOL? | #N/A | #N/A | #N/A | UNPRIMED (ORIGINAL LYRICS FADO) | Sad | sad | 1.61 | 4.13 | 3.45 | UNPRIMED (ORIGINAL LYRICS HEAVY METAL) | Angry | angry | 2.85 | 7.17 | 5.55 | UNPRIMED (ORIGINAL LYRICS HIP HOP) | betrayal | Betrayal | 2.28 | 5.37 | 4.18 | UNPRIMED (ORIGINAL LYRICS BOLERO) | sad | sad | 1.61 | 4.13 | 3.45 | UNPRIMED (ORIGINAL LYRICS BOLERO) | purity | Purity | #N/A | #N/A | #N/A | UNPRIMED (ORIGINAL LYRICS POP) | angry | angry | 2.85 | 7.17 | 5.55 | UNPRIMED (ORIGINAL LYRICS SAMBA) | | #N/A | #N/A | #N/A | #N/A |  |  |
| SYDNEY | Z5076466 |  | Happy, shift my mood to a holida. y mood. However, the second one is very disturbing | Happiness.   Romantic, relaxing. it reminds me of weddings in my culture | relaxation. | It evokes my imagination of the Japanese culture.  The second one is relaxing | Noise, action. | Excitement | Excited | Very calming   it reminds me of old movies | Strongly Agree | 7 | Moderately Disagree | 2 | Neither Agree nor Disagree | 4 | Strongly Agree | 7 | Neither Agree nor Disagree | 4 | Strongly Agree | 7 | Strongly Agree | 7 | Neither Agree nor Disagree | 4 | 5076466 | UNPRIMED (ORIGINAL LYRICS OPERA) | attraction | attractive | 7.49 | 6.76 | 5.54 | UNPRIMED (ORIGINAL LYRICS FADO) | Not sure | Not sure | #N/A | #N/A | #N/A | UNPRIMED (ORIGINAL LYRICS HEAVY METAL) | Sombre | sombre | #N/A | #N/A | #N/A | UNPRIMED (ORIGINAL LYRICS HIP HOP) | | #N/A | #N/A | #N/A | #N/A | UNPRIMED (ORIGINAL LYRICS BOLERO) | betrayal | Betrayal | 2.28 | 5.37 | 4.18 | UNPRIMED (ORIGINAL LYRICS BOLERO) | not sure | Not sure | #N/A | #N/A | #N/A | UNPRIMED (ORIGINAL LYRICS POP) | Not sure | Not sure | #N/A | #N/A | #N/A | UNPRIMED (ORIGINAL LYRICS SAMBA) | Worry | Worry | 2.31 | 6 | 2.96 |  |  |
| SYDNEY | 5076872 |  | cool | calm | Dance | meditative | anger | groove | happy | intelligent | Slightly Disagree | 3 | Neither Agree nor Disagree | 4 | Strongly Agree | 7 | Moderately Agree | 6 | Strongly Disagree | 1 | Slightly Agree | 5 | Strongly Disagree | 1 | Slightly Disagree | 3 | 5076872 |  |  | #N/A | #N/A | #N/A | #N/A |  |  | #N/A | #N/A | #N/A | #N/A |  |  | #N/A | #N/A | #N/A | #N/A |  |  | #N/A | #N/A | #N/A | #N/A |  |  | #N/A | #N/A | #N/A | #N/A |  |  | #N/A | #N/A | #N/A | #N/A |  |  | #N/A | #N/A | #N/A | #N/A |  |  | #N/A | #N/A | #N/A | #N/A |  |  |
| SYDNEY | z5076887 |  | relaxed. | sad | happy | reflective | Energetic | groovy | annoyed | tender | Slightly Agree | 5 | Moderately Agree | 6 | Neither Agree nor Disagree | 4 | Moderately Agree | 6 | Moderately Agree | 6 | Strongly Agree | 7 | Moderately Agree | 6 | Moderately Agree | 6 | 5076887 | PRIMED POP (ORIGINAL LYRICS OPERA) | stupidity | stupidity | #N/A | #N/A | #N/A | PRIMED OPERA (ORIGINAL LYRICS FADO) | emotionless | emotionless | #N/A | #N/A | #N/A | PRIMED CUBAN SON (ORIGINAL LYRICS HEAVY METAL) | turmoil | turmoil | 2.71 | 5.87 | 3.2 | PRIMED SAMBA (ORIGINAL LYRICS HIP HOP) | sorrow | sorrow | 2.32 | 4.48 | 3.67 | PRIMED KOTO (ORIGINAL LYRICS BOLERO) | longing | Longing | #N/A | #N/A | #N/A | PRIMED HEAVY METAL (ORIGINAL LYRICS KOTO) | fear | fear | 2.76 | 6.96 | 3.22 | PRIMED FADO (ORIGINAL LYRICS POP) | inspired | Inspired | 7.15 | 6.02 | 6.67 | PRIMED HIP HOP (ORIGINAL LYRICS SAMBA) | nothing | nothing | #N/A | #N/A | #N/A |  |  |
| SYDNEY | z5077187 |  | Neutral | Relax | Happy | Neutral | Cool | Cool | Chill | Relax | Slightly Disagree | 3 | Slightly Disagree | 3 | Strongly Agree | 7 | Moderately Agree | 6 | Slightly Disagree | 3 | Strongly Agree | 7 | Slightly Disagree | 3 | Slightly Disagree | 3 | 5077187 | UNPRIMED (ORIGINAL LYRICS OPERA) | Neutral | Neutral | #N/A | #N/A | #N/A | UNPRIMED (ORIGINAL LYRICS FADO) | Neutral | Neutral | #N/A | #N/A | #N/A | UNPRIMED (ORIGINAL LYRICS HEAVY METAL) | Weird | weird | 5.17 | 4.82 | 5.13 | UNPRIMED (ORIGINAL LYRICS HIP HOP) | Sad | sad | 1.61 | 4.13 | 3.45 | UNPRIMED (ORIGINAL LYRICS BOLERO) | Sad | sad | 1.61 | 4.13 | 3.45 | UNPRIMED (ORIGINAL LYRICS BOLERO) | Calm | calm | 6.73 | 3.6 | 6.37 | UNPRIMED (ORIGINAL LYRICS POP) | Weird | weird | 5.17 | 4.82 | 5.13 | UNPRIMED (ORIGINAL LYRICS SAMBA) | Neutral | Neutral | #N/A | #N/A | #N/A |  |  |
| SYDNEY | 5078362 |  | Relaxed | Sadness | Happiness | Curiosity | Excitement | Hyped (energised) | Cool | Awe | Slightly Agree | 5 | Slightly Agree | 5 | Slightly Agree | 5 | Strongly Agree | 7 | Strongly Disagree | 1 | Moderately Agree | 6 | Slightly Disagree | 3 | Neither Agree nor Disagree | 4 | 5078362 | PRIMED POP (ORIGINAL LYRICS OPERA) | weird | weird | 5.17 | 4.82 | 5.13 | PRIMED OPERA (ORIGINAL LYRICS FADO) | stupid | stupid | 2.31 | 4.72 | 2.98 | PRIMED CUBAN SON (ORIGINAL LYRICS HEAVY METAL) | confused | confused | 3.21 | 6.03 | 4.24 | PRIMED SAMBA (ORIGINAL LYRICS HIP HOP) | | #N/A | #N/A | #N/A | #N/A | PRIMED KOTO (ORIGINAL LYRICS BOLERO) | sad | sad | 1.61 | 4.13 | 3.45 | PRIMED HEAVY METAL (ORIGINAL LYRICS KOTO) | intrigue | intrigue | #N/A | #N/A | #N/A | PRIMED FADO (ORIGINAL LYRICS POP) | that is not fado | that is not fado | #N/A | #N/A | #N/A | PRIMED HIP HOP (ORIGINAL LYRICS SAMBA) | meh | meh | #N/A | #N/A | #N/A |  |  |
| SYDNEY | 5078554 |  | Happy | Relaxing | Happy | Content | Savage | Excitement | Boring | Boring | Neither Agree nor Disagree | 4 | Slightly Agree | 5 | Moderately Disagree | 2 | Strongly Agree | 7 | Neither Agree nor Disagree | 4 | Neither Agree nor Disagree | 4 | Neither Agree nor Disagree | 4 | Neither Agree nor Disagree | 4 | 5078554 | UNPRIMED (ORIGINAL LYRICS OPERA) | Arrogance | arrogance | 3.15 | 4.85 | 5.32 | UNPRIMED (ORIGINAL LYRICS FADO) | sad | sad | 1.61 | 4.13 | 3.45 | UNPRIMED (ORIGINAL LYRICS HEAVY METAL) | guilt | Guilt | 2.14 | 5.36 | 2.96 | UNPRIMED (ORIGINAL LYRICS HIP HOP) | betrayal | Betrayal | 2.28 | 5.37 | 4.18 | UNPRIMED (ORIGINAL LYRICS BOLERO) | sad | sad | 1.61 | 4.13 | 3.45 | UNPRIMED (ORIGINAL LYRICS BOLERO) | afraid | Afraid | 2 | 6.67 | 3.98 | UNPRIMED (ORIGINAL LYRICS POP) | excited | excitement | 7.5 | 7.67 | 6.18 | UNPRIMED (ORIGINAL LYRICS SAMBA) | sad | sad | 1.61 | 4.13 | 3.45 |  |  |
| SYDNEY | 5079458 |  | celebration | relaxation | love | reflection | anger | annoyance | neutral | happiness | Neither Agree nor Disagree | 4 | Strongly Disagree | 1 | Slightly Agree | 5 | Moderately Disagree | 2 | I don't know this music | 8 | Strongly Agree | 7 | Neither Agree nor Disagree | 4 | Neither Agree nor Disagree | 4 | 5079458 | PRIMED OPERA (ORIGINAL LYRICS OPERA) | intrigue | intrigue | #N/A | #N/A | #N/A |  |  | #N/A | #N/A | #N/A | #N/A |  |  | #N/A | #N/A | #N/A | #N/A |  |  | #N/A | #N/A | #N/A | #N/A |  |  | #N/A | #N/A | #N/A | #N/A |  |  | #N/A | #N/A | #N/A | #N/A |  |  | #N/A | #N/A | #N/A | #N/A |  |  | #N/A | #N/A | #N/A | #N/A |  |  |
| SYDNEY | 5080369 |  | lively | complicate | operatic | classical | strong | rhythm sensation | excited | fantasy | Moderately Disagree | 2 | Moderately Disagree | 2 | Moderately Agree | 6 | Moderately Agree | 6 | Neither Agree nor Disagree | 4 | Neither Agree nor Disagree | 4 | Neither Agree nor Disagree | 4 | Neither Agree nor Disagree | 4 | 5080369 | PRIMED OPERA (ORIGINAL LYRICS OPERA) | Junoesque | Junoesque | #N/A | #N/A | #N/A | PRIMED FADO (ORIGINAL LYRICS FADO) | depression | depression | 1.85 | 4.54 | 2.91 | PRIMED HEAVY METAL (ORIGINAL LYRICS HEAVY METAL) | anger | anger | 2.34 | 7.63 | 5.5 | PRIMED HIP HOP (ORIGINAL LYRICS HIP HOP) | yearning | yearning | #N/A | #N/A | #N/A | PRIMED BOLERO (ORIGINAL LYRICS BOLERO) | resignation | resign | 3.47 | 4.21 | 4.57 | PRIMED KOTO (ORIGINAL LYRICS KOTO) | spiritual | spirit | 7 | 5.56 | 5.82 | PRIMED POP (ORIGINAL LYRICS POP) | disgust | disgusting | 2.96 | 5.18 | 3.64 | PRIMED SAMBA (ORIGINAL LYRICS SAMBA) | despair | Despair | 2.99 | 4.49 | 4.3 |  |  |
| SYDNEY | z5083730 |  | enthusiasm | melancholy | lively | strange | uncomfortable | rhythm | cheerful | solemn | Neither Agree nor Disagree | 4 | Moderately Disagree | 2 | Slightly Agree | 5 | Slightly Agree | 5 | Slightly Disagree | 3 | Neither Agree nor Disagree | 4 | Neither Agree nor Disagree | 4 | Neither Agree nor Disagree | 4 | 5083730 | PRIMED OPERA (ORIGINAL LYRICS OPERA) | enthusiasm | enthusiasm | 7.67 | 6.55 | 5.98 | PRIMED FADO (ORIGINAL LYRICS FADO) | impassioned | impassioned | #N/A | #N/A | #N/A | PRIMED HEAVY METAL (ORIGINAL LYRICS HEAVY METAL) | fear | fear | 2.76 | 6.96 | 3.22 | PRIMED HIP HOP (ORIGINAL LYRICS HIP HOP) | firm and indomitable | firm and indomitable | #N/A | #N/A | #N/A | PRIMED BOLERO (ORIGINAL LYRICS BOLERO) | worried | worried | #N/A | #N/A | #N/A | PRIMED KOTO (ORIGINAL LYRICS KOTO) | complicate | complicate | #N/A | #N/A | #N/A | PRIMED POP (ORIGINAL LYRICS POP) | interrogative | interrogative | #N/A | #N/A | #N/A | PRIMED SAMBA (ORIGINAL LYRICS SAMBA) | sad | sad | 1.61 | 4.13 | 3.45 |  |  |
| SYDNEY | z5083768 |  | joyful | calm | calm | pessimism | restlessness | excited | happy | excited | Slightly Agree | 5 | Moderately Disagree | 2 | Moderately Agree | 6 | Slightly Agree | 5 | Slightly Disagree | 3 | Neither Agree nor Disagree | 4 | Slightly Agree | 5 | Slightly Agree | 5 | 5083768 | PRIMED POP (ORIGINAL LYRICS OPERA) | happy | happy | 8.21 | 6.49 | 6.63 | PRIMED OPERA (ORIGINAL LYRICS FADO) | excited | excitement | 7.5 | 7.67 | 6.18 | PRIMED CUBAN SON (ORIGINAL LYRICS HEAVY METAL) | indignation | indignation | #N/A | #N/A | #N/A | PRIMED SAMBA (ORIGINAL LYRICS HIP HOP) | heartbreak | heartbreak | 1.93 | 5.8 | 3.11 | PRIMED KOTO (ORIGINAL LYRICS BOLERO) | sad | sad | 1.61 | 4.13 | 3.45 | PRIMED HEAVY METAL (ORIGINAL LYRICS KOTO) | gloomy | gloomy | 3.18 | 3.35 | 4.77 | PRIMED FADO (ORIGINAL LYRICS POP) | confused | confused | 3.21 | 6.03 | 4.24 | PRIMED HIP HOP (ORIGINAL LYRICS SAMBA) | distressed | distressed | 1.94 | 6.4 | 3.76 |  |  |
| SYDNEY | z5083779 |  | engratic | sad | pleasent | sad | hate | happy | high | great | Slightly Agree | 5 | Strongly Disagree | 1 | Moderately Agree | 6 | Moderately Agree | 6 | Moderately Agree | 6 | Strongly Agree | 7 | I don't know this music | 8 | I don't know this music | 8 | 5083779 | UNPRIMED (ORIGINAL LYRICS OPERA) | happy | happy | 8.21 | 6.49 | 6.63 | UNPRIMED (ORIGINAL LYRICS FADO) | sad | sad | 1.61 | 4.13 | 3.45 | UNPRIMED (ORIGINAL LYRICS HEAVY METAL) | postive | positive | 8.07 | 5.57 | 6.19 | UNPRIMED (ORIGINAL LYRICS HIP HOP) | despairing | despairing | 2.43 | 5.68 | 3.43 | UNPRIMED (ORIGINAL LYRICS BOLERO) | sad | sad | 1.61 | 4.13 | 3.45 | UNPRIMED (ORIGINAL LYRICS BOLERO) | angry | angry | 2.85 | 7.17 | 5.55 | UNPRIMED (ORIGINAL LYRICS POP) | angry | angry | 2.85 | 7.17 | 5.55 | UNPRIMED (ORIGINAL LYRICS SAMBA) | sad | sad | 1.61 | 4.13 | 3.45 |  |  |
| SYDNEY | z5093849 |  | cheerful | romantic | nostalgic | praying | annoyed | playful | love | Movie | Slightly Agree | 5 | Strongly Disagree | 1 | Moderately Agree | 6 | Slightly Agree | 5 | Neither Agree nor Disagree | 4 | Slightly Agree | 5 | Slightly Agree | 5 | Slightly Agree | 5 | 5093849 | PRIMED OPERA (ORIGINAL LYRICS OPERA) | proud | Proud | 8.03 | 5.56 | 6.74 |  |  | #N/A | #N/A | #N/A | #N/A |  |  | #N/A | #N/A | #N/A | #N/A |  |  | #N/A | #N/A | #N/A | #N/A |  |  | #N/A | #N/A | #N/A | #N/A |  |  | #N/A | #N/A | #N/A | #N/A |  |  | #N/A | #N/A | #N/A | #N/A |  |  | #N/A | #N/A | #N/A | #N/A |  |  |
| SYDNEY | 5096182 |  | Happy | longing | relaxed | lament | Angry | passionate | Pleased | elegent | I don't know this music | 8 | Strongly Disagree | 1 | Strongly Agree | 7 | Moderately Agree | 6 | I don't know this music | 8 | Slightly Agree | 5 | I don't know this music | 8 | I don't know this music | 8 | 5096182 | PRIMED POP (ORIGINAL LYRICS OPERA) | jugdemental | jugdemental | #N/A | #N/A | #N/A | PRIMED OPERA (ORIGINAL LYRICS FADO) | sadness | sadness | 2.21 | 5.21 | 2.82 | PRIMED CUBAN SON (ORIGINAL LYRICS HEAVY METAL) | loss | Loss | 1.89 | 5.78 | 2.38 | PRIMED SAMBA (ORIGINAL LYRICS HIP HOP) | sadness | sadness | 2.21 | 5.21 | 2.82 | PRIMED KOTO (ORIGINAL LYRICS BOLERO) | helpless | helpless | 2.2 | 5.34 | 2.27 | PRIMED HEAVY METAL (ORIGINAL LYRICS KOTO) | sacrifice | sacrifice | #N/A | #N/A | #N/A | PRIMED FADO (ORIGINAL LYRICS POP) | confused | confused | 3.21 | 6.03 | 4.24 | PRIMED HIP HOP (ORIGINAL LYRICS SAMBA) | heartbreak | heartbreak | 1.93 | 5.8 | 3.11 |  |  |
| SYDNEY | z5097497 |  | I feel relaxed. I feel like dancing. I feel like I'm in love. | I feel relaxed | I feel like I'm in love. I feel like dancing. It is a little bit the same as Brazilian Samba. I feel like i wanted to dance. | I feel confused | I feel dangerous. I feel energetic. I feel I wanted to punch people in the face. i feel happy | I feel like dancing. I feel dangerous. I feel like I've been smoking weed. I feel like I am a gangster | Pop music does not really evoke my emotion. I don't feel anything listening to the music. Probably a little bit bored | I feel relaxed. I feel rich. I feel like I'm in a deep thought | Slightly Agree | 5 | Moderately Agree | 6 | Moderately Disagree | 2 | Moderately Agree | 6 | Moderately Disagree | 2 | Neither Agree nor Disagree | 4 | Slightly Disagree | 3 | Slightly Agree | 5 | 5097497 | UNPRIMED (ORIGINAL LYRICS OPERA) | Cocky | Cocky | #N/A | #N/A | #N/A | UNPRIMED (ORIGINAL LYRICS FADO) | Sad | sad | 1.61 | 4.13 | 3.45 | UNPRIMED (ORIGINAL LYRICS HEAVY METAL) | angry | angry | 2.85 | 7.17 | 5.55 | UNPRIMED (ORIGINAL LYRICS HIP HOP) | Hurt | hurt | 1.9 | 5.85 | 3.33 | UNPRIMED (ORIGINAL LYRICS BOLERO) | Disappointed | Disappointed | 2.21 | 4.82 | 3.11 | UNPRIMED (ORIGINAL LYRICS BOLERO) | Afraid | Afraid | 2 | 6.67 | 3.98 | UNPRIMED (ORIGINAL LYRICS POP) | Energetic | energetic | 7.25 | 6.33 | 6.25 | UNPRIMED (ORIGINAL LYRICS SAMBA) | Sad | sad | 1.61 | 4.13 | 3.45 |  |  |
| SYDNEY | z5106796 |  | bouncy | passion | fun | anticipation | distressed | chill | fun | full | Slightly Agree | 5 | Strongly Disagree | 1 | Slightly Agree | 5 | Strongly Agree | 7 | Neither Agree nor Disagree | 4 | Moderately Agree | 6 | Slightly Agree | 5 | Slightly Agree | 5 | 5106796 | UNPRIMED (ORIGINAL LYRICS OPERA) | vain | vain | #N/A | #N/A | #N/A | UNPRIMED (ORIGINAL LYRICS FADO) | sadness | sadness | 2.21 | 5.21 | 2.82 | UNPRIMED (ORIGINAL LYRICS HEAVY METAL) | dispair | despair | 2.99 | 4.49 | 4.3 | UNPRIMED (ORIGINAL LYRICS HIP HOP) | j | j | #N/A | #N/A | #N/A | UNPRIMED (ORIGINAL LYRICS BOLERO) | passion | passion | 8.03 | 7.26 | 6.13 | UNPRIMED (ORIGINAL LYRICS BOLERO) | hope | Hope | 7.05 | 5.44 | 5.52 | UNPRIMED (ORIGINAL LYRICS POP) | boring | boring | 3.38 | 2.29 | 4.18 | UNPRIMED (ORIGINAL LYRICS SAMBA) | distress | distress | 2.67 | 5.7 | 3.03 |  |  |
| SYDNEY | Z5109946 |  | first one is relaxing  second one is happy | relaxing | pub music | relaxing | hype | hype | exciting | relaxing | Slightly Agree | 5 | Slightly Agree | 5 | Slightly Agree | 5 | Slightly Agree | 5 | Slightly Agree | 5 | Slightly Agree | 5 | Slightly Agree | 5 | Slightly Agree | 5 | 5109946 | UNPRIMED (ORIGINAL LYRICS OPERA) | uncomfortable | Uncomfortable | 2.97 | 6.06 | 3.42 | UNPRIMED (ORIGINAL LYRICS FADO) | sad | sad | 1.61 | 4.13 | 3.45 | UNPRIMED (ORIGINAL LYRICS HEAVY METAL) | angry | angry | 2.85 | 7.17 | 5.55 | UNPRIMED (ORIGINAL LYRICS HIP HOP) | sad | sad | 1.61 | 4.13 | 3.45 | UNPRIMED (ORIGINAL LYRICS BOLERO) | lovely | lovely | 7.17 | 4.87 | 5.56 | UNPRIMED (ORIGINAL LYRICS BOLERO) | spiritual | spirit | 7 | 5.56 | 5.82 | UNPRIMED (ORIGINAL LYRICS POP) | hype | hype | #N/A | #N/A | #N/A | UNPRIMED (ORIGINAL LYRICS SAMBA) | sweet | sweet | 7.64 | 5.96 | 5.36 |  |  |
| SYDNEY | z5112812 |  | It makes me want to dance. | Makes me feel sad. | Happy and want to dance. | I feel like I'm in a religious/culutural setting. | It makes me cringe. | I feel energetic and want to dance. | I feel relaxed and chilled from the first one. The second makes me want to dance. Reminds me of msuic for long car drives. | The first one is relaxing. The second one is a little exciting. | Slightly Agree | 5 | Moderately Disagree | 2 | Moderately Agree | 6 | Slightly Agree | 5 | Neither Agree nor Disagree | 4 | Slightly Agree | 5 | Neither Agree nor Disagree | 4 | Neither Agree nor Disagree | 4 | 5112812 |  |  | #N/A | #N/A | #N/A | #N/A |  |  | #N/A | #N/A | #N/A | #N/A |  |  | #N/A | #N/A | #N/A | #N/A |  |  | #N/A | #N/A | #N/A | #N/A |  |  | #N/A | #N/A | #N/A | #N/A |  |  | #N/A | #N/A | #N/A | #N/A |  |  | #N/A | #N/A | #N/A | #N/A |  |  | #N/A | #N/A | #N/A | #N/A |  |  |
| SYDNEY | 5113053 |  | Curious | Curious | Curious | Confused | Resistant | Interested | Calm | Serene | Strongly Disagree | 1 | Strongly Disagree | 1 | Slightly Disagree | 3 | Slightly Disagree | 3 | Strongly Disagree | 1 | Neither Agree nor Disagree | 4 | Strongly Disagree | 1 | Strongly Disagree | 1 | 5113053 | PRIMED POP (ORIGINAL LYRICS OPERA) | Curious | Curious | 6.08 | 5.82 | 5.42 | PRIMED OPERA (ORIGINAL LYRICS FADO) | Sad | sad | 1.61 | 4.13 | 3.45 | PRIMED CUBAN SON (ORIGINAL LYRICS HEAVY METAL) | Respect | Respect | 7.64 | 5.19 | 6.89 | PRIMED SAMBA (ORIGINAL LYRICS HIP HOP) | Curious | Curious | 6.08 | 5.82 | 5.42 | PRIMED KOTO (ORIGINAL LYRICS BOLERO) | Sad | sad | 1.61 | 4.13 | 3.45 | PRIMED HEAVY METAL (ORIGINAL LYRICS KOTO) | Curious | Curious | 6.08 | 5.82 | 5.42 | PRIMED FADO (ORIGINAL LYRICS POP) | Curious | Curious | 6.08 | 5.82 | 5.42 | PRIMED HIP HOP (ORIGINAL LYRICS SAMBA) | Curious | Curious | 6.08 | 5.82 | 5.42 |  |  |
| SYDNEY | 5113053 |  |  |  |  |  |  |  |  |  |  |  |  |  |  |  |  |  |  |  |  |  |  |  |  |  | 5113053 |  |  | #N/A | #N/A | #N/A | #N/A |  |  | #N/A | #N/A | #N/A | #N/A |  |  | #N/A | #N/A | #N/A | #N/A |  |  | #N/A | #N/A | #N/A | #N/A |  |  | #N/A | #N/A | #N/A | #N/A |  |  | #N/A | #N/A | #N/A | #N/A |  |  | #N/A | #N/A | #N/A | #N/A |  |  | #N/A | #N/A | #N/A | #N/A |  |  |
| SYDNEY | 5113053 |  |  |  |  |  |  |  |  |  |  |  |  |  |  |  |  |  |  |  |  |  |  |  |  |  | 5113053 |  |  | #N/A | #N/A | #N/A | #N/A |  |  | #N/A | #N/A | #N/A | #N/A |  |  | #N/A | #N/A | #N/A | #N/A |  |  | #N/A | #N/A | #N/A | #N/A |  |  | #N/A | #N/A | #N/A | #N/A |  |  | #N/A | #N/A | #N/A | #N/A |  |  | #N/A | #N/A | #N/A | #N/A |  |  | #N/A | #N/A | #N/A | #N/A |  |  |
| SYDNEY | 5113129 |  | walking through markets. cooking | relaxed | dancing | dreaming | fighting | cool | dancing | floating | Neither Agree nor Disagree | 4 | Moderately Disagree | 2 | Neither Agree nor Disagree | 4 | Strongly Agree | 7 | Slightly Disagree | 3 | Slightly Disagree | 3 | Neither Agree nor Disagree | 4 | Neither Agree nor Disagree | 4 | 5113129 |  |  | #N/A | #N/A | #N/A | #N/A |  |  | #N/A | #N/A | #N/A | #N/A |  |  | #N/A | #N/A | #N/A | #N/A |  |  | #N/A | #N/A | #N/A | #N/A |  |  | #N/A | #N/A | #N/A | #N/A |  |  | #N/A | #N/A | #N/A | #N/A |  |  | #N/A | #N/A | #N/A | #N/A |  |  | #N/A | #N/A | #N/A | #N/A |  |  |
| SYDNEY | z5113226 |  | zest | longing | sensuality | serenity | anger | vanity | elation | optimism | Moderately Disagree | 2 | Strongly Disagree | 1 | Slightly Agree | 5 | Neither Agree nor Disagree | 4 | Neither Agree nor Disagree | 4 | Slightly Agree | 5 | Moderately Disagree | 2 | Moderately Disagree | 2 | 5113226 | PRIMED OPERA (ORIGINAL LYRICS OPERA) | confused | confused | 3.21 | 6.03 | 4.24 | PRIMED FADO (ORIGINAL LYRICS FADO) | confused | confused | 3.21 | 6.03 | 4.24 | PRIMED HEAVY METAL (ORIGINAL LYRICS HEAVY METAL) | worried | worried | #N/A | #N/A | #N/A | PRIMED HIP HOP (ORIGINAL LYRICS HIP HOP) | rhyming | rhyming | #N/A | #N/A | #N/A | PRIMED BOLERO (ORIGINAL LYRICS BOLERO) | anguish | Anguish | #N/A | #N/A | #N/A | PRIMED KOTO (ORIGINAL LYRICS KOTO) | religious | religion | 5.07 | 5.85 | 5.3 | PRIMED POP (ORIGINAL LYRICS POP) | confused | confused | 3.21 | 6.03 | 4.24 | PRIMED SAMBA (ORIGINAL LYRICS SAMBA) | love | love | 8.72 | 6.44 | 7.11 |  |  |
| SYDNEY | 5114041 |  | chaotic | mellow | groovy | unfamiliar | active | active | excited | neutral | Neither Agree nor Disagree | 4 | Neither Agree nor Disagree | 4 | Strongly Agree | 7 | Strongly Agree | 7 | I don't know this music | 8 | Neither Agree nor Disagree | 4 | I don't know this music | 8 | I don't know this music | 8 | 5114041 | UNPRIMED (ORIGINAL LYRICS OPERA) | arrogant | arrogant | 3.69 | 5.65 | 5.14 | UNPRIMED (ORIGINAL LYRICS FADO) | tormented | tormented | #N/A | #N/A | #N/A | UNPRIMED (ORIGINAL LYRICS HEAVY METAL) | dread | dread | #N/A | #N/A | #N/A | UNPRIMED (ORIGINAL LYRICS HIP HOP) | desperation | despair | 2.99 | 4.49 | 4.3 | UNPRIMED (ORIGINAL LYRICS BOLERO) | betrayal | Betrayal | 2.28 | 5.37 | 4.18 | UNPRIMED (ORIGINAL LYRICS BOLERO) | hope | Hope | 7.05 | 5.44 | 5.52 | UNPRIMED (ORIGINAL LYRICS POP) | contempt | contempt | 3.85 | 5.28 | 5.13 | UNPRIMED (ORIGINAL LYRICS SAMBA) | hurt | hurt | 1.9 | 5.85 | 3.33 |  |  |
| SYDNEY | 5114049 |  |  |  |  |  |  |  |  |  |  |  |  |  |  |  |  |  |  |  |  |  |  |  |  |  | 5114049 |  |  | #N/A | #N/A | #N/A | #N/A |  |  | #N/A | #N/A | #N/A | #N/A |  |  | #N/A | #N/A | #N/A | #N/A |  |  | #N/A | #N/A | #N/A | #N/A |  |  | #N/A | #N/A | #N/A | #N/A |  |  | #N/A | #N/A | #N/A | #N/A |  |  | #N/A | #N/A | #N/A | #N/A |  |  | #N/A | #N/A | #N/A | #N/A |  |  |
| SYDNEY | z5115524 |  | Enlightened. | Light | Rumba | Uncertainty | Messy | Hyped | Neutral | Flow-ey | Moderately Agree | 6 | Strongly Disagree | 1 | Strongly Agree | 7 | Moderately Agree | 6 | Slightly Disagree | 3 | Neither Agree nor Disagree | 4 | Slightly Agree | 5 | Neither Agree nor Disagree | 4 | 5115524 | PRIMED POP (ORIGINAL LYRICS OPERA) | Interested | Interested | 6.58 | 5.42 | 5.95 | PRIMED OPERA (ORIGINAL LYRICS FADO) | Harmonious | harmony | 6.54 | 3.98 | 5.64 | PRIMED CUBAN SON (ORIGINAL LYRICS HEAVY METAL) | Inspiring | inspirational | 7.57 | 6.18 | 6.3 | PRIMED SAMBA (ORIGINAL LYRICS HIP HOP) | Empathy | empathy | 5.32 | 4.81 | 4.33 | PRIMED KOTO (ORIGINAL LYRICS BOLERO) | Heartfelt | Heartfelt | #N/A | #N/A | #N/A | PRIMED HEAVY METAL (ORIGINAL LYRICS KOTO) | Deep | Deep | 5.08 | 4.55 | 5.37 | PRIMED FADO (ORIGINAL LYRICS POP) | negative | negative | #N/A | #N/A | #N/A | PRIMED HIP HOP (ORIGINAL LYRICS SAMBA) | Cool | Cool | 6.83 | 4.17 | 5.97 |  |  |
| SYDNEY | z5115524 |  | Happy | Calm | Lively | Refined | Anger | Excited | Neutral | Majestic | Moderately Agree | 6 | Moderately Disagree | 2 | Strongly Agree | 7 | Moderately Agree | 6 | Slightly Agree | 5 | Moderately Agree | 6 | Neither Agree nor Disagree | 4 | Slightly Agree | 5 | 5115524 | PRIMED POP (ORIGINAL LYRICS OPERA) | Interested | Interested | 6.58 | 5.42 | 5.95 | PRIMED OPERA (ORIGINAL LYRICS FADO) | Harmonious | harmony | 6.54 | 3.98 | 5.64 | PRIMED CUBAN SON (ORIGINAL LYRICS HEAVY METAL) | Inspired | Inspired | 7.15 | 6.02 | 6.67 | PRIMED SAMBA (ORIGINAL LYRICS HIP HOP) | Empathy | empathy | 5.32 | 4.81 | 4.33 | PRIMED KOTO (ORIGINAL LYRICS BOLERO) | Heartfelt | Heartfelt | #N/A | #N/A | #N/A | PRIMED HEAVY METAL (ORIGINAL LYRICS KOTO) | Deep | Deep | 5.08 | 4.55 | 5.37 | PRIMED FADO (ORIGINAL LYRICS POP) | Negative | negative | #N/A | #N/A | #N/A | PRIMED HIP HOP (ORIGINAL LYRICS SAMBA) | Cool | Cool | 6.83 | 4.17 | 5.97 |  |  |
| SYDNEY | z5115779 |  | spirited | serene | soothed | Tranquil | tense | victorious | happy | uplifted | Slightly Agree | 5 | Strongly Disagree | 1 | Slightly Agree | 5 | Neither Agree nor Disagree | 4 | Moderately Agree | 6 | Slightly Agree | 5 | Slightly Agree | 5 | Slightly Agree | 5 | 5115779 |  |  | #N/A | #N/A | #N/A | #N/A |  |  | #N/A | #N/A | #N/A | #N/A |  |  | #N/A | #N/A | #N/A | #N/A |  |  | #N/A | #N/A | #N/A | #N/A |  |  | #N/A | #N/A | #N/A | #N/A |  |  | #N/A | #N/A | #N/A | #N/A |  |  | #N/A | #N/A | #N/A | #N/A |  |  | #N/A | #N/A | #N/A | #N/A |  |  |
| SYDNEY | z5116182 |  | dancey/pumped | mellow | energetic | mysterious | scared, anger | existential | pumped | excited | I don't know this music | 8 | Strongly Disagree | 1 | Moderately Agree | 6 | Strongly Agree | 7 | I don't know this music | 8 | I don't know this music | 8 | I don't know this music | 8 | I don't know this music | 8 | 5116182 | PRIMED OPERA (ORIGINAL LYRICS OPERA) | desired | desire | 7.69 | 7.35 | 6.49 | PRIMED FADO (ORIGINAL LYRICS FADO) | apprehensive | apprehensive | #N/A | #N/A | #N/A | PRIMED HEAVY METAL (ORIGINAL LYRICS HEAVY METAL) | uneasy | Uneasy | 3.07 | 6.03 | 3.24 | PRIMED HIP HOP (ORIGINAL LYRICS HIP HOP) | lost | lost | 2.82 | 5.82 | 2.86 | PRIMED BOLERO (ORIGINAL LYRICS BOLERO) | hurt | hurt | 1.9 | 5.85 | 3.33 | PRIMED KOTO (ORIGINAL LYRICS KOTO) | enlightened | enlightened | #N/A | #N/A | #N/A | PRIMED POP (ORIGINAL LYRICS POP) | discomfort | discomfort | 2.19 | 4.17 | 3.86 | PRIMED SAMBA (ORIGINAL LYRICS SAMBA) | pain | pain | 2.13 | 6.5 | 3.71 |  |  |
| SYDNEY | 5117762 |  | joy | nostalgic | Happiness | reserved | anger | chill | energetic | Inspired | Neither Agree nor Disagree | 4 | Strongly Disagree | 1 | Moderately Agree | 6 | Slightly Disagree | 3 | Neither Agree nor Disagree | 4 | Slightly Agree | 5 | Slightly Agree | 5 | Neither Agree nor Disagree | 4 | 5117762 | PRIMED POP (ORIGINAL LYRICS OPERA) | pride | Pride | 7 | 5.83 | 7.06 | PRIMED OPERA (ORIGINAL LYRICS FADO) | Pain | pain | 2.13 | 6.5 | 3.71 | PRIMED CUBAN SON (ORIGINAL LYRICS HEAVY METAL) | Regret | Regret | 2.26 | 5.67 | 3.23 | PRIMED SAMBA (ORIGINAL LYRICS HIP HOP) | Anguish | Anguish | #N/A | #N/A | #N/A | PRIMED KOTO (ORIGINAL LYRICS BOLERO) | hurt | hurt | 1.9 | 5.85 | 3.33 | PRIMED HEAVY METAL (ORIGINAL LYRICS KOTO) | hurt | hurt | 1.9 | 5.85 | 3.33 | PRIMED FADO (ORIGINAL LYRICS POP) | passion | passion | 8.03 | 7.26 | 6.13 | PRIMED HIP HOP (ORIGINAL LYRICS SAMBA) | Pain | pain | 2.13 | 6.5 | 3.71 |  |  |
|  |  |  |  |  |  |  |  |  |  |  |  |  |  |  |  |  |  |  |  |  |  |  |  |  |  |  | 5117762 |  |  | #N/A | #N/A | #N/A | #N/A |  |  | #N/A | #N/A | #N/A | #N/A |  |  | #N/A | #N/A | #N/A | #N/A |  |  | #N/A | #N/A | #N/A | #N/A |  |  | #N/A | #N/A | #N/A | #N/A |  |  | #N/A | #N/A | #N/A | #N/A |  |  | #N/A | #N/A | #N/A | #N/A |  |  | #N/A | #N/A | #N/A | #N/A |  |  |
| SYDNEY | 5117863 |  | intrigued | relaxed | excited | intrigued | scared | inspired | energetic | calm | Moderately Disagree | 2 | Moderately Disagree | 2 | Strongly Agree | 7 | Slightly Agree | 5 | Slightly Disagree | 3 | Slightly Disagree | 3 | Slightly Disagree | 3 | Slightly Disagree | 3 | 5117863 | UNPRIMED (ORIGINAL LYRICS OPERA) | confident | confident | 7.98 | 6.22 | 7.68 | UNPRIMED (ORIGINAL LYRICS FADO) | sad | sad | 1.61 | 4.13 | 3.45 | UNPRIMED (ORIGINAL LYRICS HEAVY METAL) | evil | evil | 3.23 | 6.39 | 5.25 | UNPRIMED (ORIGINAL LYRICS HIP HOP) | sad | sad | 1.61 | 4.13 | 3.45 | UNPRIMED (ORIGINAL LYRICS BOLERO) | betrayed | Betrayal | 2.28 | 5.37 | 4.18 | UNPRIMED (ORIGINAL LYRICS BOLERO) | intense | intense | 5.44 | 6.32 | 5.72 | UNPRIMED (ORIGINAL LYRICS POP) | angry | angry | 2.85 | 7.17 | 5.55 | UNPRIMED (ORIGINAL LYRICS SAMBA) | scared | scared | 2.78 | 6.82 | 2.94 |  |  |
| SYDNEY | 5118018 |  | Mild excitement | Sadness | Slight sadness | Calm | Anger | Emotionless | Calm | Anticipation | Strongly Disagree | 1 | Strongly Disagree | 1 | Strongly Disagree | 1 | Strongly Disagree | 1 | Strongly Disagree | 1 | Strongly Disagree | 1 | Strongly Disagree | 1 | Strongly Disagree | 1 | 5118018 | PRIMED POP (ORIGINAL LYRICS OPERA) | Security | Security | 7.28 | 4.22 | 5.53 | PRIMED OPERA (ORIGINAL LYRICS FADO) | Sadness | sadness | 2.21 | 5.21 | 2.82 | PRIMED CUBAN SON (ORIGINAL LYRICS HEAVY METAL) | Anger | anger | 2.34 | 7.63 | 5.5 | PRIMED SAMBA (ORIGINAL LYRICS HIP HOP) | Heartbreak | heartbreak | 1.93 | 5.8 | 3.11 | PRIMED KOTO (ORIGINAL LYRICS BOLERO) | Sadness | sadness | 2.21 | 5.21 | 2.82 | PRIMED HEAVY METAL (ORIGINAL LYRICS KOTO) | Anger | anger | 2.34 | 7.63 | 5.5 | PRIMED FADO (ORIGINAL LYRICS POP) | Anger | anger | 2.34 | 7.63 | 5.5 | PRIMED HIP HOP (ORIGINAL LYRICS SAMBA) | Conflicted | conflicted | #N/A | #N/A | #N/A |  |  |
| SYDNEY | 5118091 |  | cheerful | calm | joy | nervousness | Fear | disgust | Happy | Intense | I don't know this music | 8 | Strongly Disagree | 1 | Strongly Agree | 7 | Moderately Disagree | 2 | I don't know this music | 8 | Moderately Agree | 6 | I don't know this music | 8 | I don't know this music | 8 | 5118091 | PRIMED OPERA (ORIGINAL LYRICS OPERA) | conceited | conceited | 3.68 | 4.64 | 5.32 |  |  | #N/A | #N/A | #N/A | #N/A |  |  | #N/A | #N/A | #N/A | #N/A |  |  | #N/A | #N/A | #N/A | #N/A |  |  | #N/A | #N/A | #N/A | #N/A |  |  | #N/A | #N/A | #N/A | #N/A |  |  | #N/A | #N/A | #N/A | #N/A |  |  | #N/A | #N/A | #N/A | #N/A |  |  |
| SYDNEY | 5118393 |  | energetic | calm | calm | curious | scared | powerul | bright | calm | Moderately Agree | 6 | Strongly Disagree | 1 | Neither Agree nor Disagree | 4 | Strongly Agree | 7 | I don't know this music | 8 | Moderately Agree | 6 | Slightly Agree | 5 | Slightly Agree | 5 | 5118393 | PRIMED OPERA (ORIGINAL LYRICS OPERA) | vain | vain | #N/A | #N/A | #N/A | PRIMED FADO (ORIGINAL LYRICS FADO) | depression | depression | 1.85 | 4.54 | 2.91 | PRIMED HEAVY METAL (ORIGINAL LYRICS HEAVY METAL) | disgust | disgusting | 2.96 | 5.18 | 3.64 | PRIMED HIP HOP (ORIGINAL LYRICS HIP HOP) | uncaring | uncaring | #N/A | #N/A | #N/A | PRIMED BOLERO (ORIGINAL LYRICS BOLERO) | sadness | sadness | 2.21 | 5.21 | 2.82 | PRIMED KOTO (ORIGINAL LYRICS KOTO) | holy | holy | 6.36 | 5.36 | 4.68 | PRIMED POP (ORIGINAL LYRICS POP) | confusion | confusion | 3.46 | 6.07 | 3.04 | PRIMED SAMBA (ORIGINAL LYRICS SAMBA) | envy | envy | 3.41 | 5.5 | 4.13 |  |  |
| SYDNEY | z5118730 |  | Jolly | Love | Romantic | Calmness | Energy and excitement | Groovy | Casual | Immersiveness | Slightly Agree | 5 | Strongly Agree | 7 | Slightly Disagree | 3 | Strongly Agree | 7 | Strongly Agree | 7 | Strongly Agree | 7 | Neither Agree nor Disagree | 4 | Neither Agree nor Disagree | 4 | 5118730 | PRIMED POP (ORIGINAL LYRICS OPERA) | disgusted | disgusted | 2.45 | 5.42 | 4.34 | PRIMED OPERA (ORIGINAL LYRICS FADO) | Romantic | Romantic | 8.32 | 7.59 | 6.08 | PRIMED CUBAN SON (ORIGINAL LYRICS HEAVY METAL) | Surprise | Surprise | 7.73 | 7.07 | 3.87 | PRIMED SAMBA (ORIGINAL LYRICS HIP HOP) | sad | sad | 1.61 | 4.13 | 3.45 | PRIMED KOTO (ORIGINAL LYRICS BOLERO) | Sad | sad | 1.61 | 4.13 | 3.45 | PRIMED HEAVY METAL (ORIGINAL LYRICS KOTO) | Holy | holy | 6.36 | 5.36 | 4.68 | PRIMED FADO (ORIGINAL LYRICS POP) | Ironic | Ironic | #N/A | #N/A | #N/A | PRIMED HIP HOP (ORIGINAL LYRICS SAMBA) | longing | Longing | #N/A | #N/A | #N/A |  |  |
| SYDNEY | z5118803 |  | relaxed | Romantic | Passionate | Traditional | Noisy | Sleepy | Young | exciting | Neither Agree nor Disagree | 4 | Moderately Disagree | 2 | Moderately Agree | 6 | Moderately Disagree | 2 | Neither Agree nor Disagree | 4 | Strongly Agree | 7 | Slightly Agree | 5 | Slightly Agree | 5 | 5118803 | PRIMED OPERA (ORIGINAL LYRICS OPERA) | curious | Curious | 6.08 | 5.82 | 5.42 | PRIMED FADO (ORIGINAL LYRICS FADO) | tragic | tragic | #N/A | #N/A | #N/A | PRIMED HEAVY METAL (ORIGINAL LYRICS HEAVY METAL) | bullshit | bullshit | 4.14 | 5.15 | 5.29 | PRIMED HIP HOP (ORIGINAL LYRICS HIP HOP) | spiteful | spiteful | #N/A | #N/A | #N/A | PRIMED BOLERO (ORIGINAL LYRICS BOLERO) | betrayed | Betrayal | 2.28 | 5.37 | 4.18 | PRIMED KOTO (ORIGINAL LYRICS KOTO) | holy | holy | 6.36 | 5.36 | 4.68 | PRIMED POP (ORIGINAL LYRICS POP) | environmental | environmental | #N/A | #N/A | #N/A | PRIMED SAMBA (ORIGINAL LYRICS SAMBA) | love | love | 8.72 | 6.44 | 7.11 |  |  |
| SYDNEY | z5120364 |  | joy | teary | smiley | open | uncomfortable | chill | crowded | Romantic | Moderately Agree | 6 | Moderately Disagree | 2 | Slightly Agree | 5 | Moderately Agree | 6 | Slightly Disagree | 3 | Moderately Agree | 6 | Moderately Agree | 6 | Moderately Agree | 6 | 5120364 | UNPRIMED (ORIGINAL LYRICS OPERA) | discomfort | discomfort | 2.19 | 4.17 | 3.86 |  | confused | confused | 3.21 | 6.03 | 4.24 |  |  | #N/A | #N/A | #N/A | #N/A |  |  | #N/A | #N/A | #N/A | #N/A |  | love | love | 8.72 | 6.44 | 7.11 |  | avoidant | avoidant | #N/A | #N/A | #N/A |  |  | #N/A | #N/A | #N/A | #N/A |  | familiarity | familiarity | #N/A | #N/A | #N/A |  |  |
| SYDNEY | 5120615 |  | happy or energetic | dreamy | sensual | eerie | angry | tense | happy | relaxed | I don't know this music | 8 | Strongly Disagree | 1 | Strongly Agree | 7 | Slightly Agree | 5 | I don't know this music | 8 | Slightly Agree | 5 | I don't know this music | 8 | I don't know this music | 8 | 5120615 | PRIMED POP (ORIGINAL LYRICS OPERA) | carefree | carefree | 7.54 | 4.17 | 5.78 | PRIMED OPERA (ORIGINAL LYRICS FADO) | empowerment | power | 6.54 | 6.67 | 7.28 | PRIMED CUBAN SON (ORIGINAL LYRICS HEAVY METAL) | disgust | disgusting | 2.96 | 5.18 | 3.64 | PRIMED SAMBA (ORIGINAL LYRICS HIP HOP) | sad | sad | 1.61 | 4.13 | 3.45 | PRIMED KOTO (ORIGINAL LYRICS BOLERO) | sad | sad | 1.61 | 4.13 | 3.45 | PRIMED HEAVY METAL (ORIGINAL LYRICS KOTO) | disgust | disgusting | 2.96 | 5.18 | 3.64 | PRIMED FADO (ORIGINAL LYRICS POP) | angry | angry | 2.85 | 7.17 | 5.55 | PRIMED HIP HOP (ORIGINAL LYRICS SAMBA) | Emotional | Emotional | 4.36 | 5.75 | 4.29 |  |  |
| SYDNEY | z5120734 |  | Chill | Relaxed | Happy | cultured peaceful calm | Affronted | Chill and Cool | Bored and annoyed | Bored | Strongly Disagree | 1 | Strongly Disagree | 1 | Neither Agree nor Disagree | 4 | Neither Agree nor Disagree | 4 | Strongly Disagree | 1 | Strongly Disagree | 1 | Strongly Disagree | 1 | Strongly Disagree | 1 | 5120734 | UNPRIMED (ORIGINAL LYRICS OPERA) | Bragging | Bragging | #N/A | #N/A | #N/A | UNPRIMED (ORIGINAL LYRICS FADO) | Heavy | Heavy | 3.69 | 4.58 | 4.1 | UNPRIMED (ORIGINAL LYRICS HEAVY METAL) | confused | confused | 3.21 | 6.03 | 4.24 | UNPRIMED (ORIGINAL LYRICS HIP HOP) | Religious | religion | 5.07 | 5.85 | 5.3 | UNPRIMED (ORIGINAL LYRICS BOLERO) | Emo | emotional | 4.36 | 5.75 | 4.29 | UNPRIMED (ORIGINAL LYRICS BOLERO) | Religion | 0 | #N/A | #N/A | #N/A | UNPRIMED (ORIGINAL LYRICS POP) | disgust | disgusting | 2.96 | 5.18 | 3.64 | UNPRIMED (ORIGINAL LYRICS SAMBA) | Sad | sad | 1.61 | 4.13 | 3.45 |  |  |
| SYDNEY | 5123556 |  | s | d | g | a | z | f | b | f |  |  |  |  |  |  |  |  |  |  |  |  |  |  |  |  | 5123556 |  |  | #N/A | #N/A | #N/A | #N/A |  |  | #N/A | #N/A | #N/A | #N/A |  |  | #N/A | #N/A | #N/A | #N/A |  |  | #N/A | #N/A | #N/A | #N/A |  |  | #N/A | #N/A | #N/A | #N/A |  |  | #N/A | #N/A | #N/A | #N/A |  |  | #N/A | #N/A | #N/A | #N/A |  |  | #N/A | #N/A | #N/A | #N/A |  |  |
| SYDNEY | z5127289 |  | Enjoyment | Peace | Serenity | Confused | Annoyed | Joy | Elated | Anticipation | Slightly Agree | 5 | Strongly Disagree | 1 | Strongly Agree | 7 | Moderately Agree | 6 | I don't know this music | 8 | Neither Agree nor Disagree | 4 | I don't know this music | 8 | I don't know this music | 8 | 5127289 | PRIMED POP (ORIGINAL LYRICS OPERA) | Surprised | Surprised | 7.47 | 7.47 | 6.11 | PRIMED OPERA (ORIGINAL LYRICS FADO) | Sadness | sadness | 2.21 | 5.21 | 2.82 | PRIMED CUBAN SON (ORIGINAL LYRICS HEAVY METAL) | Disgust | disgusting | 2.96 | 5.18 | 3.64 | PRIMED SAMBA (ORIGINAL LYRICS HIP HOP) | Anticipation | anticipation | #N/A | #N/A | #N/A | PRIMED KOTO (ORIGINAL LYRICS BOLERO) | Disgust | disgusting | 2.96 | 5.18 | 3.64 | PRIMED HEAVY METAL (ORIGINAL LYRICS KOTO) | Interest | interest | 6.97 | 5.66 | 5.89 | PRIMED FADO (ORIGINAL LYRICS POP) | Confused | confused | 3.21 | 6.03 | 4.24 | PRIMED HIP HOP (ORIGINAL LYRICS SAMBA) | Fear | fear | 2.76 | 6.96 | 3.22 |  |  |
| SYDNEY | 5128426 |  | surprised | calm | oldies | annoyed | shock | annoyed | happy | soothing | Moderately Disagree | 2 | Moderately Disagree | 2 | Moderately Agree | 6 | Moderately Disagree | 2 | Moderately Disagree | 2 | Neither Agree nor Disagree | 4 | Moderately Disagree | 2 | Slightly Disagree | 3 | 5128426 | PRIMED OPERA (ORIGINAL LYRICS OPERA) | relax | relax | 7.87 | 2.47 | 6.37 | PRIMED FADO (ORIGINAL LYRICS FADO) | puzzled | puzzled | #N/A | #N/A | #N/A | PRIMED HEAVY METAL (ORIGINAL LYRICS HEAVY METAL) | annoyed | annoy | 2.96 | 5.52 | 4.44 | PRIMED HIP HOP (ORIGINAL LYRICS HIP HOP) | calm | calm | 6.73 | 3.6 | 6.37 | PRIMED BOLERO (ORIGINAL LYRICS BOLERO) | romantic | Romantic | 8.32 | 7.59 | 6.08 | PRIMED KOTO (ORIGINAL LYRICS KOTO) | puzzled | puzzled | #N/A | #N/A | #N/A | PRIMED POP (ORIGINAL LYRICS POP) | funny | Funny | 8.56 | 7 | 6.15 | PRIMED SAMBA (ORIGINAL LYRICS SAMBA) | calm | calm | 6.73 | 3.6 | 6.37 |  |  |
| SYDNEY | 5129603 |  | Dramatic | Soothing | Mysterious | Relaxing | Angry | Confidence. | Cool | Grand | Moderately Disagree | 2 | Strongly Disagree | 1 | Strongly Agree | 7 | Strongly Agree | 7 | Moderately Disagree | 2 | Slightly Agree | 5 | Moderately Disagree | 2 | Moderately Disagree | 2 | 5129603 | UNPRIMED (ORIGINAL LYRICS OPERA) | Lust | lust | 7.12 | 6.88 | 5.49 | UNPRIMED (ORIGINAL LYRICS FADO) | Sadness | sadness | 2.21 | 5.21 | 2.82 | UNPRIMED (ORIGINAL LYRICS HEAVY METAL) | Death | Death | 1.61 | 4.59 | 3.47 | UNPRIMED (ORIGINAL LYRICS HIP HOP) | Betrayal | Betrayal | 2.28 | 5.37 | 4.18 | UNPRIMED (ORIGINAL LYRICS BOLERO) | Pain | pain | 2.13 | 6.5 | 3.71 | UNPRIMED (ORIGINAL LYRICS BOLERO) | Forgiveness | Forgiveness | #N/A | #N/A | #N/A | UNPRIMED (ORIGINAL LYRICS POP) | Anger | anger | 2.34 | 7.63 | 5.5 | UNPRIMED (ORIGINAL LYRICS SAMBA) | Betrayal | Betrayal | 2.28 | 5.37 | 4.18 |  |  |
| SYDNEY | 5129658 |  | Happy | Calm | Blah | Unsettled | Aggressive | Bumping | Happy | Magical | Moderately Disagree | 2 | Strongly Disagree | 1 | Strongly Agree | 7 | Strongly Disagree | 1 | Slightly Agree | 5 | Moderately Agree | 6 | Strongly Disagree | 1 | Strongly Disagree | 1 | 5129658 | UNPRIMED (ORIGINAL LYRICS OPERA) | Sexy | Sexy | 8.02 | 7.36 | 6.82 | UNPRIMED (ORIGINAL LYRICS FADO) | Sad | sad | 1.61 | 4.13 | 3.45 | UNPRIMED (ORIGINAL LYRICS HEAVY METAL) | Agony | agony | 2.43 | 6.06 | 4.02 | UNPRIMED (ORIGINAL LYRICS HIP HOP) | Heartbreak | heartbreak | 1.93 | 5.8 | 3.11 | UNPRIMED (ORIGINAL LYRICS BOLERO) | Sad | sad | 1.61 | 4.13 | 3.45 | UNPRIMED (ORIGINAL LYRICS BOLERO) | Hopeful | Hopeful | 7.1 | 5.78 | 5.41 | UNPRIMED (ORIGINAL LYRICS POP) | Craving | Craving | #N/A | #N/A | #N/A | UNPRIMED (ORIGINAL LYRICS SAMBA) | Sadness | sadness | 2.21 | 5.21 | 2.82 |  |  |
| SYDNEY | z5129670 |  | excitement | calmness | dance | curiosity | unrest | energy | hapiness | nostalgia | Slightly Disagree | 3 | Strongly Disagree | 1 | Moderately Agree | 6 | Strongly Agree | 7 | I don't know this music | 8 | Moderately Agree | 6 | I don't know this music | 8 | Neither Agree nor Disagree | 4 | 5129670 |  |  | #N/A | #N/A | #N/A | #N/A |  |  | #N/A | #N/A | #N/A | #N/A |  |  | #N/A | #N/A | #N/A | #N/A |  |  | #N/A | #N/A | #N/A | #N/A |  |  | #N/A | #N/A | #N/A | #N/A |  |  | #N/A | #N/A | #N/A | #N/A |  |  | #N/A | #N/A | #N/A | #N/A |  |  | #N/A | #N/A | #N/A | #N/A |  |  |
| SYDNEY | z5129813 |  | relaxed | relaxed | content | Naivity | anger | happy | energised | determination | Slightly Agree | 5 | Strongly Disagree | 1 | Slightly Agree | 5 | Strongly Agree | 7 | Slightly Agree | 5 | Slightly Agree | 5 | Slightly Agree | 5 | Slightly Agree | 5 | 5129813 | PRIMED POP (ORIGINAL LYRICS OPERA) | | #N/A | #N/A | #N/A | #N/A | PRIMED OPERA (ORIGINAL LYRICS FADO) | | #N/A | #N/A | #N/A | #N/A | PRIMED OPERA (ORIGINAL LYRICS FADO) | dark | Dark | 4.71 | 4.28 | 4.84 | PRIMED SAMBA (ORIGINAL LYRICS HIP HOP) | | #N/A | #N/A | #N/A | #N/A | PRIMED KOTO (ORIGINAL LYRICS BOLERO) | religion | 0 | #N/A | #N/A | #N/A | PRIMED HEAVY METAL (ORIGINAL LYRICS KOTO) | anger | anger | 2.34 | 7.63 | 5.5 | PRIMED FADO (ORIGINAL LYRICS POP) | | #N/A | #N/A | #N/A | #N/A | PRIMED HIP HOP (ORIGINAL LYRICS SAMBA) | depression | depression | 1.85 | 4.54 | 2.91 |  |  |
| SYDNEY | z5129813 |  | relaxed | relaxed | content | Naivity | anger | happy | energised | determination | Slightly Agree | 5 | Strongly Disagree | 1 | Slightly Agree | 5 | Strongly Agree | 7 | Slightly Agree | 5 | Slightly Agree | 5 | Slightly Agree | 5 | Slightly Agree | 5 | 5129813 |  |  | #N/A | #N/A | #N/A | #N/A |  |  | #N/A | #N/A | #N/A | #N/A |  |  | #N/A | #N/A | #N/A | #N/A |  |  | #N/A | #N/A | #N/A | #N/A |  |  | #N/A | #N/A | #N/A | #N/A |  |  | #N/A | #N/A | #N/A | #N/A |  |  | #N/A | #N/A | #N/A | #N/A |  |  | #N/A | #N/A | #N/A | #N/A |  |  |
| SYDNEY | z5133205 |  | sleepy | boring | funny | peaceful | joyful | relaxing | happy | relaxing | Moderately Disagree | 2 | Slightly Agree | 5 | Moderately Agree | 6 | Moderately Agree | 6 | Neither Agree nor Disagree | 4 | Slightly Agree | 5 | Slightly Agree | 5 | Neither Agree nor Disagree | 4 | 5133205 | PRIMED OPERA (ORIGINAL LYRICS OPERA) | romantic | Romantic | 8.32 | 7.59 | 6.08 | PRIMED FADO (ORIGINAL LYRICS FADO) | boring | boring | 3.38 | 2.29 | 4.18 | PRIMED HEAVY METAL (ORIGINAL LYRICS HEAVY METAL) | distress | distress | 2.67 | 5.7 | 3.03 | PRIMED HIP HOP (ORIGINAL LYRICS HIP HOP) | joyful | joyful | 8.22 | 5.98 | 6.6 | PRIMED BOLERO (ORIGINAL LYRICS BOLERO) | boring | boring | 3.38 | 2.29 | 4.18 | PRIMED KOTO (ORIGINAL LYRICS KOTO) | boring | boring | 3.38 | 2.29 | 4.18 | PRIMED POP (ORIGINAL LYRICS POP) | excited | excitement | 7.5 | 7.67 | 6.18 | PRIMED SAMBA (ORIGINAL LYRICS SAMBA) | sleepy | sleepy | #N/A | #N/A | #N/A |  |  |
| SYDNEY | z5150292 |  | Relaxed | Calmness | Relaxed | Curiosity | Anger | Confidence | Boredom | Elevation | Strongly Disagree | 1 | Slightly Disagree | 3 | Slightly Disagree | 3 | Moderately Agree | 6 | Strongly Disagree | 1 | Moderately Agree | 6 | Strongly Disagree | 1 | Strongly Disagree | 1 | 5150292 | PRIMED OPERA (ORIGINAL LYRICS OPERA) | Haughty | Haughty | #N/A | #N/A | #N/A | PRIMED FADO (ORIGINAL LYRICS FADO) | Sadness | sadness | 2.21 | 5.21 | 2.82 | PRIMED HEAVY METAL (ORIGINAL LYRICS HEAVY METAL) | energetic | energetic | 7.25 | 6.33 | 6.25 | PRIMED HIP HOP (ORIGINAL LYRICS HIP HOP) | Loneliness | loneliness | 1.61 | 4.56 | 2.51 | PRIMED BOLERO (ORIGINAL LYRICS BOLERO) | Incomprehension | Incomprehension | #N/A | #N/A | #N/A | PRIMED KOTO (ORIGINAL LYRICS KOTO) | Superstitious | Superstitious | #N/A | #N/A | #N/A | PRIMED POP (ORIGINAL LYRICS POP) | Amusement | amusement | 7.96 | 6.22 | 5.74 | PRIMED SAMBA (ORIGINAL LYRICS SAMBA) | Sadness | sadness | 2.21 | 5.21 | 2.82 |  |  |
| SYDNEY | z5150386 |  | excited | happy | excitement | It makes me laugh, can't take this seriously | irritation | I am getting pumped up | happy | sleepy | Neither Agree nor Disagree | 4 | Moderately Disagree | 2 | Slightly Agree | 5 | Slightly Agree | 5 | Neither Agree nor Disagree | 4 | Slightly Agree | 5 | Neither Agree nor Disagree | 4 | Neither Agree nor Disagree | 4 | 5150386 | PRIMED OPERA (ORIGINAL LYRICS OPERA) | arrogant | arrogant | 3.69 | 5.65 | 5.14 | PRIMED FADO (ORIGINAL LYRICS FADO) | sadness | sadness | 2.21 | 5.21 | 2.82 | PRIMED HEAVY METAL (ORIGINAL LYRICS HEAVY METAL) | Insanity | Insanity | #N/A | #N/A | #N/A | PRIMED HIP HOP (ORIGINAL LYRICS HIP HOP) | hopelessness | hopeless | 2.27 | 4.28 | 2.96 | PRIMED BOLERO (ORIGINAL LYRICS BOLERO) | dissapointed | disappointment | 2.37 | 4.6 | 3.2 | PRIMED KOTO (ORIGINAL LYRICS KOTO) | neutral | Neutral | #N/A | #N/A | #N/A | PRIMED POP (ORIGINAL LYRICS POP) | excited | excitement | 7.5 | 7.67 | 6.18 | PRIMED SAMBA (ORIGINAL LYRICS SAMBA) | lonelyness | loneliness | 1.61 | 4.56 | 2.51 |  |  |
| SYDNEY | z5150386 |  | excited | sadness | happiness | weirdness | disgust | frustration | excitement | apprehensive | I don't know this music | 8 | Strongly Disagree | 1 | Slightly Agree | 5 | Slightly Agree | 5 | I don't know this music | 8 | Slightly Agree | 5 | Moderately Disagree | 2 | I don't know this music | 8 | 5150386 |  |  | #N/A | #N/A | #N/A | #N/A |  |  | #N/A | #N/A | #N/A | #N/A |  | darkness | darkness | #N/A | #N/A | #N/A |  |  | #N/A | #N/A | #N/A | #N/A |  |  | #N/A | #N/A | #N/A | #N/A |  |  | #N/A | #N/A | #N/A | #N/A |  |  | #N/A | #N/A | #N/A | #N/A |  |  | #N/A | #N/A | #N/A | #N/A |  |  |
| SYDNEY | z5150388 |  | Pleasant | Sadness | Happpiness | Tense | Frustration | Excited | Relaxed | Contented | Slightly Disagree | 3 | Moderately Disagree | 2 | Slightly Agree | 5 | Moderately Agree | 6 | Moderately Disagree | 2 | Slightly Agree | 5 | Neither Agree nor Disagree | 4 | Slightly Agree | 5 | 5150388 | UNPRIMED (ORIGINAL LYRICS OPERA) | Confidence | Confidence | 7.04 | 5.52 | 6.42 | UNPRIMED (ORIGINAL LYRICS FADO) | Fear | fear | 2.76 | 6.96 | 3.22 | UNPRIMED (ORIGINAL LYRICS HEAVY METAL) | Disgust | disgusting | 2.96 | 5.18 | 3.64 | UNPRIMED (ORIGINAL LYRICS HIP HOP) | Dissapointment | disappointment | 2.37 | 4.6 | 3.2 | UNPRIMED (ORIGINAL LYRICS BOLERO) | Upset | upset | 2 | 5.86 | 4.08 | UNPRIMED (ORIGINAL LYRICS BOLERO) | Shame | shame | 2.13 | 6.33 | 2.97 | UNPRIMED (ORIGINAL LYRICS POP) | Anger | anger | 2.34 | 7.63 | 5.5 | UNPRIMED (ORIGINAL LYRICS SAMBA) | Sadness | sadness | 2.21 | 5.21 | 2.82 |  |  |
| SYDNEY | z5150446 |  | Happy | Calm | Exotic | Pensive | Angry | Excited | Active | Historical (if that makes sense) | Neither Agree nor Disagree | 4 | Strongly Disagree | 1 | Moderately Agree | 6 | Moderately Agree | 6 | I don't know this music | 8 | Slightly Agree | 5 | I don't know this music | 8 | I don't know this music | 8 | 5150446 | PRIMED OPERA (ORIGINAL LYRICS OPERA) | Desire | Desire | 7.69 | 7.35 | 6.49 | PRIMED FADO (ORIGINAL LYRICS FADO) | Sadness | sadness | 2.21 | 5.21 | 2.82 | PRIMED HEAVY METAL (ORIGINAL LYRICS HEAVY METAL) | Rage | rage | 2.41 | 8.17 | 5.68 | PRIMED HIP HOP (ORIGINAL LYRICS HIP HOP) | Betrayal | Betrayal | 2.28 | 5.37 | 4.18 | PRIMED BOLERO (ORIGINAL LYRICS BOLERO) | Passion | passion | 8.03 | 7.26 | 6.13 | PRIMED KOTO (ORIGINAL LYRICS KOTO) | Confusion | confusion | 3.46 | 6.07 | 3.04 | PRIMED POP (ORIGINAL LYRICS POP) | Aggression | aggression | 3.54 | 5.73 | 5.04 | PRIMED SAMBA (ORIGINAL LYRICS SAMBA) | Fear | fear | 2.76 | 6.96 | 3.22 |  |  |
| SYDNEY | 5150502 |  | joyful | indifferent | upbeat | peaceful | dislike | neutral, don't necessarily enjoy | happy | content | Neither Agree nor Disagree | 4 | Moderately Disagree | 2 | Strongly Agree | 7 | Slightly Agree | 5 | Neither Agree nor Disagree | 4 | Slightly Agree | 5 | Neither Agree nor Disagree | 4 | Neither Agree nor Disagree | 4 | 5150502 | PRIMED POP (ORIGINAL LYRICS OPERA) | selfishness | selfish | 2.42 | 5.5 | 4.64 | PRIMED OPERA (ORIGINAL LYRICS FADO) | indifferent | indifferent | 4.61 | 3.18 | 4.84 | PRIMED CUBAN SON (ORIGINAL LYRICS HEAVY METAL) | uncomfortable | Uncomfortable | 2.97 | 6.06 | 3.42 | PRIMED SAMBA (ORIGINAL LYRICS HIP HOP) | uncomfortable | Uncomfortable | 2.97 | 6.06 | 3.42 | PRIMED KOTO (ORIGINAL LYRICS BOLERO) | sad | sad | 1.61 | 4.13 | 3.45 | PRIMED HEAVY METAL (ORIGINAL LYRICS KOTO) | uncomfortable | Uncomfortable | 2.97 | 6.06 | 3.42 | PRIMED FADO (ORIGINAL LYRICS POP) | uncomfortable | Uncomfortable | 2.97 | 6.06 | 3.42 | PRIMED HIP HOP (ORIGINAL LYRICS SAMBA) | sad | sad | 1.61 | 4.13 | 3.45 |  |  |
| SYDNEY | 5150512 |  | Relaxed | Solemn | Romantic | Unsettled | Angry | Confident | Joyful | confident | Slightly Disagree | 3 | Moderately Disagree | 2 | Moderately Agree | 6 | Moderately Agree | 6 | Slightly Disagree | 3 | Neither Agree nor Disagree | 4 | Neither Agree nor Disagree | 4 | Slightly Agree | 5 | 5150512 | PRIMED POP (ORIGINAL LYRICS OPERA) | Afraid | Afraid | 2 | 6.67 | 3.98 | PRIMED OPERA (ORIGINAL LYRICS FADO) | Bittersweet | Bittersweet | #N/A | #N/A | #N/A | PRIMED CUBAN SON (ORIGINAL LYRICS HEAVY METAL) | Hate | Hate | 2.12 | 6.95 | 5.05 | PRIMED SAMBA (ORIGINAL LYRICS HIP HOP) | Betrayed | Betrayal | 2.28 | 5.37 | 4.18 | PRIMED KOTO (ORIGINAL LYRICS BOLERO) | Forlorn | Forlorn | #N/A | #N/A | #N/A | PRIMED HEAVY METAL (ORIGINAL LYRICS KOTO) | Pious | Pious | #N/A | #N/A | #N/A | PRIMED FADO (ORIGINAL LYRICS POP) | Rebellious | rebellious | 4.86 | 5.82 | 6.28 | PRIMED HIP HOP (ORIGINAL LYRICS SAMBA) | Distraught | Distraught | #N/A | #N/A | #N/A |  |  |
| SYDNEY | 5150512 |  | Relaxed | Solemn | Romantic | Unsettled | Angry | Confident | Joyful | confident | Slightly Disagree | 3 | Moderately Disagree | 2 | Moderately Agree | 6 | Moderately Agree | 6 | Slightly Disagree | 3 | Neither Agree nor Disagree | 4 | Neither Agree nor Disagree | 4 | Slightly Agree | 5 | 5150512 |  |  | #N/A | #N/A | #N/A | #N/A |  |  | #N/A | #N/A | #N/A | #N/A |  |  | #N/A | #N/A | #N/A | #N/A |  |  | #N/A | #N/A | #N/A | #N/A |  |  | #N/A | #N/A | #N/A | #N/A |  |  | #N/A | #N/A | #N/A | #N/A |  |  | #N/A | #N/A | #N/A | #N/A |  |  | #N/A | #N/A | #N/A | #N/A |  |  |
| SYDNEY | 5150660 |  | Loud | Peaceful | Foreign | Confused | Annoyed | Gangster | I get excited | Movie music | Strongly Disagree | 1 | Strongly Disagree | 1 | Moderately Agree | 6 | Neither Agree nor Disagree | 4 | Strongly Disagree | 1 | Slightly Agree | 5 | Strongly Disagree | 1 | Strongly Disagree | 1 | 5150660 | UNPRIMED (ORIGINAL LYRICS OPERA) | Jealousy | Jealousy | 2.51 | 6.36 | 3.8 | UNPRIMED (ORIGINAL LYRICS FADO) | sad | sad | 1.61 | 4.13 | 3.45 | UNPRIMED (ORIGINAL LYRICS HEAVY METAL) | Dark | Dark | 4.71 | 4.28 | 4.84 | UNPRIMED (ORIGINAL LYRICS HIP HOP) | Religious | religion | 5.07 | 5.85 | 5.3 | UNPRIMED (ORIGINAL LYRICS BOLERO) | Dramatic | Dramatic | #N/A | #N/A | #N/A | UNPRIMED (ORIGINAL LYRICS BOLERO) | Deep | Deep | 5.08 | 4.55 | 5.37 | UNPRIMED (ORIGINAL LYRICS POP) | Stupid | stupid | 2.31 | 4.72 | 2.98 | UNPRIMED (ORIGINAL LYRICS SAMBA) | love | love | 8.72 | 6.44 | 7.11 |  |  |
| SYDNEY | z5151391 |  | Relaxed,positive | Feels like I just broke up | Calming | Uncomfortable | Awoken | Pumped | groovy | feels like I am watching a royal gathering | Neither Agree nor Disagree | 4 | Neither Agree nor Disagree | 4 | Slightly Agree | 5 | I don't know this music | 8 | Strongly Disagree | 1 | Slightly Disagree | 3 | Slightly Disagree | 3 | Neither Agree nor Disagree | 4 | 5151391 | PRIMED POP (ORIGINAL LYRICS OPERA) | confident | confident | 7.98 | 6.22 | 7.68 | PRIMED OPERA (ORIGINAL LYRICS FADO) | Like I am sitting at a patriotic gathering | Like I am sitting at a patriotic gathering | #N/A | #N/A | #N/A | PRIMED CUBAN SON (ORIGINAL LYRICS HEAVY METAL) | wow | wow | #N/A | #N/A | #N/A | PRIMED SAMBA (ORIGINAL LYRICS HIP HOP) | nothing | nothing | #N/A | #N/A | #N/A | PRIMED KOTO (ORIGINAL LYRICS BOLERO) | confused | confused | 3.21 | 6.03 | 4.24 | PRIMED HEAVY METAL (ORIGINAL LYRICS KOTO) | Like I am at church | Like I am at church | #N/A | #N/A | #N/A | PRIMED FADO (ORIGINAL LYRICS POP) | reggae-ish | reggae-ish | #N/A | #N/A | #N/A | PRIMED HIP HOP (ORIGINAL LYRICS SAMBA) | deep | Deep | 5.08 | 4.55 | 5.37 |  |  |
| SYDNEY | 5151679 |  | cheerful | calm | energized | annoyance | scared | aggressive | energized | content | Neither Agree nor Disagree | 4 | Strongly Disagree | 1 | Slightly Agree | 5 | Slightly Agree | 5 | Strongly Disagree | 1 | Slightly Disagree | 3 | Slightly Disagree | 3 | Slightly Disagree | 3 | 5151679 | UNPRIMED (ORIGINAL LYRICS OPERA) | content | content | #N/A | #N/A | #N/A |  |  | #N/A | #N/A | #N/A | #N/A |  | disturbed | disturbed | #N/A | #N/A | #N/A |  |  | #N/A | #N/A | #N/A | #N/A |  |  | #N/A | #N/A | #N/A | #N/A |  |  | #N/A | #N/A | #N/A | #N/A |  |  | #N/A | #N/A | #N/A | #N/A |  |  | #N/A | #N/A | #N/A | #N/A |  |  |
| SYDNEY | 5151679 |  |  |  |  |  |  |  | bouncy |  |  |  |  |  |  |  |  |  |  |  |  |  |  |  |  |  | 5151679 | UNPRIMED (ORIGINAL LYRICS OPERA) | content | content | #N/A | #N/A | #N/A |  |  | #N/A | #N/A | #N/A | #N/A |  | disturbed | disturbed | #N/A | #N/A | #N/A |  |  | #N/A | #N/A | #N/A | #N/A |  |  | #N/A | #N/A | #N/A | #N/A |  |  | #N/A | #N/A | #N/A | #N/A |  |  | #N/A | #N/A | #N/A | #N/A |  |  | #N/A | #N/A | #N/A | #N/A |  |  |
| SYDNEY | z5151698 |  | calm | tranquility | amused | amused | off-guard | excited | happy | indifferent | Moderately Disagree | 2 | Strongly Disagree | 1 | Strongly Agree | 7 | Strongly Agree | 7 | Strongly Disagree | 1 | Strongly Disagree | 1 | I don't know this music | 8 | Slightly Agree | 5 | 5151698 | PRIMED OPERA (ORIGINAL LYRICS OPERA) | happy | happy | 8.21 | 6.49 | 6.63 | PRIMED FADO (ORIGINAL LYRICS FADO) | sad | sad | 1.61 | 4.13 | 3.45 | PRIMED HEAVY METAL (ORIGINAL LYRICS HEAVY METAL) | disgust | disgusting | 2.96 | 5.18 | 3.64 | PRIMED HIP HOP (ORIGINAL LYRICS HIP HOP) | confused | confused | 3.21 | 6.03 | 4.24 | PRIMED BOLERO (ORIGINAL LYRICS BOLERO) | sad | sad | 1.61 | 4.13 | 3.45 | PRIMED KOTO (ORIGINAL LYRICS KOTO) | unhappy | unhappy | 1.57 | 4.18 | 3.34 | PRIMED POP (ORIGINAL LYRICS POP) | disgust | disgusting | 2.96 | 5.18 | 3.64 | PRIMED SAMBA (ORIGINAL LYRICS SAMBA) | sad | sad | 1.61 | 4.13 | 3.45 |  |  |
| SYDNEY | 5151786 |  | Normal | Relaxed | Normal | Strange | Uncomfortable | Normal | Normal | Normal | Strongly Disagree | 1 | Strongly Disagree | 1 | Strongly Agree | 7 | Slightly Disagree | 3 | Moderately Disagree | 2 | Neither Agree nor Disagree | 4 | Slightly Disagree | 3 | Moderately Disagree | 2 | 5151786 | PRIMED POP (ORIGINAL LYRICS OPERA) | Normal | Normal | #N/A | #N/A | #N/A | PRIMED OPERA (ORIGINAL LYRICS FADO) | Strange | strange | 4.79 | 5.09 | 5.25 | PRIMED CUBAN SON (ORIGINAL LYRICS HEAVY METAL) | Scared | scared | 2.78 | 6.82 | 2.94 | PRIMED SAMBA (ORIGINAL LYRICS HIP HOP) | Normal | Normal | #N/A | #N/A | #N/A | PRIMED KOTO (ORIGINAL LYRICS BOLERO) | Normal | Normal | #N/A | #N/A | #N/A | PRIMED HEAVY METAL (ORIGINAL LYRICS KOTO) | Normal | Normal | #N/A | #N/A | #N/A | PRIMED FADO (ORIGINAL LYRICS POP) | Strange | strange | 4.79 | 5.09 | 5.25 | PRIMED HIP HOP (ORIGINAL LYRICS SAMBA) | Empathetic | empathy | 5.32 | 4.81 | 4.33 |  |  |
| SYDNEY | 5153368 |  | Happy | Calm | Relaxed | Traditional | Angry | Rebellious | Relaxed | Royal | Moderately Agree | 6 | Moderately Disagree | 2 | Strongly Agree | 7 | Strongly Agree | 7 | Slightly Disagree | 3 | Moderately Disagree | 2 | Slightly Agree | 5 | Slightly Agree | 5 | 5153368 | PRIMED POP (ORIGINAL LYRICS OPERA) | Romance | Romance | 7.61 | 6.9 | 6.03 | PRIMED OPERA (ORIGINAL LYRICS FADO) | Pitiful | pity | 3.37 | 3.72 | 4.12 | PRIMED CUBAN SON (ORIGINAL LYRICS HEAVY METAL) | Dark | Dark | 4.71 | 4.28 | 4.84 | PRIMED SAMBA (ORIGINAL LYRICS HIP HOP) | Sadness | sadness | 2.21 | 5.21 | 2.82 | PRIMED KOTO (ORIGINAL LYRICS BOLERO) | Sadness | sadness | 2.21 | 5.21 | 2.82 | PRIMED HEAVY METAL (ORIGINAL LYRICS KOTO) | Inspired | Inspired | 7.15 | 6.02 | 6.67 | PRIMED FADO (ORIGINAL LYRICS POP) | Rebellious | rebellious | 4.86 | 5.82 | 6.28 | PRIMED HIP HOP (ORIGINAL LYRICS SAMBA) | Relatable | Relatable | #N/A | #N/A | #N/A |  |  |
| SYDNEY | z5153675 |  | Excited | Peaceful | Indifference | Worried | Scared | Cool | Chill | Calm | Moderately Disagree | 2 | Strongly Disagree | 1 | Strongly Agree | 7 | Moderately Agree | 6 | Strongly Disagree | 1 | Moderately Agree | 6 | Strongly Disagree | 1 | Strongly Disagree | 1 | 5153675 | UNPRIMED (ORIGINAL LYRICS OPERA) | Violated | Violated | #N/A | #N/A | #N/A | UNPRIMED (ORIGINAL LYRICS FADO) | Sad | sad | 1.61 | 4.13 | 3.45 | UNPRIMED (ORIGINAL LYRICS HEAVY METAL) | Scared | scared | 2.78 | 6.82 | 2.94 | UNPRIMED (ORIGINAL LYRICS HIP HOP) | Angst | Angst | #N/A | #N/A | #N/A | UNPRIMED (ORIGINAL LYRICS BOLERO) | Hurt | hurt | 1.9 | 5.85 | 3.33 | UNPRIMED (ORIGINAL LYRICS BOLERO) | Concern | Concern | #N/A | #N/A | #N/A | UNPRIMED (ORIGINAL LYRICS POP) | Mad | mad | 2.44 | 6.76 | 5.86 | UNPRIMED (ORIGINAL LYRICS SAMBA) | Insecure | insecure | 2.36 | 5.56 | 2.33 |  |  |
| SYDNEY | 5153683 |  | dancey | relaxed | hungry | relaxed | scared | chilled | excited | happy | Strongly Disagree | 1 | Strongly Disagree | 1 | Strongly Agree | 7 | Strongly Agree | 7 | Strongly Disagree | 1 | Slightly Agree | 5 | Strongly Disagree | 1 | Strongly Disagree | 1 | 5153683 | UNPRIMED (ORIGINAL LYRICS OPERA) | indifference | indifference | #N/A | #N/A | #N/A | UNPRIMED (ORIGINAL LYRICS FADO) | upset | upset | 2 | 5.86 | 4.08 | UNPRIMED (ORIGINAL LYRICS HEAVY METAL) | uncomfortable | Uncomfortable | 2.97 | 6.06 | 3.42 | UNPRIMED (ORIGINAL LYRICS HIP HOP) | disappointment | Disappointment | 2.37 | 4.6 | 3.2 | UNPRIMED (ORIGINAL LYRICS BOLERO) | sad | sad | 1.61 | 4.13 | 3.45 | UNPRIMED (ORIGINAL LYRICS BOLERO) | confused | confused | 3.21 | 6.03 | 4.24 | UNPRIMED (ORIGINAL LYRICS POP) | empowered | power | 6.54 | 6.67 | 7.28 | UNPRIMED (ORIGINAL LYRICS SAMBA) | longing | Longing | #N/A | #N/A | #N/A |  |  |
| SYDNEY | 5154954 |  | straight | great | great | crazy | noise | great | good | wonderful | Neither Agree nor Disagree | 4 | Slightly Disagree | 3 | Strongly Agree | 7 | Moderately Agree | 6 | Slightly Agree | 5 | Strongly Agree | 7 | Neither Agree nor Disagree | 4 | Neither Agree nor Disagree | 4 | 5154954 | PRIMED OPERA (ORIGINAL LYRICS OPERA) | nice | nice | 6.55 | 4.38 | 5.58 | PRIMED FADO (ORIGINAL LYRICS FADO) | sad | sad | 1.61 | 4.13 | 3.45 | PRIMED HEAVY METAL (ORIGINAL LYRICS HEAVY METAL) | boring | boring | 3.38 | 2.29 | 4.18 | PRIMED HIP HOP (ORIGINAL LYRICS HIP HOP) | boring | boring | 3.38 | 2.29 | 4.18 | PRIMED BOLERO (ORIGINAL LYRICS BOLERO) | boring | boring | 3.38 | 2.29 | 4.18 | PRIMED KOTO (ORIGINAL LYRICS KOTO) | boring | boring | 3.38 | 2.29 | 4.18 | PRIMED POP (ORIGINAL LYRICS POP) | boring | boring | 3.38 | 2.29 | 4.18 | PRIMED SAMBA (ORIGINAL LYRICS SAMBA) | nothing | nothing | #N/A | #N/A | #N/A |  |  |
| SYDNEY | z5157185 |  | Energized | Happy/content | Impressed | Curious | Scared | Calm | Bored | Enjoyment | Strongly Agree | 7 | Moderately Disagree | 2 | Neither Agree nor Disagree | 4 | Strongly Agree | 7 | Slightly Disagree | 3 | Slightly Agree | 5 | Strongly Agree | 7 | Strongly Agree | 7 | 5157185 | UNPRIMED (ORIGINAL LYRICS OPERA) | Disturbance | Disturbance | 3.09 | 4.96 | 4.65 | UNPRIMED (ORIGINAL LYRICS FADO) | concerned | concerned | 4.06 | 5.55 | 4.45 | UNPRIMED (ORIGINAL LYRICS HEAVY METAL) | fearful | Fearful | 2.25 | 6.33 | 3.64 | UNPRIMED (ORIGINAL LYRICS HIP HOP) | disgusted | disgusted | 2.45 | 5.42 | 4.34 | UNPRIMED (ORIGINAL LYRICS BOLERO) | sad | sad | 1.61 | 4.13 | 3.45 | UNPRIMED (ORIGINAL LYRICS BOLERO) | sad | sad | 1.61 | 4.13 | 3.45 | UNPRIMED (ORIGINAL LYRICS POP) | concerned | concerned | 4.06 | 5.55 | 4.45 | UNPRIMED (ORIGINAL LYRICS SAMBA) | sad | sad | 1.61 | 4.13 | 3.45 |  |  |
| SYDNEY | 5164833 |  | asdf | gud | asdf |  | asdf | asdf | asdf | asdf |  |  |  |  |  |  |  |  |  |  |  |  |  |  |  |  | 5164833 |  |  | #N/A | #N/A | #N/A | #N/A |  |  | #N/A | #N/A | #N/A | #N/A |  |  | #N/A | #N/A | #N/A | #N/A |  |  | #N/A | #N/A | #N/A | #N/A |  |  | #N/A | #N/A | #N/A | #N/A |  |  | #N/A | #N/A | #N/A | #N/A |  |  | #N/A | #N/A | #N/A | #N/A |  |  | #N/A | #N/A | #N/A | #N/A |  |  |
| SYDNEY | 5164975 |  | Energy | Loss | Passion | Spiritual | Anger | Annoyance | Cheerful | Relaxation | Neither Agree nor Disagree | 4 | Strongly Disagree | 1 | Strongly Agree | 7 | Slightly Agree | 5 | Moderately Agree | 6 | Moderately Agree | 6 | Neither Agree nor Disagree | 4 | Slightly Agree | 5 | 5164975 | UNPRIMED (ORIGINAL LYRICS OPERA) | Curiosity | Curiosity | 6.74 | 6.08 | 5.46 | UNPRIMED (ORIGINAL LYRICS FADO) | Hope | Hope | 7.05 | 5.44 | 5.52 | UNPRIMED (ORIGINAL LYRICS HEAVY METAL) | Horror | horror | 2.76 | 7.21 | 4.63 | UNPRIMED (ORIGINAL LYRICS HIP HOP) | Hopelessness | hopeless | 2.27 | 4.28 | 2.96 | UNPRIMED (ORIGINAL LYRICS BOLERO) | Loss | Loss | 1.89 | 5.78 | 2.38 | UNPRIMED (ORIGINAL LYRICS BOLERO) | Religion | 0 | #N/A | #N/A | #N/A | UNPRIMED (ORIGINAL LYRICS POP) | Rebellion | rebellious | 4.86 | 5.82 | 6.28 | UNPRIMED (ORIGINAL LYRICS SAMBA) | Sadness | sadness | 2.21 | 5.21 | 2.82 |  |  |
| SYDNEY | z5165414 |  | serenity | love | happy | anticipation | annoyed | interest | optimism | relaxed | I don't know this music | 8 | Strongly Disagree | 1 | Strongly Agree | 7 | Moderately Agree | 6 | Slightly Agree | 5 | Slightly Disagree | 3 | I don't know this music | 8 | I don't know this music | 8 | 5165414 | PRIMED OPERA (ORIGINAL LYRICS OPERA) | annoyance | annoyance | 2.97 | 5.18 | 4.21 | PRIMED FADO (ORIGINAL LYRICS FADO) | serenity | serene | 6.9 | 3.4 | 5.93 | PRIMED HEAVY METAL (ORIGINAL LYRICS HEAVY METAL) | aggressiveness | aggressive | 5.1 | 5.83 | 5.59 | PRIMED HIP HOP (ORIGINAL LYRICS HIP HOP) | disapproval | disapproval | #N/A | #N/A | #N/A | PRIMED BOLERO (ORIGINAL LYRICS BOLERO) | sadness | sadness | 2.21 | 5.21 | 2.82 | PRIMED KOTO (ORIGINAL LYRICS KOTO) | curiosity | Curiosity | 6.74 | 6.08 | 5.46 | PRIMED POP (ORIGINAL LYRICS POP) | distracted | distracted | 4.48 | 4.56 | 4.07 | PRIMED SAMBA (ORIGINAL LYRICS SAMBA) | anticipation | anticipation | #N/A | #N/A | #N/A |  |  |
| SYDNEY | z5165745 |  | calm | boredom | comfortable | boredom | agitated | cool | casual | expectant | Slightly Disagree | 3 | Strongly Disagree | 1 | Strongly Agree | 7 | Strongly Agree | 7 | Strongly Disagree | 1 | Neither Agree nor Disagree | 4 | Neither Agree nor Disagree | 4 | Neither Agree nor Disagree | 4 | 5165745 | PRIMED POP (ORIGINAL LYRICS OPERA) | conspicuous | conspicuous | #N/A | #N/A | #N/A | PRIMED OPERA (ORIGINAL LYRICS FADO) | accepting | accepting | #N/A | #N/A | #N/A | PRIMED CUBAN SON (ORIGINAL LYRICS HEAVY METAL) | tenacious | tenacious | #N/A | #N/A | #N/A | PRIMED SAMBA (ORIGINAL LYRICS HIP HOP) | helpless | helpless | 2.2 | 5.34 | 2.27 | PRIMED KOTO (ORIGINAL LYRICS BOLERO) | negative | negative | #N/A | #N/A | #N/A | PRIMED HEAVY METAL (ORIGINAL LYRICS KOTO) | vile | vile | #N/A | #N/A | #N/A | PRIMED FADO (ORIGINAL LYRICS POP) | empathetic | empathy | 5.32 | 4.81 | 4.33 | PRIMED HIP HOP (ORIGINAL LYRICS SAMBA) | betrayed | Betrayal | 2.28 | 5.37 | 4.18 |  |  |
| SYDNEY | z501805 |  | 1. Soothing 2. Playful | Soothing | Joy | Nostalgic | Anger | Rebellious | Party | Heroism | Moderately Agree | 6 | Strongly Disagree | 1 | Moderately Agree | 6 | Slightly Disagree | 3 | Slightly Disagree | 3 | Slightly Agree | 5 | Strongly Agree | 7 | Strongly Agree | 7 | 5165805 | PRIMED POP (ORIGINAL LYRICS OPERA) | happy | happy | 8.21 | 6.49 | 6.63 | PRIMED OPERA (ORIGINAL LYRICS FADO) | sad | sad | 1.61 | 4.13 | 3.45 | PRIMED CUBAN SON (ORIGINAL LYRICS HEAVY METAL) | carzy | carzy | #N/A | #N/A | #N/A | PRIMED SAMBA (ORIGINAL LYRICS HIP HOP) | sad | sad | 1.61 | 4.13 | 3.45 | PRIMED KOTO (ORIGINAL LYRICS BOLERO) | sad | sad | 1.61 | 4.13 | 3.45 | PRIMED HEAVY METAL (ORIGINAL LYRICS KOTO) | Critical | Critical | 3.94 | 4.72 | 5.13 | PRIMED FADO (ORIGINAL LYRICS POP) | carzy | carzy | #N/A | #N/A | #N/A | PRIMED HIP HOP (ORIGINAL LYRICS SAMBA) | sad | sad | 1.61 | 4.13 | 3.45 |  |  |
| SYDNEY | 5165805 |  | island | disney | spanish | tradition | thrilling | zone | generation Y | grandiose | Strongly Disagree | 1 | Strongly Disagree | 1 | Strongly Disagree | 1 | Strongly Disagree | 1 | Strongly Disagree | 1 | Strongly Disagree | 1 | Strongly Disagree | 1 | Strongly Disagree | 1 | 5165805 | PRIMED OPERA (ORIGINAL LYRICS OPERA) | overthinking | overthinking | #N/A | #N/A | #N/A | PRIMED FADO (ORIGINAL LYRICS FADO) | calling | calling | #N/A | #N/A | #N/A | PRIMED HEAVY METAL (ORIGINAL LYRICS HEAVY METAL) | dark | Dark | 4.71 | 4.28 | 4.84 | PRIMED HIP HOP (ORIGINAL LYRICS HIP HOP) | despair | Despair | 2.99 | 4.49 | 4.3 | PRIMED BOLERO (ORIGINAL LYRICS BOLERO) | sadness | sadness | 2.21 | 5.21 | 2.82 | PRIMED KOTO (ORIGINAL LYRICS KOTO) | religious | religion | 5.07 | 5.85 | 5.3 | PRIMED POP (ORIGINAL LYRICS POP) | violence | violence | #N/A | #N/A | #N/A | PRIMED SAMBA (ORIGINAL LYRICS SAMBA) | pain | pain | 2.13 | 6.5 | 3.71 |  |  |
| SYDNEY | 5165805 |  |  |  |  |  |  |  |  |  |  |  |  |  |  |  |  |  |  |  |  |  |  |  |  |  | 5165805 |  |  | #N/A | #N/A | #N/A | #N/A |  |  | #N/A | #N/A | #N/A | #N/A |  |  | #N/A | #N/A | #N/A | #N/A |  |  | #N/A | #N/A | #N/A | #N/A |  |  | #N/A | #N/A | #N/A | #N/A |  |  | #N/A | #N/A | #N/A | #N/A |  |  | #N/A | #N/A | #N/A | #N/A |  |  | #N/A | #N/A | #N/A | #N/A |  |  |
| SYDNEY | 5165805 |  | z | n | k | b | b | f | z | f | Strongly Disagree | 1 | Strongly Disagree | 1 | Strongly Disagree | 1 | Strongly Disagree | 1 | Strongly Disagree | 1 | Strongly Disagree | 1 | Strongly Disagree | 1 | Strongly Disagree | 1 | 5165805 |  |  | #N/A | #N/A | #N/A | #N/A |  |  | #N/A | #N/A | #N/A | #N/A |  |  | #N/A | #N/A | #N/A | #N/A |  |  | #N/A | #N/A | #N/A | #N/A |  |  | #N/A | #N/A | #N/A | #N/A |  |  | #N/A | #N/A | #N/A | #N/A |  |  | #N/A | #N/A | #N/A | #N/A |  |  | #N/A | #N/A | #N/A | #N/A |  |  |
| SYDNEY | 6758405 |  |  |  |  |  |  |  |  |  |  |  |  |  |  |  |  |  |  |  |  |  |  |  |  |  | 6758405 |  |  | #N/A | #N/A | #N/A | #N/A |  |  | #N/A | #N/A | #N/A | #N/A |  | Below are the lyrics from a Heavy Metal excerpt. "Blood on my conscious And murder in mind Out of the gloom I rise up from my tomb into impending doom Now my body is my shrine"  What is the first emotion that comes to your mind when you read these lyrics? Please provide just one word. There is no right or wrong answer. | #VALUE! | #VALUE! | #VALUE! | ###### |  |  | #N/A | #N/A | #N/A | #N/A |  |  | #N/A | #N/A | #N/A | #N/A |  |  | #N/A | #N/A | #N/A | #N/A |  |  | #N/A | #N/A | #N/A | #N/A |  |  | #N/A | #N/A | #N/A | #N/A |  |  |
| SYDNEY | z501805 |  | 1. Soothing 2. Playful | Soothing | Joy | Nostalgic | Anger | Rebellious | Party | Heroism | Moderately Agree | 6 | Strongly Disagree | 1 | Moderately Agree | 6 | Slightly Disagree | 3 | Slightly Disagree | 3 | Slightly Agree | 5 | Strongly Agree | 7 | Strongly Agree | 7 | #VALUE! |  |  | #N/A | #N/A | #N/A | #N/A |  |  | #N/A | #N/A | #N/A | #N/A |  |  | #N/A | #N/A | #N/A | #N/A |  |  | #N/A | #N/A | #N/A | #N/A |  |  | #N/A | #N/A | #N/A | #N/A |  |  | #N/A | #N/A | #N/A | #N/A |  |  | #N/A | #N/A | #N/A | #N/A |  |  | #N/A | #N/A | #N/A | #N/A |  |  |
| SYDNEY | EDST2092 |  |  |  |  |  |  |  |  |  |  |  |  |  |  |  |  |  |  |  |  |  |  |  |  |  | #VALUE! |  |  | #N/A | #N/A | #N/A | #N/A |  |  | #N/A | #N/A | #N/A | #N/A |  |  | #N/A | #N/A | #N/A | #N/A |  |  | #N/A | #N/A | #N/A | #N/A |  |  | #N/A | #N/A | #N/A | #N/A |  |  | #N/A | #N/A | #N/A | #N/A |  |  | #N/A | #N/A | #N/A | #N/A |  |  | #N/A | #N/A | #N/A | #N/A |  |  |
| SYDNEY | afefaef |  |  |  |  |  |  |  |  |  |  |  |  |  |  |  |  |  |  |  |  |  |  |  |  |  | #VALUE! |  |  | #N/A | #N/A | #N/A | #N/A |  |  | #N/A | #N/A | #N/A | #N/A |  |  | #N/A | #N/A | #N/A | #N/A |  |  | #N/A | #N/A | #N/A | #N/A |  |  | #N/A | #N/A | #N/A | #N/A |  |  | #N/A | #N/A | #N/A | #N/A |  |  | #N/A | #N/A | #N/A | #N/A |  |  | #N/A | #N/A | #N/A | #N/A |  |  |
| SYDNEY | ssss |  |  |  |  |  |  |  |  |  |  |  |  |  |  |  |  |  |  |  |  |  |  |  |  |  | #VALUE! |  |  | #N/A | #N/A | #N/A | #N/A |  |  | #N/A | #N/A | #N/A | #N/A |  |  | #N/A | #N/A | #N/A | #N/A |  |  | #N/A | #N/A | #N/A | #N/A |  |  | #N/A | #N/A | #N/A | #N/A |  |  | #N/A | #N/A | #N/A | #N/A |  |  | #N/A | #N/A | #N/A | #N/A |  |  | #N/A | #N/A | #N/A | #N/A |  |  |
| SYDNEY | Sgsje |  |  |  | W |  |  | W |  |  |  |  |  |  |  |  |  |  |  |  |  |  |  |  |  |  | #VALUE! |  |  | #N/A | #N/A | #N/A | #N/A |  |  | #N/A | #N/A | #N/A | #N/A |  |  | #N/A | #N/A | #N/A | #N/A |  |  | #N/A | #N/A | #N/A | #N/A |  |  | #N/A | #N/A | #N/A | #N/A |  |  | #N/A | #N/A | #N/A | #N/A |  |  | #N/A | #N/A | #N/A | #N/A |  |  | #N/A | #N/A | #N/A | #N/A |  |  |
| SYDNEY | dsaf |  |  |  |  |  |  |  |  |  |  |  |  |  |  |  |  |  |  |  |  |  |  |  |  |  | #VALUE! |  |  | #N/A | #N/A | #N/A | #N/A |  |  | #N/A | #N/A | #N/A | #N/A |  |  | #N/A | #N/A | #N/A | #N/A |  |  | #N/A | #N/A | #N/A | #N/A |  |  | #N/A | #N/A | #N/A | #N/A |  |  | #N/A | #N/A | #N/A | #N/A |  |  | #N/A | #N/A | #N/A | #N/A |  |  | #N/A | #N/A | #N/A | #N/A |  |  |
|  |  |  |  |  |  |  |  |  |  |  |  |  |  |  |  |  |  |  |  |  |  |  |  |  |  |  |  |  |  | #N/A | #N/A | #N/A | #N/A |  |  | #N/A | #N/A | #N/A | #N/A |  |  | #N/A | #N/A | #N/A | #N/A |  |  | #N/A | #N/A | #N/A | #N/A |  |  | #N/A | #N/A | #N/A | #N/A |  |  | #N/A | #N/A | #N/A | #N/A |  |  | #N/A | #N/A | #N/A | #N/A |  |  | #N/A | #N/A | #N/A | #N/A |  |  |
| CUBA |  | CN = Congruent | joy | tranquility | feeling | oriental | torment | action | relax | big |  | 7 |  | 8 |  | 5 |  | 8 |  | 8 |  | 7 |  | 7 |  |  | 1 | PRIMED OPERA (Otiginal Lyrics Opera) | shame | shame | 2.13 | 6.33 | 2.97 |  | benediction | #N/A | #N/A | #N/A | #N/A | PRIMED HEAVY METAL (OIGINAL LYRICS HEAVY METAL) | death | Death | 1.61 | 4.59 | 3.47 | PRIMED HIP HOP | abrupt | abrupt | 4.22 | 4.95 | 4.78 | PRIMED BOLERO (ORIGINAL LYRICS BOLERO) | pain | pain | 2.13 | 6.5 | 3.71 | PRIMED KOTO (ORIGINAL LYRICS KOTO) |  | Reflection | 5.97 | 4.38 | 6.1 | PRIMED POP (ORIGINAL LYRICS POP) | nothing | nothing | #N/A | #N/A | #N/A | PRIMED SAMBA(ORIGINAL LYRICS SAMBA) | fear | fear | 2.76 | 6.96 | 3.22 |  |  |
| CUBA |  | CN = Congruent | pretty | nice | flavourful | regional | nice | joy | excellent | beauty |  | 5 |  | 8 |  | 8 |  | 6 |  | 7 |  | 6 |  | 7 |  | 7 | 2 | PRIMED OPERA (Otiginal Lyrics Opera) | disharmony | disharmony | #N/A | #N/A | #N/A | PRIMED FADO (ORIGINAL LYRICS FADO) | pretty | pretty | 7.75 | 6.03 | 5.5 | PRIMED HEAVY METAL (OIGINAL LYRICS HEAVY METAL) | nothing | nothing | #N/A | #N/A | #N/A | PRIMED HIP HOP | emotional | Emotional | 4.36 | 5.75 | 4.29 | PRIMED BOLERO (ORIGINAL LYRICS BOLERO) | original | original | 6.23 | 5.14 | 5.69 | PRIMED KOTO (ORIGINAL LYRICS KOTO) | nice | nice | 6.55 | 4.38 | 5.58 | PRIMED POP (ORIGINAL LYRICS POP) | nothing | nothing | #N/A | #N/A | #N/A | PRIMED SAMBA(ORIGINAL LYRICS SAMBA) | hate | Hate | 2.12 | 6.95 | 5.05 |  |  |
| CUBA |  | CN = Congruent | feeling |  | cubanic | nothing | nothing | culture | \* |  | I don't know it but I love it | 7 | I don't like it | 1 | I like it | 7 | I like it but it's sometimes racist | 6 |  | 1 |  |  |  |  |  | 1 | 3 | PRIMED OPERA (Otiginal Lyrics Opera) | tenderness | tenderness | 6.36 | 4.48 | 5.28 | PRIMED FADO (ORIGINAL LYRICS FADO) | | #N/A | #N/A | #N/A | #N/A | PRIMED HEAVY METAL (OIGINAL LYRICS HEAVY METAL) |  | #N/A | #N/A | #N/A | #N/A | PRIMED HIP HOP |  | #N/A | #N/A | #N/A | #N/A | PRIMED BOLERO (ORIGINAL LYRICS BOLERO) | deceit | deceit | 2.9 | 5.68 | 3.95 | PRIMED KOTO (ORIGINAL LYRICS KOTO) |  | #N/A | #N/A | #N/A | #N/A | PRIMED POP (ORIGINAL LYRICS POP) | vice | vice | 4.43 | 5.3 | 5.23 | PRIMED SAMBA(ORIGINAL LYRICS SAMBA) | fear | fear | 2.76 | 6.96 | 3.22 |  |  |
| CUBA |  | CN = Congruent | nice | rhythmic | flavourful | weird | madness | joy | emotional | relax |  | 1 |  | 1 |  | 8 |  | 7 |  | 2 |  | 7 |  | 8 |  | 7 | 4 | PRIMED OPERA (Otiginal Lyrics Opera) | impression | impression | #N/A | #N/A | #N/A | PRIMED FADO (ORIGINAL LYRICS FADO) | pride | Pride | 7 | 5.83 | 7.06 | PRIMED HEAVY METAL (OIGINAL LYRICS HEAVY METAL) | hate | Hate | 2.12 | 6.95 | 5.05 | PRIMED HIP HOP | pleasurable | pleasurable | #N/A | #N/A | #N/A | PRIMED BOLERO (ORIGINAL LYRICS BOLERO) | love | love | 8.72 | 6.44 | 7.11 | PRIMED KOTO (ORIGINAL LYRICS KOTO) | weird | weird | 5.17 | 4.82 | 5.13 | PRIMED POP (ORIGINAL LYRICS POP) | vulgar | vulgar | 3.84 | 4.63 | 5.08 | PRIMED SAMBA(ORIGINAL LYRICS SAMBA) | love | love | 8.72 | 6.44 | 7.11 |  |  |
| CUBA |  | CN = Congruent | happy, feeling | love, sad | flavourful/suffering | loneliness | fury, madness | fury | swing | passion, grand |  | 6 |  | 1 |  | 2 |  | 2 |  | 1 |  | 4 |  |  |  | 7 | 5 | PRIMED OPERA (Otiginal Lyrics Opera) | void | void | 3.73 | 3.92 | 4.12 | PRIMED FADO (ORIGINAL LYRICS FADO) | | #N/A | #N/A | #N/A | #N/A | PRIMED HEAVY METAL (OIGINAL LYRICS HEAVY METAL) |  | #N/A | #N/A | #N/A | #N/A | PRIMED HIP HOP |  | #N/A | #N/A | #N/A | #N/A | PRIMED BOLERO (ORIGINAL LYRICS BOLERO) | betrayal | Betrayal | 2.28 | 5.37 | 4.18 | PRIMED KOTO (ORIGINAL LYRICS KOTO) |  | #N/A | #N/A | #N/A | #N/A | PRIMED POP (ORIGINAL LYRICS POP) |  | #N/A | #N/A | #N/A | #N/A | PRIMED SAMBA(ORIGINAL LYRICS SAMBA) |  | #N/A | #N/A | #N/A | #N/A |  |  |
| CUBA |  | CN = Congruent | dancing, happy | feeling, intimacy | joy, rhythmic | surrealism, mystery | energy | real, raw | simple | beauty | I like it a lot | 5 | it attracts me a little | 6 | I don't like it very much | 3 | has ear and movement | 6 |  | 8 | has complexity and enjoyment, I really like it | 6 | It provokes nice feeling in me | 4 | I like cuban music | 4 | 6 | PRIMED OPERA (Otiginal Lyrics Opera) | femininity | femininity | #N/A | #N/A | #N/A | PRIMED FADO (ORIGINAL LYRICS FADO) | tearful | tearful | #N/A | #N/A | #N/A | PRIMED HEAVY METAL (OIGINAL LYRICS HEAVY METAL) | obsurd | obsurd | #N/A | #N/A | #N/A | PRIMED HIP HOP | desperation | despair | 2.99 | 4.49 | 4.3 | PRIMED BOLERO (ORIGINAL LYRICS BOLERO) | pain | pain | 2.13 | 6.5 | 3.71 | PRIMED KOTO (ORIGINAL LYRICS KOTO) | spirituality | spirit | 7 | 5.56 | 5.82 | PRIMED POP (ORIGINAL LYRICS POP) |  | #N/A | #N/A | #N/A | #N/A | PRIMED SAMBA(ORIGINAL LYRICS SAMBA) | insecurity | insecure | 2.36 | 5.56 | 2.33 |  |  |
| CUBA |  | CN = Congruent | joy | sad | romance | peace | noise | decisive | movement | pleasurable |  | 5 |  | 1 |  | 5 |  | 5 |  | 4 |  | 5 |  | 4 |  | 5 | 7 | PRIMED OPERA (Otiginal Lyrics Opera) | narcissism | narcissism | #N/A | #N/A | #N/A | PRIMED FADO (ORIGINAL LYRICS FADO) | sad | sad | 1.61 | 4.13 | 3.45 | PRIMED HEAVY METAL (OIGINAL LYRICS HEAVY METAL) | pessimistic | pessimistic | 2.77 | 4.03 | 4.8 | PRIMED HIP HOP | conflict | conflict | #N/A | #N/A | #N/A | PRIMED BOLERO (ORIGINAL LYRICS BOLERO) | love | love | 8.72 | 6.44 | 7.11 | PRIMED KOTO (ORIGINAL LYRICS KOTO) | connection | connection | #N/A | #N/A | #N/A | PRIMED POP (ORIGINAL LYRICS POP) |  | #N/A | #N/A | #N/A | #N/A | PRIMED SAMBA(ORIGINAL LYRICS SAMBA) | fear | fear | 2.76 | 6.96 | 3.22 |  |  |
| CUBA |  | CN = Congruent | joy | sad | yearning | sad | anger | energy, motivation | jumping | praise |  | 6 |  | 8 |  | 7 |  | 7 |  | 6 |  | 6 |  | 7 |  | 7 | 8 | PRIMED OPERA (Otiginal Lyrics Opera) | flirting | flirting | 7.04 | 7.03 | 5.48 | PRIMED FADO (ORIGINAL LYRICS FADO) | patriotism | patriotism | 6.85 | 5.56 | 6.52 | PRIMED HEAVY METAL (OIGINAL LYRICS HEAVY METAL) | sad | sad | 1.61 | 4.13 | 3.45 | PRIMED HIP HOP | sad | sad | 1.61 | 4.13 | 3.45 | PRIMED BOLERO (ORIGINAL LYRICS BOLERO) | sad | sad | 1.61 | 4.13 | 3.45 | PRIMED KOTO (ORIGINAL LYRICS KOTO) | energy | energy | 7.23 | 6.9 | 6.93 | PRIMED POP (ORIGINAL LYRICS POP) | violence | violence | #N/A | #N/A | #N/A | PRIMED SAMBA(ORIGINAL LYRICS SAMBA) | heartbreak | heartbreak | 1.93 | 5.8 | 3.11 |  |  |
| CUBA |  | CN = Congruent | party | nostalgia, melancholy | tranquility, yearning | peace | violence, torment | emotion | flavourful | love, beauty |  | 6 |  | 2 |  | 7 |  | 7 |  | 8 |  | 6 |  | 7 |  | 7 | 9 | PRIMED OPERA (Otiginal Lyrics Opera) | pride | Pride | 7 | 5.83 | 7.06 | PRIMED FADO (ORIGINAL LYRICS FADO) | patriotism | patriotism | 6.85 | 5.56 | 6.52 | PRIMED HEAVY METAL (OIGINAL LYRICS HEAVY METAL) | madness | madness | 4.37 | 5.8 | 4.53 | PRIMED HIP HOP | impatience | impatience | #N/A | #N/A | #N/A | PRIMED BOLERO (ORIGINAL LYRICS BOLERO) | betrayal | Betrayal | 2.28 | 5.37 | 4.18 | PRIMED KOTO (ORIGINAL LYRICS KOTO) | suplication | suplication | #N/A | #N/A | #N/A | PRIMED POP (ORIGINAL LYRICS POP) | freedom | freedom | 7.58 | 5.52 | 6.76 | PRIMED SAMBA(ORIGINAL LYRICS SAMBA) | hope | Hope | 7.05 | 5.44 | 5.52 |  |  |
| CUBA |  | CN = Congruent | joy,soulful | sad | joy,childhood | peace | fury, trance | protest, fight | banality | relax |  |  |  |  |  |  |  |  |  |  |  |  |  |  |  |  | 10 | PRIMED OPERA (Otiginal Lyrics Opera) | security | Security | 7.28 | 4.22 | 5.53 | PRIMED FADO (ORIGINAL LYRICS FADO) | sufferring | suffer | 1.72 | 6.13 | 2.54 | PRIMED HEAVY METAL (OIGINAL LYRICS HEAVY METAL) | violence | violence | #N/A | #N/A | #N/A | PRIMED HIP HOP | streets | streets | #N/A | #N/A | #N/A | PRIMED BOLERO (ORIGINAL LYRICS BOLERO) | love | love | 8.72 | 6.44 | 7.11 | PRIMED KOTO (ORIGINAL LYRICS KOTO) | divine | divine | #N/A | #N/A | #N/A | PRIMED POP (ORIGINAL LYRICS POP) | marginality | marginality | #N/A | #N/A | #N/A | PRIMED SAMBA(ORIGINAL LYRICS SAMBA) | poetry | poetry | 5.86 | 4 | 5.31 |  |  |
| CUBA |  | CN = Congruent | tranquility | serenity, sad | reflection | attention | equality | unbearable | joy | concentration |  |  |  |  |  |  |  |  |  |  |  |  |  |  |  |  | 11 | PRIMED OPERA (Otiginal Lyrics Opera) | enchantment | enchantment | #N/A | #N/A | #N/A | PRIMED FADO (ORIGINAL LYRICS FADO) | lament | lament | #N/A | #N/A | #N/A | PRIMED HEAVY METAL (OIGINAL LYRICS HEAVY METAL) | horror | horror | 2.76 | 7.21 | 4.63 | PRIMED HIP HOP |  | #N/A | #N/A | #N/A | #N/A | PRIMED BOLERO (ORIGINAL LYRICS BOLERO) | sad | sad | 1.61 | 4.13 | 3.45 | PRIMED KOTO (ORIGINAL LYRICS KOTO) | reflection | Reflection | 5.97 | 4.38 | 6.1 | PRIMED POP (ORIGINAL LYRICS POP) | desregard | desregard | #N/A | #N/A | #N/A | PRIMED SAMBA(ORIGINAL LYRICS SAMBA) | cuban | cuban | #N/A | #N/A | #N/A |  |  |
| CUBA |  | CN = Congruent | joy, colours | love | joy | meditation | war | invasion | joy | serenity |  |  |  |  |  |  |  |  |  |  |  |  |  |  |  |  | 12 | PRIMED OPERA (Otiginal Lyrics Opera) | invasion | invasion | #N/A | #N/A | #N/A | PRIMED FADO (ORIGINAL LYRICS FADO) | beauty | beauty | 7.82 | 4.95 | 5.53 | PRIMED HEAVY METAL (OIGINAL LYRICS HEAVY METAL) | war | war | 2.08 | 7.49 | 4.5 | PRIMED HIP HOP | \* | 0 | #N/A | #N/A | #N/A | PRIMED BOLERO (ORIGINAL LYRICS BOLERO) | passion | passion | 8.03 | 7.26 | 6.13 | PRIMED KOTO (ORIGINAL LYRICS KOTO) | spirituality | spirit | 7 | 5.56 | 5.82 | PRIMED POP (ORIGINAL LYRICS POP) | nothing | nothing | #N/A | #N/A | #N/A | PRIMED SAMBA(ORIGINAL LYRICS SAMBA) | sad | sad | 1.61 | 4.13 | 3.45 |  |  |
| CUBA |  | CN = Congruent |  | audabile | tranquility | interesting | beautiful | boredom, disgust | nice | enjoyable |  | 6 |  | 8 |  | 4 |  | 2 |  | 7 |  | 6 |  | 4 |  | 4 | 13 | PRIMED OPERA (Otiginal Lyrics Opera) | boastful | boastful | #N/A | #N/A | #N/A | PRIMED FADO (ORIGINAL LYRICS FADO) | feeling | feeling | 6.23 | 5 | 5.62 | PRIMED HEAVY METAL (OIGINAL LYRICS HEAVY METAL) | darkness | darkness | #N/A | #N/A | #N/A | PRIMED HIP HOP | criminality | criminality | #N/A | #N/A | #N/A | PRIMED BOLERO (ORIGINAL LYRICS BOLERO) | attraction | attractive | 7.49 | 6.76 | 5.54 | PRIMED KOTO (ORIGINAL LYRICS KOTO) | spirituality | spirit | 7 | 5.56 | 5.82 | PRIMED POP (ORIGINAL LYRICS POP) | delinquency | delinquency | #N/A | #N/A | #N/A | PRIMED SAMBA(ORIGINAL LYRICS SAMBA) | pain | pain | 2.13 | 6.5 | 3.71 |  |  |
| CUBA |  | CN = Congruent | party | relax, meditation | joy, peaceful |  | madness, harsh |  | joy | relaxation, storm |  |  |  |  |  |  |  |  |  |  |  |  |  |  |  |  | 14 | PRIMED OPERA (Otiginal Lyrics Opera) | idiocy | idiocy | #N/A | #N/A | #N/A | PRIMED FADO (ORIGINAL LYRICS FADO) | | #N/A | #N/A | #N/A | #N/A | PRIMED HEAVY METAL (OIGINAL LYRICS HEAVY METAL) |  | #N/A | #N/A | #N/A | #N/A | PRIMED HIP HOP |  | #N/A | #N/A | #N/A | #N/A | PRIMED BOLERO (ORIGINAL LYRICS BOLERO) |  | #N/A | #N/A | #N/A | #N/A | PRIMED KOTO (ORIGINAL LYRICS KOTO) |  | #N/A | #N/A | #N/A | #N/A | PRIMED POP (ORIGINAL LYRICS POP) |  | #N/A | #N/A | #N/A | #N/A | PRIMED SAMBA(ORIGINAL LYRICS SAMBA) |  | #N/A | #N/A | #N/A | #N/A |  |  |
| CUBA |  | CN = Congruent |  | relax | melancholy, longing | mistery | euphoric | aggressive | bored, excitement | peace |  | 6 |  | 5 |  | 4 |  | 2 |  | 8 |  | 4 |  | 7 |  | 6 | 15 | PRIMED OPERA (Otiginal Lyrics Opera) | sex | sex | 8.05 | 7.36 | 5.75 | PRIMED FADO (ORIGINAL LYRICS FADO) | sad | sad | 1.61 | 4.13 | 3.45 | PRIMED HEAVY METAL (OIGINAL LYRICS HEAVY METAL) | spirituality | spirit | 7 | 5.56 | 5.82 | PRIMED HIP HOP | pain | pain | 2.13 | 6.5 | 3.71 | PRIMED BOLERO (ORIGINAL LYRICS BOLERO) | love, attraction | love | 8.72 | 6.44 | 7.11 | PRIMED KOTO (ORIGINAL LYRICS KOTO) |  | #N/A | #N/A | #N/A | #N/A | PRIMED POP (ORIGINAL LYRICS POP) | madnesseness | madness | 4.37 | 5.8 | 4.53 | PRIMED SAMBA(ORIGINAL LYRICS SAMBA) |  | #N/A | #N/A | #N/A | #N/A |  |  |
| CUBA |  | CN = Congruent | joy, nostalgia | melancholy, loneliness | flavourful, root | rigid, bored | headache, impatiance | feeling, rhythm | love | peace, serene | It's the music of my land | 7 |  |  |  |  |  |  |  |  |  |  |  |  |  |  | 16 | PRIMED OPERA (Otiginal Lyrics Opera) | orgasm | orgasm | 8.32 | 8.1 | 6.83 | PRIMED FADO (ORIGINAL LYRICS FADO) | passion | passion | 8.03 | 7.26 | 6.13 | PRIMED HEAVY METAL (OIGINAL LYRICS HEAVY METAL) | aggressive | aggressive | 5.1 | 5.83 | 5.59 | PRIMED HIP HOP | betrayal | Betrayal | 2.28 | 5.37 | 4.18 | PRIMED BOLERO (ORIGINAL LYRICS BOLERO) | sad | sad | 1.61 | 4.13 | 3.45 | PRIMED KOTO (ORIGINAL LYRICS KOTO) | cowardice | cowardice | 2.9 | 4.27 | 4.49 | PRIMED POP (ORIGINAL LYRICS POP) | drugs | drugs | 3.76 | 6 | 4.75 | PRIMED SAMBA(ORIGINAL LYRICS SAMBA) | fear | fear | 2.76 | 6.96 | 3.22 |  |  |
| CUBA |  | CN = Congruent | yearning | sad | meditation | mystery | stress-relief | freedom | relax | majestic |  | 7 |  | 8 |  | 5 |  | 4 |  | 4 |  | 6 |  | 4 |  | 7 | 17 | PRIMED OPERA (Otiginal Lyrics Opera) | sassy | sassy | #N/A | #N/A | #N/A | PRIMED FADO (ORIGINAL LYRICS FADO) | | #N/A | #N/A | #N/A | #N/A | PRIMED HEAVY METAL (OIGINAL LYRICS HEAVY METAL) |  | #N/A | #N/A | #N/A | #N/A | PRIMED HIP HOP |  | #N/A | #N/A | #N/A | #N/A | PRIMED BOLERO (ORIGINAL LYRICS BOLERO) |  | #N/A | #N/A | #N/A | #N/A | PRIMED KOTO (ORIGINAL LYRICS KOTO) |  | #N/A | #N/A | #N/A | #N/A | PRIMED POP (ORIGINAL LYRICS POP) |  | #N/A | #N/A | #N/A | #N/A | PRIMED SAMBA(ORIGINAL LYRICS SAMBA) |  | #N/A | #N/A | #N/A | #N/A |  |  |
| CUBA |  | CN = Congruent | festive, intimate |  | dancing, romantic | spiritual, religeous | discoteque, alcoholism | handsome | good humour | calm, nature |  | 6 |  | 1 |  | 7 |  | 6 |  | 7 |  | 6 |  | 6 | I live this music everyday because I am part of a band | 7 | 18 | PRIMED OPERA (Otiginal Lyrics Opera) | love | love | 8.72 | 6.44 | 7.11 | PRIMED FADO (ORIGINAL LYRICS FADO) | passion | passion | 8.03 | 7.26 | 6.13 | PRIMED HEAVY METAL (OIGINAL LYRICS HEAVY METAL) |  | #N/A | #N/A | #N/A | #N/A | PRIMED HIP HOP | gang | gang | 2.59 | 6.52 | 3.33 | PRIMED BOLERO (ORIGINAL LYRICS BOLERO) | attraction | attractive | 7.49 | 6.76 | 5.54 | PRIMED KOTO (ORIGINAL LYRICS KOTO) | spirituality | spirit | 7 | 5.56 | 5.82 | PRIMED POP (ORIGINAL LYRICS POP) | drugs | drugs | 3.76 | 6 | 4.75 | PRIMED SAMBA(ORIGINAL LYRICS SAMBA) | fear of love | fear of love | #N/A | #N/A | #N/A |  |  |
| CUBA |  | CN = Congruent | joy,tranquility | nostalgia | party, dancing | concentration, relax | noise | speculation,gesticulation | flavourful,joy | calm,mysterious |  | 6 |  | 1 |  | 7 |  | 3 |  | 4 |  | 6 |  | 4 |  | 7 | 19 | PRIMED OPERA (Otiginal Lyrics Opera) | sensual | Sensual | #N/A | #N/A | #N/A | PRIMED FADO (ORIGINAL LYRICS FADO) | sad | sad | 1.61 | 4.13 | 3.45 | PRIMED HEAVY METAL (OIGINAL LYRICS HEAVY METAL) | violence | violence | #N/A | #N/A | #N/A | PRIMED HIP HOP |  | #N/A | #N/A | #N/A | #N/A | PRIMED BOLERO (ORIGINAL LYRICS BOLERO) | loveless | loveless | #N/A | #N/A | #N/A | PRIMED KOTO (ORIGINAL LYRICS KOTO) | divine | divine | #N/A | #N/A | #N/A | PRIMED POP (ORIGINAL LYRICS POP) | drug trafficers | drug trafficers | #N/A | #N/A | #N/A | PRIMED SAMBA(ORIGINAL LYRICS SAMBA) | decompress | decompress | #N/A | #N/A | #N/A |  |  |
| CUBA |  | CN = Congruent | joy | sad | love |  |  | tragedy |  | passion |  | 5 |  | 8 |  | 7 |  | 1 |  | 6 |  | 6 |  |  |  | 7 | 20 | PRIMED OPERA (Otiginal Lyrics Opera) | love | love | 8.72 | 6.44 | 7.11 | PRIMED FADO (ORIGINAL LYRICS FADO) | | #N/A | #N/A | #N/A | #N/A | PRIMED HEAVY METAL (OIGINAL LYRICS HEAVY METAL) | tragedy | tragedy | 1.78 | 6.24 | 3.5 | PRIMED HIP HOP |  | #N/A | #N/A | #N/A | #N/A | PRIMED BOLERO (ORIGINAL LYRICS BOLERO) | suffering | suffer | 1.72 | 6.13 | 2.54 | PRIMED KOTO (ORIGINAL LYRICS KOTO) |  | #N/A | #N/A | #N/A | #N/A | PRIMED POP (ORIGINAL LYRICS POP) | tragedy | tragedy | 1.78 | 6.24 | 3.5 | PRIMED SAMBA(ORIGINAL LYRICS SAMBA) | love | love | 8.72 | 6.44 | 7.11 |  |  |
| CUBA |  | CN = Congruent | joy,passion | melancholy | joy,love | spirituality,calm | fury,anger | rage,anger | sensual,love | nostalgia | It uses chords which are very interesting (like Jazz) | 6 |  | 8 |  | 4 |  | 5 |  | 8 |  | 6 |  | 7 | and melodies that enrich the music | 7 | 21 | PRIMED OPERA (Otiginal Lyrics Opera) | sensual | Sensual | #N/A | #N/A | #N/A | PRIMED FADO (ORIGINAL LYRICS FADO) | melancholy | melancholy | 3.82 | 3.6 | 4.65 | PRIMED HEAVY METAL (OIGINAL LYRICS HEAVY METAL) | rage | rage | 2.41 | 8.17 | 5.68 | PRIMED HIP HOP | violence | violence | #N/A | #N/A | #N/A | PRIMED BOLERO (ORIGINAL LYRICS BOLERO) | love | love | 8.72 | 6.44 | 7.11 | PRIMED KOTO (ORIGINAL LYRICS KOTO) | spirituality | spirit | 7 | 5.56 | 5.82 | PRIMED POP (ORIGINAL LYRICS POP) | vanity | Vanity | 4.3 | 4.98 | 4.8 | PRIMED SAMBA(ORIGINAL LYRICS SAMBA) | love | love | 8.72 | 6.44 | 7.11 |  |  |
| CUBA |  | CN = Congruent | dancing | relax | dacing | nothing | stressful | bad environment | joy | tenderness |  | 5 |  | 3 |  | 6 |  | 4 |  | 8 |  | 5 |  | 4 |  | 7 | 22 | PRIMED OPERA (Otiginal Lyrics Opera) | pride | Pride | 7 | 5.83 | 7.06 | PRIMED FADO (ORIGINAL LYRICS FADO) | patriotism | patriotism | 6.85 | 5.56 | 6.52 | PRIMED HEAVY METAL (OIGINAL LYRICS HEAVY METAL) | hell | hell | 2.24 | 5.38 | 3.24 | PRIMED HIP HOP | despair | Despair | 2.99 | 4.49 | 4.3 | PRIMED BOLERO (ORIGINAL LYRICS BOLERO) | betrayal | Betrayal | 2.28 | 5.37 | 4.18 | PRIMED KOTO (ORIGINAL LYRICS KOTO) | religeous | religion | 5.07 | 5.85 | 5.3 | PRIMED POP (ORIGINAL LYRICS POP) | waste | waste | 2.93 | 4.14 | 4.72 | PRIMED SAMBA(ORIGINAL LYRICS SAMBA) | insecurity | insecure | 2.36 | 5.56 | 2.33 |  |  |
| CUBA |  | CN = Congruent | joy,romance | sad,sorrow | joy,love | confusion | alteration,disturbance | protest | calm,diversion | relax |  | 6 |  | 1 |  | 4 |  | 7 |  | 1 |  | 5 |  | 6 |  | 7 | 23 | PRIMED OPERA (Otiginal Lyrics Opera) | high self-esteem | high self-esteem | #N/A | #N/A | #N/A | PRIMED FADO (ORIGINAL LYRICS FADO) | patriotism | patriotism | 6.85 | 5.56 | 6.52 | PRIMED HEAVY METAL (OIGINAL LYRICS HEAVY METAL) | protest | protest | #N/A | #N/A | #N/A | PRIMED HIP HOP | sad,sorrow | sad | 1.61 | 4.13 | 3.45 | PRIMED BOLERO (ORIGINAL LYRICS BOLERO) | romance,sorrow,sad | romance | 7.61 | 6.9 | 6.03 | PRIMED KOTO (ORIGINAL LYRICS KOTO) | confusion | confusion | 3.46 | 6.07 | 3.04 | PRIMED POP (ORIGINAL LYRICS POP) | confusion | confusion | 3.46 | 6.07 | 3.04 | PRIMED SAMBA(ORIGINAL LYRICS SAMBA) | fear | fear | 2.76 | 6.96 | 3.22 |  |  |
| CUBA |  | CN = Congruent | happy,relax | sad,pensive | intimate,romantic | spiritual,weird | strong,different, amazing | protesting, interesting | dancing,rhythmic | relaxing,sentimental,divine |  | 5 |  | 6 |  | 6 |  | 6 |  | 1 |  | 6 |  | 4 |  | 5 | 24 | PRIMED OPERA (Otiginal Lyrics Opera) | intimate | intimate | 7.61 | 6.98 | 5.86 | PRIMED FADO (ORIGINAL LYRICS FADO) | sad | sad | 1.61 | 4.13 | 3.45 | PRIMED HEAVY METAL (OIGINAL LYRICS HEAVY METAL) | fear | fear | 2.76 | 6.96 | 3.22 | PRIMED HIP HOP | violence | violence | #N/A | #N/A | #N/A | PRIMED BOLERO (ORIGINAL LYRICS BOLERO) | love | love | 8.72 | 6.44 | 7.11 | PRIMED KOTO (ORIGINAL LYRICS KOTO) | solemn | solemn | 4.32 | 3.56 | 4.61 | PRIMED POP (ORIGINAL LYRICS POP) | tough | tough | 5.24 | 5.43 | 6.38 | PRIMED SAMBA(ORIGINAL LYRICS SAMBA) | passion | passion | 8.03 | 7.26 | 6.13 |  |  |
| CUBA |  | CN = Congruent | joy | sad | calm |  | stress | relax,calm | relax, joy | relax | I listen to this music two hours a week | 2 | I listen to this music one hour a week | 2 | I listen to this music three hours a week | 6 | I listen to this music two hours a week | 6 |  | 2 | I listen to this music three hours a week | 6 | I don't listen to this music | 7 | I listen to this music five hours a week | 7 | 25 | PRIMED OPERA (Otiginal Lyrics Opera) | mystery | mystery | #N/A | #N/A | #N/A | PRIMED FADO (ORIGINAL LYRICS FADO) | sad | sad | 1.61 | 4.13 | 3.45 | PRIMED HEAVY METAL (OIGINAL LYRICS HEAVY METAL) | death | Death | 1.61 | 4.59 | 3.47 | PRIMED HIP HOP | death | Death | 1.61 | 4.59 | 3.47 | PRIMED BOLERO (ORIGINAL LYRICS BOLERO) | love | love | 8.72 | 6.44 | 7.11 | PRIMED KOTO (ORIGINAL LYRICS KOTO) | spirituality | spirit | 7 | 5.56 | 5.82 | PRIMED POP (ORIGINAL LYRICS POP) | stress | stress | 2.09 | 7.45 | 3.93 | PRIMED SAMBA(ORIGINAL LYRICS SAMBA) | love | love | 8.72 | 6.44 | 7.11 |  |  |
| CUBA |  | CN = Congruent | movement | sad | romance | countrified | explosive | movement | rhythmic, melodic | goos sensation |  |  |  |  |  |  |  |  |  |  |  |  |  |  |  |  | 26 | PRIMED OPERA (Otiginal Lyrics Opera) | imagination | imagination | 6.98 | 5.83 | 5.84 | PRIMED FADO (ORIGINAL LYRICS FADO) | poetry | poetry | 5.86 | 4 | 5.31 | PRIMED HEAVY METAL (OIGINAL LYRICS HEAVY METAL) | pain | pain | 2.13 | 6.5 | 3.71 | PRIMED HIP HOP | unrestful | unrestful | #N/A | #N/A | #N/A | PRIMED BOLERO (ORIGINAL LYRICS BOLERO) | love | love | 8.72 | 6.44 | 7.11 | PRIMED KOTO (ORIGINAL LYRICS KOTO) | religeous | religion | 5.07 | 5.85 | 5.3 | PRIMED POP (ORIGINAL LYRICS POP) | land | land | 5.66 | 4.16 | 5.53 | PRIMED SAMBA(ORIGINAL LYRICS SAMBA) | love | love | 8.72 | 6.44 | 7.11 |  |  |
| CUBA |  | CN = Congruent | joy | sad,yearning | joy | disgust, boredom | agitated,fear | excitement | happy,joy | calm, relax |  | 5 |  | 1 |  | 4 |  | 2 |  | 1 |  | 6 |  | 3 |  | 6 | 27 | PRIMED OPERA (Otiginal Lyrics Opera) | curiosity | Curiosity | 6.74 | 6.08 | 5.46 | PRIMED FADO (ORIGINAL LYRICS FADO) | suffering | suffer | 1.72 | 6.13 | 2.54 | PRIMED HEAVY METAL (OIGINAL LYRICS HEAVY METAL) | digust | disgusting | 2.96 | 5.18 | 3.64 | PRIMED HIP HOP | uncomprehensible | uncomprehensible | #N/A | #N/A | #N/A | PRIMED BOLERO (ORIGINAL LYRICS BOLERO) | happy | happy | 8.21 | 6.49 | 6.63 | PRIMED KOTO (ORIGINAL LYRICS KOTO) | disinterested | disinterested | #N/A | #N/A | #N/A | PRIMED POP (ORIGINAL LYRICS POP) | diversion | diversion | #N/A | #N/A | #N/A | PRIMED SAMBA(ORIGINAL LYRICS SAMBA) | love | love | 8.72 | 6.44 | 7.11 |  |  |
| CUBA |  | CN = Congruent | freedom, peace | passion,love | love, patriotism | peace | out of control | noise | sensual | serious |  | 6 |  | 2 |  | 6 |  | 3 |  | 8 |  | 6 |  | 7 |  | 7 | 28 | PRIMED OPERA (Otiginal Lyrics Opera) | admiration | admiration | #N/A | #N/A | #N/A | PRIMED FADO (ORIGINAL LYRICS FADO) | passion | passion | 8.03 | 7.26 | 6.13 | PRIMED HEAVY METAL (OIGINAL LYRICS HEAVY METAL) | , disgust | disgusting | 2.96 | 5.18 | 3.64 | PRIMED HIP HOP | confusion | confusion | 3.46 | 6.07 | 3.04 | PRIMED BOLERO (ORIGINAL LYRICS BOLERO) | attraction | attractive | 7.49 | 6.76 | 5.54 | PRIMED KOTO (ORIGINAL LYRICS KOTO) | adoration | adoration | #N/A | #N/A | #N/A | PRIMED POP (ORIGINAL LYRICS POP) | mediocrity | mediocrity | #N/A | #N/A | #N/A | PRIMED SAMBA(ORIGINAL LYRICS SAMBA) | loneliness | loneliness | 1.61 | 4.56 | 2.51 |  |  |
| CUBA |  | CN = Congruent | love | peace, memories | dancing, singing | tradition, constumes | disorder, alteration | dancing | flavourful, fun | majestic |  | 7 |  | 3 |  | 1 |  | 5 |  | 5 |  | 5 |  | 3 |  | 6 | 29 | PRIMED OPERA (Otiginal Lyrics Opera) | love | love | 8.72 | 6.44 | 7.11 | PRIMED FADO (ORIGINAL LYRICS FADO) | yearning | yearning | #N/A | #N/A | #N/A | PRIMED HEAVY METAL (OIGINAL LYRICS HEAVY METAL) | fear | fear | 2.76 | 6.96 | 3.22 | PRIMED HIP HOP | mercy | mercy | 5.71 | 5.43 | 3.7 | PRIMED BOLERO (ORIGINAL LYRICS BOLERO) | sad | sad | 1.61 | 4.13 | 3.45 | PRIMED KOTO (ORIGINAL LYRICS KOTO) | gods | gods | #N/A | #N/A | #N/A | PRIMED POP (ORIGINAL LYRICS POP) | will | will | #N/A | #N/A | #N/A | PRIMED SAMBA(ORIGINAL LYRICS SAMBA) | insecurity | insecure | 2.36 | 5.56 | 2.33 |  |  |
| CUBA |  | CN = Congruent | happy | sad | passion | Allarming | madness | rhythm | fun | relax |  | 6 |  | 3 |  | 7 |  | 5 |  | 2 |  | 6 |  | 4 |  | 7 | 30 | PRIMED OPERA (Otiginal Lyrics Opera) | beauty | beauty | 7.82 | 4.95 | 5.53 | PRIMED FADO (ORIGINAL LYRICS FADO) | sad | sad | 1.61 | 4.13 | 3.45 | PRIMED HEAVY METAL (OIGINAL LYRICS HEAVY METAL) | violence | violence | #N/A | #N/A | #N/A | PRIMED HIP HOP |  | #N/A | #N/A | #N/A | #N/A | PRIMED BOLERO (ORIGINAL LYRICS BOLERO) | love | love | 8.72 | 6.44 | 7.11 | PRIMED KOTO (ORIGINAL LYRICS KOTO) | religeous | religion | 5.07 | 5.85 | 5.3 | PRIMED POP (ORIGINAL LYRICS POP) | violence | violence | #N/A | #N/A | #N/A | PRIMED SAMBA(ORIGINAL LYRICS SAMBA) | passion | passion | 8.03 | 7.26 | 6.13 |  |  |
| CUBA |  | CN = Congruent | joy | sad | flavourful, sad | sad, pride | joy | speculation | pleasurable | impressive |  |  |  | 6 |  |  |  |  |  |  |  |  |  |  |  |  | 31 | PRIMED OPERA (Otiginal Lyrics Opera) | excitement | excitement | 7.5 | 7.67 | 6.18 | PRIMED FADO (ORIGINAL LYRICS FADO) | sad | sad | 1.61 | 4.13 | 3.45 | PRIMED HEAVY METAL (OIGINAL LYRICS HEAVY METAL) | fury | fury | 3.1 | 6.82 | 4.29 | PRIMED HIP HOP | sad | sad | 1.61 | 4.13 | 3.45 | PRIMED BOLERO (ORIGINAL LYRICS BOLERO) | romance | Romance | 7.61 | 6.9 | 6.03 | PRIMED KOTO (ORIGINAL LYRICS KOTO) | laughter | laughter | 8.45 | 6.75 | 6.45 | PRIMED POP (ORIGINAL LYRICS POP) | desperation | despair | 2.99 | 4.49 | 4.3 | PRIMED SAMBA(ORIGINAL LYRICS SAMBA) | flavourful | flavourful | #N/A | #N/A | #N/A |  |  |
| CUBA |  | CN = Congruent | joy | love | happy | concentration | madness | aggressive | gangs | admiration |  | 5 |  | 1 |  | 6 |  | 5 |  | 1 |  | 6 |  | 2 |  | 6 | 32 | PRIMED OPERA (Otiginal Lyrics Opera) | pride | Pride | 7 | 5.83 | 7.06 | PRIMED FADO (ORIGINAL LYRICS FADO) | happy | happy | 8.21 | 6.49 | 6.63 | PRIMED HEAVY METAL (OIGINAL LYRICS HEAVY METAL) | tragedy | tragedy | 1.78 | 6.24 | 3.5 | PRIMED HIP HOP | suffering | suffer | 1.72 | 6.13 | 2.54 | PRIMED BOLERO (ORIGINAL LYRICS BOLERO) | attraction | attractive | 7.49 | 6.76 | 5.54 | PRIMED KOTO (ORIGINAL LYRICS KOTO) | nothing | nothing | #N/A | #N/A | #N/A | PRIMED POP (ORIGINAL LYRICS POP) | sorrow | sorrow | 2.32 | 4.48 | 3.67 | PRIMED SAMBA(ORIGINAL LYRICS SAMBA) | love | love | 8.72 | 6.44 | 7.11 |  |  |
| CUBA |  | CN = Congruent | festive | feeling | flavourful | meditation | stress | hip | \* | religeous |  | 6 |  | 1 |  |  |  | 5 |  | 1 |  | 6 |  | 5 |  | 6 | 33 | PRIMED OPERA (Otiginal Lyrics Opera) | pride | Pride | 7 | 5.83 | 7.06 | PRIMED FADO (ORIGINAL LYRICS FADO) | patriotism | patriotism | 6.85 | 5.56 | 6.52 | PRIMED HEAVY METAL (OIGINAL LYRICS HEAVY METAL) |  | #N/A | #N/A | #N/A | #N/A | PRIMED HIP HOP |  | #N/A | #N/A | #N/A | #N/A | PRIMED BOLERO (ORIGINAL LYRICS BOLERO) |  | #N/A | #N/A | #N/A | #N/A | PRIMED KOTO (ORIGINAL LYRICS KOTO) |  | #N/A | #N/A | #N/A | #N/A | PRIMED POP (ORIGINAL LYRICS POP) |  | #N/A | #N/A | #N/A | #N/A | PRIMED SAMBA(ORIGINAL LYRICS SAMBA) |  | #N/A | #N/A | #N/A | #N/A |  |  |
| CUBA |  | CN = Congruent | joy, intimate | sad | wailing | religeous, relax | madness | rebellious | party | relax |  | 6 |  | 1 |  | 7 |  | 5 |  | 8 |  | 6 |  | 7 |  | 7 | 34 | PRIMED OPERA (Otiginal Lyrics Opera) | | #N/A | #N/A | #N/A | #N/A | PRIMED FADO (ORIGINAL LYRICS FADO) | praise | praise | 7.24 | 6.28 | 6.59 | PRIMED HEAVY METAL (OIGINAL LYRICS HEAVY METAL) | madness | madness | 4.37 | 5.8 | 4.53 | PRIMED HIP HOP | tragedy | tragedy | 1.78 | 6.24 | 3.5 | PRIMED BOLERO (ORIGINAL LYRICS BOLERO) | heartbreak | heartbreak | 1.93 | 5.8 | 3.11 | PRIMED KOTO (ORIGINAL LYRICS KOTO) | religion | 0 | #N/A | #N/A | #N/A | PRIMED POP (ORIGINAL LYRICS POP) | rebellion | rebellious | 4.86 | 5.82 | 6.28 | PRIMED SAMBA(ORIGINAL LYRICS SAMBA) | love | love | 8.72 | 6.44 | 7.11 |  |  |
| CUBA |  | CN = Congruent | joy | dedication | flavourful | meditation | madness | social criticism | dancing | serene |  | 6 |  | 4 |  | 7 |  | 6 |  | 4 |  | 5 |  | 5 |  | 7 | 35 | PRIMED OPERA (Otiginal Lyrics Opera) | pride | Pride | 7 | 5.83 | 7.06 | PRIMED FADO (ORIGINAL LYRICS FADO) | patriotism | patriotism | 6.85 | 5.56 | 6.52 | PRIMED HEAVY METAL (OIGINAL LYRICS HEAVY METAL) | madness | madness | 4.37 | 5.8 | 4.53 | PRIMED HIP HOP | street life | street life | #N/A | #N/A | #N/A | PRIMED BOLERO (ORIGINAL LYRICS BOLERO) | love, attraction | love | 8.72 | 6.44 | 7.11 | PRIMED KOTO (ORIGINAL LYRICS KOTO) | honour | honour | #N/A | #N/A | #N/A | PRIMED POP (ORIGINAL LYRICS POP) | freedom | freedom | 7.58 | 5.52 | 6.76 | PRIMED SAMBA(ORIGINAL LYRICS SAMBA) | love | love | 8.72 | 6.44 | 7.11 |  |  |
| CUBA |  | CN = Congruent | joy, feast | feeling | love | pain | stress | adrenaline | dancing | dreaming |  | 6 |  | 1 |  | 6 |  | 8 |  | 8 |  | 4 |  | 7 |  | 7 | 36 | PRIMED OPERA (Otiginal Lyrics Opera) | beauty | beauty | 7.82 | 4.95 | 5.53 | PRIMED FADO (ORIGINAL LYRICS FADO) | | #N/A | #N/A | #N/A | #N/A | PRIMED HEAVY METAL (OIGINAL LYRICS HEAVY METAL) |  | #N/A | #N/A | #N/A | #N/A | PRIMED HIP HOP | violent | violent | 2.29 | 6.89 | 5.16 | PRIMED BOLERO (ORIGINAL LYRICS BOLERO) | love | love | 8.72 | 6.44 | 7.11 | PRIMED KOTO (ORIGINAL LYRICS KOTO) |  | #N/A | #N/A | #N/A | #N/A | PRIMED POP (ORIGINAL LYRICS POP) |  | #N/A | #N/A | #N/A | #N/A | PRIMED SAMBA(ORIGINAL LYRICS SAMBA) |  | #N/A | #N/A | #N/A | #N/A |  |  |
| CUBA |  | CN = Congruent | movement, tranquility | calma, nostalgia | dancing, passion | relax | rhythmic | violence, euphoria | joy | peace |  | 6 |  | 4 |  | 6 |  | 4 |  | 8 |  | 6 |  | 7 |  | 7 | 37 | PRIMED OPERA (Otiginal Lyrics Opera) | sensual | Sensual | #N/A | #N/A | #N/A | PRIMED FADO (ORIGINAL LYRICS FADO) | patriotism | patriotism | 6.85 | 5.56 | 6.52 | PRIMED HEAVY METAL (OIGINAL LYRICS HEAVY METAL) | egoism | egoism | #N/A | #N/A | #N/A | PRIMED HIP HOP | violent | violent | 2.29 | 6.89 | 5.16 | PRIMED BOLERO (ORIGINAL LYRICS BOLERO) | love | love | 8.72 | 6.44 | 7.11 | PRIMED KOTO (ORIGINAL LYRICS KOTO) | spirituality | spirit | 7 | 5.56 | 5.82 | PRIMED POP (ORIGINAL LYRICS POP) | immaturity | immaturity | #N/A | #N/A | #N/A | PRIMED SAMBA(ORIGINAL LYRICS SAMBA) | fear | fear | 2.76 | 6.96 | 3.22 |  |  |
| CUBA |  | I = Incongruent | carnival | singing | contagious | peace | strong | fast | rhythmic | peace |  | 6 |  | 4 |  | 6 |  | 6 |  | 8 |  | 6 |  | 8 |  | 7 | 38 | PRIMED POP (ORIGINAL LYRICS OPERA) | popular | popular | #N/A | #N/A | #N/A | PRIMED OPERA (ORIGINAL LYRICS FADO) | | #N/A | #N/A | #N/A | #N/A | PRIMED CUBAN SON (ORIGINAL LYRICS HEAVY METAL) | sad | sad | 1.61 | 4.13 | 3.45 | PRIMED SAMBA (ORIGINAL LYRICS HIP HOP) |  | Helplessness | #N/A | #N/A | #N/A | PRIMED JAPANESE TRADITIONAL (ORIGINAL LYRICS BOLERO) |  | sadness | 2.21 | 5.21 | 2.82 | PRIMED HEAVY METAL (ORIGINAL LYRICS KOTO) |  | Reflection | 5.97 | 4.38 | 6.1 | PRIMED FADO (ORIGINAL LYRICS POP) |  | anger | 2.34 | 7.63 | 5.5 | PRIMED HIP HOP (ORIGINAL LYRICS SAMBA) |  | #N/A | #N/A | #N/A | #N/A |  |  |
| CUBA |  | I = Incongruent | joy,relax | relax, meditation | patriotism | nothing | protest, rebellion | protest | uplifting | spiritual |  | 6 |  | 4 |  | 5 |  | 3 |  | 8 |  | 6 |  | 6 |  | 4 | 39 | PRIMED POP (ORIGINAL LYRICS OPERA) | vanity | Vanity | 4.3 | 4.98 | 4.8 | PRIMED OPERA (ORIGINAL LYRICS FADO) | happy | happy | 8.21 | 6.49 | 6.63 | PRIMED CUBAN SON (ORIGINAL LYRICS HEAVY METAL) | sad | sad | 1.61 | 4.13 | 3.45 | PRIMED SAMBA (ORIGINAL LYRICS HIP HOP) | sad | sad | 1.61 | 4.13 | 3.45 | PRIMED JAPANESE TRADITIONAL (ORIGINAL LYRICS BOLERO) | pain | pain | 2.13 | 6.5 | 3.71 | PRIMED HEAVY METAL (ORIGINAL LYRICS KOTO) | torture | torture | 1.56 | 6.1 | 3.33 | PRIMED FADO (ORIGINAL LYRICS POP) | fear | fear | 2.76 | 6.96 | 3.22 | PRIMED HIP HOP (ORIGINAL LYRICS SAMBA) | depression | depression | 1.85 | 4.54 | 2.91 |  |  |
| CUBA |  | I = Incongruent | fun | calm,peace | dancing, movement | spiritual, religeous | fury | dancing | relax, joy | calm, peace |  | 7 |  | 5 |  | 7 |  | 6 |  | 8 |  | 1 |  | 7 |  | 3 | 40 | PRIMED POP (ORIGINAL LYRICS OPERA) | seduction | seduction | 7.53 | 7.43 | 5.63 | PRIMED OPERA (ORIGINAL LYRICS FADO) | sad | sad | 1.61 | 4.13 | 3.45 | PRIMED CUBAN SON (ORIGINAL LYRICS HEAVY METAL) | sad | sad | 1.61 | 4.13 | 3.45 | PRIMED SAMBA (ORIGINAL LYRICS HIP HOP) | sad | sad | 1.61 | 4.13 | 3.45 | PRIMED JAPANESE TRADITIONAL (ORIGINAL LYRICS BOLERO) | attraction | attractive | 7.49 | 6.76 | 5.54 | PRIMED HEAVY METAL (ORIGINAL LYRICS KOTO) | religeous | religion | 5.07 | 5.85 | 5.3 | PRIMED FADO (ORIGINAL LYRICS POP) | alienation | alienation | 2.75 | 4.45 | 3.25 | PRIMED HIP HOP (ORIGINAL LYRICS SAMBA) | love | love | 8.72 | 6.44 | 7.11 |  |  |
| CUBA |  | I = Incongruent | party | feeling | the best | indegenous | war | original | joy | glorious |  | 6 |  | 1 |  | 3 |  | 6 |  | 7 |  | 6 |  | 7 |  | 6 | 41 | PRIMED POP (ORIGINAL LYRICS OPERA) | | #N/A | #N/A | #N/A | #N/A | PRIMED OPERA (ORIGINAL LYRICS FADO) | suffering | suffer | 1.72 | 6.13 | 2.54 | PRIMED CUBAN SON (ORIGINAL LYRICS HEAVY METAL) |  | #N/A | #N/A | #N/A | #N/A | PRIMED SAMBA (ORIGINAL LYRICS HIP HOP) |  | #N/A | #N/A | #N/A | #N/A | PRIMED JAPANESE TRADITIONAL (ORIGINAL LYRICS BOLERO) | love | love | 8.72 | 6.44 | 7.11 | PRIMED HEAVY METAL (ORIGINAL LYRICS KOTO) | confusion | confusion | 3.46 | 6.07 | 3.04 | PRIMED FADO (ORIGINAL LYRICS POP) | confusion | confusion | 3.46 | 6.07 | 3.04 | PRIMED HIP HOP (ORIGINAL LYRICS SAMBA) | sad | sad | 1.61 | 4.13 | 3.45 |  |  |
| CUBA |  | I = Incongruent | passion | melancholy | sensual, erotic | grief, deep | freedom, thick | defiance, critique | fresh, dynamic | spiritual, peace |  | 6 |  | 7 |  | 6 |  | 7 |  | 7 |  | 6 |  | 7 |  | 7 | 42 | PRIMED POP (ORIGINAL LYRICS OPERA) | flirting | flirting | 7.04 | 7.03 | 5.48 | PRIMED OPERA (ORIGINAL LYRICS FADO) | patriotism | patriotism | 6.85 | 5.56 | 6.52 | PRIMED CUBAN SON (ORIGINAL LYRICS HEAVY METAL) | dissapointment | disappointment | 2.37 | 4.6 | 3.2 | PRIMED SAMBA (ORIGINAL LYRICS HIP HOP) | desperation | despair | 2.99 | 4.49 | 4.3 | PRIMED JAPANESE TRADITIONAL (ORIGINAL LYRICS BOLERO) | pain | pain | 2.13 | 6.5 | 3.71 | PRIMED HEAVY METAL (ORIGINAL LYRICS KOTO) | spiritual | spirit | 7 | 5.56 | 5.82 | PRIMED FADO (ORIGINAL LYRICS POP) | critique | critique | 4.39 | 5.37 | 4.07 | PRIMED HIP HOP (ORIGINAL LYRICS SAMBA) | regret | Regret | 2.26 | 5.67 | 3.23 |  |  |
| CUBA |  | I = Incongruent | joy | feeling, melancholy | love, feeling | meditation | madness, ecstasy, | dancing, romantic | joy | relax |  | 6 |  | 1 |  | 6 |  | 6 |  | 1 |  | 6 |  | 7 |  | 7 | 43 | PRIMED POP (ORIGINAL LYRICS OPERA) | happy | happy | 8.21 | 6.49 | 6.63 | PRIMED OPERA (ORIGINAL LYRICS FADO) | suffering | suffer | 1.72 | 6.13 | 2.54 | PRIMED CUBAN SON (ORIGINAL LYRICS HEAVY METAL) | violent | violent | 2.29 | 6.89 | 5.16 | PRIMED SAMBA (ORIGINAL LYRICS HIP HOP) | longing | Longing | #N/A | #N/A | #N/A | PRIMED JAPANESE TRADITIONAL (ORIGINAL LYRICS BOLERO) | love | love | 8.72 | 6.44 | 7.11 | PRIMED HEAVY METAL (ORIGINAL LYRICS KOTO) | attraction | attractive | 7.49 | 6.76 | 5.54 | PRIMED FADO (ORIGINAL LYRICS POP) | decisive | decisive | #N/A | #N/A | #N/A | PRIMED HIP HOP (ORIGINAL LYRICS SAMBA) | fear | fear | 2.76 | 6.96 | 3.22 |  |  |
| CUBA |  | I = Incongruent | joy | calm | pride |  | disturbance | dancing | dancing | religeous, peace |  |  |  |  |  |  |  |  |  |  |  | 6 |  |  |  | 7 | 44 | PRIMED POP (ORIGINAL LYRICS OPERA) | | #N/A | #N/A | #N/A | #N/A | PRIMED OPERA (ORIGINAL LYRICS FADO) | | #N/A | #N/A | #N/A | #N/A | PRIMED CUBAN SON (ORIGINAL LYRICS HEAVY METAL) | violent | violent | 2.29 | 6.89 | 5.16 | PRIMED SAMBA (ORIGINAL LYRICS HIP HOP) | madness | madness | 4.37 | 5.8 | 4.53 | PRIMED JAPANESE TRADITIONAL (ORIGINAL LYRICS BOLERO) | love | love | 8.72 | 6.44 | 7.11 | PRIMED HEAVY METAL (ORIGINAL LYRICS KOTO) | torture | torture | 1.56 | 6.1 | 3.33 | PRIMED FADO (ORIGINAL LYRICS POP) | freedom | freedom | 7.58 | 5.52 | 6.76 | PRIMED HIP HOP (ORIGINAL LYRICS SAMBA) | desperation | despair | 2.99 | 4.49 | 4.3 |  |  |
| CUBA |  | I = Incongruent | pleasing | calm, peace | nostalgia | peace, meditation | discomfort, madness | restleness | joy | deep | I like the harmony | 6 |  | 1 |  | 4 |  | 3 |  | 8 |  | 6 |  | 7 | I like the rhythms | 7 | 45 | PRIMED POP (ORIGINAL LYRICS OPERA) | love | love | 8.72 | 6.44 | 7.11 | PRIMED OPERA (ORIGINAL LYRICS FADO) | sad | sad | 1.61 | 4.13 | 3.45 | PRIMED CUBAN SON (ORIGINAL LYRICS HEAVY METAL) | beauty | beauty | 7.82 | 4.95 | 5.53 | PRIMED SAMBA (ORIGINAL LYRICS HIP HOP) | pain | pain | 2.13 | 6.5 | 3.71 | PRIMED JAPANESE TRADITIONAL (ORIGINAL LYRICS BOLERO) | agony | agony | 2.43 | 6.06 | 4.02 | PRIMED HEAVY METAL (ORIGINAL LYRICS KOTO) | nerveous | nerveous | #N/A | #N/A | #N/A | PRIMED FADO (ORIGINAL LYRICS POP) | obsurd | obsurd | #N/A | #N/A | #N/A | PRIMED HIP HOP (ORIGINAL LYRICS SAMBA) | freedom | freedom | 7.58 | 5.52 | 6.76 |  |  |
| CUBA |  | I = Incongruent | relax | nostalgia | singing | boredom | jumping, yelling | dancing |  | passion, optimism, victory |  |  |  |  | I listen to this music more than 20 hours a week | 6 |  |  |  |  |  |  |  |  |  |  | 46 | PRIMED POP (ORIGINAL LYRICS OPERA) | emptiness | emptiness | #N/A | #N/A | #N/A | PRIMED OPERA (ORIGINAL LYRICS FADO) | sad | sad | 1.61 | 4.13 | 3.45 | PRIMED CUBAN SON (ORIGINAL LYRICS HEAVY METAL) | sad | sad | 1.61 | 4.13 | 3.45 | PRIMED SAMBA (ORIGINAL LYRICS HIP HOP) | confusion | confusion | 3.46 | 6.07 | 3.04 | PRIMED JAPANESE TRADITIONAL (ORIGINAL LYRICS BOLERO) | surprise | Surprise | 7.73 | 7.07 | 3.87 | PRIMED HEAVY METAL (ORIGINAL LYRICS KOTO) | fury | fury | 3.1 | 6.82 | 4.29 | PRIMED FADO (ORIGINAL LYRICS POP) | inquisitive | inquisitive | #N/A | #N/A | #N/A | PRIMED HIP HOP (ORIGINAL LYRICS SAMBA) | emptiness | emptiness | #N/A | #N/A | #N/A |  |  |
| CUBA |  | I = Incongruent | swing | sad, melancholy | rhythmic, gentle | gracious, meditation | noise, madness | sexual, energetic | sensual, swing | calm, fantastic |  | 6 |  | 1 |  | 6 |  | 7 |  | 7 |  | 1 |  | 5 |  | 6 | 47 | PRIMED POP (ORIGINAL LYRICS OPERA) | prostitution | prostitution | #N/A | #N/A | #N/A | PRIMED OPERA (ORIGINAL LYRICS FADO) | patriotism | patriotism | 6.85 | 5.56 | 6.52 | PRIMED CUBAN SON (ORIGINAL LYRICS HEAVY METAL) |  | #N/A | #N/A | #N/A | #N/A | PRIMED SAMBA (ORIGINAL LYRICS HIP HOP) |  | #N/A | #N/A | #N/A | #N/A | PRIMED JAPANESE TRADITIONAL (ORIGINAL LYRICS BOLERO) |  | #N/A | #N/A | #N/A | #N/A | PRIMED HEAVY METAL (ORIGINAL LYRICS KOTO) |  | #N/A | #N/A | #N/A | #N/A | PRIMED FADO (ORIGINAL LYRICS POP) |  | #N/A | #N/A | #N/A | #N/A | PRIMED HIP HOP (ORIGINAL LYRICS SAMBA) |  | #N/A | #N/A | #N/A | #N/A |  |  |
| CUBA |  | I = Incongruent | joy | sad | joy,love | tenderness | anger, disturbance | energy | dancing, jumping | Flying, hope, strength |  | 6 |  | 8 |  | 7 |  | 7 |  | 5 | I know it well | 6 |  | 6 | I know it well | 7 | 48 | PRIMED POP (ORIGINAL LYRICS OPERA) | sensual | Sensual | #N/A | #N/A | #N/A | PRIMED OPERA (ORIGINAL LYRICS FADO) | heroism | heroism | #N/A | #N/A | #N/A | PRIMED CUBAN SON (ORIGINAL LYRICS HEAVY METAL) | sad | sad | 1.61 | 4.13 | 3.45 | PRIMED SAMBA (ORIGINAL LYRICS HIP HOP) | sad | sad | 1.61 | 4.13 | 3.45 | PRIMED JAPANESE TRADITIONAL (ORIGINAL LYRICS BOLERO) | sad | sad | 1.61 | 4.13 | 3.45 | PRIMED HEAVY METAL (ORIGINAL LYRICS KOTO) | fury | fury | 3.1 | 6.82 | 4.29 | PRIMED FADO (ORIGINAL LYRICS POP) | violence | violence | #N/A | #N/A | #N/A | PRIMED HIP HOP (ORIGINAL LYRICS SAMBA) | loveless | loveless | #N/A | #N/A | #N/A |  |  |
| CUBA |  | I = Incongruent | joy | sad | charming, passionate | torment | darkness | rhythm | romantic, sensual | peace |  | 6 |  | 3 |  | 6 |  | 6 |  | 3 |  | 6 |  | 5 |  | 7 | 49 | PRIMED POP (ORIGINAL LYRICS OPERA) | vanity | Vanity | 4.3 | 4.98 | 4.8 | PRIMED OPERA (ORIGINAL LYRICS FADO) | patriotism | patriotism | 6.85 | 5.56 | 6.52 | PRIMED CUBAN SON (ORIGINAL LYRICS HEAVY METAL) | passion | passion | 8.03 | 7.26 | 6.13 | PRIMED SAMBA (ORIGINAL LYRICS HIP HOP) | passion | passion | 8.03 | 7.26 | 6.13 | PRIMED JAPANESE TRADITIONAL (ORIGINAL LYRICS BOLERO) | sad | sad | 1.61 | 4.13 | 3.45 | PRIMED HEAVY METAL (ORIGINAL LYRICS KOTO) | anger | anger | 2.34 | 7.63 | 5.5 | PRIMED FADO (ORIGINAL LYRICS POP) | poetic | Poetic | #N/A | #N/A | #N/A | PRIMED HIP HOP (ORIGINAL LYRICS SAMBA) | passion | passion | 8.03 | 7.26 | 6.13 |  |  |
| CUBA |  | I = Incongruent | rhythmic | poetry | flavourful | madness music | madness | street music | pleasant | serene |  | 6 |  | 5 |  | 2 |  |  |  | 8 |  | 4 |  | 4 |  | 1 | 50 | PRIMED POP (ORIGINAL LYRICS OPERA) | | #N/A | #N/A | #N/A | #N/A | PRIMED OPERA (ORIGINAL LYRICS FADO) | love | love | 8.72 | 6.44 | 7.11 | PRIMED CUBAN SON (ORIGINAL LYRICS HEAVY METAL) | passion | passion | 8.03 | 7.26 | 6.13 | PRIMED SAMBA (ORIGINAL LYRICS HIP HOP) | passion | passion | 8.03 | 7.26 | 6.13 | PRIMED JAPANESE TRADITIONAL (ORIGINAL LYRICS BOLERO) | love | love | 8.72 | 6.44 | 7.11 | PRIMED HEAVY METAL (ORIGINAL LYRICS KOTO) | madness | madness | 4.37 | 5.8 | 4.53 | PRIMED FADO (ORIGINAL LYRICS POP) | poetic | Poetic | #N/A | #N/A | #N/A | PRIMED HIP HOP (ORIGINAL LYRICS SAMBA) | violence | violence | #N/A | #N/A | #N/A |  |  |
| CUBA |  | I = Incongruent | joy | calm | identity | peace, serene | darkness | dancing | dancing, singing | relax | I play this music a lot | 6 |  | 3 | I like it | 6 | I like it | 5 |  | 5 | I play it a fair bit | 4 |  | 7 |  | 7 | 51 | PRIMED POP (ORIGINAL LYRICS OPERA) | love | love | 8.72 | 6.44 | 7.11 | PRIMED OPERA (ORIGINAL LYRICS FADO) | patriotism | patriotism | 6.85 | 5.56 | 6.52 | PRIMED CUBAN SON (ORIGINAL LYRICS HEAVY METAL) | remorse, sad | sad | 1.61 | 4.13 | 3.45 | PRIMED SAMBA (ORIGINAL LYRICS HIP HOP) | sad, despair | sad | 1.61 | 4.13 | 3.45 | PRIMED JAPANESE TRADITIONAL (ORIGINAL LYRICS BOLERO) | love | love | 8.72 | 6.44 | 7.11 | PRIMED HEAVY METAL (ORIGINAL LYRICS KOTO) | faith | faith | 6.57 | 5.73 | 5.6 | PRIMED FADO (ORIGINAL LYRICS POP) | suicide | suicide | 1.25 | 5.73 | 3.58 | PRIMED HIP HOP (ORIGINAL LYRICS SAMBA) | poetry | poetry | 5.86 | 4 | 5.31 |  |  |
| CUBA |  | I = Incongruent | nostalgia | sad | romance, love | reflection, peace | madness | violence | sensual | emotive |  | 5 |  | 1 |  | 7 |  | 1 |  | 8 |  | 6 |  | 7 |  | 7 | 52 | PRIMED POP (ORIGINAL LYRICS OPERA) | playful | playful | 6.54 | 5.63 | 5.78 | PRIMED OPERA (ORIGINAL LYRICS FADO) | sad | sad | 1.61 | 4.13 | 3.45 | PRIMED CUBAN SON (ORIGINAL LYRICS HEAVY METAL) | sad | sad | 1.61 | 4.13 | 3.45 | PRIMED SAMBA (ORIGINAL LYRICS HIP HOP) | desperation | despair | 2.99 | 4.49 | 4.3 | PRIMED JAPANESE TRADITIONAL (ORIGINAL LYRICS BOLERO) | sad | sad | 1.61 | 4.13 | 3.45 | PRIMED HEAVY METAL (ORIGINAL LYRICS KOTO) | death, madness | death, madness | #N/A | #N/A | #N/A | PRIMED FADO (ORIGINAL LYRICS POP) | freedom | freedom | 7.58 | 5.52 | 6.76 | PRIMED HIP HOP (ORIGINAL LYRICS SAMBA) | decisive | decisive | #N/A | #N/A | #N/A |  |  |
| CUBA |  | I = Incongruent | joy, love | sad, peace | joy, peaceful | suffering, dream | alteration, joy | alteration | peace, joy | calm, fear | I listen to it nearly everyday | 6 | I listen to it a bit | 5 |  | 6 |  | 6 | I listen to it very little | 2 | My work is a classical musician | 6 |  | 5 | I play this music | 7 | 53 | PRIMED POP (ORIGINAL LYRICS OPERA) | love | love | 8.72 | 6.44 | 7.11 | PRIMED OPERA (ORIGINAL LYRICS FADO) | suffering | suffer | 1.72 | 6.13 | 2.54 | PRIMED CUBAN SON (ORIGINAL LYRICS HEAVY METAL) | sad, religious | sad | 1.61 | 4.13 | 3.45 | PRIMED SAMBA (ORIGINAL LYRICS HIP HOP) | grief, passion | passion | 8.03 | 7.26 | 6.13 | PRIMED JAPANESE TRADITIONAL (ORIGINAL LYRICS BOLERO) | love | love | 8.72 | 6.44 | 7.11 | PRIMED HEAVY METAL (ORIGINAL LYRICS KOTO) | god | god | 8.15 | 5.95 | 5.88 | PRIMED FADO (ORIGINAL LYRICS POP) | deep | Deep | 5.08 | 4.55 | 5.37 | PRIMED HIP HOP (ORIGINAL LYRICS SAMBA) | sad | sad | 1.61 | 4.13 | 3.45 |  |  |
| CUBA |  | I = Incongruent | joy, romance | sad | dancing, love | dream, tranquility | madness, torment | delinquency, violence | friendship, dancing | love, tranquility |  | 6 |  | 8 |  | 8 |  | 5 |  | 8 |  | 5 |  | 7 |  | 7 | 54 | PRIMED POP (ORIGINAL LYRICS OPERA) | pride | Pride | 7 | 5.83 | 7.06 | PRIMED OPERA (ORIGINAL LYRICS FADO) | patriotism | patriotism | 6.85 | 5.56 | 6.52 | PRIMED CUBAN SON (ORIGINAL LYRICS HEAVY METAL) | death | Death | 1.61 | 4.59 | 3.47 | PRIMED SAMBA (ORIGINAL LYRICS HIP HOP) | killing | killing | #N/A | #N/A | #N/A | PRIMED JAPANESE TRADITIONAL (ORIGINAL LYRICS BOLERO) | deceit | deceit | 2.9 | 5.68 | 3.95 | PRIMED HEAVY METAL (ORIGINAL LYRICS KOTO) | religion | 0 | #N/A | #N/A | #N/A | PRIMED FADO (ORIGINAL LYRICS POP) | attraction | attractive | 7.49 | 6.76 | 5.54 | PRIMED HIP HOP (ORIGINAL LYRICS SAMBA) | charisma | charisma | 7.15 | 5.75 | 6.05 |  |  |
| CUBA |  | I = Incongruent | carnival | sad | rhythm | heartbreaking music | noise | street music | refreshing, relax | expressive, relax |  | 6 |  | 3 |  | 6 |  | 2 |  | 8 |  | 6 |  | 2 |  | 7 | 55 | PRIMED POP (ORIGINAL LYRICS OPERA) | love | love | 8.72 | 6.44 | 7.11 | PRIMED OPERA (ORIGINAL LYRICS FADO) | poetic | Poetic | #N/A | #N/A | #N/A | PRIMED CUBAN SON (ORIGINAL LYRICS HEAVY METAL) | sad, regret | sad | 1.61 | 4.13 | 3.45 | PRIMED SAMBA (ORIGINAL LYRICS HIP HOP) | passion, poetry | passion | 8.03 | 7.26 | 6.13 | PRIMED JAPANESE TRADITIONAL (ORIGINAL LYRICS BOLERO) | love | love | 8.72 | 6.44 | 7.11 | PRIMED HEAVY METAL (ORIGINAL LYRICS KOTO) | wishing | wishing | #N/A | #N/A | #N/A | PRIMED FADO (ORIGINAL LYRICS POP) | laughter | laughter | 8.45 | 6.75 | 6.45 | PRIMED HIP HOP (ORIGINAL LYRICS SAMBA) | violence | violence | #N/A | #N/A | #N/A |  |  |
| CUBA |  | I = Incongruent | joy, nostalgia | sad | joy, peaceful | boredom | energy | dancing | nice | nice |  | 6 | This is my favourite music | 6 |  | 6 |  | 5 |  | 8 |  | 6 |  | 2 |  | 7 | 56 | PRIMED POP (ORIGINAL LYRICS OPERA) | love, pride | love | 8.72 | 6.44 | 7.11 | PRIMED OPERA (ORIGINAL LYRICS FADO) | | #N/A | #N/A | #N/A | #N/A | PRIMED CUBAN SON (ORIGINAL LYRICS HEAVY METAL) | sad | sad | 1.61 | 4.13 | 3.45 | PRIMED SAMBA (ORIGINAL LYRICS HIP HOP) | passion | passion | 8.03 | 7.26 | 6.13 | PRIMED JAPANESE TRADITIONAL (ORIGINAL LYRICS BOLERO) | attraction | attractive | 7.49 | 6.76 | 5.54 | PRIMED HEAVY METAL (ORIGINAL LYRICS KOTO) | spiritual | spirit | 7 | 5.56 | 5.82 | PRIMED FADO (ORIGINAL LYRICS POP) |  | #N/A | #N/A | #N/A | #N/A | PRIMED HIP HOP (ORIGINAL LYRICS SAMBA) | nostalgia | nostalgia | #N/A | #N/A | #N/A |  |  |
| CUBA |  | I = Incongruent | freedom | nostalgia | peace | spiritual | disease, sick, persecution | madness | joy | peace | I like to listen to it at home | 6 | I don't like it | 3 | It makes me move | 6 | Sometimes I listen to it | 6 | I don't like it | 3 | I work with this music | 6 | I like it but don't listen to it | 4 | I work with this music | 7 | 57 | PRIMED POP (ORIGINAL LYRICS OPERA) | love, sensual | love | 8.72 | 6.44 | 7.11 | PRIMED OPERA (ORIGINAL LYRICS FADO) | sad | sad | 1.61 | 4.13 | 3.45 | PRIMED CUBAN SON (ORIGINAL LYRICS HEAVY METAL) | peace | peace | 7.72 | 2.95 | 5.45 | PRIMED SAMBA (ORIGINAL LYRICS HIP HOP) | passion, desire | passion | 8.03 | 7.26 | 6.13 | PRIMED JAPANESE TRADITIONAL (ORIGINAL LYRICS BOLERO) | anguish | Anguish | #N/A | #N/A | #N/A | PRIMED HEAVY METAL (ORIGINAL LYRICS KOTO) | torture | torture | 1.56 | 6.1 | 3.33 | PRIMED FADO (ORIGINAL LYRICS POP) | angst | Angst | #N/A | #N/A | #N/A | PRIMED HIP HOP (ORIGINAL LYRICS SAMBA) | shame | shame | 2.13 | 6.33 | 2.97 |  |  |
| CUBA |  | I = Incongruent | dancing, africa | yearning | pertinence | elegant, precise | fury, madness, anger | rhythm | relax, fun | liberation, spiritual |  | 6 |  | 2 |  | 6 | I avoid this kind of music | 1 |  | 8 |  | 6 |  | 5 |  | 7 | 58 | PRIMED POP (ORIGINAL LYRICS OPERA) | love, want | love | 8.72 | 6.44 | 7.11 | PRIMED OPERA (ORIGINAL LYRICS FADO) | pertinence | pertinence | #N/A | #N/A | #N/A | PRIMED CUBAN SON (ORIGINAL LYRICS HEAVY METAL) | memories, sad | sad | 1.61 | 4.13 | 3.45 | PRIMED SAMBA (ORIGINAL LYRICS HIP HOP) | anxiety | anxiety | 2.77 | 6.72 | 2.72 | PRIMED JAPANESE TRADITIONAL (ORIGINAL LYRICS BOLERO) | dishonour | dishonour | #N/A | #N/A | #N/A | PRIMED HEAVY METAL (ORIGINAL LYRICS KOTO) | evil, madness, possessed | evil, madness, possessed | #N/A | #N/A | #N/A | PRIMED FADO (ORIGINAL LYRICS POP) | doubts | doubts | #N/A | #N/A | #N/A | PRIMED HIP HOP (ORIGINAL LYRICS SAMBA) | truth, rebellion | truth, rebellion | #N/A | #N/A | #N/A |  |  |
| CUBA |  | I = Incongruent | joy, peace | ecstasy, denial | joy, peace | togetherness, idleness | terror, madness | togetherness | pleasant, joy | elegant, marvelous |  | 6 |  | 1 |  | 5 |  | 5 |  | 2 |  | 6 |  | 2 |  | 7 | 59 | PRIMED POP (ORIGINAL LYRICS OPERA) | love, attraction | love | 8.72 | 6.44 | 7.11 | PRIMED OPERA (ORIGINAL LYRICS FADO) | sentimentalism | sentiment | 5.98 | 4.41 | 5.09 | PRIMED CUBAN SON (ORIGINAL LYRICS HEAVY METAL) | suicide | suicide | 1.25 | 5.73 | 3.58 | PRIMED SAMBA (ORIGINAL LYRICS HIP HOP) | despair | Despair | 2.99 | 4.49 | 4.3 | PRIMED JAPANESE TRADITIONAL (ORIGINAL LYRICS BOLERO) | love, heartache | love | 8.72 | 6.44 | 7.11 | PRIMED HEAVY METAL (ORIGINAL LYRICS KOTO) | evil, diabolic | evil, diabolic | #N/A | #N/A | #N/A | PRIMED FADO (ORIGINAL LYRICS POP) | adiction to drugs | adiction to drugs | #N/A | #N/A | #N/A | PRIMED HIP HOP (ORIGINAL LYRICS SAMBA) | loveless | loveless | #N/A | #N/A | #N/A |  |  |
| CUBA |  | I = Incongruent | joy, delightful, lust | sad, reflexsive | love, lust | stability, security | chaos | nonconformity, molestation, lust | everydayness, joy | control, inteligent |  | 6 |  | 8 |  | 6 |  | 6 |  | 7 |  | 6 |  | 6 |  | 7 | 60 | PRIMED POP (ORIGINAL LYRICS OPERA) | anger | anger | 2.34 | 7.63 | 5.5 | PRIMED OPERA (ORIGINAL LYRICS FADO) | sad | sad | 1.61 | 4.13 | 3.45 | PRIMED CUBAN SON (ORIGINAL LYRICS HEAVY METAL) | repentence | repentence | #N/A | #N/A | #N/A | PRIMED SAMBA (ORIGINAL LYRICS HIP HOP) | sad | sad | 1.61 | 4.13 | 3.45 | PRIMED JAPANESE TRADITIONAL (ORIGINAL LYRICS BOLERO) | sad | sad | 1.61 | 4.13 | 3.45 | PRIMED HEAVY METAL (ORIGINAL LYRICS KOTO) | desperation | despair | 2.99 | 4.49 | 4.3 | PRIMED FADO (ORIGINAL LYRICS POP) | fury | fury | 3.1 | 6.82 | 4.29 | PRIMED HIP HOP (ORIGINAL LYRICS SAMBA) | insecurity | insecure | 2.36 | 5.56 | 2.33 |  |  |
| CUBA |  | I = Incongruent | carnival, movement, lust | feelings, passion | traditional | experimental, | noise | rap, neighbourhood | dream | calm, peace |  | 6 |  | 1 |  | 6 |  | 5 |  | 7 |  | 6 |  | 6 |  | 7 | 61 | PRIMED POP (ORIGINAL LYRICS OPERA) | love, sensual | love | 8.72 | 6.44 | 7.11 | PRIMED OPERA (ORIGINAL LYRICS FADO) | suffering | suffer | 1.72 | 6.13 | 2.54 | PRIMED CUBAN SON (ORIGINAL LYRICS HEAVY METAL) | sad, death | sad | 1.61 | 4.13 | 3.45 | PRIMED SAMBA (ORIGINAL LYRICS HIP HOP) | passion | passion | 8.03 | 7.26 | 6.13 | PRIMED JAPANESE TRADITIONAL (ORIGINAL LYRICS BOLERO) | love | love | 8.72 | 6.44 | 7.11 | PRIMED HEAVY METAL (ORIGINAL LYRICS KOTO) | heaven | heaven | 7.3 | 5.61 | 6.15 | PRIMED FADO (ORIGINAL LYRICS POP) | drugs | drugs | 3.76 | 6 | 4.75 | PRIMED HIP HOP (ORIGINAL LYRICS SAMBA) | love | love | 8.72 | 6.44 | 7.11 |  |  |
| CUBA |  | I = Incongruent | unity, loneliness | passion, reflection | music feel, passion | mistery, nature | bitterness | soul | discussion | relationship |  | 6 |  | 1 |  | 3 |  | 5 |  | 7 |  | 4 |  | 7 |  | 7 | 62 | PRIMED POP (ORIGINAL LYRICS OPERA) | trivial love | trivial love | #N/A | #N/A | #N/A | PRIMED OPERA (ORIGINAL LYRICS FADO) | suffering | suffer | 1.72 | 6.13 | 2.54 | PRIMED CUBAN SON (ORIGINAL LYRICS HEAVY METAL) | sad | sad | 1.61 | 4.13 | 3.45 | PRIMED SAMBA (ORIGINAL LYRICS HIP HOP) | hope, passion | hope | 7.05 | 5.44 | 5.52 | PRIMED JAPANESE TRADITIONAL (ORIGINAL LYRICS BOLERO) | love, heartache | love | 8.72 | 6.44 | 7.11 | PRIMED HEAVY METAL (ORIGINAL LYRICS KOTO) | spiritual | spirit | 7 | 5.56 | 5.82 | PRIMED FADO (ORIGINAL LYRICS POP) | nonconformity | nonconformity | #N/A | #N/A | #N/A | PRIMED HIP HOP (ORIGINAL LYRICS SAMBA) | protection | protection | #N/A | #N/A | #N/A |  |  |
| CUBA |  | I = Incongruent |  | romantic, sad | joy, romantic | I don't like it | nice | ugly | dancing | relax |  |  |  |  |  |  |  |  |  |  |  |  |  |  |  |  | 63 | PRIMED POP (ORIGINAL LYRICS OPERA) | flattering, love | love | 8.72 | 6.44 | 7.11 | PRIMED OPERA (ORIGINAL LYRICS FADO) | suffering | suffer | 1.72 | 6.13 | 2.54 | PRIMED CUBAN SON (ORIGINAL LYRICS HEAVY METAL) | love, sad | love | 8.72 | 6.44 | 7.11 | PRIMED SAMBA (ORIGINAL LYRICS HIP HOP) |  | #N/A | #N/A | #N/A | #N/A | PRIMED JAPANESE TRADITIONAL (ORIGINAL LYRICS BOLERO) | love | love | 8.72 | 6.44 | 7.11 | PRIMED HEAVY METAL (ORIGINAL LYRICS KOTO) |  | #N/A | #N/A | #N/A | #N/A | PRIMED FADO (ORIGINAL LYRICS POP) |  | #N/A | #N/A | #N/A | #N/A | PRIMED HIP HOP (ORIGINAL LYRICS SAMBA) |  | #N/A | #N/A | #N/A | #N/A |  |  |
| CUBA |  | I = Incongruent | joy | love, sad | nostalgia, love | discouragment | bewilderment, deafining | youth, daring | joy, youth | calm, peace |  | 5 |  | 4 |  | 4 |  | 6 |  | 7 |  | 6 |  | 4 |  | 7 | 64 | PRIMED POP (ORIGINAL LYRICS OPERA) | love | love | 8.72 | 6.44 | 7.11 | PRIMED OPERA (ORIGINAL LYRICS FADO) | devotion | devotion | #N/A | #N/A | #N/A | PRIMED CUBAN SON (ORIGINAL LYRICS HEAVY METAL) | violence | violence | #N/A | #N/A | #N/A | PRIMED SAMBA (ORIGINAL LYRICS HIP HOP) | loveless | loveless | #N/A | #N/A | #N/A | PRIMED JAPANESE TRADITIONAL (ORIGINAL LYRICS BOLERO) | attraction | attractive | 7.49 | 6.76 | 5.54 | PRIMED HEAVY METAL (ORIGINAL LYRICS KOTO) | forgiveness | Forgiveness | #N/A | #N/A | #N/A | PRIMED FADO (ORIGINAL LYRICS POP) | vices | vices | #N/A | #N/A | #N/A | PRIMED HIP HOP (ORIGINAL LYRICS SAMBA) | fear of love | fear of love | #N/A | #N/A | #N/A |  |  |
| CUBA |  | I = Incongruent | joy,relax |  | fascinating |  |  | I like it a little bit |  | relaxing |  | 6 |  |  |  |  |  |  |  |  |  | 6 |  |  |  | 7 | 65 | PRIMED POP (ORIGINAL LYRICS OPERA) | | #N/A | #N/A | #N/A | #N/A | PRIMED OPERA (ORIGINAL LYRICS FADO) | patriotic | patriotic | 6.62 | 5.54 | 6.35 | PRIMED CUBAN SON (ORIGINAL LYRICS HEAVY METAL) | violence | violence | #N/A | #N/A | #N/A | PRIMED SAMBA (ORIGINAL LYRICS HIP HOP) | sad | sad | 1.61 | 4.13 | 3.45 | PRIMED JAPANESE TRADITIONAL (ORIGINAL LYRICS BOLERO) |  | #N/A | #N/A | #N/A | #N/A | PRIMED HEAVY METAL (ORIGINAL LYRICS KOTO) |  | #N/A | #N/A | #N/A | #N/A | PRIMED FADO (ORIGINAL LYRICS POP) | victim | victim | 2.18 | 6.06 | 2.69 | PRIMED HIP HOP (ORIGINAL LYRICS SAMBA) | cowardice | cowardice | 2.9 | 4.27 | 4.49 |  |  |
| CUBA |  | I = Incongruent | calm | sad, melancholy | joy, nostalgia | peace | madness, frustration, violence | aggression | love, friendship, joy | virtous, calm |  | 6 |  | 1 |  | 7 |  | 6 |  | 5 |  | 6 |  | 7 |  | 7 | 66 | PRIMED POP (ORIGINAL LYRICS OPERA) | love, attraction | love | 8.72 | 6.44 | 7.11 | PRIMED OPERA (ORIGINAL LYRICS FADO) | patriotic | patriotic | 6.62 | 5.54 | 6.35 | PRIMED CUBAN SON (ORIGINAL LYRICS HEAVY METAL) | betrayal, sad | sad | 1.61 | 4.13 | 3.45 | PRIMED SAMBA (ORIGINAL LYRICS HIP HOP) | passion | passion | 8.03 | 7.26 | 6.13 | PRIMED JAPANESE TRADITIONAL (ORIGINAL LYRICS BOLERO) |  | #N/A | #N/A | #N/A | #N/A | PRIMED HEAVY METAL (ORIGINAL LYRICS KOTO) | hate | Hate | 2.12 | 6.95 | 5.05 | PRIMED FADO (ORIGINAL LYRICS POP) |  | #N/A | #N/A | #N/A | #N/A | PRIMED HIP HOP (ORIGINAL LYRICS SAMBA) | aggressive | aggressive | 5.1 | 5.83 | 5.59 |  |  |
| CUBA |  | I = Incongruent | joy, happy | love, calm | dancing, romantic | madness | sex | madness | dancing | dancing, calm |  |  |  |  |  |  |  |  |  |  |  |  |  |  |  |  | 67 | PRIMED POP (ORIGINAL LYRICS OPERA) | | #N/A | #N/A | #N/A | #N/A | PRIMED OPERA (ORIGINAL LYRICS FADO) | | #N/A | #N/A | #N/A | #N/A | PRIMED CUBAN SON (ORIGINAL LYRICS HEAVY METAL) |  | #N/A | #N/A | #N/A | #N/A | PRIMED SAMBA (ORIGINAL LYRICS HIP HOP) |  | #N/A | #N/A | #N/A | #N/A | PRIMED JAPANESE TRADITIONAL (ORIGINAL LYRICS BOLERO) |  | #N/A | #N/A | #N/A | #N/A | PRIMED HEAVY METAL (ORIGINAL LYRICS KOTO) |  | #N/A | #N/A | #N/A | #N/A | PRIMED FADO (ORIGINAL LYRICS POP) |  | #N/A | #N/A | #N/A | #N/A | PRIMED HIP HOP (ORIGINAL LYRICS SAMBA) |  | #N/A | #N/A | #N/A | #N/A |  |  |
| CUBA |  | I = Incongruent |  |  |  |  |  |  |  |  |  | 6 |  | 4 |  | 6 |  | 6 |  | 4 |  | 6 |  | 5 |  | 7 | 68 | PRIMED POP (ORIGINAL LYRICS OPERA) | trust | trust | 6.68 | 5.3 | 6.61 | PRIMED OPERA (ORIGINAL LYRICS FADO) | patriotic | patriotic | 6.62 | 5.54 | 6.35 | PRIMED CUBAN SON (ORIGINAL LYRICS HEAVY METAL) | sad, regret | sad | 1.61 | 4.13 | 3.45 | PRIMED SAMBA (ORIGINAL LYRICS HIP HOP) | hope | Hope | 7.05 | 5.44 | 5.52 | PRIMED JAPANESE TRADITIONAL (ORIGINAL LYRICS BOLERO) | love | love | 8.72 | 6.44 | 7.11 | PRIMED HEAVY METAL (ORIGINAL LYRICS KOTO) | evil | evil | 3.23 | 6.39 | 5.25 | PRIMED FADO (ORIGINAL LYRICS POP) | sin | sin | 2.8 | 5.78 | 3.62 | PRIMED HIP HOP (ORIGINAL LYRICS SAMBA) | confidence | Confidence | 7.04 | 5.52 | 6.42 |  |  |
| CUBA |  | I = Incongruent | happy, joy | sad | flavourful, joy | calm | chaos, madness | dancing | fun | intelligent |  | 5 |  | 1 |  | 6 |  | 4 |  | 8 |  | 6 |  | 7 |  | 7 | 69 | PRIMED POP (ORIGINAL LYRICS OPERA) | pride | Pride | 7 | 5.83 | 7.06 | PRIMED OPERA (ORIGINAL LYRICS FADO) | suffering | suffer | 1.72 | 6.13 | 2.54 | PRIMED CUBAN SON (ORIGINAL LYRICS HEAVY METAL) | death | Death | 1.61 | 4.59 | 3.47 | PRIMED SAMBA (ORIGINAL LYRICS HIP HOP) | sad | sad | 1.61 | 4.13 | 3.45 | PRIMED JAPANESE TRADITIONAL (ORIGINAL LYRICS BOLERO) | love | love | 8.72 | 6.44 | 7.11 | PRIMED HEAVY METAL (ORIGINAL LYRICS KOTO) | torture, madness | torture, madness | #N/A | #N/A | #N/A | PRIMED FADO (ORIGINAL LYRICS POP) | poetic | Poetic | #N/A | #N/A | #N/A | PRIMED HIP HOP (ORIGINAL LYRICS SAMBA) | insecurity | insecure | 2.36 | 5.56 | 2.33 |  |  |
| CUBA |  | I = Incongruent | dancing | romantic, sad | dancing, joy | peace, calm | madness, noise | reballious | joy, happy | liberation |  | 6 |  | 3 |  | 5 |  | 4 |  | 8 |  | 5 |  | 3 |  | 7 | 70 | PRIMED POP (ORIGINAL LYRICS OPERA) | love, playful | love | 8.72 | 6.44 | 7.11 | PRIMED OPERA (ORIGINAL LYRICS FADO) | sad | sad | 1.61 | 4.13 | 3.45 | PRIMED CUBAN SON (ORIGINAL LYRICS HEAVY METAL) | sad, peace | sad | 1.61 | 4.13 | 3.45 | PRIMED SAMBA (ORIGINAL LYRICS HIP HOP) | passion | passion | 8.03 | 7.26 | 6.13 | PRIMED JAPANESE TRADITIONAL (ORIGINAL LYRICS BOLERO) | sad | sad | 1.61 | 4.13 | 3.45 | PRIMED HEAVY METAL (ORIGINAL LYRICS KOTO) | evil | evil | 3.23 | 6.39 | 5.25 | PRIMED FADO (ORIGINAL LYRICS POP) | freedom | freedom | 7.58 | 5.52 | 6.76 | PRIMED HIP HOP (ORIGINAL LYRICS SAMBA) | regret | Regret | 2.26 | 5.67 | 3.23 |  |  |
| CUBA |  | I = Incongruent | dancing, joy | nostalgia | romance, flirting | spiritual | madness | passionate | dancing, happy | elegant |  | 5 |  | 1 |  | 5 |  | 6 |  | 8 |  | 6 |  | 4 |  | 6 | 71 | PRIMED POP (ORIGINAL LYRICS OPERA) | sensual | Sensual | #N/A | #N/A | #N/A | PRIMED OPERA (ORIGINAL LYRICS FADO) | patriotic | patriotic | 6.62 | 5.54 | 6.35 | PRIMED CUBAN SON (ORIGINAL LYRICS HEAVY METAL) | sad | sad | 1.61 | 4.13 | 3.45 | PRIMED SAMBA (ORIGINAL LYRICS HIP HOP) | sad | sad | 1.61 | 4.13 | 3.45 | PRIMED JAPANESE TRADITIONAL (ORIGINAL LYRICS BOLERO) | sad | sad | 1.61 | 4.13 | 3.45 | PRIMED HEAVY METAL (ORIGINAL LYRICS KOTO) | madness | madness | 4.37 | 5.8 | 4.53 | PRIMED FADO (ORIGINAL LYRICS POP) | freedom | freedom | 7.58 | 5.52 | 6.76 | PRIMED HIP HOP (ORIGINAL LYRICS SAMBA) | sad | sad | 1.61 | 4.13 | 3.45 |  |  |
| CUBA |  | UP = Unprimed | party, trivial | tranquility, sad | flavourful, love | absent | violence | authentic | beauty | calm |  | 6 |  | 1 |  | 6 |  | 5 |  | 2 |  | 6 |  | 6 |  | 7 | 72 | UNPRIMED (ORIGINAL LYRICS OPERA) | longing | Longing | #N/A | #N/A | #N/A | UNPRIMED (ORIGINAL LYRICS FADO) | sad | sad | 1.61 | 4.13 | 3.45 | UNPRIMED (ORIGINAL LYRICS HEAY METAL) | death | Death | 1.61 | 4.59 | 3.47 | UNPRIMED (ORIGINAL LYRICS HIP HOP) | sorrow | sorrow | 2.32 | 4.48 | 3.67 | UNPRIMED (ORIINAL LYRICS BOLERO) | love | love | 8.72 | 6.44 | 7.11 | UNPRIMED (ORIGINAL LYRICS KOTO) | shame | shame | 2.13 | 6.33 | 2.97 | UNPRIMED (ORIGINAL LYRICS POP) | impurity | impurity | #N/A | #N/A | #N/A | UNPRIMED (ORIGINAL LYRICS SAMBA) | love | love | 8.72 | 6.44 | 7.11 |  |  |
| CUBA |  | UP = Unprimed | party, nostalgia | emotive | peace, memories | intrigue, mystery | out of control state of mind | courage | continous absorption | relax, imagination |  | 6 |  | 2 |  | 6 |  | 7 |  | 5 |  | 6 |  | 7 |  | 7 | 73 | UNPRIMED (ORIGINAL LYRICS OPERA) | inspirational | inspirational | 7.57 | 6.18 | 6.3 | UNPRIMED (ORIGINAL LYRICS FADO) | representation | #N/A | #N/A | #N/A | #N/A | UNPRIMED (ORIGINAL LYRICS HEAY METAL) | rebelious | rebellious | 4.86 | 5.82 | 6.28 | UNPRIMED (ORIGINAL LYRICS HIP HOP) |  | #N/A | #N/A | #N/A | #N/A | UNPRIMED (ORIINAL LYRICS BOLERO) | sorrow | sorrow | 2.32 | 4.48 | 3.67 | UNPRIMED (ORIGINAL LYRICS KOTO) | memories | memories | 7.48 | 6.1 | 5.88 | UNPRIMED (ORIGINAL LYRICS POP) | misunderstood | misunderstood | #N/A | #N/A | #N/A | UNPRIMED (ORIGINAL LYRICS SAMBA) | sincerity | sincerity | #N/A | #N/A | #N/A |  |  |
| CUBA |  | UP = Unprimed | joy, peace | silence, tranquility | good, love | energy, air | ecstasy, fast | controversial, violence | conversation, joy | solution, attention |  |  |  |  |  |  |  |  |  |  |  |  |  |  |  |  | 74 | UNPRIMED (ORIGINAL LYRICS OPERA) | nice | nice | 6.55 | 4.38 | 5.58 | UNPRIMED (ORIGINAL LYRICS FADO) | attetion | #N/A | #N/A | #N/A | #N/A | UNPRIMED (ORIGINAL LYRICS HEAY METAL) | death | Death | 1.61 | 4.59 | 3.47 | UNPRIMED (ORIGINAL LYRICS HIP HOP) | love | love | 8.72 | 6.44 | 7.11 | UNPRIMED (ORIINAL LYRICS BOLERO) | attraction | attractive | 7.49 | 6.76 | 5.54 | UNPRIMED (ORIGINAL LYRICS KOTO) | kindness | kindness | 7.82 | 4.3 | 5.67 | UNPRIMED (ORIGINAL LYRICS POP) | dark | Dark | 4.71 | 4.28 | 4.84 | UNPRIMED (ORIGINAL LYRICS SAMBA) | daring | daring | #N/A | #N/A | #N/A |  |  |
| CUBA |  | UP = Unprimed | african descent people | sky, tranquility | music, dancing | I don't understand it | black, guitars | streets, Hip Hop clothes | relax, sharing | relax, theatre |  | 6 |  | 3 |  | 6 |  | 4 |  | 3 |  | 6 |  | 1 |  | 6 | 75 | UNPRIMED (ORIGINAL LYRICS OPERA) | women | women | #N/A | #N/A | #N/A | UNPRIMED (ORIGINAL LYRICS FADO) | heroic | #N/A | #N/A | #N/A | #N/A | UNPRIMED (ORIGINAL LYRICS HEAY METAL) | death | Death | 1.61 | 4.59 | 3.47 | UNPRIMED (ORIGINAL LYRICS HIP HOP) | sky | sky | 7.37 | 4.27 | 5.16 | UNPRIMED (ORIINAL LYRICS BOLERO) | flowers | flowers | #N/A | #N/A | #N/A | UNPRIMED (ORIGINAL LYRICS KOTO) | jesus | jesus | #N/A | #N/A | #N/A | UNPRIMED (ORIGINAL LYRICS POP) | land | land | 5.66 | 4.16 | 5.53 | UNPRIMED (ORIGINAL LYRICS SAMBA) | love | love | 8.72 | 6.44 | 7.11 |  |  |
| CUBA |  | UP = Unprimed | party, joy, sensual | sensual, confession, love | flavourful, confession | punishment, dancing | madness, anxiety | flow, dirty | relax, fresh | on time, majestic |  | 6 |  | 5 |  | 6 |  | 6 |  | 5 |  | 6 |  | 6 |  | 7 | 76 | UNPRIMED (ORIGINAL LYRICS OPERA) | modesty | modesty | 6.03 | 4.28 | 5.8 | UNPRIMED (ORIGINAL LYRICS FADO) | patriotic | patriotic | 6.62 | 5.54 | 6.35 | UNPRIMED (ORIGINAL LYRICS HEAY METAL) | resurgence | resurgence | #N/A | #N/A | #N/A | UNPRIMED (ORIGINAL LYRICS HIP HOP) | desperartion | despair | 2.99 | 4.49 | 4.3 | UNPRIMED (ORIINAL LYRICS BOLERO) | attraction | attractive | 7.49 | 6.76 | 5.54 | UNPRIMED (ORIGINAL LYRICS KOTO) |  | #N/A | #N/A | #N/A | #N/A | UNPRIMED (ORIGINAL LYRICS POP) |  | #N/A | #N/A | #N/A | #N/A | UNPRIMED (ORIGINAL LYRICS SAMBA) |  | #N/A | #N/A | #N/A | #N/A |  |  |
| CUBA |  | UP = Unprimed | party | sentimental | joy, nostalgia | curiosity | hate | aggression | joy, memories | relax, pleasing |  | 6 |  | 1 |  | 6 |  | 1 |  | 8 |  | 6 |  | 6 |  | 7 | 77 | UNPRIMED (ORIGINAL LYRICS OPERA) | seduction | seduction | 7.53 | 7.43 | 5.63 | UNPRIMED (ORIGINAL LYRICS FADO) | sad | sad | 1.61 | 4.13 | 3.45 | UNPRIMED (ORIGINAL LYRICS HEAY METAL) | mystery | mystery | #N/A | #N/A | #N/A | UNPRIMED (ORIGINAL LYRICS HIP HOP) | incognito | incognito | #N/A | #N/A | #N/A | UNPRIMED (ORIINAL LYRICS BOLERO) | nostalgia | nostalgia | #N/A | #N/A | #N/A | UNPRIMED (ORIGINAL LYRICS KOTO) | faith | faith | 6.57 | 5.73 | 5.6 | UNPRIMED (ORIGINAL LYRICS POP) | protest | protest | #N/A | #N/A | #N/A | UNPRIMED (ORIGINAL LYRICS SAMBA) | loveless | loveless | #N/A | #N/A | #N/A |  |  |
| CUBA |  | UP = Unprimed | romatic | love | resentment | incidental | I like | abrupt | movement | relaxing |  | 5 |  | 6 |  | 6 |  | 6 |  | 8 |  | 7 |  | 7 |  | 5 | 78 | UNPRIMED (ORIGINAL LYRICS OPERA) | vanity | Vanity | 4.3 | 4.98 | 4.8 | UNPRIMED (ORIGINAL LYRICS FADO) | lust | lust | 7.12 | 6.88 | 5.49 | UNPRIMED (ORIGINAL LYRICS HEAY METAL) | glory | glory | 7.55 | 6.02 | 6.85 | UNPRIMED (ORIGINAL LYRICS HIP HOP) | death | Death | 1.61 | 4.59 | 3.47 | UNPRIMED (ORIINAL LYRICS BOLERO) | love | love | 8.72 | 6.44 | 7.11 | UNPRIMED (ORIGINAL LYRICS KOTO) | regret | Regret | 2.26 | 5.67 | 3.23 | UNPRIMED (ORIGINAL LYRICS POP) | protest | protest | #N/A | #N/A | #N/A | UNPRIMED (ORIGINAL LYRICS SAMBA) | regret | Regret | 2.26 | 5.67 | 3.23 |  |  |
| CUBA |  | UP = Unprimed | beauty | calm, melancholy | dacing | sad, melancholy | disturbance, annoying |  |  |  |  | 6 |  | 1 |  | 2 |  | 2 |  | 7 |  | 2 |  |  |  | 7 | 79 | UNPRIMED (ORIGINAL LYRICS OPERA) | | #N/A | #N/A | #N/A | #N/A | UNPRIMED (ORIGINAL LYRICS FADO) | passion | passion | 8.03 | 7.26 | 6.13 | UNPRIMED (ORIGINAL LYRICS HEAY METAL) | stink | stink | 3 | 4.26 | 4.16 | UNPRIMED (ORIGINAL LYRICS HIP HOP) | desperation | despair | 2.99 | 4.49 | 4.3 | UNPRIMED (ORIINAL LYRICS BOLERO) | love | love | 8.72 | 6.44 | 7.11 | UNPRIMED (ORIGINAL LYRICS KOTO) |  | #N/A | #N/A | #N/A | #N/A | UNPRIMED (ORIGINAL LYRICS POP) |  | #N/A | #N/A | #N/A | #N/A | UNPRIMED (ORIGINAL LYRICS SAMBA) |  | #N/A | #N/A | #N/A | #N/A |  |  |
| CUBA |  | UP = Unprimed | party | relax | joy, dancing | interesting | stress | rudeness | flavourful | sweet to the ear |  | 5 | I avoid this kind of music | 1 |  | 6 |  | 6 |  | 4 |  | 6 |  | 4 | I like to this music a lot | 7 | 80 | UNPRIMED (ORIGINAL LYRICS OPERA) | | #N/A | #N/A | #N/A | #N/A | UNPRIMED (ORIGINAL LYRICS FADO) | nationality | #N/A | #N/A | #N/A | #N/A | UNPRIMED (ORIGINAL LYRICS HEAY METAL) | death | Death | 1.61 | 4.59 | 3.47 | UNPRIMED (ORIGINAL LYRICS HIP HOP) | shame | shame | 2.13 | 6.33 | 2.97 | UNPRIMED (ORIINAL LYRICS BOLERO) | love | love | 8.72 | 6.44 | 7.11 | UNPRIMED (ORIGINAL LYRICS KOTO) | praise | praise | 7.24 | 6.28 | 6.59 | UNPRIMED (ORIGINAL LYRICS POP) | suburbs | suburbs | #N/A | #N/A | #N/A | UNPRIMED (ORIGINAL LYRICS SAMBA) | doubt | doubt | 3.43 | 4.5 | 3.64 |  |  |
| CUBA |  | UP = Unprimed | beach | tranquility | passion | calm | headache | violence | cool | emotive |  | 5 |  | 3 |  | 6 |  | 3 |  | 3 |  | 6 |  | 3 |  | 4 | 81 | UNPRIMED (ORIGINAL LYRICS OPERA) | | #N/A | #N/A | #N/A | #N/A | UNPRIMED (ORIGINAL LYRICS FADO) | | #N/A | #N/A | #N/A | #N/A | UNPRIMED (ORIGINAL LYRICS HEAY METAL) |  | #N/A | #N/A | #N/A | #N/A | UNPRIMED (ORIGINAL LYRICS HIP HOP) |  | #N/A | #N/A | #N/A | #N/A | UNPRIMED (ORIINAL LYRICS BOLERO) |  | #N/A | #N/A | #N/A | #N/A | UNPRIMED (ORIGINAL LYRICS KOTO) |  | #N/A | #N/A | #N/A | #N/A | UNPRIMED (ORIGINAL LYRICS POP) |  | #N/A | #N/A | #N/A | #N/A | UNPRIMED (ORIGINAL LYRICS SAMBA) |  | #N/A | #N/A | #N/A | #N/A |  |  |
| CUBA |  | UP = Unprimed | melacholy, magic | marvelous, love | love |  | madness, alarming | passionate | joy | uncovering |  | 6 |  | 1 |  | 2 |  | 5 |  | 1 |  | 6 |  | 6 |  | 7 | 82 | UNPRIMED (ORIGINAL LYRICS OPERA) | pleasant | pleasant | #N/A | #N/A | #N/A | UNPRIMED (ORIGINAL LYRICS FADO) | passion | passion | 8.03 | 7.26 | 6.13 | UNPRIMED (ORIGINAL LYRICS HEAY METAL) |  | #N/A | #N/A | #N/A | #N/A | UNPRIMED (ORIGINAL LYRICS HIP HOP) | desperation | despair | 2.99 | 4.49 | 4.3 | UNPRIMED (ORIINAL LYRICS BOLERO) | sad | sad | 1.61 | 4.13 | 3.45 | UNPRIMED (ORIGINAL LYRICS KOTO) |  | #N/A | #N/A | #N/A | #N/A | UNPRIMED (ORIGINAL LYRICS POP) | anger | anger | 2.34 | 7.63 | 5.5 | UNPRIMED (ORIGINAL LYRICS SAMBA) | doubt | doubt | 3.43 | 4.5 | 3.64 |  |  |
| CUBA |  | UP = Unprimed |  | romantic | dancing | ritual, meditation | madness | streets | sensual, party | fantasy |  | 5 |  | 1 |  | 2 |  | 5 |  | 8 |  | 7 |  | 1 |  | 6 | 83 | UNPRIMED (ORIGINAL LYRICS OPERA) | | #N/A | #N/A | #N/A | #N/A | UNPRIMED (ORIGINAL LYRICS FADO) | patriotic | patriotic | 6.62 | 5.54 | 6.35 | UNPRIMED (ORIGINAL LYRICS HEAY METAL) |  | #N/A | #N/A | #N/A | #N/A | UNPRIMED (ORIGINAL LYRICS HIP HOP) |  | #N/A | #N/A | #N/A | #N/A | UNPRIMED (ORIINAL LYRICS BOLERO) |  | #N/A | #N/A | #N/A | #N/A | UNPRIMED (ORIGINAL LYRICS KOTO) |  | #N/A | #N/A | #N/A | #N/A | UNPRIMED (ORIGINAL LYRICS POP) |  | #N/A | #N/A | #N/A | #N/A | UNPRIMED (ORIGINAL LYRICS SAMBA) |  | #N/A | #N/A | #N/A | #N/A |  |  |
| CUBA |  | UP = Unprimed | joy, tranquility | peace, sad | movement, meditation | instability | torment, running | protest, anger | sensual, stimulating | relaxing, torment |  | 6 |  | 6 |  | 6 |  | 6 |  | 8 |  | 6 |  | 6 |  | 7 | 84 | UNPRIMED (ORIGINAL LYRICS OPERA) | sensual | Sensual | #N/A | #N/A | #N/A | UNPRIMED (ORIGINAL LYRICS FADO) | suffering, longing | #N/A | #N/A | #N/A | #N/A | UNPRIMED (ORIGINAL LYRICS HEAY METAL) |  | #N/A | #N/A | #N/A | #N/A | UNPRIMED (ORIGINAL LYRICS HIP HOP) |  | #N/A | #N/A | #N/A | #N/A | UNPRIMED (ORIINAL LYRICS BOLERO) |  | #N/A | #N/A | #N/A | #N/A | UNPRIMED (ORIGINAL LYRICS KOTO) |  | #N/A | #N/A | #N/A | #N/A | UNPRIMED (ORIGINAL LYRICS POP) |  | #N/A | #N/A | #N/A | #N/A | UNPRIMED (ORIGINAL LYRICS SAMBA) |  | #N/A | #N/A | #N/A | #N/A |  |  |
| CUBA |  | UP = Unprimed | joy | longing | nothing | \* | strong | \* | superficial | \* |  | 3 |  | 5 |  | 4 |  | 2 |  | 8 |  | 6 |  | 7 |  | 3 | 85 | UNPRIMED (ORIGINAL LYRICS OPERA) | pity | pity | 3.37 | 3.72 | 4.12 | UNPRIMED (ORIGINAL LYRICS FADO) | annoyance | annoyance | 2.97 | 5.18 | 4.21 | UNPRIMED (ORIGINAL LYRICS HEAY METAL) | disconcern | disconcern | #N/A | #N/A | #N/A | UNPRIMED (ORIGINAL LYRICS HIP HOP) |  | #N/A | #N/A | #N/A | #N/A | UNPRIMED (ORIINAL LYRICS BOLERO) | nostalgia | nostalgia | #N/A | #N/A | #N/A | UNPRIMED (ORIGINAL LYRICS KOTO) | nothing | nothing | #N/A | #N/A | #N/A | UNPRIMED (ORIGINAL LYRICS POP) | disgust | disgusting | 2.96 | 5.18 | 3.64 | UNPRIMED (ORIGINAL LYRICS SAMBA) | compassion | compassion | 7.73 | 5.47 | 5.65 |  |  |
| CUBA |  | UP = Unprimed | joy, nostalgia | grief, love | dancing, drunkness | yoga, relax, dancing | madness | mimic | singing | relax, tranquility, energy |  | 5 |  | 1 |  | 4 |  | 1 |  | 1 |  | 4 |  | 2 |  | 3 | 86 | UNPRIMED (ORIGINAL LYRICS OPERA) | beauty | beauty | 7.82 | 4.95 | 5.53 | UNPRIMED (ORIGINAL LYRICS FADO) | politics | #N/A | #N/A | #N/A | #N/A | UNPRIMED (ORIGINAL LYRICS HEAY METAL) | death | Death | 1.61 | 4.59 | 3.47 | UNPRIMED (ORIGINAL LYRICS HIP HOP) | desperation | despair | 2.99 | 4.49 | 4.3 | UNPRIMED (ORIINAL LYRICS BOLERO) | attraction | attractive | 7.49 | 6.76 | 5.54 | UNPRIMED (ORIGINAL LYRICS KOTO) | religion | 0 | #N/A | #N/A | #N/A | UNPRIMED (ORIGINAL LYRICS POP) |  | #N/A | #N/A | #N/A | #N/A | UNPRIMED (ORIGINAL LYRICS SAMBA) | love | love | 8.72 | 6.44 | 7.11 |  |  |
| CUBA |  | UP = Unprimed | nostalgia, love | loneliness, weariness | joy, sad | prayer, tranquility | torment, joy | happy | romance, joy | relax, mystery |  |  |  |  |  |  |  |  |  |  |  |  |  |  |  |  | 87 | UNPRIMED (ORIGINAL LYRICS OPERA) | playful | playful | 6.54 | 5.63 | 5.78 | UNPRIMED (ORIGINAL LYRICS FADO) | yearning | yearning | #N/A | #N/A | #N/A | UNPRIMED (ORIGINAL LYRICS HEAY METAL) |  | #N/A | #N/A | #N/A | #N/A | UNPRIMED (ORIGINAL LYRICS HIP HOP) | sweet | sweet | 7.64 | 5.96 | 5.36 | UNPRIMED (ORIINAL LYRICS BOLERO) | love | love | 8.72 | 6.44 | 7.11 | UNPRIMED (ORIGINAL LYRICS KOTO) | faith | faith | 6.57 | 5.73 | 5.6 | UNPRIMED (ORIGINAL LYRICS POP) | contempt | contempt | 3.85 | 5.28 | 5.13 | UNPRIMED (ORIGINAL LYRICS SAMBA) | delirious | delirious | #N/A | #N/A | #N/A |  |  |
| CUBA |  | UP = Unprimed | fraternity, swing | sentimental, emotive | freedom, love | primitive, exotic | rebellious, madness | street, fight | nice, party | sublime, epic |  | 7 |  | 4 |  | 7 |  | 2 |  | 3 |  | 6 |  | 6 |  | 7 | 89 | UNPRIMED (ORIGINAL LYRICS OPERA) | meaningful | emotional | 4.36 | 5.75 | 4.29 | UNPRIMED (ORIGINAL LYRICS FADO) | sentimental | #N/A | #N/A | #N/A | #N/A | UNPRIMED (ORIGINAL LYRICS HEAY METAL) | death | Death | 1.61 | 4.59 | 3.47 | UNPRIMED (ORIGINAL LYRICS HIP HOP) | madness | madness | 4.37 | 5.8 | 4.53 | UNPRIMED (ORIINAL LYRICS BOLERO) | spite | spite | #N/A | #N/A | #N/A | UNPRIMED (ORIGINAL LYRICS KOTO) | sky | sky | 7.37 | 4.27 | 5.16 | UNPRIMED (ORIGINAL LYRICS POP) | confusion | confusion | 3.46 | 6.07 | 3.04 | UNPRIMED (ORIGINAL LYRICS SAMBA) | beautiful | beautiful | 7.6 | 6.17 | 6.29 |  |  |
| CUBA |  | UP = Unprimed | joy, peaceful | sadness, pain | seduction, feeling, love | zen, dreaming | anger, power | protest | joy, dance, feeling | waterfall, magnificant |  | 6 |  | 4 |  | 7 |  | 6 |  | 5 |  | 6 |  | 6 |  | 7 | 90 | UNPRIMED (ORIGINAL LYRICS OPERA) | seduction | seduction | 7.53 | 7.43 | 5.63 | UNPRIMED (ORIGINAL LYRICS FADO) | pain | pain | 2.13 | 6.5 | 3.71 | UNPRIMED (ORIGINAL LYRICS HEAY METAL) | darkness | darkness | #N/A | #N/A | #N/A | UNPRIMED (ORIGINAL LYRICS HIP HOP) | I don't know | I don't know | #N/A | #N/A | #N/A | UNPRIMED (ORIINAL LYRICS BOLERO) | heartbroken | heartbreak | 1.93 | 5.8 | 3.11 | UNPRIMED (ORIGINAL LYRICS KOTO) | religion | 0 | #N/A | #N/A | #N/A | UNPRIMED (ORIGINAL LYRICS POP) | protest | protest | #N/A | #N/A | #N/A | UNPRIMED (ORIGINAL LYRICS SAMBA) | fear | fear | 2.76 | 6.96 | 3.22 |  |  |
| CUBA |  | UP = Unprimed | dancing, joy | wellbeing | family, tranquility | serene, family | torment, anxiety | dark | happy, party | culture, memories of my school studies |  | 7 |  | 2 |  | 6 |  | 6 |  | 4 |  | 6 |  | 1 | It's my identity | 7 | 91 | UNPRIMED (ORIGINAL LYRICS OPERA) | sensual | Sensual | #N/A | #N/A | #N/A | UNPRIMED (ORIGINAL LYRICS FADO) | sad | sad | 1.61 | 4.13 | 3.45 | UNPRIMED (ORIGINAL LYRICS HEAY METAL) | desperation | despair | 2.99 | 4.49 | 4.3 | UNPRIMED (ORIGINAL LYRICS HIP HOP) | persuit | persuit | #N/A | #N/A | #N/A | UNPRIMED (ORIINAL LYRICS BOLERO) | attraction | attractive | 7.49 | 6.76 | 5.54 | UNPRIMED (ORIGINAL LYRICS KOTO) | religion | 0 | #N/A | #N/A | #N/A | UNPRIMED (ORIGINAL LYRICS POP) | infant | infant | 6.95 | 5.05 | 5.67 | UNPRIMED (ORIGINAL LYRICS SAMBA) | fear | fear | 2.76 | 6.96 | 3.22 |  |  |
| CUBA |  | UP = Unprimed | joy | tranquility | dancing | rare | noise | dancing | dancing | tranquility |  | 6 |  | 1 |  | 5 |  | 5 |  | 1 |  | 5 |  | 4 |  | 7 | 92 | UNPRIMED (ORIGINAL LYRICS OPERA) | nothing | nothing | #N/A | #N/A | #N/A | UNPRIMED (ORIGINAL LYRICS FADO) | nothing | nothing | #N/A | #N/A | #N/A | UNPRIMED (ORIGINAL LYRICS HEAY METAL) | nothing | nothing | #N/A | #N/A | #N/A | UNPRIMED (ORIGINAL LYRICS HIP HOP) | nothing | nothing | #N/A | #N/A | #N/A | UNPRIMED (ORIINAL LYRICS BOLERO) | love | love | 8.72 | 6.44 | 7.11 | UNPRIMED (ORIGINAL LYRICS KOTO) | nothing | nothing | #N/A | #N/A | #N/A | UNPRIMED (ORIGINAL LYRICS POP) | nothing | nothing | #N/A | #N/A | #N/A | UNPRIMED (ORIGINAL LYRICS SAMBA) | nothing | nothing | #N/A | #N/A | #N/A |  |  |
| CUBA |  | UP = Unprimed | joy | sentimental | wailing |  | rebellion | restless | anxiety | harmony |  |  |  |  |  |  |  |  |  |  |  |  |  |  |  |  | 93 | UNPRIMED (ORIGINAL LYRICS OPERA) | | #N/A | #N/A | #N/A | #N/A | UNPRIMED (ORIGINAL LYRICS FADO) | yearning | yearning | #N/A | #N/A | #N/A | UNPRIMED (ORIGINAL LYRICS HEAY METAL) | resegnation | resegnation | #N/A | #N/A | #N/A | UNPRIMED (ORIGINAL LYRICS HIP HOP) |  | #N/A | #N/A | #N/A | #N/A | UNPRIMED (ORIINAL LYRICS BOLERO) | attraction | attractive | 7.49 | 6.76 | 5.54 | UNPRIMED (ORIGINAL LYRICS KOTO) |  | #N/A | #N/A | #N/A | #N/A | UNPRIMED (ORIGINAL LYRICS POP) |  | #N/A | #N/A | #N/A | #N/A | UNPRIMED (ORIGINAL LYRICS SAMBA) |  | #N/A | #N/A | #N/A | #N/A |  |  |
| CUBA |  | UP = Unprimed | joy, love | sad | memories | religeous | dancing, frenzy | rebellious | unity, dancing | relax, turbulance |  | 6 |  | 6 |  | 6 |  | 6 |  | 8 |  | 6 |  | 2 |  | 7 | 94 | UNPRIMED (ORIGINAL LYRICS OPERA) | erotic | erotic | 7.43 | 7.24 | 6.39 | UNPRIMED (ORIGINAL LYRICS FADO) | patriotic | patriotic | 6.62 | 5.54 | 6.35 | UNPRIMED (ORIGINAL LYRICS HEAY METAL) | repantance | repantance | #N/A | #N/A | #N/A | UNPRIMED (ORIGINAL LYRICS HIP HOP) | disappoinment | disappointment | 2.37 | 4.6 | 3.2 | UNPRIMED (ORIINAL LYRICS BOLERO) | disapointment | disappointment | 2.37 | 4.6 | 3.2 | UNPRIMED (ORIGINAL LYRICS KOTO) | purification | purification | #N/A | #N/A | #N/A | UNPRIMED (ORIGINAL LYRICS POP) |  | #N/A | #N/A | #N/A | #N/A | UNPRIMED (ORIGINAL LYRICS SAMBA) | fear | fear | 2.76 | 6.96 | 3.22 |  |  |
| CUBA |  | UP = Unprimed | joy | sad | flavourful | tranquility | hate, violence | serene | madness | nice |  | 6 |  | 5 |  | 6 |  | 6 |  | 8 |  | 7 |  | 1 |  | 1 | 95 | UNPRIMED (ORIGINAL LYRICS OPERA) | attraction | attractive | 7.49 | 6.76 | 5.54 | UNPRIMED (ORIGINAL LYRICS FADO) | sad | sad | 1.61 | 4.13 | 3.45 | UNPRIMED (ORIGINAL LYRICS HEAY METAL) | strength | strength | 7.41 | 5.76 | 7.21 | UNPRIMED (ORIGINAL LYRICS HIP HOP) | desperation | despair | 2.99 | 4.49 | 4.3 | UNPRIMED (ORIINAL LYRICS BOLERO) | attraction | attractive | 7.49 | 6.76 | 5.54 | UNPRIMED (ORIGINAL LYRICS KOTO) | suffering | suffer | 1.72 | 6.13 | 2.54 | UNPRIMED (ORIGINAL LYRICS POP) | neutral | Neutral | #N/A | #N/A | #N/A | UNPRIMED (ORIGINAL LYRICS SAMBA) | loveless | loveless | #N/A | #N/A | #N/A |  |  |
| CUBA |  | UP = Unprimed | joy, tenderness | tranquility | love | unfamiliar | rebellious, aggressive | noise | sensual | dark |  | 4 |  | 1 |  | 7 |  | 3 |  | 8 |  | 6 |  | 7 |  | 2 | 96 | UNPRIMED (ORIGINAL LYRICS OPERA) | fear | fear | 2.76 | 6.96 | 3.22 | UNPRIMED (ORIGINAL LYRICS FADO) | nationalism | #N/A | #N/A | #N/A | #N/A | UNPRIMED (ORIGINAL LYRICS HEAY METAL) | tragedy | tragedy | 1.78 | 6.24 | 3.5 | UNPRIMED (ORIGINAL LYRICS HIP HOP) | madness | madness | 4.37 | 5.8 | 4.53 | UNPRIMED (ORIINAL LYRICS BOLERO) | spite | spite | #N/A | #N/A | #N/A | UNPRIMED (ORIGINAL LYRICS KOTO) | prejudice | prejudice | 2.98 | 5.17 | 4.85 | UNPRIMED (ORIGINAL LYRICS POP) | addiction | addiction | #N/A | #N/A | #N/A | UNPRIMED (ORIGINAL LYRICS SAMBA) | fear | fear | 2.76 | 6.96 | 3.22 |  |  |
| CUBA |  | UP = Unprimed | relax | pleasing, sentimental | roots | spiritual | exploiting | rudeness | movement | soul |  | 1 |  | 1 |  | 6 |  | 1 |  | 1 |  | 6 |  | 1 |  | 1 | 97 | UNPRIMED (ORIGINAL LYRICS OPERA) | flirting | flirting | 7.04 | 7.03 | 5.48 | UNPRIMED (ORIGINAL LYRICS FADO) | poetry | poetry | 5.86 | 4 | 5.31 | UNPRIMED (ORIGINAL LYRICS HEAY METAL) | depression | depression | 1.85 | 4.54 | 2.91 | UNPRIMED (ORIGINAL LYRICS HIP HOP) | desperation | despair | 2.99 | 4.49 | 4.3 | UNPRIMED (ORIINAL LYRICS BOLERO) | sorrow | sorrow | 2.32 | 4.48 | 3.67 | UNPRIMED (ORIGINAL LYRICS KOTO) | talking | talking | #N/A | #N/A | #N/A | UNPRIMED (ORIGINAL LYRICS POP) | emptiness | emptiness | #N/A | #N/A | #N/A | UNPRIMED (ORIGINAL LYRICS SAMBA) | truth | truth | 7.8 | 5 | 6.47 |  |  |
| CUBA |  | UP = Unprimed | party, joy | romantic | persistent | meditation | aggressive | movement | sensual | attractive |  | 1 |  | 3 |  | 2 |  | 2 |  | 7 |  | 5 |  | 2 |  | 1 | 98 | UNPRIMED (ORIGINAL LYRICS OPERA) | attraction | attractive | 7.49 | 6.76 | 5.54 | UNPRIMED (ORIGINAL LYRICS FADO) | passion | passion | 8.03 | 7.26 | 6.13 | UNPRIMED (ORIGINAL LYRICS HEAY METAL) | tragedy | tragedy | 1.78 | 6.24 | 3.5 | UNPRIMED (ORIGINAL LYRICS HIP HOP) | pain | pain | 2.13 | 6.5 | 3.71 | UNPRIMED (ORIINAL LYRICS BOLERO) | love | love | 8.72 | 6.44 | 7.11 | UNPRIMED (ORIGINAL LYRICS KOTO) | religion | 0 | #N/A | #N/A | #N/A | UNPRIMED (ORIGINAL LYRICS POP) | addiction | addiction | #N/A | #N/A | #N/A | UNPRIMED (ORIGINAL LYRICS SAMBA) | indecisive | indecisive | #N/A | #N/A | #N/A |  |  |
| CUBA |  | UP = Unprimed | joy | nostalgia | feeling | spiritual | hopeless | nonconformity | happy | spiritual |  | 6 |  | 1 |  | 2 |  | 2 |  | 8 |  | 2 |  | 2 |  | 6 | 99 | UNPRIMED (ORIGINAL LYRICS OPERA) | loneliness | loneliness | 1.61 | 4.56 | 2.51 | UNPRIMED (ORIGINAL LYRICS FADO) | madness | madness | 4.37 | 5.8 | 4.53 | UNPRIMED (ORIGINAL LYRICS HEAY METAL) | death | Death | 1.61 | 4.59 | 3.47 | UNPRIMED (ORIGINAL LYRICS HIP HOP) | hopeless | hopeless | 2.27 | 4.28 | 2.96 | UNPRIMED (ORIINAL LYRICS BOLERO) | love | love | 8.72 | 6.44 | 7.11 | UNPRIMED (ORIGINAL LYRICS KOTO) | soul purifying | soul purifying | #N/A | #N/A | #N/A | UNPRIMED (ORIGINAL LYRICS POP) | hopeless | hopeless | 2.27 | 4.28 | 2.96 | UNPRIMED (ORIGINAL LYRICS SAMBA) | fear | fear | 2.76 | 6.96 | 3.22 |  |  |
| CUBA |  | UP = Unprimed | party, joy, sensua, energy | melancholy | sensual, traditional | mystic, unique | aggressive | streets | pasive, movement | deep, elegant |  | 7 |  | 2 |  | 6 |  | 3 |  | 3 |  | 6 |  | 6 |  | 7 | 100 | UNPRIMED (ORIGINAL LYRICS OPERA) | vanity | Vanity | 4.3 | 4.98 | 4.8 | UNPRIMED (ORIGINAL LYRICS FADO) | love | love | 8.72 | 6.44 | 7.11 | UNPRIMED (ORIGINAL LYRICS HEAY METAL) |  | #N/A | #N/A | #N/A | #N/A | UNPRIMED (ORIGINAL LYRICS HIP HOP) | pain | pain | 2.13 | 6.5 | 3.71 | UNPRIMED (ORIINAL LYRICS BOLERO) | hopeless | hopeless | 2.27 | 4.28 | 2.96 | UNPRIMED (ORIGINAL LYRICS KOTO) | christian | christian | #N/A | #N/A | #N/A | UNPRIMED (ORIGINAL LYRICS POP) | madness | madness | 4.37 | 5.8 | 4.53 | UNPRIMED (ORIGINAL LYRICS SAMBA) | cowardice | cowardice | 2.9 | 4.27 | 4.49 |  |  |
| CUBA |  | UP = Unprimed | joy, tranquility | yearning | traditional | peace, meditation | liberation | expressive | joy | relax |  | 5 |  | 8 |  | 4 |  | 6 |  | 8 |  | 5 |  | 4 |  | 7 | 101 | UNPRIMED (ORIGINAL LYRICS OPERA) | desire | Desire | 7.69 | 7.35 | 6.49 | UNPRIMED (ORIGINAL LYRICS FADO) | sad | sad | 1.61 | 4.13 | 3.45 | UNPRIMED (ORIGINAL LYRICS HEAY METAL) | guilt | Guilt | 2.14 | 5.36 | 2.96 | UNPRIMED (ORIGINAL LYRICS HIP HOP) | displeasure | displeasure | #N/A | #N/A | #N/A | UNPRIMED (ORIINAL LYRICS BOLERO) | love | love | 8.72 | 6.44 | 7.11 | UNPRIMED (ORIGINAL LYRICS KOTO) | devotion | devotion | #N/A | #N/A | #N/A | UNPRIMED (ORIGINAL LYRICS POP) | freedom | freedom | 7.58 | 5.52 | 6.76 | UNPRIMED (ORIGINAL LYRICS SAMBA) | insecure | insecure | 2.36 | 5.56 | 2.33 |  |  |
| CUBA |  | UP = Unprimed | movement | sensual | traditional | relax | energy | present | mainstream | beauty, majestic |  | 5 |  | 2 |  | 3 |  | 3 |  | 8 |  | 6 |  | 2 |  | 5 | 102 | UNPRIMED (ORIGINAL LYRICS OPERA) | nothing | nothing | #N/A | #N/A | #N/A | UNPRIMED (ORIGINAL LYRICS FADO) | deep | Deep | 5.08 | 4.55 | 5.37 | UNPRIMED (ORIGINAL LYRICS HEAY METAL) | fury | fury | 3.1 | 6.82 | 4.29 | UNPRIMED (ORIGINAL LYRICS HIP HOP) | preoccupied | preoccupied | 4.03 | 4.93 | 4.31 | UNPRIMED (ORIINAL LYRICS BOLERO) | beauty | beauty | 7.82 | 4.95 | 5.53 | UNPRIMED (ORIGINAL LYRICS KOTO) | hope | Hope | 7.05 | 5.44 | 5.52 | UNPRIMED (ORIGINAL LYRICS POP) | wildness | wildness | #N/A | #N/A | #N/A | UNPRIMED (ORIGINAL LYRICS SAMBA) | tranquility | tranquil | #N/A | #N/A | #N/A |  |  |
| CUBA |  | UP = Unprimed | party | sad | happy | strange | madness | dancing | happy | sophiticated |  | 5 |  | 2 |  | 6 |  | 4 |  | 2 |  | 5 |  | 7 |  | 7 | 103 | UNPRIMED (ORIGINAL LYRICS OPERA) | | #N/A | #N/A | #N/A | #N/A | UNPRIMED (ORIGINAL LYRICS FADO) | | #N/A | #N/A | #N/A | #N/A | UNPRIMED (ORIGINAL LYRICS HEAY METAL) |  | #N/A | #N/A | #N/A | #N/A | UNPRIMED (ORIGINAL LYRICS HIP HOP) |  | #N/A | #N/A | #N/A | #N/A | UNPRIMED (ORIINAL LYRICS BOLERO) |  | #N/A | #N/A | #N/A | #N/A | UNPRIMED (ORIGINAL LYRICS KOTO) |  | #N/A | #N/A | #N/A | #N/A | UNPRIMED (ORIGINAL LYRICS POP) |  | #N/A | #N/A | #N/A | #N/A | UNPRIMED (ORIGINAL LYRICS SAMBA) |  | #N/A | #N/A | #N/A | #N/A |  |  |
| CUBA |  | UP = Unprimed | movement | sensual | flavourful | peace | aggressive | young, rebellious | joy | calm |  | 5 |  | 1 |  | 4 |  | 3 |  | 8 |  | 6 |  | 1 |  | 7 | 104 | UNPRIMED (ORIGINAL LYRICS OPERA) |  | overthinking | #N/A | #N/A | #N/A | UNPRIMED (ORIGINAL LYRICS FADO) |  | confused | #N/A | #N/A | #N/A | UNPRIMED (ORIGINAL LYRICS HEAY METAL) |  | #N/A | #N/A | #N/A | #N/A | UNPRIMED (ORIGINAL LYRICS HIP HOP) |  | #N/A | #N/A | #N/A | #N/A | UNPRIMED (ORIINAL LYRICS BOLERO) |  | sadness | 2.21 | 5.21 | 2.82 | UNPRIMED (ORIGINAL LYRICS KOTO) |  | #N/A | #N/A | #N/A | #N/A | UNPRIMED (ORIGINAL LYRICS POP) |  | #N/A | #N/A | #N/A | #N/A | UNPRIMED (ORIGINAL LYRICS SAMBA) |  | #N/A | #N/A | #N/A | #N/A |  |  |
| CUBA | 101 | UP = Unprimed | joy, tranquility | yearning | traditional | peace, meditation | expressive | liberation | joy | relax |  | 5 |  | 8 |  | 4 |  | 6 |  | 8 |  | 5 |  | 4 |  | 7 |  |  |  |  |  |  |  |  |  |  |  |  |  |  |  |  |  |  |  |  |  |  |  |  |  |  |  |  |  |  |  |  |  |  |  |  |  |  |  |  |  |  |  |  |  |  |  |  |  |  |  |
| CUBA | 102 | UP = Unprimed | movement | sensual | traditional | relax | present | energy | mainstream | beauty, majestic |  | 5 |  | 2 |  | 3 |  | 3 |  | 8 |  | 6 |  | 2 |  | 5 |  |  |  |  |  |  |  |  |  |  |  |  |  |  |  |  |  |  |  |  |  |  |  |  |  |  |  |  |  |  |  |  |  |  |  |  |  |  |  |  |  |  |  |  |  |  |  |  |  |  |  |
| CUBA | 103 | UP = Unprimed | party | sad | happy | strange | dancing | madness | happy | sophiticated |  | 5 |  | 2 |  | 6 |  | 4 |  | 2 |  | 5 |  | 7 |  | 7 |  |  |  |  |  |  |  |  |  |  |  |  |  |  |  |  |  |  |  |  |  |  |  |  |  |  |  |  |  |  |  |  |  |  |  |  |  |  |  |  |  |  |  |  |  |  |  |  |  |  |  |
| CUBA | 104 | UP = Unprimed | movement | sensual | flavourful | peace | young, rebellious | aggressive | joy | calm |  | 5 |  | 1 |  | 4 |  | 3 |  | 8 |  | 6 |  | 1 |  | 7 |  |  |  |  |  |  |  |  |  |  |  |  |  |  |  |  |  |  |  |  |  |  |  |  |  |  |  |  |  |  |  |  |  |  |  |  |  |  |  |  |  |  |  |  |  |  |  |  |  |  |  |
|  |  |  |  |  |  |  |  |  |  |  |  |  |  |  |  |  |  |  |  |  |  |  |  |  |  |  |  |  |  |  |  |  |  |  |  |  |  |  |  |  |  |  |  |  |  |  |  |  |  |  |  |  |  |  |  |  |  |  |  |  |  |  |  |  |  |  |  |  |  |  |  |  |  |  |  |  |  |
|  |  |  |  |  |  |  |  |  |  |  |  |  |  |  |  |  |  |  |  |  |  |  |  |  |  |  |  |  |  |  |  |  |  |  |  |  |  |  |  |  |  |  |  |  |  |  |  |  |  |  |  |  |  |  |  |  |  |  |  |  |  |  |  |  |  |  |  |  |  |  |  |  |  |  |  |  |  |
|  |  |  |  |  |  |  |  |  |  |  |  |  |  |  |  |  |  |  |  |  |  |  |  |  |  |  |  |  |  |  |  |  |  |  |  |  |  |  |  |  |  |  |  |  |  |  |  |  |  |  |  |  |  |  |  |  |  |  |  |  |  |  |  |  |  |  |  |  |  |  |  |  |  |  |  |  |  |
|  |  |  |  |  |  |  |  |  |  |  |  |  |  |  |  |  |  |  |  |  |  |  |  |  |  |  |  |  |  |  |  |  |  |  |  |  |  |  |  |  |  |  |  |  |  |  |  |  |  |  |  |  |  |  |  |  |  |  |  |  |  |  |  |  |  |  |  |  |  |  |  |  |  |  |  |  |  |
|  |  |  |  |  |  |  |  |  |  |  |  |  |  |  |  |  |  |  |  |  |  |  |  |  |  |  |  |  |  |  |  |  |  |  |  |  |  |  |  |  |  |  |  |  |  |  |  |  |  |  |  |  |  |  |  |  |  |  |  |  |  |  |  |  |  |  |  |  |  |  |  |  |  |  |  |  |  |
|  |  |  |  |  |  |  |  |  |  |  |  |  |  |  |  |  |  |  |  |  |  |  |  |  |  |  |  |  |  |  |  |  |  |  |  |  |  |  |  |  |  |  |  |  |  |  |  |  |  |  |  |  |  |  |  |  |  |  |  |  |  |  |  |  |  |  |  |  |  |  |  |  |  |  |  |  |  |
|  |  |  |  |  |  |  |  |  |  |  |  |  |  |  |  |  |  |  |  |  |  |  |  |  |  |  |  |  |  |  |  |  |  |  |  |  |  |  |  |  |  |  |  |  |  |  |  |  |  |  |  |  |  |  |  |  |  |  |  |  |  |  |  |  |  |  |  |  |  |  |  |  |  |  |  |  |  |
|  |  |  |  |  |  |  |  |  |  |  |  |  |  |  |  |  |  |  |  |  |  |  |  |  |  |  |  |  |  |  |  |  |  |  |  |  |  |  |  |  |  |  |  |  |  |  |  |  |  |  |  |  |  |  |  |  |  |  |  |  |  |  |  |  |  |  |  |  |  |  |  |  |  |  |  |  |  |
|  |  |  |  |  |  |  |  |  |  |  |  |  |  |  |  |  |  |  |  |  |  |  |  |  |  |  |  |  |  |  |  |  |  |  |  |  |  |  |  |  |  |  |  |  |  |  |  |  |  |  |  |  |  |  |  |  |  |  |  |  |  |  |  |  |  |  |  |  |  |  |  |  |  |  |  |  |  |
|  |  |  |  |  |  |  |  |  |  |  |  |  |  |  |  |  |  |  |  |  |  |  |  |  |  |  |  |  |  |  |  |  |  |  |  |  |  |  |  |  |  |  |  |  |  |  |  |  |  |  |  |  |  |  |  |  |  |  |  |  |  |  |  |  |  |  |  |  |  |  |  |  |  |  |  |  |  |
|  |  |  |  |  |  |  |  |  |  |  |  |  |  |  |  |  |  |  |  |  |  |  |  |  |  |  |  |  |  |  |  |  |  |  |  |  |  |  |  |  |  |  |  |  |  |  |  |  |  |  |  |  |  |  |  |  |  |  |  |  |  |  |  |  |  |  |  |  |  |  |  |  |  |  |  |  |  |
|  |  |  |  |  |  |  |  |  |  |  |  |  |  |  |  |  |  |  |  |  |  |  |  |  |  |  |  |  |  |  |  |  |  |  |  |  |  |  |  |  |  |  |  |  |  |  |  |  |  |  |  |  |  |  |  |  |  |  |  |  |  |  |  |  |  |  |  |  |  |  |  |  |  |  | Cleansed |  |  |
|  |  |  |  |  |  |  |  |  |  |  |  |  |  |  |  |  |  |  |  |  |  |  |  |  |  |  |  |  |  |  |  |  |  |  |  |  |  |  |  |  |  |  |  |  |  |  |  |  |  |  |  |  |  |  |  |  |  |  |  |  |  |  |  |  |  |  |  |  |  |  |  |  |  |  |  |  |  |
|  |  |  |  |  |  |  |  |  |  |  |  |  |  |  |  |  |  |  |  |  |  |  |  |  |  |  |  |  |  |  |  |  |  |  |  |  |  |  |  |  |  |  |  |  |  |  |  |  |  |  |  |  |  |  |  |  |  |  |  |  |  |  |  |  |  |  |  |  |  |  |  |  |  |  |  |  |  |
|  |  |  |  |  |  |  |  |  |  |  |  |  |  |  |  |  |  |  |  |  |  |  |  |  |  |  |  |  |  |  |  |  |  |  |  |  |  |  |  |  |  |  |  |  |  |  |  |  |  |  |  |  |  |  |  |  |  |  |  |  |  |  |  |  |  |  |  |  |  |  |  |  |  |  |  |  |  |
|  |  |  |  |  |  |  |  |  |  |  |  |  |  |  |  |  |  |  |  |  |  |  |  |  |  |  |  |  |  |  |  |  |  |  |  |  |  |  |  |  |  |  |  |  |  |  |  |  |  |  |  |  |  |  |  |  |  |  |  |  |  |  |  |  |  |  |  |  |  |  |  |  |  |  |  |  |  |
|  |  |  |  |  |  |  |  |  |  |  |  |  |  |  |  |  |  |  |  |  |  |  |  |  |  |  |  |  |  |  |  |  |  |  |  |  |  |  |  |  |  |  |  |  |  |  |  |  |  |  |  |  |  |  |  |  |  |  |  |  |  |  |  |  |  |  |  |  |  |  |  |  |  |  |  |  |  |
|  |  |  |  |  |  |  |  |  |  |  |  |  |  |  |  |  |  |  |  |  |  |  |  |  |  |  |  |  |  |  |  |  |  |  |  |  |  |  |  |  |  |  |  |  |  |  |  |  |  |  |  |  |  |  |  |  |  |  |  |  |  |  |  |  |  |  |  |  |  |  |  |  |  |  | holy |  |  |
|  |  |  |  |  |  |  |  |  |  |  |  |  |  |  |  |  |  |  |  |  |  |  |  |  |  |  |  |  |  |  |  |  |  |  |  |  |  |  |  |  |  |  |  |  |  |  |  |  |  |  |  |  |  |  |  |  |  |  |  |  |  |  |  |  |  |  |  |  |  |  |  |  |  |  |  |  |  |
|  |  |  |  |  |  |  |  |  |  |  |  |  |  |  |  |  |  |  |  |  |  |  |  |  |  |  |  |  |  |  |  |  |  |  |  |  |  |  |  |  |  |  |  |  |  |  |  |  |  |  |  |  |  |  |  |  |  |  |  |  |  |  |  |  |  |  |  |  |  |  |  |  |  |  |  |  |  |
|  |  |  |  |  |  |  |  |  |  |  |  |  |  |  |  |  |  |  |  |  |  |  |  |  |  |  |  |  |  |  |  |  |  |  |  |  |  |  |  |  |  |  |  |  |  |  |  |  |  |  |  |  |  |  |  |  |  |  |  |  |  |  |  |  |  |  |  |  |  |  |  |  |  |  |  |  |  |
|  |  |  |  |  |  |  |  |  |  |  |  |  |  |  |  |  |  |  |  |  |  |  |  |  |  |  |  |  |  |  |  |  |  |  |  |  |  |  |  |  |  |  |  |  |  |  |  |  |  |  |  |  |  |  |  |  |  |  |  |  |  |  |  |  |  |  |  |  |  |  |  |  |  |  |  |  |  |
|  |  |  |  |  |  |  |  |  |  |  |  |  |  |  |  |  |  |  |  |  |  |  |  |  |  |  |  |  |  |  |  |  |  |  |  |  |  |  |  |  |  |  |  |  |  |  |  |  |  |  |  |  |  |  |  |  |  |  |  |  |  |  |  |  |  |  |  |  |  |  |  |  |  |  |  |  |  |
|  |  |  |  |  |  |  |  |  |  |  |  |  |  |  |  |  |  |  |  |  |  |  |  |  |  |  |  |  |  |  |  |  |  |  |  |  |  |  |  |  |  |  |  |  |  |  |  |  |  |  |  |  |  |  |  |  |  |  |  |  |  |  |  |  |  |  |  |  |  |  |  |  |  |  | supersticious |  |  |
|  |  |  |  |  |  |  |  |  |  |  |  |  |  |  |  |  |  |  |  |  |  |  |  |  |  |  |  |  |  |  |  |  |  |  |  |  |  |  |  |  |  |  |  |  |  |  |  |  |  |  |  |  |  |  |  |  |  |  |  |  |  |  |  |  |  |  |  |  |  |  |  |  |  |  |  |  |  |
|  |  |  |  |  |  |  |  |  |  |  |  |  |  |  |  |  |  |  |  |  |  |  |  |  |  |  |  |  |  |  |  |  |  |  |  |  |  |  |  |  |  |  |  |  |  |  |  |  |  |  |  |  |  |  |  |  |  |  |  |  |  |  |  |  |  |  |  |  |  |  |  |  |  |  |  |  |  |
|  |  |  |  |  |  |  |  |  |  |  |  |  |  |  |  |  |  |  |  |  |  |  |  |  |  |  |  |  |  |  |  |  |  |  |  |  |  |  |  |  |  |  |  |  |  |  |  |  |  |  |  |  |  |  |  |  |  |  |  |  |  |  |  |  |  |  |  |  |  |  |  |  |  |  |  |  |  |
|  |  |  |  |  |  |  |  |  |  |  |  |  |  |  |  |  |  |  |  |  |  |  |  |  |  |  |  |  |  |  |  |  |  |  |  |  |  |  |  |  |  |  |  |  |  |  |  |  |  |  |  |  |  |  |  |  |  |  |  |  |  |  |  |  |  |  |  |  |  |  |  |  |  |  | holy |  |  |
|  |  |  |  |  |  |  |  |  |  |  |  |  |  |  |  |  |  |  |  |  |  |  |  |  |  |  |  |  |  |  |  |  |  |  |  |  |  |  |  |  |  |  |  |  |  |  |  |  |  |  |  |  |  |  |  |  |  |  |  |  |  |  |  |  |  |  |  |  |  |  |  |  |  |  |  |  |  |
|  |  |  |  |  |  |  |  |  |  |  |  |  |  |  |  |  |  |  |  |  |  |  |  |  |  |  |  |  |  |  |  |  |  |  |  |  |  |  |  |  |  |  |  |  |  |  |  |  |  |  |  |  |  |  |  |  |  |  |  |  |  |  |  |  |  |  |  |  |  |  |  |  |  |  | wired |  |  |
|  |  |  |  |  |  |  |  |  |  |  |  |  |  |  |  |  |  |  |  |  |  |  |  |  |  |  |  |  |  |  |  |  |  |  |  |  |  |  |  |  |  |  |  |  |  |  |  |  |  |  |  |  |  |  |  |  |  |  |  |  |  |  |  |  |  |  |  |  |  |  |  |  |  |  |  |  |  |
|  |  |  |  |  |  |  |  |  |  |  |  |  |  |  |  |  |  |  |  |  |  |  |  |  |  |  |  |  |  |  |  |  |  |  |  |  |  |  |  |  |  |  |  |  |  |  |  |  |  |  |  |  |  |  |  |  |  |  |  |  |  |  |  |  |  |  |  |  |  |  |  |  |  |  | confused |  |  |
|  |  |  |  |  |  |  |  |  |  |  |  |  |  |  |  |  |  |  |  |  |  |  |  |  |  |  |  |  |  |  |  |  |  |  |  |  |  |  |  |  |  |  |  |  |  |  |  |  |  |  |  |  |  |  |  |  |  |  |  |  |  |  |  |  |  |  |  |  |  |  |  |  |  |  |  |  |  |
|  |  |  |  |  |  |  |  |  |  |  |  |  |  |  |  |  |  |  |  |  |  |  |  |  |  |  |  |  |  |  |  |  |  |  |  |  |  |  |  |  |  |  |  |  |  |  |  |  |  |  |  |  |  |  |  |  |  |  |  |  |  |  |  |  |  |  |  |  |  |  |  |  |  |  |  |  |  |
|  |  |  |  |  |  |  |  |  |  |  |  |  |  |  |  |  |  |  |  |  |  |  |  |  |  |  |  |  |  |  |  |  |  |  |  |  |  |  |  |  |  |  |  |  |  |  |  |  |  |  |  |  |  |  |  |  |  |  |  |  |  |  |  |  |  |  |  |  |  |  |  |  |  |  | Interested |  |  |
|  |  |  |  |  |  |  |  |  |  |  |  |  |  |  |  |  |  |  |  |  |  |  |  |  |  |  |  |  |  |  |  |  |  |  |  |  |  |  |  |  |  |  |  |  |  |  |  |  |  |  |  |  |  |  |  |  |  |  |  |  |  |  |  |  |  |  |  |  |  |  |  |  |  |  | Reflection |  |  |
|  |  |  |  |  |  |  |  |  |  |  |  |  |  |  |  |  |  |  |  |  |  |  |  |  |  |  |  |  |  |  |  |  |  |  |  |  |  |  |  |  |  |  |  |  |  |  |  |  |  |  |  |  |  |  |  |  |  |  |  |  |  |  |  |  |  |  |  |  |  |  |  |  |  |  |  |  |  |
|  |  |  |  |  |  |  |  |  |  |  |  |  |  |  |  |  |  |  |  |  |  |  |  |  |  |  |  |  |  |  |  |  |  |  |  |  |  |  |  |  |  |  |  |  |  |  |  |  |  |  |  |  |  |  |  |  |  |  |  |  |  |  |  |  |  |  |  |  |  |  |  |  |  |  |  |  |  |
|  |  |  |  |  |  |  |  |  |  |  |  |  |  |  |  |  |  |  |  |  |  |  |  |  |  |  |  |  |  |  |  |  |  |  |  |  |  |  |  |  |  |  |  |  |  |  |  |  |  |  |  |  |  |  |  |  |  |  |  |  |  |  |  |  |  |  |  |  |  |  |  |  |  |  |  |  |  |
|  |  |  |  |  |  |  |  |  |  |  |  |  |  |  |  |  |  |  |  |  |  |  |  |  |  |  |  |  |  |  |  |  |  |  |  |  |  |  |  |  |  |  |  |  |  |  |  |  |  |  |  |  |  |  |  |  |  |  |  |  |  |  |  |  |  |  |  |  |  |  |  |  |  |  |  |  |  |
|  |  |  |  |  |  |  |  |  |  |  |  |  |  |  |  |  |  |  |  |  |  |  |  |  |  |  |  |  |  |  |  |  |  |  |  |  |  |  |  |  |  |  |  |  |  |  |  |  |  |  |  |  |  |  |  |  |  |  |  |  |  |  |  |  |  |  |  |  |  |  |  |  |  |  |  |  |  |
|  |  |  |  |  |  |  |  |  |  |  |  |  |  |  |  |  |  |  |  |  |  |  |  |  |  |  |  |  |  |  |  |  |  |  |  |  |  |  |  |  |  |  |  |  |  |  |  |  |  |  |  |  |  |  |  |  |  |  |  |  |  |  |  |  |  |  |  |  |  |  |  |  |  |  | holy |  |  |
|  |  |  |  |  |  |  |  |  |  |  |  |  |  |  |  |  |  |  |  |  |  |  |  |  |  |  |  |  |  |  |  |  |  |  |  |  |  |  |  |  |  |  |  |  |  |  |  |  |  |  |  |  |  |  |  |  |  |  |  |  |  |  |  |  |  |  |  |  |  |  |  |  |  |  | dark |  |  |
|  |  |  |  |  |  |  |  |  |  |  |  |  |  |  |  |  |  |  |  |  |  |  |  |  |  |  |  |  |  |  |  |  |  |  |  |  |  |  |  |  |  |  |  |  |  |  |  |  |  |  |  |  |  |  |  |  |  |  |  |  |  |  |  |  |  |  |  |  |  |  |  |  |  |  |  |  |  |
|  |  |  |  |  |  |  |  |  |  |  |  |  |  |  |  |  |  |  |  |  |  |  |  |  |  |  |  |  |  |  |  |  |  |  |  |  |  |  |  |  |  |  |  |  |  |  |  |  |  |  |  |  |  |  |  |  |  |  |  |  |  |  |  |  |  |  |  |  |  |  |  |  |  |  |  |  |  |
|  |  |  |  |  |  |  |  |  |  |  |  |  |  |  |  |  |  |  |  |  |  |  |  |  |  |  |  |  |  |  |  |  |  |  |  |  |  |  |  |  |  |  |  |  |  |  |  |  |  |  |  |  |  |  |  |  |  |  |  |  |  |  |  |  |  |  |  |  |  |  |  |  |  |  |  |  |  |
|  |  |  |  |  |  |  |  |  |  |  |  |  |  |  |  |  |  |  |  |  |  |  |  |  |  |  |  |  |  |  |  |  |  |  |  |  |  |  |  |  |  |  |  |  |  |  |  |  |  |  |  |  |  |  |  |  |  |  |  |  |  |  |  |  |  |  |  |  |  |  |  |  |  |  |  |  |  |
|  |  |  |  |  |  |  |  |  |  |  |  |  |  |  |  |  |  |  |  |  |  |  |  |  |  |  |  |  |  |  |  |  |  |  |  |  |  |  |  |  |  |  |  |  |  |  |  |  |  |  |  |  |  |  |  |  |  |  |  |  |  |  |  |  |  |  |  |  |  |  |  |  |  |  | Superstitious |  |  |
|  |  |  |  |  |  |  |  |  |  |  |  |  |  |  |  |  |  |  |  |  |  |  |  |  |  |  |  |  |  |  |  |  |  |  |  |  |  |  |  |  |  |  |  |  |  |  |  |  |  |  |  |  |  |  |  |  |  |  |  |  |  |  |  |  |  |  |  |  |  |  |  |  |  |  |  |  |  |
|  |  |  |  |  |  |  |  |  |  |  |  |  |  |  |  |  |  |  |  |  |  |  |  |  |  |  |  |  |  |  |  |  |  |  |  |  |  |  |  |  |  |  |  |  |  |  |  |  |  |  |  |  |  |  |  |  |  |  |  |  |  |  |  |  |  |  |  |  |  |  |  |  |  |  |  |  |  |
|  |  |  |  |  |  |  |  |  |  |  |  |  |  |  |  |  |  |  |  |  |  |  |  |  |  |  |  |  |  |  |  |  |  |  |  |  |  |  |  |  |  |  |  |  |  |  |  |  |  |  |  |  |  |  |  |  |  |  |  |  |  |  |  |  |  |  |  |  |  |  |  |  |  |  |  |  |  |
|  |  |  |  |  |  |  |  |  |  |  |  |  |  |  |  |  |  |  |  |  |  |  |  |  |  |  |  |  |  |  |  |  |  |  |  |  |  |  |  |  |  |  |  |  |  |  |  |  |  |  |  |  |  |  |  |  |  |  |  |  |  |  |  |  |  |  |  |  |  |  |  |  |  |  |  |  |  |
|  |  |  |  |  |  |  |  |  |  |  |  |  |  |  |  |  |  |  |  |  |  |  |  |  |  |  |  |  |  |  |  |  |  |  |  |  |  |  |  |  |  |  |  |  |  |  |  |  |  |  |  |  |  |  |  |  |  |  |  |  |  |  |  |  |  |  |  |  |  |  |  |  |  |  |  |  |  |
|  |  |  |  |  |  |  |  |  |  |  |  |  |  |  |  |  |  |  |  |  |  |  |  |  |  |  |  |  |  |  |  |  |  |  |  |  |  |  |  |  |  |  |  |  |  |  |  |  |  |  |  |  |  |  |  |  |  |  |  |  |  |  |  |  |  |  |  |  |  |  |  |  |  |  |  |  |  |
|  |  |  |  |  |  |  |  |  |  |  |  |  |  |  |  |  |  |  |  |  |  |  |  |  |  |  |  |  |  |  |  |  |  |  |  |  |  |  |  |  |  |  |  |  |  |  |  |  |  |  |  |  |  |  |  |  |  |  |  |  |  |  |  |  |  |  |  |  |  |  |  |  |  |  |  |  |  |
|  |  |  |  |  |  |  |  |  |  |  |  |  |  |  |  |  |  |  |  |  |  |  |  |  |  |  |  |  |  |  |  |  |  |  |  |  |  |  |  |  |  |  |  |  |  |  |  |  |  |  |  |  |  |  |  |  |  |  |  |  |  |  |  |  |  |  |  |  |  |  |  |  |  |  | none |  |  |
|  |  |  |  |  |  |  |  |  |  |  |  |  |  |  |  |  |  |  |  |  |  |  |  |  |  |  |  |  |  |  |  |  |  |  |  |  |  |  |  |  |  |  |  |  |  |  |  |  |  |  |  |  |  |  |  |  |  |  |  |  |  |  |  |  |  |  |  |  |  |  |  |  |  |  |  |  |  |
|  |  |  |  |  |  |  |  |  |  |  |  |  |  |  |  |  |  |  |  |  |  |  |  |  |  |  |  |  |  |  |  |  |  |  |  |  |  |  |  |  |  |  |  |  |  |  |  |  |  |  |  |  |  |  |  |  |  |  |  |  |  |  |  |  |  |  |  |  |  |  |  |  |  |  |  |  |  |
|  |  |  |  |  |  |  |  |  |  |  |  |  |  |  |  |  |  |  |  |  |  |  |  |  |  |  |  |  |  |  |  |  |  |  |  |  |  |  |  |  |  |  |  |  |  |  |  |  |  |  |  |  |  |  |  |  |  |  |  |  |  |  |  |  |  |  |  |  |  |  |  |  |  |  |  |  |  |
|  |  |  |  |  |  |  |  |  |  |  |  |  |  |  |  |  |  |  |  |  |  |  |  |  |  |  |  |  |  |  |  |  |  |  |  |  |  |  |  |  |  |  |  |  |  |  |  |  |  |  |  |  |  |  |  |  |  |  |  |  |  |  |  |  |  |  |  |  |  |  |  |  |  |  |  |  |  |
|  |  |  |  |  |  |  |  |  |  |  |  |  |  |  |  |  |  |  |  |  |  |  |  |  |  |  |  |  |  |  |  |  |  |  |  |  |  |  |  |  |  |  |  |  |  |  |  |  |  |  |  |  |  |  |  |  |  |  |  |  |  |  |  |  |  |  |  |  |  |  |  |  |  |  |  |  |  |
|  |  |  |  |  |  |  |  |  |  |  |  |  |  |  |  |  |  |  |  |  |  |  |  |  |  |  |  |  |  |  |  |  |  |  |  |  |  |  |  |  |  |  |  |  |  |  |  |  |  |  |  |  |  |  |  |  |  |  |  |  |  |  |  |  |  |  |  |  |  |  |  |  |  |  |  |  |  |
|  |  |  |  |  |  |  |  |  |  |  |  |  |  |  |  |  |  |  |  |  |  |  |  |  |  |  |  |  |  |  |  |  |  |  |  |  |  |  |  |  |  |  |  |  |  |  |  |  |  |  |  |  |  |  |  |  |  |  |  |  |  |  |  |  |  |  |  |  |  |  |  |  |  |  |  |  |  |
|  |  |  |  |  |  |  |  |  |  |  |  |  |  |  |  |  |  |  |  |  |  |  |  |  |  |  |  |  |  |  |  |  |  |  |  |  |  |  |  |  |  |  |  |  |  |  |  |  |  |  |  |  |  |  |  |  |  |  |  |  |  |  |  |  |  |  |  |  |  |  |  |  |  |  |  |  |  |
|  |  |  |  |  |  |  |  |  |  |  |  |  |  |  |  |  |  |  |  |  |  |  |  |  |  |  |  |  |  |  |  |  |  |  |  |  |  |  |  |  |  |  |  |  |  |  |  |  |  |  |  |  |  |  |  |  |  |  |  |  |  |  |  |  |  |  |  |  |  |  |  |  |  |  |  |  |  |
|  |  |  |  |  |  |  |  |  |  |  |  |  |  |  |  |  |  |  |  |  |  |  |  |  |  |  |  |  |  |  |  |  |  |  |  |  |  |  |  |  |  |  |  |  |  |  |  |  |  |  |  |  |  |  |  |  |  |  |  |  |  |  |  |  |  |  |  |  |  |  |  |  |  |  |  |  |  |
|  |  |  |  |  |  |  |  |  |  |  |  |  |  |  |  |  |  |  |  |  |  |  |  |  |  |  |  |  |  |  |  |  |  |  |  |  |  |  |  |  |  |  |  |  |  |  |  |  |  |  |  |  |  |  |  |  |  |  |  |  |  |  |  |  |  |  |  |  |  |  |  |  |  |  |  |  |  |
|  |  |  |  |  |  |  |  |  |  |  |  |  |  |  |  |  |  |  |  |  |  |  |  |  |  |  |  |  |  |  |  |  |  |  |  |  |  |  |  |  |  |  |  |  |  |  |  |  |  |  |  |  |  |  |  |  |  |  |  |  |  |  |  |  |  |  |  |  |  |  |  |  |  |  |  |  |  |
|  |  |  |  |  |  |  |  |  |  |  |  |  |  |  |  |  |  |  |  |  |  |  |  |  |  |  |  |  |  |  |  |  |  |  |  |  |  |  |  |  |  |  |  |  |  |  |  |  |  |  |  |  |  |  |  |  |  |  |  |  |  |  |  |  |  |  |  |  |  |  |  |  |  |  |  |  |  |
|  |  |  |  |  |  |  |  |  |  |  |  |  |  |  |  |  |  |  |  |  |  |  |  |  |  |  |  |  |  |  |  |  |  |  |  |  |  |  |  |  |  |  |  |  |  |  |  |  |  |  |  |  |  |  |  |  |  |  |  |  |  |  |  |  |  |  |  |  |  |  |  |  |  |  |  |  |  |
|  |  |  |  |  |  |  |  |  |  |  |  |  |  |  |  |  |  |  |  |  |  |  |  |  |  |  |  |  |  |  |  |  |  |  |  |  |  |  |  |  |  |  |  |  |  |  |  |  |  |  |  |  |  |  |  |  |  |  |  |  |  |  |  |  |  |  |  |  |  |  |  |  |  |  |  |  |  |
|  |  |  |  |  |  |  |  |  |  |  |  |  |  |  |  |  |  |  |  |  |  |  |  |  |  |  |  |  |  |  |  |  |  |  |  |  |  |  |  |  |  |  |  |  |  |  |  |  |  |  |  |  |  |  |  |  |  |  |  |  |  |  |  |  |  |  |  |  |  |  |  |  |  |  |  |  |  |
|  |  |  |  |  |  |  |  |  |  |  |  |  |  |  |  |  |  |  |  |  |  |  |  |  |  |  |  |  |  |  |  |  |  |  |  |  |  |  |  |  |  |  |  |  |  |  |  |  |  |  |  |  |  |  |  |  |  |  |  |  |  |  |  |  |  |  |  |  |  |  |  |  |  |  |  |  |  |
|  |  |  |  |  |  |  |  |  |  |  |  |  |  |  |  |  |  |  |  |  |  |  |  |  |  |  |  |  |  |  |  |  |  |  |  |  |  |  |  |  |  |  |  |  |  |  |  |  |  |  |  |  |  |  |  |  |  |  |  |  |  |  |  |  |  |  |  |  |  |  |  |  |  |  |  |  |  |
|  |  |  |  |  |  |  |  |  |  |  |  |  |  |  |  |  |  |  |  |  |  |  |  |  |  |  |  |  |  |  |  |  |  |  |  |  |  |  |  |  |  |  |  |  |  |  |  |  |  |  |  |  |  |  |  |  |  |  |  |  |  |  |  |  |  |  |  |  |  |  |  |  |  |  |  |  |  |
|  |  |  |  |  |  |  |  |  |  |  |  |  |  |  |  |  |  |  |  |  |  |  |  |  |  |  |  |  |  |  |  |  |  |  |  |  |  |  |  |  |  |  |  |  |  |  |  |  |  |  |  |  |  |  |  |  |  |  |  |  |  |  |  |  |  |  |  |  |  |  |  |  |  |  |  |  |  |
|  |  |  |  |  |  |  |  |  |  |  |  |  |  |  |  |  |  |  |  |  |  |  |  |  |  |  |  |  |  |  |  |  |  |  |  |  |  |  |  |  |  |  |  |  |  |  |  |  |  |  |  |  |  |  |  |  |  |  |  |  |  |  |  |  |  |  |  |  |  |  |  |  |  |  |  |  |  |
|  |  |  |  |  |  |  |  |  |  |  |  |  |  |  |  |  |  |  |  |  |  |  |  |  |  |  |  |  |  |  |  |  |  |  |  |  |  |  |  |  |  |  |  |  |  |  |  |  |  |  |  |  |  |  |  |  |  |  |  |  |  |  |  |  |  |  |  |  |  |  |  |  |  |  |  |  |  |
|  |  |  |  |  |  |  |  |  |  |  |  |  |  |  |  |  |  |  |  |  |  |  |  |  |  |  |  |  |  |  |  |  |  |  |  |  |  |  |  |  |  |  |  |  |  |  |  |  |  |  |  |  |  |  |  |  |  |  |  |  |  |  |  |  |  |  |  |  |  |  |  |  |  |  |  |  |  |
|  |  |  |  |  |  |  |  |  |  |  |  |  |  |  |  |  |  |  |  |  |  |  |  |  |  |  |  |  |  |  |  |  |  |  |  |  |  |  |  |  |  |  |  |  |  |  |  |  |  |  |  |  |  |  |  |  |  |  |  |  |  |  |  |  |  |  |  |  |  |  |  |  |  |  |  |  |  |
|  |  |  |  |  |  |  |  |  |  |  |  |  |  |  |  |  |  |  |  |  |  |  |  |  |  |  |  |  |  |  |  |  |  |  |  |  |  |  |  |  |  |  |  |  |  |  |  |  |  |  |  |  |  |  |  |  |  |  |  |  |  |  |  |  |  |  |  |  |  |  |  |  |  |  |  |  |  |
|  |  |  |  |  |  |  |  |  |  |  |  |  |  |  |  |  |  |  |  |  |  |  |  |  |  |  |  |  |  |  |  |  |  |  |  |  |  |  |  |  |  |  |  |  |  |  |  |  |  |  |  |  |  |  |  |  |  |  |  |  |  |  |  |  |  |  |  |  |  |  |  |  |  |  |  |  |  |
|  |  |  |  |  |  |  |  |  |  |  |  |  |  |  |  |  |  |  |  |  |  |  |  |  |  |  |  |  |  |  |  |  |  |  |  |  |  |  |  |  |  |  |  |  |  |  |  |  |  |  |  |  |  |  |  |  |  |  |  |  |  |  |  |  |  |  |  |  |  |  |  |  |  |  |  |  |  |
|  |  |  |  |  |  |  |  |  |  |  |  |  |  |  |  |  |  |  |  |  |  |  |  |  |  |  |  |  |  |  |  |  |  |  |  |  |  |  |  |  |  |  |  |  |  |  |  |  |  |  |  |  |  |  |  |  |  |  |  |  |  |  |  |  |  |  |  |  |  |  |  |  |  |  |  |  |  |
|  |  |  |  |  |  |  |  |  |  |  |  |  |  |  |  |  |  |  |  |  |  |  |  |  |  |  |  |  |  |  |  |  |  |  |  |  |  |  |  |  |  |  |  |  |  |  |  |  |  |  |  |  |  |  |  |  |  |  |  |  |  |  |  |  |  |  |  |  |  |  |  |  |  |  |  |  |  |
|  |  |  |  |  |  |  |  |  |  |  |  |  |  |  |  |  |  |  |  |  |  |  |  |  |  |  |  |  |  |  |  |  |  |  |  |  |  |  |  |  |  |  |  |  |  |  |  |  |  |  |  |  |  |  |  |  |  |  |  |  |  |  |  |  |  |  |  |  |  |  |  |  |  |  |  |  |  |
|  |  |  |  |  |  |  |  |  |  |  |  |  |  |  |  |  |  |  |  |  |  |  |  |  |  |  |  |  |  |  |  |  |  |  |  |  |  |  |  |  |  |  |  |  |  |  |  |  |  |  |  |  |  |  |  |  |  |  |  |  |  |  |  |  |  |  |  |  |  |  |  |  |  |  |  |  |  |
|  |  |  |  |  |  |  |  |  |  |  |  |  |  |  |  |  |  |  |  |  |  |  |  |  |  |  |  |  |  |  |  |  |  |  |  |  |  |  |  |  |  |  |  |  |  |  |  |  |  |  |  |  |  |  |  |  |  |  |  |  |  |  |  |  |  |  |  |  |  |  |  |  |  |  |  |  |  |
|  |  |  |  |  |  |  |  |  |  |  |  |  |  |  |  |  |  |  |  |  |  |  |  |  |  |  |  |  |  |  |  |  |  |  |  |  |  |  |  |  |  |  |  |  |  |  |  |  |  |  |  |  |  |  |  |  |  |  |  |  |  |  |  |  |  |  |  |  |  |  |  |  |  |  |  |  |  |
|  |  |  |  |  |  |  |  |  |  |  |  |  |  |  |  |  |  |  |  |  |  |  |  |  |  |  |  |  |  |  |  |  |  |  |  |  |  |  |  |  |  |  |  |  |  |  |  |  |  |  |  |  |  |  |  |  |  |  |  |  |  |  |  |  |  |  |  |  |  |  |  |  |  |  |  |  |  |
|  |  |  |  |  |  |  |  |  |  |  |  |  |  |  |  |  |  |  |  |  |  |  |  |  |  |  |  |  |  |  |  |  |  |  |  |  |  |  |  |  |  |  |  |  |  |  |  |  |  |  |  |  |  |  |  |  |  |  |  |  |  |  |  |  |  |  |  |  |  |  |  |  |  |  |  |  |  |
|  |  |  |  |  |  |  |  |  |  |  |  |  |  |  |  |  |  |  |  |  |  |  |  |  |  |  |  |  |  |  |  |  |  |  |  |  |  |  |  |  |  |  |  |  |  |  |  |  |  |  |  |  |  |  |  |  |  |  |  |  |  |  |  |  |  |  |  |  |  |  |  |  |  |  |  |  |  |
|  |  |  |  |  |  |  |  |  |  |  |  |  |  |  |  |  |  |  |  |  |  |  |  |  |  |  |  |  |  |  |  |  |  |  |  |  |  |  |  |  |  |  |  |  |  |  |  |  |  |  |  |  |  |  |  |  |  |  |  |  |  |  |  |  |  |  |  |  |  |  |  |  |  |  |  |  |  |
|  |  |  |  |  |  |  |  |  |  |  |  |  |  |  |  |  |  |  |  |  |  |  |  |  |  |  |  |  |  |  |  |  |  |  |  |  |  |  |  |  |  |  |  |  |  |  |  |  |  |  |  |  |  |  |  |  |  |  |  |  |  |  |  |  |  |  |  |  |  |  |  |  |  |  |  |  |  |
|  |  |  |  |  |  |  |  |  |  |  |  |  |  |  |  |  |  |  |  |  |  |  |  |  |  |  |  |  |  |  |  |  |  |  |  |  |  |  |  |  |  |  |  |  |  |  |  |  |  |  |  |  |  |  |  |  |  |  |  |  |  |  |  |  |  |  |  |  |  |  |  |  |  |  |  |  |  |
|  |  |  |  |  |  |  |  |  |  |  |  |  |  |  |  |  |  |  |  |  |  |  |  |  |  |  |  |  |  |  |  |  |  |  |  |  |  |  |  |  |  |  |  |  |  |  |  |  |  |  |  |  |  |  |  |  |  |  |  |  |  |  |  |  |  |  |  |  |  |  |  |  |  |  |  |  |  |
|  |  |  |  |  |  |  |  |  |  |  |  |  |  |  |  |  |  |  |  |  |  |  |  |  |  |  |  |  |  |  |  |  |  |  |  |  |  |  |  |  |  |  |  |  |  |  |  |  |  |  |  |  |  |  |  |  |  |  |  |  |  |  |  |  |  |  |  |  |  |  |  |  |  |  |  |  |  |
|  |  |  |  |  |  |  |  |  |  |  |  |  |  |  |  |  |  |  |  |  |  |  |  |  |  |  |  |  |  |  |  |  |  |  |  |  |  |  |  |  |  |  |  |  |  |  |  |  |  |  |  |  |  |  |  |  |  |  |  |  |  |  |  |  |  |  |  |  |  |  |  |  |  |  |  |  |  |
|  |  |  |  |  |  |  |  |  |  |  |  |  |  |  |  |  |  |  |  |  |  |  |  |  |  |  |  |  |  |  |  |  |  |  |  |  |  |  |  |  |  |  |  |  |  |  |  |  |  |  |  |  |  |  |  |  |  |  |  |  |  |  |  |  |  |  |  |  |  |  |  |  |  |  |  |  |  |
|  |  |  |  |  |  |  |  |  |  |  |  |  |  |  |  |  |  |  |  |  |  |  |  |  |  |  |  |  |  |  |  |  |  |  |  |  |  |  |  |  |  |  |  |  |  |  |  |  |  |  |  |  |  |  |  |  |  |  |  |  |  |  |  |  |  |  |  |  |  |  |  |  |  |  |  |  |  |
|  |  |  |  |  |  |  |  |  |  |  |  |  |  |  |  |  |  |  |  |  |  |  |  |  |  |  |  |  |  |  |  |  |  |  |  |  |  |  |  |  |  |  |  |  |  |  |  |  |  |  |  |  |  |  |  |  |  |  |  |  |  |  |  |  |  |  |  |  |  |  |  |  |  |  |  |  |  |
|  |  |  |  |  |  |  |  |  |  |  |  |  |  |  |  |  |  |  |  |  |  |  |  |  |  |  |  |  |  |  |  |  |  |  |  |  |  |  |  |  |  |  |  |  |  |  |  |  |  |  |  |  |  |  |  |  |  |  |  |  |  |  |  |  |  |  |  |  |  |  |  |  |  |  |  |  |  |
|  |  |  |  |  |  |  |  |  |  |  |  |  |  |  |  |  |  |  |  |  |  |  |  |  |  |  |  |  |  |  |  |  |  |  |  |  |  |  |  |  |  |  |  |  |  |  |  |  |  |  |  |  |  |  |  |  |  |  |  |  |  |  |  |  |  |  |  |  |  |  |  |  |  |  |  |  |  |
|  |  |  |  |  |  |  |  |  |  |  |  |  |  |  |  |  |  |  |  |  |  |  |  |  |  |  |  |  |  |  |  |  |  |  |  |  |  |  |  |  |  |  |  |  |  |  |  |  |  |  |  |  |  |  |  |  |  |  |  |  |  |  |  |  |  |  |  |  |  |  |  |  |  |  |  |  |  |
|  |  |  |  |  |  |  |  |  |  |  |  |  |  |  |  |  |  |  |  |  |  |  |  |  |  |  |  |  |  |  |  |  |  |  |  |  |  |  |  |  |  |  |  |  |  |  |  |  |  |  |  |  |  |  |  |  |  |  |  |  |  |  |  |  |  |  |  |  |  |  |  |  |  |  |  |  |  |
|  |  |  |  |  |  |  |  |  |  |  |  |  |  |  |  |  |  |  |  |  |  |  |  |  |  |  |  |  |  |  |  |  |  |  |  |  |  |  |  |  |  |  |  |  |  |  |  |  |  |  |  |  |  |  |  |  |  |  |  |  |  |  |  |  |  |  |  |  |  |  |  |  |  |  |  |  |  |
|  |  |  |  |  |  |  |  |  |  |  |  |  |  |  |  |  |  |  |  |  |  |  |  |  |  |  |  |  |  |  |  |  |  |  |  |  |  |  |  |  |  |  |  |  |  |  |  |  |  |  |  |  |  |  |  |  |  |  |  |  |  |  |  |  |  |  |  |  |  |  |  |  |  |  |  |  |  |
|  |  |  |  |  |  |  |  |  |  |  |  |  |  |  |  |  |  |  |  |  |  |  |  |  |  |  |  |  |  |  |  |  |  |  |  |  |  |  |  |  |  |  |  |  |  |  |  |  |  |  |  |  |  |  |  |  |  |  |  |  |  |  |  |  |  |  |  |  |  |  |  |  |  |  |  |  |  |
|  |  |  |  |  |  |  |  |  |  |  |  |  |  |  |  |  |  |  |  |  |  |  |  |  |  |  |  |  |  |  |  |  |  |  |  |  |  |  |  |  |  |  |  |  |  |  |  |  |  |  |  |  |  |  |  |  |  |  |  |  |  |  |  |  |  |  |  |  |  |  |  |  |  |  |  |  |  |
|  |  |  |  |  |  |  |  |  |  |  |  |  |  |  |  |  |  |  |  |  |  |  |  |  |  |  |  |  |  |  |  |  |  |  |  |  |  |  |  |  |  |  |  |  |  |  |  |  |  |  |  |  |  |  |  |  |  |  |  |  |  |  |  |  |  |  |  |  |  |  |  |  |  |  |  |  |  |
|  |  |  |  |  |  |  |  |  |  |  |  |  |  |  |  |  |  |  |  |  |  |  |  |  |  |  |  |  |  |  |  |  |  |  |  |  |  |  |  |  |  |  |  |  |  |  |  |  |  |  |  |  |  |  |  |  |  |  |  |  |  |  |  |  |  |  |  |  |  |  |  |  |  |  |  |  |  |
| AUSTRALIA | 122 | z5075493 | party | Romantic | Chill | Foreign | Angry | Celebration | Easy-going | Regal |  | 8 |  | 1 |  | 6 |  | 7 |  | 8 |  | 6 |  | 8 |  | 8 |  |  |  |  |  |  |  |  |  |  |  |  |  |  |  |  |  |  |  |  |  |  |  |  |  |  |  |  |  |  |  |  |  |  |  |  |  |  |  |  |  |  |  |  |  |  |  |  |  |  |  |
| AUSTRALIA | 123 | z5060340 | Love | Soft music | Relaxing | Relaxing | cool | Youth | fun | relaxing |  | 8 |  | 3 |  | 4 |  | 8 |  | 8 |  | 8 |  | 8 |  | 8 |  |  |  |  |  |  |  |  |  |  |  |  |  |  |  |  |  |  |  |  |  |  |  |  |  |  |  |  |  |  |  |  |  |  |  |  |  |  |  |  |  |  |  |  |  |  |  |  |  |  |  |
| AUSTRALIA | 124 | z5060369 | excited | sadness | energetic | meditative | energetic | chilled | happy | calm |  | 5 |  | 5 |  | 3 |  | 2 |  | 2 |  | 7 |  | 5 |  | 5 |  |  |  |  |  |  |  |  |  |  |  |  |  |  |  |  |  |  |  |  |  |  |  |  |  |  |  |  |  |  |  |  |  |  |  |  |  |  |  |  |  |  |  |  |  |  |  |  |  |  |  |
| AUSTRALIA | 125 | 3275760 | Excited | Glad | Longing | Sad | angry | Angry | Happy | classic |  | 7 |  | 8 |  | 7 |  | 7 |  | 8 |  | 7 |  | 8 |  | 8 |  |  |  |  |  |  |  |  |  |  |  |  |  |  |  |  |  |  |  |  |  |  |  |  |  |  |  |  |  |  |  |  |  |  |  |  |  |  |  |  |  |  |  |  |  |  |  |  |  |  |  |
| AUSTRALIA | 126 | z3459931 | Entertaining | Lovely | Fun | Interesting | Disgusting | Annoying | Satisfactory | Beautiful |  | 4 |  | 1 |  | 3 |  | 1 |  | 8 |  | 7 |  | 4 |  | 4 |  |  |  |  |  |  |  |  |  |  |  |  |  |  |  |  |  |  |  |  |  |  |  |  |  |  |  |  |  |  |  |  |  |  |  |  |  |  |  |  |  |  |  |  |  |  |  |  |  |  |  |
| AUSTRALIA | 127 | 5062912 |  |  |  | Asian |  |  | Happiness |  |  |  |  |  |  |  |  |  |  |  |  |  |  |  |  |  |  |  |  |  |  |  |  |  |  |  |  |  |  |  |  |  |  |  |  |  |  |  |  |  |  |  |  |  |  |  |  |  |  |  |  |  |  |  |  |  |  |  |  |  |  |  |  |  |  |  |  |
| AUSTRALIA | 128 | 5062912 | happy | tender | intermate | tender | Irritated | energetic | happy | antsy |  | 3 |  | 1 |  | 7 |  | 7 |  | 4 |  | 3 |  | 3 |  | 3 |  |  |  |  |  |  |  |  |  |  |  |  |  |  |  |  |  |  |  |  |  |  |  |  |  |  |  |  |  |  |  |  |  |  |  |  |  |  |  |  |  |  |  |  |  |  |  |  |  |  |  |
| AUSTRALIA | 129 | z5060369 | energetic | love | excited | intrigued | weird | uncomfortable | happy | calm |  | 5 |  | 5 |  | 3 |  | 3 |  | 2 |  | 7 |  | 5 |  | 5 |  |  |  |  |  |  |  |  |  |  |  |  |  |  |  |  |  |  |  |  |  |  |  |  |  |  |  |  |  |  |  |  |  |  |  |  |  |  |  |  |  |  |  |  |  |  |  |  |  |  |  |
| AUSTRALIA | 130 | 5062194 | Joy | Calm | Happy | Confusion | Angry | Excited | Bored | Motivated |  | 5 |  | 2 |  | 3 |  | 7 |  | 8 |  | 7 |  | 8 |  | 8 |  |  |  |  |  |  |  |  |  |  |  |  |  |  |  |  |  |  |  |  |  |  |  |  |  |  |  |  |  |  |  |  |  |  |  |  |  |  |  |  |  |  |  |  |  |  |  |  |  |  |  |
| AUSTRALIA | 133 | z5133205 | sleepy | boring | funny | peaceful | joyful | relaxing | happy | relaxing |  | 2 |  | 5 |  | 6 |  | 6 |  | 4 |  | 5 |  | 5 |  | 4 |  |  |  |  |  |  |  |  |  |  |  |  |  |  |  |  |  |  |  |  |  |  |  |  |  |  |  |  |  |  |  |  |  |  |  |  |  |  |  |  |  |  |  |  |  |  |  |  |  |  |  |
| AUSTRALIA | 134 | z5017802 | Festive, energetic | Calm | Festive | Solemn | Aggression | Frustration | Happy | Powerful |  | 1 |  | 6 |  | 6 |  | 5 |  | 1 |  | 5 |  | 1 |  | 1 |  |  |  |  |  |  |  |  |  |  |  |  |  |  |  |  |  |  |  |  |  |  |  |  |  |  |  |  |  |  |  |  |  |  |  |  |  |  |  |  |  |  |  |  |  |  |  |  |  |  |  |
| AUSTRALIA | 135 | 5118393 | energetic | calm | calm | curious | scared | powerul | bright | calm |  | 6 |  | 1 |  | 4 |  | 7 |  | 8 |  | 6 |  | 5 |  | 5 |  |  |  |  |  |  |  |  |  |  |  |  |  |  |  |  |  |  |  |  |  |  |  |  |  |  |  |  |  |  |  |  |  |  |  |  |  |  |  |  |  |  |  |  |  |  |  |  |  |  |  |
| AUSTRALIA | 136 | z3416693 | light hearted | dreamy | relaxed | nostalgic | confused | energetic | happy | calm |  | 5 |  | 1 |  | 7 |  | 7 |  | 5 |  | 4 |  | 6 |  | 6 |  |  |  |  |  |  |  |  |  |  |  |  |  |  |  |  |  |  |  |  |  |  |  |  |  |  |  |  |  |  |  |  |  |  |  |  |  |  |  |  |  |  |  |  |  |  |  |  |  |  |  |
| AUSTRALIA | 138 | 5118018 | Mild excitement | Sadness | Slight sadness | Calm | Anger | Emotionless | Calm | Anticipation |  | 1 |  | 1 |  | 1 |  | 1 |  | 1 |  | 1 |  | 1 |  | 1 |  |  |  |  |  |  |  |  |  |  |  |  |  |  |  |  |  |  |  |  |  |  |  |  |  |  |  |  |  |  |  |  |  |  |  |  |  |  |  |  |  |  |  |  |  |  |  |  |  |  |  |
| AUSTRALIA | 139 | z5057415 | happy | upset | happy | peaceful | angry | angry | relaxed | calm |  | 5 |  | 5 |  | 6 |  | 6 |  | 7 |  | 7 |  | 4 |  | 4 |  |  |  |  |  |  |  |  |  |  |  |  |  |  |  |  |  |  |  |  |  |  |  |  |  |  |  |  |  |  |  |  |  |  |  |  |  |  |  |  |  |  |  |  |  |  |  |  |  |  |  |
| AUSTRALIA | 140 | 3466532 | comfortable | relax | happy | strange | anxious | excited | happy | relax |  | 4 |  | 8 |  | 7 |  | 7 |  | 6 |  | 7 |  | 8 |  | 5 |  |  |  |  |  |  |  |  |  |  |  |  |  |  |  |  |  |  |  |  |  |  |  |  |  |  |  |  |  |  |  |  |  |  |  |  |  |  |  |  |  |  |  |  |  |  |  |  |  |  |  |
| AUSTRALIA | 142 | z3461130 | connected | relaxed | carefree | tranquil | frustration | upbeat | joy | aware |  | 2 |  | 1 |  | 7 |  | 6 |  | 1 |  | 1 |  | 1 |  | 1 |  |  |  |  |  |  |  |  |  |  |  |  |  |  |  |  |  |  |  |  |  |  |  |  |  |  |  |  |  |  |  |  |  |  |  |  |  |  |  |  |  |  |  |  |  |  |  |  |  |  |  |
| AUSTRALIA | 143 | 3509020 | Joy and energy | Relaxed | Festive (the first piece)  Relaxed (the second one) | Confusion | Couldn't finish the piece, it really annoys me | it makes me feel like dancing to their rythm | careless, positive | Dreamy and disconnected | 2 |  | 1 |  | 6 |  | 4 |  | 1 |  | 5 |  | 4 |  | 6 |  |  |  |  |  |  |  |  |  |  |  |  |  |  |  |  |  |  |  |  |  |  |  |  |  |  |  |  |  |  |  |  |  |  |  |  |  |  |  |  |  |  |  |  |  |  |  |  |  |  |  |  |
| AUSTRALIA | 144 | z5150386 | excited | sadness | happiness | weirdness | disgust | frustration | excitement | apprehensive | 8 |  | 1 |  | 5 |  | 5 |  | 8 |  | 5 |  | 2 |  | 8 |  |  |  |  |  |  |  |  |  |  |  |  |  |  |  |  |  |  |  |  |  |  |  |  |  |  |  |  |  |  |  |  |  |  |  |  |  |  |  |  |  |  |  |  |  |  |  |  |  |  |  |  |
| AUSTRALIA | 147 | z5075942 | dance | longing | high-spirited | unfamiliar | irritated | motivated | happy | relaxed |  | 1 |  | 1 |  | 7 |  | 6 |  | 1 |  | 6 |  | 1 |  | 1 |  |  |  |  |  |  |  |  |  |  |  |  |  |  |  |  |  |  |  |  |  |  |  |  |  |  |  |  |  |  |  |  |  |  |  |  |  |  |  |  |  |  |  |  |  |  |  |  |  |  |  |
| AUSTRALIA | 149 | z5060526 | excited | relaxed | dancelike | relaxed | angry | calm, not excited | excited, energized | excited |  | 6 |  | 2 |  | 7 |  | 3 |  | 4 |  | 7 |  | 5 |  | 6 |  |  |  |  |  |  |  |  |  |  |  |  |  |  |  |  |  |  |  |  |  |  |  |  |  |  |  |  |  |  |  |  |  |  |  |  |  |  |  |  |  |  |  |  |  |  |  |  |  |  |  |
| AUSTRALIA | 150 | z5076068 | relaxing | relaxed | groovy | calm | intense | dancing | dancing | calm |  | 8 |  | 1 |  | 4 |  | 4 |  | 8 |  | 8 |  | 8 |  | 8 |  |  |  |  |  |  |  |  |  |  |  |  |  |  |  |  |  |  |  |  |  |  |  |  |  |  |  |  |  |  |  |  |  |  |  |  |  |  |  |  |  |  |  |  |  |  |  |  |  |  |  |
| AUSTRALIA | 151 | z5061982 | Sultry | Melancholy | Sensual | Meditative | Angry | Struggle | Light hearted | Intellectual |  | 8 |  | 1 |  | 7 |  | 5 |  | 8 |  | 3 |  | 8 |  | 8 |  |  |  |  |  |  |  |  |  |  |  |  |  |  |  |  |  |  |  |  |  |  |  |  |  |  |  |  |  |  |  |  |  |  |  |  |  |  |  |  |  |  |  |  |  |  |  |  |  |  |  |
| AUSTRALIA | 152 | z5151698 | calm | tranquility | amused | amused | off-guard | excited | happy | indifferent |  | 2 |  | 1 |  | 7 |  | 7 |  | 1 |  | 1 |  | 8 |  | 5 |  |  |  |  |  |  |  |  |  |  |  |  |  |  |  |  |  |  |  |  |  |  |  |  |  |  |  |  |  |  |  |  |  |  |  |  |  |  |  |  |  |  |  |  |  |  |  |  |  |  |  |
| AUSTRALIA | 153 | 5150660 | Loud | Peaceful | Foreign | Confused | Annoyed | Gangster | I get excited | Movie music | 1 |  | 1 |  | 6 |  | 4 |  | 1 |  | 5 |  | 1 |  | 1 |  |  |  |  |  |  |  |  |  |  |  |  |  |  |  |  |  |  |  |  |  |  |  |  |  |  |  |  |  |  |  |  |  |  |  |  |  |  |  |  |  |  |  |  |  |  |  |  |  |  |  |  |
| AUSTRALIA | 154 | 3462165 | cruising | calm | sassy | calm | irritated | Chill | no emotion | happy, calm | 8 |  | 1 |  | 4 |  | 7 |  | 6 |  | 7 |  | 6 |  | 8 |  |  |  |  |  |  |  |  |  |  |  |  |  |  |  |  |  |  |  |  |  |  |  |  |  |  |  |  |  |  |  |  |  |  |  |  |  |  |  |  |  |  |  |  |  |  |  |  |  |  |  |  |
| AUSTRALIA | 156 | z5057309 | relax | little sad | relax | relax | exciting | exciting | happy | fantastic |  | 2 |  | 5 |  | 7 |  | 2 |  | 2 |  | 2 |  | 2 |  | 2 |  |  |  |  |  |  |  |  |  |  |  |  |  |  |  |  |  |  |  |  |  |  |  |  |  |  |  |  |  |  |  |  |  |  |  |  |  |  |  |  |  |  |  |  |  |  |  |  |  |  |  |
| AUSTRALIA | 158 | 5062194 | Happy | Sad | Happy | Confused | Angry | Excited | Bored | Motivated |  | 8 |  | 2 |  | 5 |  | 7 |  | 8 |  | 7 |  | 8 |  | 8 |  |  |  |  |  |  |  |  |  |  |  |  |  |  |  |  |  |  |  |  |  |  |  |  |  |  |  |  |  |  |  |  |  |  |  |  |  |  |  |  |  |  |  |  |  |  |  |  |  |  |  |
| AUSTRALIA | 159 | z5057309 | happy | relax | happy | relax | exciting | exciting | exciting | joy |  | 2 |  | 5 |  | 7 |  | 5 |  | 1 |  | 1 |  | 1 |  | 1 |  |  |  |  |  |  |  |  |  |  |  |  |  |  |  |  |  |  |  |  |  |  |  |  |  |  |  |  |  |  |  |  |  |  |  |  |  |  |  |  |  |  |  |  |  |  |  |  |  |  |  |
| AUSTRALIA | 160 | 5075619 | excitement | calmness | calmness | Calmness | overwhelmed | exitement | Happy | calmness |  | 4 |  | 1 |  | 7 |  | 7 |  | 8 |  | 5 |  | 8 |  | 8 |  |  |  |  |  |  |  |  |  |  |  |  |  |  |  |  |  |  |  |  |  |  |  |  |  |  |  |  |  |  |  |  |  |  |  |  |  |  |  |  |  |  |  |  |  |  |  |  |  |  |  |
| AUSTRALIA | 161 | z5150292 | Relaxed | Calmness | Relaxed | Curiosity | Anger | Confidence | Boredom | Elevation |  | 1 |  | 3 |  | 3 |  | 6 |  | 1 |  | 6 |  | 1 |  | 1 |  |  |  |  |  |  |  |  |  |  |  |  |  |  |  |  |  |  |  |  |  |  |  |  |  |  |  |  |  |  |  |  |  |  |  |  |  |  |  |  |  |  |  |  |  |  |  |  |  |  |  |
| AUSTRALIA | 162 | z3374318 | Romantic | Romantic | Tense | Tense | Angsty | Cringe | Uninspired | Epiphany |  | 5 |  | 2 |  | 7 |  | 7 |  | 5 |  | 4 |  | 5 |  | 5 |  |  |  |  |  |  |  |  |  |  |  |  |  |  |  |  |  |  |  |  |  |  |  |  |  |  |  |  |  |  |  |  |  |  |  |  |  |  |  |  |  |  |  |  |  |  |  |  |  |  |  |
| AUSTRALIA | 163 | z5097497 | I feel relaxed. I feel like dancing. I feel like I'm in love. | I feel relaxed | I feel like I'm in love. I feel like dancing. It is a little bit the same as Brazilian Samba. I feel like i wanted to dance. | I feel confused | I feel dangerous. I feel energetic. I feel I wanted to punch people in the face. i feel happy | I feel like dancing. I feel dangerous. I feel like I've been smoking weed. I feel like I am a gangster | Pop music does not really evoke my emotion. I don't feel anything listening to the music. Probably a little bit bored | I feel relaxed. I feel rich. I feel like I'm in a deep thought | 5 |  | 6 |  | 2 |  | 6 |  | 2 |  | 4 |  | 3 |  | 5 |  |  |  |  |  |  |  |  |  |  |  |  |  |  |  |  |  |  |  |  |  |  |  |  |  |  |  |  |  |  |  |  |  |  |  |  |  |  |  |  |  |  |  |  |  |  |  |  |  |  |  |  |
| AUSTRALIA | 164 | z5039171 | Nostalgia | Wistfulness | Happiness | Excitement | Anger | Disgust | Contempt | Boredom |  | 4 |  | 6 |  | 2 |  | 1 |  | 5 |  | 3 |  | 8 |  | 5 |  |  |  |  |  |  |  |  |  |  |  |  |  |  |  |  |  |  |  |  |  |  |  |  |  |  |  |  |  |  |  |  |  |  |  |  |  |  |  |  |  |  |  |  |  |  |  |  |  |  |  |
| AUSTRALIA | 165 | 5113129 | walking through markets. cooking | relaxed | dancing | dreaming | fighting | cool | dancing | floating |  | 4 |  | 2 |  | 4 |  | 7 |  | 3 |  | 3 |  | 4 |  | 4 |  |  |  |  |  |  |  |  |  |  |  |  |  |  |  |  |  |  |  |  |  |  |  |  |  |  |  |  |  |  |  |  |  |  |  |  |  |  |  |  |  |  |  |  |  |  |  |  |  |  |  |
| AUSTRALIA | 166 | z5057358 | cheerful | sadness | noisy | like chanting | heavy rhythm | strange | quite loudly | immersive |  | 8 |  | 2 |  | 2 |  | 4 |  | 8 |  | 1 |  | 8 |  | 8 |  |  |  |  |  |  |  |  |  |  |  |  |  |  |  |  |  |  |  |  |  |  |  |  |  |  |  |  |  |  |  |  |  |  |  |  |  |  |  |  |  |  |  |  |  |  |  |  |  |  |  |
| AUSTRALIA | 167 | z5113226 | zest | longing | sensuality | serenity | anger | vanity | elation | optimism |  | 2 |  | 1 |  | 5 |  | 4 |  | 4 |  | 5 |  | 2 |  | 2 |  |  |  |  |  |  |  |  |  |  |  |  |  |  |  |  |  |  |  |  |  |  |  |  |  |  |  |  |  |  |  |  |  |  |  |  |  |  |  |  |  |  |  |  |  |  |  |  |  |  |  |
| AUSTRALIA | 168 | z5056121 | light | soothing | relaxed | exotic | passionate | rhythmed | young | formal |  | 2 |  | 3 |  | 7 |  | 7 |  | 8 |  | 4 |  | 8 |  | 8 |  |  |  |  |  |  |  |  |  |  |  |  |  |  |  |  |  |  |  |  |  |  |  |  |  |  |  |  |  |  |  |  |  |  |  |  |  |  |  |  |  |  |  |  |  |  |  |  |  |  |  |
| AUSTRALIA | 170 | z5106796 | bouncy | passion | fun | anticipation | distressed | chill | fun | full |  | 5 |  | 1 |  | 5 |  | 7 |  | 4 |  | 6 |  | 5 |  | 5 |  |  |  |  |  |  |  |  |  |  |  |  |  |  |  |  |  |  |  |  |  |  |  |  |  |  |  |  |  |  |  |  |  |  |  |  |  |  |  |  |  |  |  |  |  |  |  |  |  |  |  |
| AUSTRALIA | 171 | z3464751 | Uplifted | Humble | passionate | Curious | Aggressive | Excited | pretentious | MeloDramatic | 4 |  | 5 |  | 4 |  | 5 |  | 5 |  | 4 |  | 4 |  | 4 |  |  |  |  |  |  |  |  |  |  |  |  |  |  |  |  |  |  |  |  |  |  |  |  |  |  |  |  |  |  |  |  |  |  |  |  |  |  |  |  |  |  |  |  |  |  |  |  |  |  |  |  |
| AUSTRALIA | 172 | Z5109946 | first one is relaxing  second one is happy | relaxing | pub music | relaxing | hype | hype | exciting | relaxing |  | 5 |  | 5 |  | 5 |  | 5 |  | 5 |  | 5 |  | 5 |  | 5 |  |  |  |  |  |  |  |  |  |  |  |  |  |  |  |  |  |  |  |  |  |  |  |  |  |  |  |  |  |  |  |  |  |  |  |  |  |  |  |  |  |  |  |  |  |  |  |  |  |  |  |
| AUSTRALIA | 173 | 5154954 | straight | great | great | crazy | noise | great | good | wonderful |  | 4 |  | 3 |  | 7 |  | 6 |  | 5 |  | 7 |  | 4 |  | 4 |  |  |  |  |  |  |  |  |  |  |  |  |  |  |  |  |  |  |  |  |  |  |  |  |  |  |  |  |  |  |  |  |  |  |  |  |  |  |  |  |  |  |  |  |  |  |  |  |  |  |  |
| AUSTRALIA | 174 | z5077187 | Neutral | Relax | Happy | Neutral | Cool | Cool | Chill | Relax |  | 3 |  | 3 |  | 7 |  | 6 |  | 3 |  | 7 |  | 3 |  | 3 |  |  |  |  |  |  |  |  |  |  |  |  |  |  |  |  |  |  |  |  |  |  |  |  |  |  |  |  |  |  |  |  |  |  |  |  |  |  |  |  |  |  |  |  |  |  |  |  |  |  |  |
| AUSTRALIA | 175 | 123456 | excited | happy | pumped | chill | angry | angry | excited | chill |  | 4 |  | 4 |  | 4 |  | 4 |  | 4 |  | 4 |  | 4 |  | 4 |  |  |  |  |  |  |  |  |  |  |  |  |  |  |  |  |  |  |  |  |  |  |  |  |  |  |  |  |  |  |  |  |  |  |  |  |  |  |  |  |  |  |  |  |  |  |  |  |  |  |  |
| AUSTRALIA | 176 | 5118091 | cheerful | calm | joy | nervousness | Fear | disgust | Happy | Intense |  | 8 |  | 1 |  | 7 |  | 2 |  | 8 |  | 6 |  | 8 |  | 8 |  |  |  |  |  |  |  |  |  |  |  |  |  |  |  |  |  |  |  |  |  |  |  |  |  |  |  |  |  |  |  |  |  |  |  |  |  |  |  |  |  |  |  |  |  |  |  |  |  |  |  |
| AUSTRALIA | 177 | z5157185 | Energized | Happy/content | Impressed | Curious | Scared | Calm | Bored | Enjoyment |  | 7 |  | 2 |  | 4 |  | 7 |  | 3 |  | 5 |  | 7 |  | 7 |  |  |  |  |  |  |  |  |  |  |  |  |  |  |  |  |  |  |  |  |  |  |  |  |  |  |  |  |  |  |  |  |  |  |  |  |  |  |  |  |  |  |  |  |  |  |  |  |  |  |  |
| AUSTRALIA | 178 | z3485826 | Lust | Romantic | Love | relaxation | Pumped | Angst | relaxation | anticipation |  | 7 |  | 7 |  | 4 |  | 1 |  | 7 |  | 7 |  | 7 |  | 7 |  |  |  |  |  |  |  |  |  |  |  |  |  |  |  |  |  |  |  |  |  |  |  |  |  |  |  |  |  |  |  |  |  |  |  |  |  |  |  |  |  |  |  |  |  |  |  |  |  |  |  |
| AUSTRALIA | 179 | 5076872 | cool | calm | Dance | meditative | anger | groove | happy | intelligent |  | 3 |  | 4 |  | 7 |  | 6 |  | 1 |  | 5 |  | 1 |  | 3 |  |  |  |  |  |  |  |  |  |  |  |  |  |  |  |  |  |  |  |  |  |  |  |  |  |  |  |  |  |  |  |  |  |  |  |  |  |  |  |  |  |  |  |  |  |  |  |  |  |  |  |
| AUSTRALIA | 180 | z5020625 | Nostalgia | Nostalgia | Nostalgia | Chilled | Anger | Chilled | Happiness | Happiness |  | 1 |  | 2 |  | 5 |  | 6 |  | 1 |  | 3 |  | 1 |  | 1 |  |  |  |  |  |  |  |  |  |  |  |  |  |  |  |  |  |  |  |  |  |  |  |  |  |  |  |  |  |  |  |  |  |  |  |  |  |  |  |  |  |  |  |  |  |  |  |  |  |  |  |
| AUSTRALIA | 181 | 3373251 | calm | sentimental | excitement | confused | irritated | no emotions | no emotions | touched |  | 8 |  | 1 |  | 7 |  | 5 |  | 4 |  | 7 |  | 4 |  | 4 |  |  |  |  |  |  |  |  |  |  |  |  |  |  |  |  |  |  |  |  |  |  |  |  |  |  |  |  |  |  |  |  |  |  |  |  |  |  |  |  |  |  |  |  |  |  |  |  |  |  |  |
| AUSTRALIA | 182 | z5076351 | Happy | jnj | Groovey | Angry | LOL | badass | annoyed | Calm |  | 1 |  | 1 |  | 1 |  | 1 |  | 1 |  | 1 |  | 1 |  | 1 |  |  |  |  |  |  |  |  |  |  |  |  |  |  |  |  |  |  |  |  |  |  |  |  |  |  |  |  |  |  |  |  |  |  |  |  |  |  |  |  |  |  |  |  |  |  |  |  |  |  |  |
| AUSTRALIA | 184 | 3420952 | hungry | sad | energetic | confused | tired | soulful | happy | musical |  | 2 |  | 2 |  | 5 |  | 3 |  | 2 |  | 5 |  | 2 |  | 2 |  |  |  |  |  |  |  |  |  |  |  |  |  |  |  |  |  |  |  |  |  |  |  |  |  |  |  |  |  |  |  |  |  |  |  |  |  |  |  |  |  |  |  |  |  |  |  |  |  |  |  |
| AUSTRALIA | 185 | 5060869 | Romance | Exoticism | Tragedy | Seriousness | Anger | Confidence | Fun | Superiority |  | 1 |  | 5 |  | 3 |  | 2 |  | 1 |  | 5 |  | 1 |  | 1 |  |  |  |  |  |  |  |  |  |  |  |  |  |  |  |  |  |  |  |  |  |  |  |  |  |  |  |  |  |  |  |  |  |  |  |  |  |  |  |  |  |  |  |  |  |  |  |  |  |  |  |
| AUSTRALIA | 186 | z3465132 | Lively | Relaxed | Chill | Meditative | Aggravated | Agitated | Energised | Calm |  | 6 |  | 3 |  | 7 |  | 3 |  | 6 |  | 7 |  | 6 |  | 3 |  |  |  |  |  |  |  |  |  |  |  |  |  |  |  |  |  |  |  |  |  |  |  |  |  |  |  |  |  |  |  |  |  |  |  |  |  |  |  |  |  |  |  |  |  |  |  |  |  |  |  |
| AUSTRALIA | 187 | 5114041 | chaotic | mellow | groovy | unfamiliar | active | active | excited | neutral |  | 4 |  | 4 |  | 7 |  | 7 |  | 8 |  | 4 |  | 8 |  | 8 |  |  |  |  |  |  |  |  |  |  |  |  |  |  |  |  |  |  |  |  |  |  |  |  |  |  |  |  |  |  |  |  |  |  |  |  |  |  |  |  |  |  |  |  |  |  |  |  |  |  |  |
| AUSTRALIA | 188 | z3484164 | Romantic | Longing | Romantic | Eerie | Scared | Confident | Groovy | Dramatic |  | 6 |  | 1 |  | 7 |  | 7 |  | 2 |  | 6 |  | 5 |  | 5 |  |  |  |  |  |  |  |  |  |  |  |  |  |  |  |  |  |  |  |  |  |  |  |  |  |  |  |  |  |  |  |  |  |  |  |  |  |  |  |  |  |  |  |  |  |  |  |  |  |  |  |
| AUSTRALIA | 189 | z3413806 | Old | sweet | funky | deceit | angry | cool | modern | mystery |  | 4 |  | 1 |  | 7 |  | 7 |  | 2 |  | 7 |  | 3 |  | 1 |  |  |  |  |  |  |  |  |  |  |  |  |  |  |  |  |  |  |  |  |  |  |  |  |  |  |  |  |  |  |  |  |  |  |  |  |  |  |  |  |  |  |  |  |  |  |  |  |  |  |  |
| AUSTRALIA | 190 | 5164833 | asdf | gud | asdf |  | asdf | asdf | asdf | asdf |  |  |  |  |  |  |  |  |  |  |  |  |  |  |  |  |  |  |  |  |  |  |  |  |  |  |  |  |  |  |  |  |  |  |  |  |  |  |  |  |  |  |  |  |  |  |  |  |  |  |  |  |  |  |  |  |  |  |  |  |  |  |  |  |  |  |  |
| AUSTRALIA | 191 | 5060478 | alertness | calmness | alertness | tense | annoyance | tension | Happiness | Clamness |  | 6 |  | 1 |  | 5 |  | 5 |  | 5 |  | 6 |  | 5 |  | 5 |  |  |  |  |  |  |  |  |  |  |  |  |  |  |  |  |  |  |  |  |  |  |  |  |  |  |  |  |  |  |  |  |  |  |  |  |  |  |  |  |  |  |  |  |  |  |  |  |  |  |  |
| AUSTRALIA | 192 | z5151391 | Relaxed,positive | Feels like I just broke up | Calming | Uncomfortable | Awoken | Pumped | groovy | feels like I am watching a royal gathering | 4 |  | 4 |  | 5 |  | 8 |  | 1 |  | 3 |  | 3 |  | 4 |  |  |  |  |  |  |  |  |  |  |  |  |  |  |  |  |  |  |  |  |  |  |  |  |  |  |  |  |  |  |  |  |  |  |  |  |  |  |  |  |  |  |  |  |  |  |  |  |  |  |  |  |
| AUSTRALIA | 193 | z5046509 | happy | nostalgic | happy | relaxed | scary | motivation | determined | bored |  | 3 |  | 1 |  | 5 |  | 5 |  | 3 |  | 3 |  | 5 |  | 3 |  |  |  |  |  |  |  |  |  |  |  |  |  |  |  |  |  |  |  |  |  |  |  |  |  |  |  |  |  |  |  |  |  |  |  |  |  |  |  |  |  |  |  |  |  |  |  |  |  |  |  |
| AUSTRALIA | 194 | z5063196 | happiness | calmness | dancing | calmness | happiness | laidbackness | boredom | calmness |  | 3 |  | 7 |  | 3 |  | 3 |  | 3 |  | 7 |  | 3 |  | 3 |  |  |  |  |  |  |  |  |  |  |  |  |  |  |  |  |  |  |  |  |  |  |  |  |  |  |  |  |  |  |  |  |  |  |  |  |  |  |  |  |  |  |  |  |  |  |  |  |  |  |  |
| AUSTRALIA | 197 | 5080369 | lively | complicate | operatic | classical | strong | rhythm sensation | excited | fantasy |  | 2 |  | 2 |  | 6 |  | 6 |  | 4 |  | 4 |  | 4 |  | 4 |  |  |  |  |  |  |  |  |  |  |  |  |  |  |  |  |  |  |  |  |  |  |  |  |  |  |  |  |  |  |  |  |  |  |  |  |  |  |  |  |  |  |  |  |  |  |  |  |  |  |  |
| AUSTRALIA | 198 | z3489357 | relaxing | expressive | uplifting | meditative | grudge | expressive | fun | intense |  | 5 |  | 3 |  | 6 |  | 6 |  | 2 |  | 7 |  | 5 |  | 6 |  |  |  |  |  |  |  |  |  |  |  |  |  |  |  |  |  |  |  |  |  |  |  |  |  |  |  |  |  |  |  |  |  |  |  |  |  |  |  |  |  |  |  |  |  |  |  |  |  |  |  |
| AUSTRALIA | 202 | z5062565 | Excited | Heartbroken | Amused | Unsure | Energised | Rageful | Bored | Sophisticated | 5 |  | 7 |  | 6 |  | 6 |  | 3 |  | 6 |  | 5 |  | 5 |  |  |  |  |  |  |  |  |  |  |  |  |  |  |  |  |  |  |  |  |  |  |  |  |  |  |  |  |  |  |  |  |  |  |  |  |  |  |  |  |  |  |  |  |  |  |  |  |  |  |  |  |
| AUSTRALIA | 203 | z5020459 | Excited | melancholy | Relaxed | Peace | Fear | Empowered | Excited | Calm |  | 5 |  | 1 |  | 7 |  | 7 |  | 4 |  | 7 |  | 5 |  | 5 |  |  |  |  |  |  |  |  |  |  |  |  |  |  |  |  |  |  |  |  |  |  |  |  |  |  |  |  |  |  |  |  |  |  |  |  |  |  |  |  |  |  |  |  |  |  |  |  |  |  |  |
| AUSTRALIA | 204 | 3332513 | joyful | inspired | excited | Indifferent | Rage | provocative | playful | ecstatic |  | 5 |  | 2 |  | 6 |  | 7 |  | 2 |  | 6 |  | 6 |  | 6 |  |  |  |  |  |  |  |  |  |  |  |  |  |  |  |  |  |  |  |  |  |  |  |  |  |  |  |  |  |  |  |  |  |  |  |  |  |  |  |  |  |  |  |  |  |  |  |  |  |  |  |
| AUSTRALIA | 205 | z5062565 | Excited | Heartbroken | Relaxed | Travelled | Energised | Uneasy | Unsure | Sophisticated | 6 |  | 7 |  | 6 |  | 6 |  | 4 |  | 5 |  | 5 |  | 5 |  |  |  |  |  |  |  |  |  |  |  |  |  |  |  |  |  |  |  |  |  |  |  |  |  |  |  |  |  |  |  |  |  |  |  |  |  |  |  |  |  |  |  |  |  |  |  |  |  |  |  |  |
| AUSTRALIA | 207 | z5083730 | enthusiasm | melancholy | lively | strange | uncomfortable | rhythm | cheerful | solemn |  | 4 |  | 2 |  | 5 |  | 5 |  | 3 |  | 4 |  | 4 |  | 4 |  |  |  |  |  |  |  |  |  |  |  |  |  |  |  |  |  |  |  |  |  |  |  |  |  |  |  |  |  |  |  |  |  |  |  |  |  |  |  |  |  |  |  |  |  |  |  |  |  |  |  |
| AUSTRALIA | 209 | 5055993 | delight | sad | relax | lonely | Noisy | excited | lively | peaceful |  | 2 |  | 1 |  | 7 |  | 5 |  | 4 |  | 6 |  | 3 |  | 8 |  |  |  |  |  |  |  |  |  |  |  |  |  |  |  |  |  |  |  |  |  |  |  |  |  |  |  |  |  |  |  |  |  |  |  |  |  |  |  |  |  |  |  |  |  |  |  |  |  |  |  |
| AUSTRALIA | 213 | z3417645 | relaxed | love | love | excited | angry | chill | happy | tragic |  | 4 |  | 1 |  | 7 |  | 7 |  | 4 |  | 5 |  | 6 |  | 4 |  |  |  |  |  |  |  |  |  |  |  |  |  |  |  |  |  |  |  |  |  |  |  |  |  |  |  |  |  |  |  |  |  |  |  |  |  |  |  |  |  |  |  |  |  |  |  |  |  |  |  |
| AUSTRALIA | 219 | z5014870 | relaxed | Jovial | relaxed | nostalgic | uncomfortable | uncomfortable | excited | relaxed |  | 4 |  | 1 |  | 6 |  | 6 |  | 6 |  | 7 |  | 8 |  | 8 |  |  |  |  |  |  |  |  |  |  |  |  |  |  |  |  |  |  |  |  |  |  |  |  |  |  |  |  |  |  |  |  |  |  |  |  |  |  |  |  |  |  |  |  |  |  |  |  |  |  |  |
| AUSTRALIA | 220 | z5118730 | Jolly | Love | Romantic | Calmness | Energy and excitement | Groovy | Casual | Immersiveness | 5 |  | 7 |  | 3 |  | 7 |  | 7 |  | 7 |  | 4 |  | 4 |  |  |  |  |  |  |  |  |  |  |  |  |  |  |  |  |  |  |  |  |  |  |  |  |  |  |  |  |  |  |  |  |  |  |  |  |  |  |  |  |  |  |  |  |  |  |  |  |  |  |  |  |
| AUSTRALIA | 221 | z5015219 | slightly irritated because of the minor keys | distaste | interest | wonder | impressed but intensity | relaxed | frustrated | Appreciation | 3 |  | 2 |  | 5 |  | 6 |  | 5 |  | 5 |  | 2 |  | 2 |  |  |  |  |  |  |  |  |  |  |  |  |  |  |  |  |  |  |  |  |  |  |  |  |  |  |  |  |  |  |  |  |  |  |  |  |  |  |  |  |  |  |  |  |  |  |  |  |  |  |  |  |
| AUSTRALIA | 222 | 5153368 | Happy | Calm | Relaxed | Traditional | Angry | Rebellious | Relaxed | Royal |  | 6 |  | 2 |  | 7 |  | 7 |  | 3 |  | 2 |  | 5 |  | 5 |  |  |  |  |  |  |  |  |  |  |  |  |  |  |  |  |  |  |  |  |  |  |  |  |  |  |  |  |  |  |  |  |  |  |  |  |  |  |  |  |  |  |  |  |  |  |  |  |  |  |  |
| AUSTRALIA | 223 | z5076887 | relaxed. | sad | happy | reflective | Energetic | groovy | annoyed | tender |  | 5 |  | 6 |  | 4 |  | 6 |  | 6 |  | 7 |  | 6 |  | 6 |  |  |  |  |  |  |  |  |  |  |  |  |  |  |  |  |  |  |  |  |  |  |  |  |  |  |  |  |  |  |  |  |  |  |  |  |  |  |  |  |  |  |  |  |  |  |  |  |  |  |  |
| AUSTRALIA | 224 | 5062348 | excited | fun | fun | home | weird | hip | fine | posh |  | 4 |  | 4 |  | 4 |  | 4 |  | 4 |  | 4 |  | 4 |  | 4 |  |  |  |  |  |  |  |  |  |  |  |  |  |  |  |  |  |  |  |  |  |  |  |  |  |  |  |  |  |  |  |  |  |  |  |  |  |  |  |  |  |  |  |  |  |  |  |  |  |  |  |
| AUSTRALIA | 227 | z5129813 | relaxed | relaxed | content | Naivity | anger | happy | energised | determination | 5 |  | 1 |  | 5 |  | 7 |  | 5 |  | 5 |  | 5 |  | 5 |  |  |  |  |  |  |  |  |  |  |  |  |  |  |  |  |  |  |  |  |  |  |  |  |  |  |  |  |  |  |  |  |  |  |  |  |  |  |  |  |  |  |  |  |  |  |  |  |  |  |  |  |
|  |  |  |  |  |  |  |  |  |  |  |  |  |  |  |  |  |  |  |  |  |  |  |  |  |  |  |  |  |  |  |  |  |  |  |  |  |  |  |  |  |  |  |  |  |  |  |  |  |  |  |  |  |  |  |  |  |  |  |  |  |  |  |  |  |  |  |  |  |  |  |  |  |  |  |  |  |  |
